# Investigating Bay-Substituted 6,7-Dihydrodibenzo[*b,j*][4,7]phenanthroline, a Class of Tunable Hindered Rotors: Synthesis and Molecular Dynamics

**DOI:** 10.1021/acs.joc.4c01476

**Published:** 2025-06-03

**Authors:** Yen-Cheng Lu, Jhih-Syong Jhang, Chih-Hsiu Lin

**Affiliations:** † Institute of Chemistry, 38017 Academia Sinica, Taipei 115024, Taiwan, Republic of China; ‡ Department of Chemistry, National Central University, Taoyuan 320317, Taiwan, Republic of China

## Abstract

We developed a convenient and flexible synthesis of 6,7-dihydrodibenzo­[*b,j*]­[4,7]­phenanthroline derivatives with bay region substituents via Friedländer condensation. Thanks to the mild reaction conditions, unsymmetrical bay-substituted systems also become accessible. The rigid phenanthroline framework holds various functional groups in the bay region in enough proximity for intramolecular interactions to manifest. Measuring the hindered rotational motions in this system unveils subtle structure–property relationships. Most notably, through rotamer distribution, π–π interactions between polycyclic aromatic hydrocarbons were found to scale with the π-surface coverage areas.

## Graphical abstract

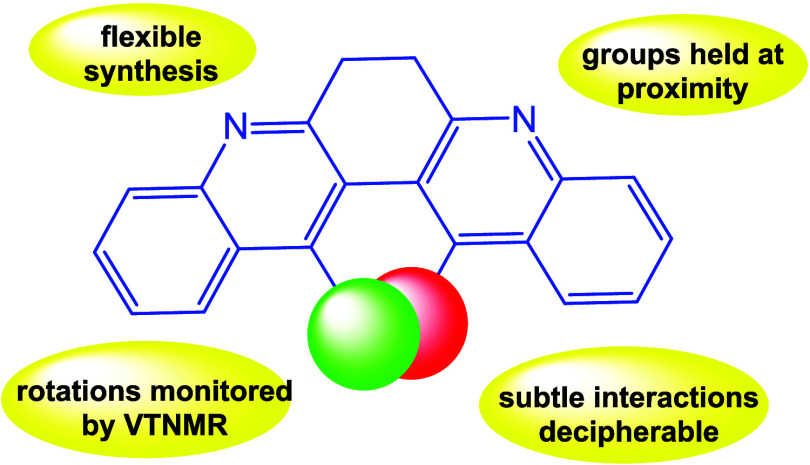

## Introduction

Noncovalent interaction between aromatic groups^1^ is the force that governs many phenomena in biology and material science, including protein folding,^2^ drug–protein docking,^3^ and charge transfer in the condensed phase.^4^ The interaction between π-systems is comprised of electrostatic, dispersion, and solvophobic components,^5^ each having a different dependency on distance and geometry. Therefore, it is a great challenge to interpret, predict, and engineer aromatic interactions. Aryl-spacer-aryl triad molecules were constructed to understand and decipher such a heterogeneous interaction. The ideal spacer should be rigid to hold the aryl moieties apart at a reachable distance. Meanwhile, the spacer must also be flexible enough to accommodate conformation switches and rotations that enable the association and dissociation of the aryl moieties. The challenge in this endeavor is to balance the opposite requirements, yet keep the synthesis straightforward and adaptable.

The zigzag and bay edges of polycyclic aromatic hydrocarbons (PAH) are widely recognized platforms to hold aryl substituents at proximity. The backbones of PAHs are rigid. As a result, the peripheral substituents are forced into stacking conformations that are impossible with flexible spacers.^6^ The major challenge to constructing such systems is to overcome the steric interactions within narrow spaces. A classical strategy is to convert corresponding halides into the aryl groups via transition-metal catalyzed coupling (Figure 1).^7^ This strategy is the standard protocol to construct 1,8-diaryl naphthalene.^8^ More recently, bay region perylene tetrabromides were used by Würthner to construct chiral rylenes.^9^ Two innovative approaches tackle this difficulty by incorporating groups into the bay region simultaneously as the bay region is formed during annulation reactions. Chalifoux’s group employed alkyne-aryl cyclization^10^ to synthesize peropyrene with four aryl substituents in its bay regions.^11^ Utilizing an alkyne-aryl acetaldehyde benzannulation, Itami and co-workers successfully synthesized a series of 4,5-diaryl phenanthrene with diverse substituents (*ortho*-substituted phenyl, heterocycles, alkoxy) in the bay region.^12^ In this Letter, a mild and versatile annulation strategy toward the bay region substituted 6,7-dihydrodibenzo­[*b,j*]­[4,7]­phenanthroline is presented. This strategy is also adopted to incorporate heterostacks in the bay region. Rotation barriers and rotamer distributions are measured to reveal subtle steric and electronic effects on intramolecular interactions.

**Figure 1:**
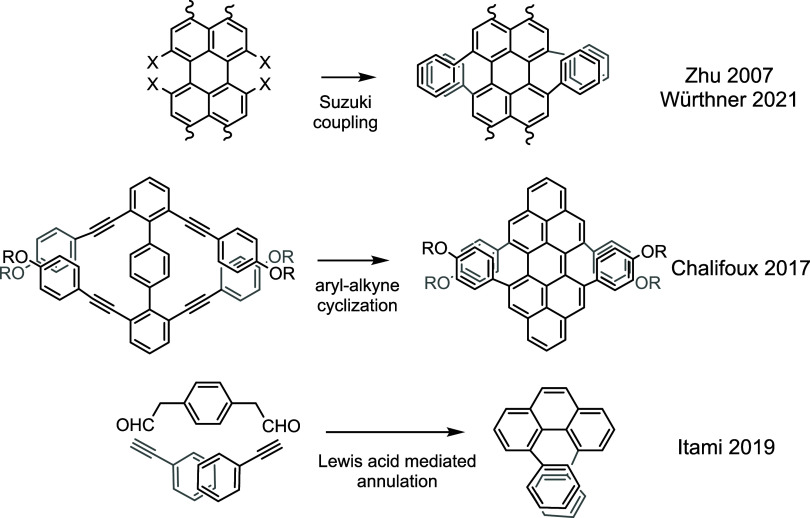
Examples of bay region substituted systems.

## Results and Discussion

We discovered the synthetic route to bay-substituted 6,7-dihydrodibenzo­[*b,j*]­[4,7]­phenanthroline serendipitously. The original intent is to synthesize diazapentacene from 1,4-cyclohexadione and 2-aminobenzophenone via Friedländer annulation (Scheme 1).^13^ Surprisingly, only the more strained angular annulation product **2** was formed with moderate yield. The AB-type NMR signal (3.45 and 3.33 ppm, *J* = 10 Hz) indicates that compound **2** possesses a relatively rigid chiral structure. Two sets of broad signals around 7.0 and 6.5 ppm are consistent with two phenyl groups located in the bay region. Such a crowded arrangement hampers the rotation of phenyl substituents. As a result, protons from one phenyl group reside near the paratropic current of the other one. The preferential production of the more hindered product **2** is explained with the mechanism in Scheme 2. After the first quinoline unit emerges, the second condensation reaction could furnish the linearly or angularly fused product. We propose that the observed selectivity is governed by the superior stability of enamine intermediate A. Since A is more stable due to extensive conjugation (and probably the push–pull effect), the product distribution is dominated by the kinetic pathway through this intermediate.

**Scheme 1:**
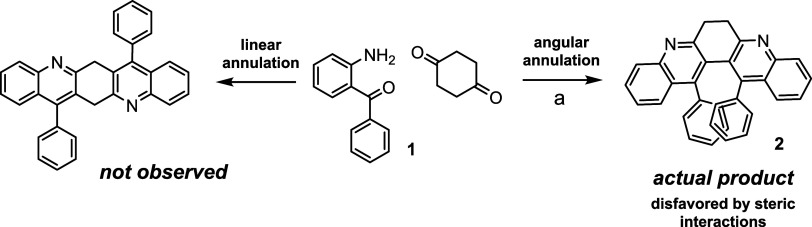
Synthesis of (13,14-Dipheny-6,7-dihydro-dibenzo­[*b,j*]­[4,7]­phenanthroline) via Two-fold Friedländer Condensation^a^

a (a) 5% trifliuoroacetic acid in dichloroethane (by volume), reflux 16 h, 63% after recrystallization.

**Scheme 2:**
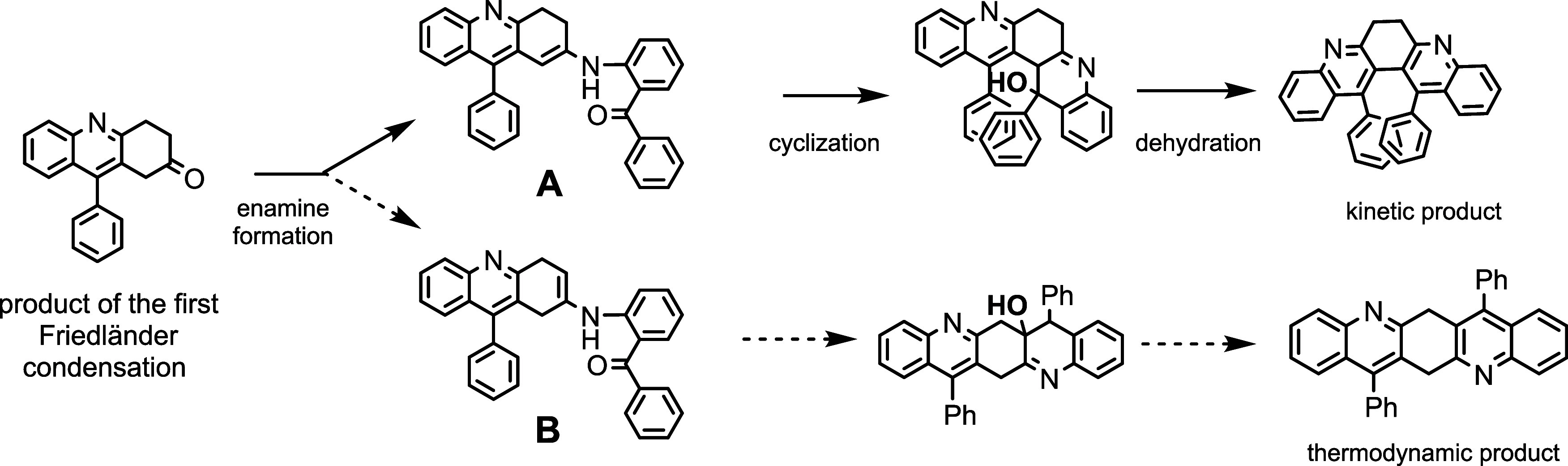
Proposed Mechanism Leading to Crowded Condensation Product **2**

X-ray crystallography established the structure of **2** (13,14-diphenyl-6,7-dihydro-dibenzo­[*b,j*]­[4,7]­phenanthroline) unambiguously (Figure 2, and SI1, pS1–46). The dihedral angle of the bis-quinoline skeleton is 48.09°. The two phenyl substituents adopt a face-to-face stacking conformation with a plane-to-plane distance of 2.96 Å and center-to-center displacement of 2.87 Å. Therefore, the bay region substituted system constitutes a synthetically accessible platform where aryl groups are preorganized to form face-to-face dimers.

**Figure 2:**
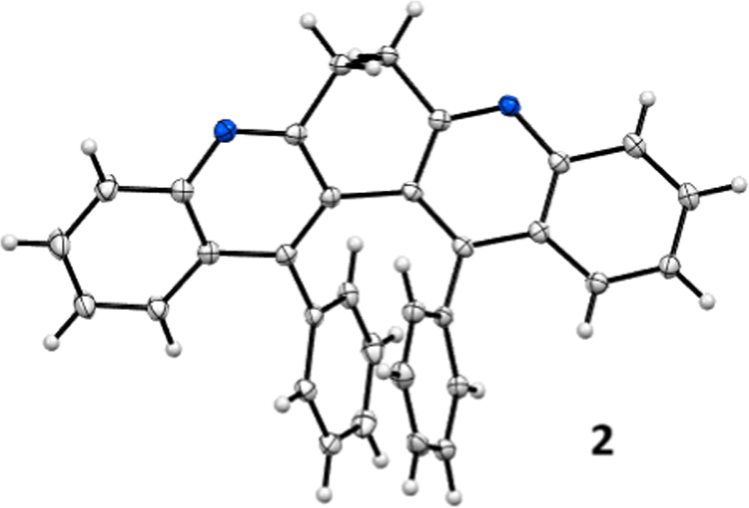
Crystal structure of **2** (with 30% probability of thermal ellipsoid).

To optimize the double annulation, several acids were screened as promoters (Table 1). Trifluoroacetic acid/dichloroethane exhibit the most robust reactivity. Both 5 and 10% solutions deliver good yields of **2** within 8 h. No product degradation is observed with longer reaction times (16 h). Reaction in neat acetic acid gave a much diminished yield (entry 5). The diphenyl phosphate catalyzed reaction also gave an inferior yield. When Lewis acid catalyst Y­(OTf)_3_ was employed (in reflux acetonitrile), **2** was hardly detectable. *p*-Toluene sulfonic acid shows catalytic efficiency comparable to that of trifluoroacetic acid. However, this solid catalyst is hard to apply at high loading (>10 equiv) for less reactive substrates. Since trifluoroacetic acid is inexpensive, easy to operate, and adaptable for further optimization, all subsequent reactions in the present study employ this reagent.

**Table 1:** Yields of Double Friedländer Condensation Reaction with Various Acidic Catalysts^c^,^d^

	catalyst	solvent (reflux)	time (h)	yield
1	trifluoroactic acid (5%)^a^	DCE	8	81%
2	trifluoroactic acid (5%)^a^	DCE	16	81% (63% after crystallization)
3	trifluoroactic acid (10%)^a^	DCE	8	81%
4	trifluoroactic acid (10%)^a^	DCE	16	87%
5	acetic acid (neat)	X	16	30%
6	diphenyl phosphate (25%)^b^	DCE	16	35%
7	Y(OTf)_3_ (25%)^b^	CH_3_CN	16	<10%
8	TsOH (75%)^b^	DCE	16	61%
9	TsOH (3 equiv)^b^	DCE	16	76%

a The percentages of trifluoroacetic acid refer to the volume of DCE.

b The percentages of other catalysts refer to the limiting reagent, 1,4-hexadione.

c DCE = dichloroethane.

d The reactions were conducted with 2.5 equiv of 2-aminobenzophenonoe and 1 equiv of 1,4-hexadione in ∼50 mM DCE solution. The catalyst loadings, reaction temperatures and times are listed in the table.

A series of Boc-protected ortho-amino diaryl ketones were synthesized (Scheme 3) to test the scope and limitation of this 2-fold annulation approach. Boc-aniline 3 is first lithiated via direct ortho lithiation.^14^ The lithiated intermediate then underwent the addition to aryl aldehydes to afford diaryl alcohol. Dess-Martin oxidation then furnishes the building blocks (**4a**-**4l**) for the subsequent transformation.^15^ The deprotection of Boc and 2-fold Friedländer condensation were achieved in one pot to produce a series of bay-substituted 6,7-dihydrodibenzo­[*b,j*]­[4,7]­phenanthroline (**5a**-**5l**) as depicted in Scheme 2. The aryl dimers incorporated in the bay region include *para*-substituted phenyl groups, *ortho*-substituted phenyl groups, heterocycles, and polycyclic aromatic systems. Dimethyl, bis-trifluoromethyl, diisopropyl, and diester derivatives (**5n**-**5q**) are likewise prepared. (Unprotected amino ketone **4m**, **4p**, and **4q** were synthesized via known procedures.)^16^ Amino ketone **4n** and **4o** are commercially available. The yields for the double condensation (20–90%) roughly correlated with steric crowdedness in the bay region. The yields for derivatives with leaner substituents (**5n** and **5o**) are far higher than those with bulkier ones (**5f** and **5j**). For the most hindered substrate, 9-anthryl substrate **4m**, not even the mono condensation product can form. The dismal yield for diester **5q** results from the intramolecular cyclization of starting *ortho*-aminophenyl oxoacetate to form isatin.

**Scheme 3:**
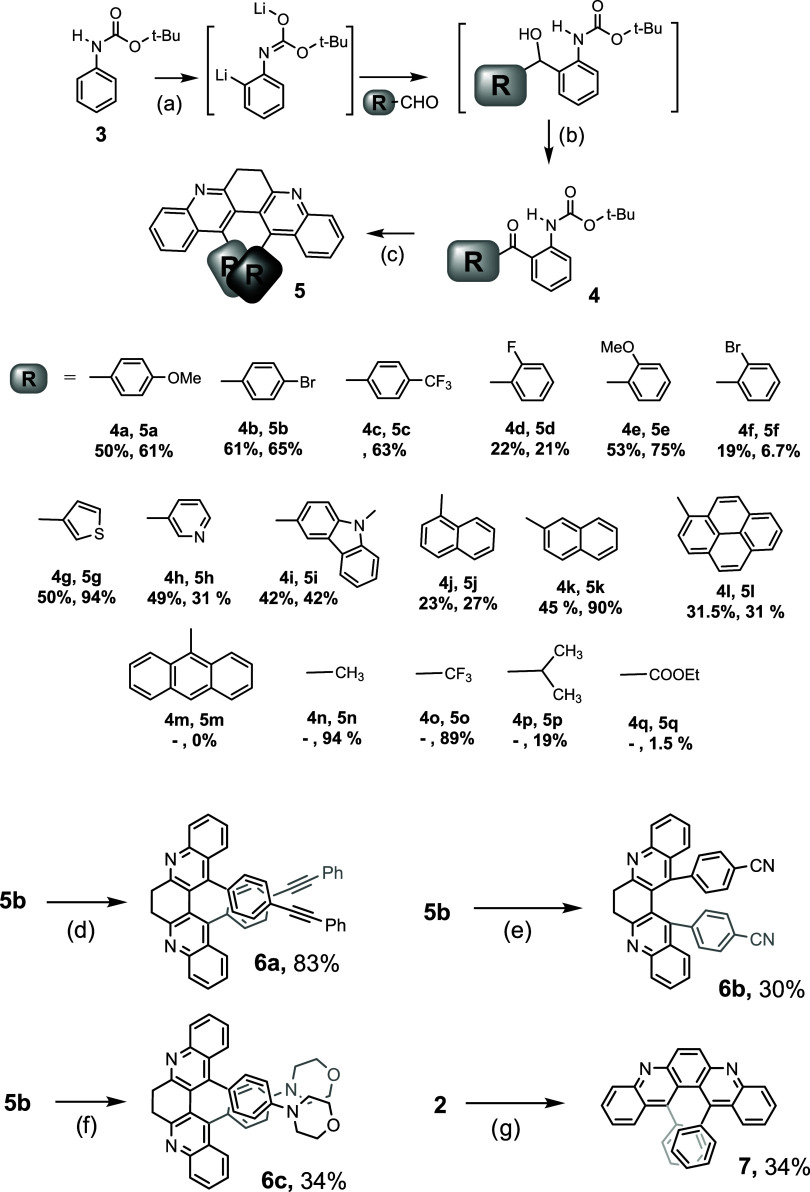
Synthesis of Bay-Substituted 6,7-Dihydro-dibenzo­[*b,j*]­[4,7]­phenanthroline via Double Friedländer Condensation and Derivatization (**5a**–**5q**, **6-7**)­^a^

a (a) 2.2 eq *t-*BuLi, 0 °C, 2 h, then R-CHO, (b) Dess-Martin periondinane, CH_2_Cl_2_, 2.5 h. (**4m**–**4q** are unprotected amino ketone derivatives.) (c) Trifliuoroacetic acid, dichloroethane, reflux 16 h. (d) Pd­(PPh_3_)_4_, CuI, phenylacetylene, NEt_3_, (e) Pd­(PPh_3_)_4_, Zn­(CN)_2_, DMF. (f) (*t-*Bu_3_P)_2_Pd, KOtBu, morpholine, toluene. (g) DDQ, dichlorobenzene, 140 °C.

The aforementioned protocol requires different substrates for each product. Alternatively, dibromide **5b** is utilized as an intermediate to generate structural diversity. The aryl bromide was derivatized through palladium-catalyzed Sonogashira coupling,^17^ Rosenmund von Braun cyanation,^18^ and Buchwald-Hartwig amination^19^ to give bis-alkyne **6a**, dinitrile **6b**, and diamine **6c** (Scheme 3). The ethylene bridge connecting the quinoline units can undergo dehydrogenation (DDQ) to furnish the fully conjugated dibenzo phenanthroline skeleton. The structure of **7** was determined via X-ray crystallography (Figure 3, and SI1, pS1–48). Due to aromatization, the dihedral angle in **7** contracts by 9° (48.09 vs 39.07°) compared to **2**. However, the compressed dihedral angle has little effect on the arrangement of the phenyl dimer. The face-to-face distances remain 2.91 Å, and the center-to-center displacement is 2.89 Å, almost identical to those of **2** (2.96 and 2.87 Å).

**Figure 3:**
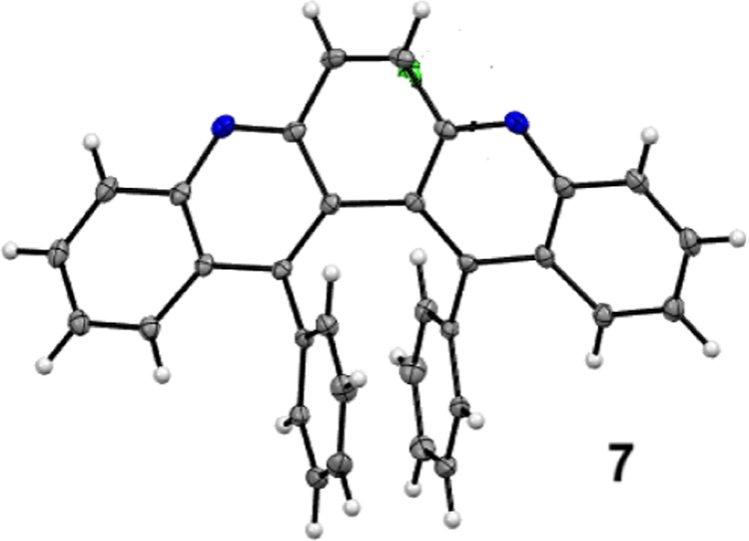
Crystal structure of **7** (with 30% probability of thermal ellipsoid).

Bay-substituted molecules reported to date (Figure 1) carry two identical substituents. This proclivity reflects the limitation of the Suzuki coupling and alkyne-aryl cyclization protocols. In this study, the stepwise nature of double Friedländer condensation provides a solution to this difficulty. According to the mechanism in Scheme 2, the monoannulation phenyl and methyl-substituted intermediate (**8** and **9**) can be intercepted by employing an excess of 1,4-cyclohexadione in the condensation reactions (Scheme 4). The second condensation reaction then installs different groups in the bay region. With the two-stage annulation strategy, heteroaryl stacks like phenyl-thienyl (**10a**), phenyl-pyridyl (**10b**), phenyl-naphthyl (**10c**), and phenyl-pyrenyl (**10d**) were constructed. Aryl-alkyl stacks (**10e**–**10h**) like phenyl-methyl, phenyl-trifluoromethyl, and pyrenyl-methyl were also assembled.

**Scheme 4:**
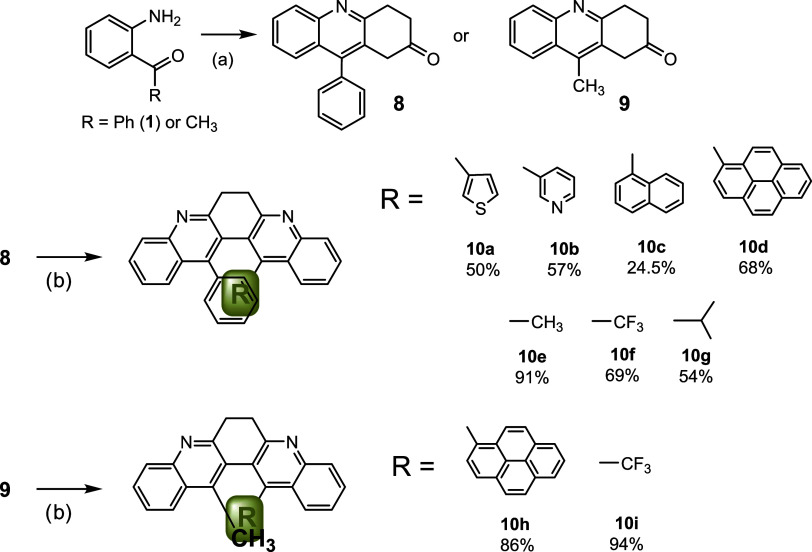
Synthesis of Bay Region Unsymmetrically Substituted 6,7-Dihydrodibenzo­[*b,j*]­[4,7]­phenanthroline (**10a**–**10i**)­^a^

a (a) Trifliuoroacetic acid, dichloroethane, excess cyclohexadione, reflux 16 h. (b) Trifliuoroacetic acid, dichloroethane, reflux 16 h.

There are two modes of hindered rotation in these bay-substituted systems. The rotation of the quinoline-quinoline single bond leads to interconversion between enantiomeric conformations (Figure 4a). The rotation around aryl-quinoline bonds switches the *ortho*-substituent between the “in-bay” and the “out-of-bay” positions (Figure 4b). These hindered rotations were monitored through the evolution of the corresponding NMR signals within a range of temperatures. After their coalescence temperatures are determined, the rotational barriers are estimated via the Eyring equation. Although this approach only gives the free energies of activation at specific temperatures, for such a unimolecular process in a rigid system, these data are still helpful in mapping the energy landscapes.

**Figure 4:**
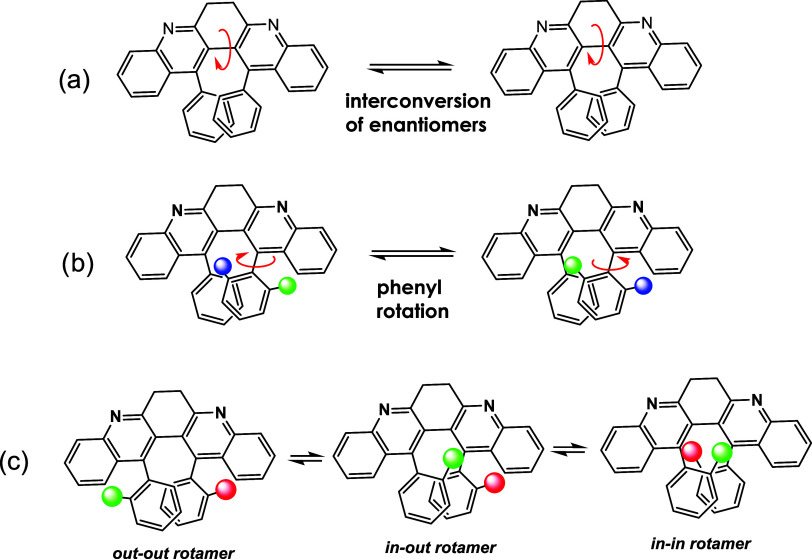
Two rotation modes (a and b) and three rotamers (c) in bay-substituted 6,7-dihydrodibenzo­[*b,j*]­[4,7]­phenanthroline derivatives.

The interconversion of enantiomers is investigated by monitoring the AB-type signals at near 3.5 ppm. The barrier of diphenyl **2** is 18.37 kcal/mol (coalescence temperature = 383 K, SI2, Figure 1). Compounds with sp^2^-carbon-based bay region substituents (dithienyl **5g**, diester **5q**, and phenyl-thienyl **10a**) all possess similar barriers (18.18, 18.57, and 18.50 kcal/mol, SI2, Table 1). Curiously, the interconversion barriers for compounds with smaller substituents (methyl and trifluoromethyl in **5n** and **5o**) are higher (coalescence temperature >393 K, ΔG* > 18.6 kcal/mol).

The phenyl rotation (Figure 4b) is monitored via the coalescence of broad signals around 6.5 ppm (SI2, and Figure 8). The rotation barrier of diphenyl **2** is 13.94 kcal/mol (coalescence temperature of 297 K). For compounds with phenyl-thienyl (**10a**) and phenyl-naphthyl (**10c**) heterostacks, the barriers are similar (13.95 and 14.34 kcal/mol for **10a** and **10c** respectively, SI2, Table S2–2). However, the phenyl rotations in phenyl-methyl, phenyl-trifluoromethyl, and phenyl isopropyl-substituted compounds (14.99 kcal/mol for **10e**,15.62 kcal/mol for **10f**, and 16.78 kcal/mol for **10g**) are noticeably decelerated compared to those in **2**, **10a**, and **10c**.

According to the results, methyl and trifluoromethyl groups (in **5n**, **5o**, **10f**, and **10g**) impose larger steric influences than the aromatic groups (phenyl, thienyl, and naphthyl in **2**, **10a**, and **10c**) in both enantiomer interconversion and phenyl rotation. At first sight, this finding seems counterintuitive because methyl and trifluoromethyl are usually perceived to be less bulky than the aromatic moieties. However, to the phenyl rotor, spherical methyl and trifluoromethyl groups are more effective brakes than the planar aromatic groups due to their larger “effective radius,” which is a set of conceptual parameters extrapolated from measuring the rotation barriers of the 2,2′-biphenyl scaffold.^20^ Compared with the biaryl rotors, the present systems are far more rigid. Nevertheless, due to the structural similarity between the transition states of the biaryl rotor and bay region, the qualitative correlation still holds.

The dynamics of phenyl rotation in stacked diaryl clusters is an indicator of π–π stacking interactions. In systems with stronger stacking interactions, the activation energies of rotation are higher because the ground states are stabilized. On the contrary, weaker stacking interactions lead to lower activation energies. This approach was first employed by Siegel to evaluate the electronic influence on the strength of π–π stacking in 1,8-diaryl naphthalene.^21a^ The seminal study established that π–π stacking interactions are electrostatic in nature as proposed by Hunter and Sanders.^21b^ In the present study, the π–π stacking aryl dimers in bay-substituted dihydrodibenzophenanthroline derivatives exhibit substantial center-to-center displacement. (The interacting aryl groups in1,8-diaryl naphthalene scaffold exhibit no such displacement.) To test the validity of the Hunter-Sanders model in aryl stacks with significant center-to-center displacements, we probed the dynamics of aryl rotation in five *para*-substituted derivatives (**5a**, **5b**, **5c**, **6b**, and **6c**). Compared to diphenyl compound **2** (rotation barrier = 13.94 kcal/mol), compounds carrying electron-withdrawing substituents (Br, CF_3_, and CN in **5b**, **5c**, and **6b**) exhibit impeded rotation (rotation barrier = 14.27, 14.33, 14.29 kcal/mol, respectively, SI2, Table S2–2). On the other hand, electron-donating substituents (OMe and morpholinyl in **5a** and **6c**) facilitate the rotation (rotation barrier = 13.35 and 13.31 kcal/mol, respectively, SI2, Table S2–2). These results are similar to those reported by Siegel,^21a^ indicating that π–π stacking in displaced systems is still dictated by electrostatic interaction. Despite the difference in ground state stacking geometry (no center-to-center displacement versus large center-to-center displacement), the electronic effects on activation energies are comparable (spanning a range of ∼ 1 kcal/mol). The preliminary results suggest that the π–π stacking of the phenyl groups is not necessarily mediated by the overlapping area of the two aryl surfaces. A local, direct interaction model proposed by Wheeler might provide a better interpretation for the observation.^21c,21d^

For compounds carrying *ortho*-substituted phenyl groups (**5d**, **5e**, and **5f**), the distribution of three rotamers (in-in, in-out, and out-out in Figure 4c) can be determined with NMR and used to quantify the stability of the corresponding stacking structures. Due to steric interactions, the out-out rotamers of dimethoxy **5e** (crystal structure, SI1, table S1–2) and dibromide **5f** dominate the population (68% for **5e** and 100% for **5f**). Yet, the in-out rotamer of fluoro-substituted **5d** is more stable than both in-in and out-out counterparts^22^ because electrostatic interaction can overwhelm the smaller steric influence imposed by fluorine atoms. Therefore, the present system also constitutes a new class of “molecular torsional balance” to estimate interactions between proximal groups.^23^

Dinaphthyl and dipyrenyl-substituted **5k** and **5l** are models to evaluate the relationship between the overlapping area of PAH units and their π–π stacking interactions. In these compounds, the confined bay region forces the naphthyl and pyrenyl units to adopt nearly parallel alignments with suitable distances for π–π interactions to take effect. Both compounds possess three rotamers (in-in, in-out, and out-out), and each rotamer has a distinct π–π overlap. Hence, the distribution of rotamers should reflect the magnitude of π–π stacking interactions in each conformation. The NMR signal assignment of rotamers is based on the structures optimized by molecular mechanics (MMFF spartan) and diagnostic upfield aromatic signals (5.5–6.5 ppm). For **5l**, two pyrenyl protons of the in-in rotamer (C_2_) are located above the π-surface of the other pyrenyl unit (Figure 5). The signals corresponding to these protons appear at 5.79 and 6.18 ppm. Similarly, the unsymmetrical in-out rotamer (C_1_) possesses four shielded pyrenyl protons, which appear as four doublet signals at 5.38, 5.69, 5.76, and 6.35 ppm. The one shielded proton in the out-out rotamer is represented by the doublet at 5.92 ppm. The ratio of these three rotamers (in-in: in-out: out-out) is 25:5:1 at 298 K (see SI2, figure S2–20), indicating the in-in and in-out rotamers are more stable than the out-out rotamer by 1.92 and 0.54 kcal/mol.^24^ Yet, chiral HPLC analysis indicates that these rotamers are not interconvertible near room temperature (SI2, figure S2–21). This observation is consistent with another stacked dipyrenyl system, 1,8-dipyrenyl naphthalene, where different rotamers can be crystallized separately.^6^ ^c^ Therefore, the observed distribution could be resulted from the kinetic effect during the Friedländer cyclization.

**Figure 5:**
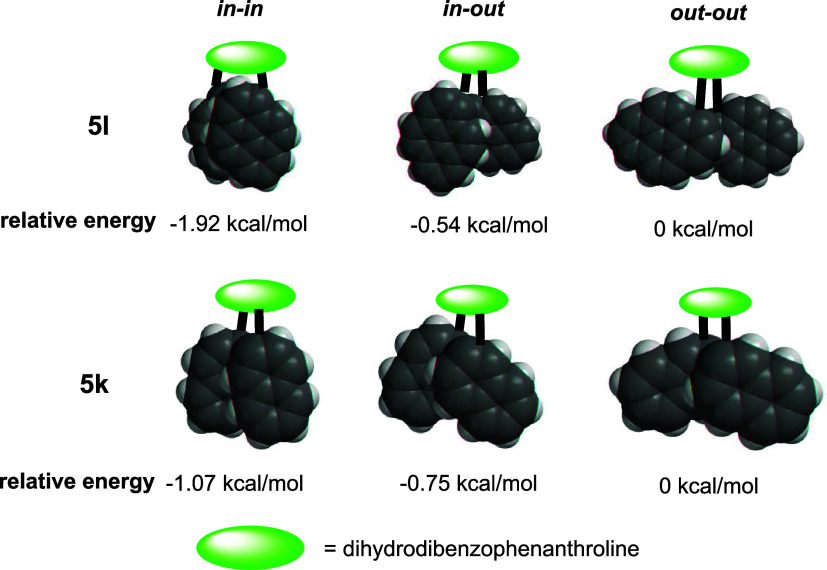
Arrangements of dipyrenyl and dinaphthyl stacks in rotamers of **5k** and **5l** (optimized with MMFF, dihydrodibenzophenanthroline scaffold is omitted for clarity) and relative energies (measured via rotamer distributions) of rotamers.

The rotamer equilibrium in compound **5k**, with 2-naphthyl substituents, was likewise studied. In this more flexible system, the NMR signals of rotamers are resolvable only at 233 K (in-in: in-out: out-out = 10:10:1, see SI2, Figure S2–22). The ratio reveals that in-in and in-out rotamers are more stable than the out-out one by 1.07 and 0.75 kcal/mol. From the relative stability of the rotamers, the π–π interactions in in-in rotamers seem the strongest, and those in out-out rotamers are the weakest. Since the π–π surface overlaps of in-in rotamers are also the largest while those of the out-out rotamers are the smallest (according to the MMFF optimized structures, Figure 5), these results suggest that π–π interactions scale with the overlapping areas of the aromatic stacks.

## Conclusions

In summary, we established bay-substituted 6,7-dihydrodibenzo­[*b,j*]­[4,7]­phenanthroline as a readily accessible and versatile scaffold to investigate subtle interactions between neighboring functional groups. The double Friedländer route is adaptable to incorporate many types of functional groups in the crowded bay region (**5a**-**5q**). Unsymmetrically substituted derivatives (**10a**-**10i**) are accessible via a two-stage Friedländer annulation protocol. This scaffold is rigid enough to hold functional groups at proximity, yet it is also flexible so that the dynamics of crucial bond rotations can be monitored. The results reveal that enantiomer interconversion and phenyl rotation barriers in the current system are governed by the effective radius of bay region substituents, not their overall volumes. Electronic effects on stacking interactions were probed. The results indicate that the Hunter-Sanders model remains valid in displaced systems. The rotamer distributions of naphthyl- and pyrenyl-substituted systems (**5k** and **5l**) unveil the correlation between π–π interactions and overlapping areas of interacting PAH units. More than a tool to measure noncovalent interactions, this structural motif can also serve as a platform to organize such interactions. Potential applications in foldamers and molecular machines shall be exploited.

## Supplementary Material





## Data Availability

The data underlying this study are available in the published article and its Supporting Information.

## Notes

General procedures for synthesis, spectroscopic characterizations, and crystal structure data (PDF)
^1^H and ^13^C NMR spectrum for new compounds and variable temperature NMR experiments (PDF)

## §

Institute of Chemistry, Academia Sinica. Academia Rd. second Sec. No. 127, Taipei 115201, Taiwan, Republic of China
