# Supplementary material for: Investigating Bay-Substituted 6,7-Dihydrodibenzo[b,j][4,7]phenanthroline, a Class of Tunable Hindered Rotors: Synthesis and Molecular Dynamics
Source: J Org Chem. 2025 Jun 3;90(23):7547–54. doi: 10.1021/acs.joc.4c01476 (PMC12172051; doi:10.1021/acs.joc.4c01476)
Supplement: Supplementary file 1 [file jo4c01476_si_001.pdf]

Title: Investigating bay-substituted 6,7-dihydrodibenzo[b,j][4,7]phenanthroline, a class of tunable hindered rotors: Synthesis and molecular dynamics

Authors: Yen-Cheng Lu[b], Jhih-Syong Jhang[a], Chih-Hsiu Lin[a] *

a. Institute of Chemistry, Academia Sinica, Taipei, Taiwan, Republic of China, 115024

b. Department of Chemistry, National Central University Taoyuan, Taiwan, Republic of China

Email of corresponding author: chemopera@gate.sinica.edu.tw

Supporting information 1: General procedures for synthesis, spectroscopic characterizations, and crystal structure data

Table of Contents

Synthesis and characterization of **2**…………………………..…..**pS1-2-pS1-4**

Synthesis and characterization of **4a-4q**……………………..**pS1-4-pS1-14**

Synthesis and characterization of **5a-5q**………………..…..**pS1-14-pS1-29**

Synthesis and characterization of **6a, 6b, 6c, and 7**……..…..**pS1-pS1-34**

Synthesis and characterization of **8** and **9**……………..….....**pS1-35-pS1-37**

Synthesis and characterization of **10a-10i**…………..……..**pS1-38-pS1-45**

Crystal structure data of **2, 5e,** and **7** (table S1-1-S1-3).…**pS1-46-pS1-48**

Materials and general procedures: All starting compounds and reagents were purchased from commercial sources and used without further purification. All reaction anhydrous solvents were purchased from commercial sources and used without further purification. All reactions were performed under 1 atmosphere of dry nitrogen or argon in oven-dried reaction vessels and well-stirred with magnetic stirring devices. Heated reactions were all conducted with oil baths. All reactions were monitored by analytical thin layer chromatography (TLC, 250 EM silica gel 60 F_{254} plates, visualized with a 254 nm UV lamp) or ^{1}H NMR. Flash column chromatography was performed with silica gel 60 (1.11567.9025, 0.040-0.063 mm) as the stationary phase. All reported ratios of eluent solvents mixtures are based on volume. ^{1}H and ^{13}C NMR spectrum for characterization are all recorded in d_1-chloroform solutions. Inseparable grease impurities were observed in some of the compounds. The variable temperature NMR experiments to measure racemization barriers are taken in d_8-toluene. The high-resolution mass spectra were obtained with double focusing magnetic sector as the analyzer. X-ray crystallography were conducted on a Bruker D8-Venture instrument.

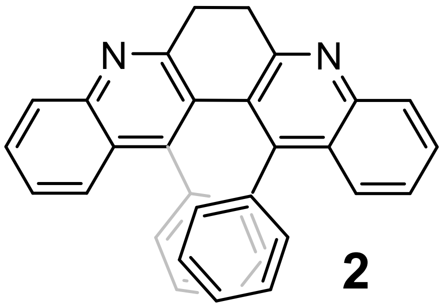

2

A dichloroethane solution (7.5 mL) of 2-aminobenzophenon (197 mg, 1 mmol, 1 eq) and 1,4-hexadione (38 mg, 0.35 mmol, 0.35 eq) was placed in a pressurized tube. To the mixture was added TFA (0.4 mL) and the container was sealed with a Teflon screw-cap. The reaction was refluxed for 20 hrs before diluted with CH_2Cl_2 (30 mL). The solution was washed with saturated $NaHCO_3$ solution (2x30 mL). The organic portion was dried over $MgSO_4$ before concentrated in vacuo. The crude product was then purified with flash chromatography (ethyl acetate: CH_2Cl_2 = 1: 1). **2** was obtained as a dark solid after recrystallization (99 mg, 63 %). The same condition was applied to larger scale reactions (up to 500 mg) with similar yield. The single crystal was grown from a saturated solution of chloroform by slow evaporation.

^{1}H NMR (500 MHz, $CDCl_3$): δ 8.08 (d, J = 8.5 Hz, 2H), 7.65-7.70 (m, 4H), 7.31 (t, J = 8 Hz, 2H), 7.20 (t, J = 7.5 Hz, 2H), 7.16-6.95 (br, 4H), 6.73-6.20 (br, 4H), 3.45 (d, J = 10 Hz, 2H), 3.33 (d, J = 10. Hz, 2H); ^{13}C {^{1}H} NMR (125 MHz, $CDCl_3$): δ 162.4, 147.1, 146.3, 135.4, 131.3 (br), 129.4, 128.7, 128.2, 127.5, 126.0, 125.9, 125.7, 125.0, 35.0. HRMS m/z (ESI): $[M+H]^+$ Calcd for $C_{48}H_{31}N_2$ 435.1855; Found 435.1848.

General procedure **A**: In a two-necked round-bottom flask was placed an ether solution of **3** (500 mg, 2.5 mmol in 10 mL, 0.25 mM) under nitrogen. To the reaction vessel was slowly added *t*-BuLi (1.9 M, 3.4 mL, 2.5 eq.) at -78 ^{o}C. The suspension disappears after the addition of the first equivalent. After the completion of addition, the resulting lightly yellow solution was warmed to -20 ^{o}C and kept at that temperature for 2.5 hrs. Ether solutions of various

aldehydes (1.2 mL in 12 mL diethyl ether, ~2 eq.-2.5 eq., 0.4 mM) were then added. After 2.5 hs at room temperature, the reaction was quenched with a saturated NH_4Cl solution. After the solvent was removed in vacuo, the residue was extracted with ethyl acetate. The combined organic portion was dried over $MgSO_4$ before concentrated. The crude alcohol was dissolved in CH_2Cl_2 (15 mL). To this solution was added Dess-Martin periodinane (5 mmol, 5 mL of 0.1 M solution, 2 eq.) at 0 ^{o}C. The reaction was then stirred at room temperature for 2.5 hrs before extracted with saturated $NaHCO_3$ solution (3x 30mL). The organic portion was dried over $MgSO_4$ and concentrated to furnish the crude ketone product. Pure product was obtained after flash chromatography.

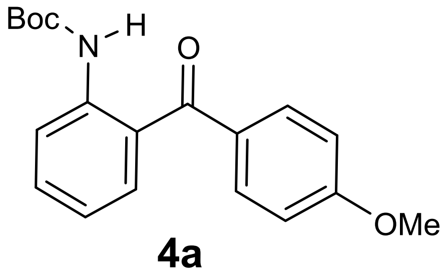

4a

4a was synthesized according to general procedure A as a white powder (221 mg, 50%) after flash chromatography (CH_2Cl_2: hexane = 1: 1).

^{1}H NMR (500 MHz, $CDCl_3$): δ 9.72 (s, 1H), 8.36 (d, *J* = 8Hz, 1H), 7.75-7.72 (m, 2H), 7.53-7.50 (m, 2H), 7.00 (td, *J* = 8 Hz, *J* =1 Hz, 1H), 6.98-6.95 (m, 2H), 3.89 (s, 3H), 1.51 (1.51, 9H); ^{13}C {^{1}H} NMR (125 MHz, $CDCl_3$): δ 197.6, 163.3, 153.1, 140.7, 133.4, 132.8, 132.6, 131.1, 123.8, 120.7, 120.1, 113.6, 80.5, 55.5, 28.3. HRMS m/z (FAB): $[M]^+$ Calcd for $C_{19}H_{21}NO_4$ 321.1471; Found 327.1473.

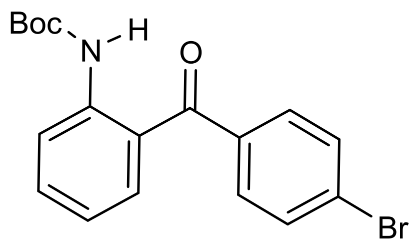

4b was synthesized according to general procedure A as a white powder (210 mg, 61%) after flash chromatography. (CH_2Cl_2: hexane = 1: 1)

^1H NMR (500 MHz, $CDCl_3$): δ 9.96 (s, 1H), 8.41 (dd, *J* = 8 Hz, *J* = 1 Hz, 1H), 7.62-7.52 (m, 5H), 7.46 (d, *J* = 8 Hz, 1H), 6.99 (td, *J* = 8 Hz, *J* = 1 Hz, 1H), 1.52 (s, 9H); ^{13}C {^1H} NMR (125 MHz, $CDCl_3$): δ 198.0, 152.9, 141.4, 137.5, 134.3, 133.2, 131.5, 131.3, 127.3, 122.4, 120.7, 120.0, 80.7, 28.2. HRMS m/z (FAB): $[M]^+$ Calcd for $C_{18}H_{18}NO_3Br$ 375.0470; Found 375.0475.

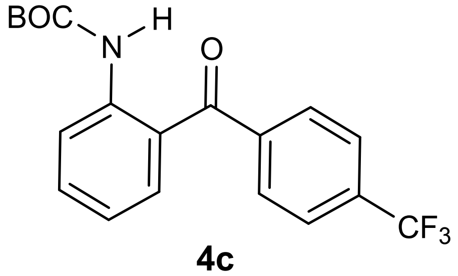

4c

4c was synthesized according to general procedure A as a white powder (378 mg, 40% yield after flash chromatography (CH_2Cl_2: hexane = 1: 1).

^1H NMR (500 MHz, $CDCl_3$): δ 10.12 (s, 1H), 8.45 (dd, *J* = 8.5 Hz, 1 Hz, 1H), 7.79-7.34 (m, 4H), 7.67 (td, *J* = 8 Hz, 1 Hz, 1H), 7.45 (dd, *J* = 8 Hz, 1.5 Hz, 1H), 7.00 (td, *J* = 7.5 Hz, 1 Hz, 1H), 1.52 (s, 9H); ^{13}C {^1H} NMR (125 MHz, $CDCl_3$): δ 198.2, 152.9, 142.1, 141.9, 134.9, 133.6, 133.5 (q, $J_{F\text{-}C}$ = 33.75 Hz),

129.9, 125.3, 125.3, 123.6 (q, $J_{F\text{-}C}$ = 271.25 Hz), 121.9, 120.8, 120.0, 80.9, 28.3; ^{19}F NMR (470 MHz, $CDCl_3$): d -63.01. HRMS m/z (EI): $[M]^+$ Calcd for $C_{19}H_{18}F_3NO_3$ 365.1239; Found 365.1236.

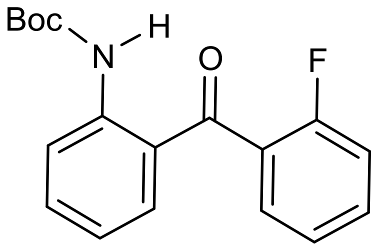

4d

4d was synthesized from general procedure A as a white powder (75 mg, 22 %) after flash chromatography (CH_2Cl_2: hexane = 1:1).

^{1}H NMR (500 MHz, $CDCl_3$): δ 10.64 (s, 1H), 8.50 (dd, *J* = 8.5 Hz, 0.5 Hz, 1H), 7.56-7.48 (m, 2H), 7.47-7.42 (m, 2H), 7.25 (td, *J* = 7.5 Hz, 1 Hz, 1H), 7.15 (t, *J* = 8.5 Hz, 1H), 6.95 (td, *J* = 8 Hz, 1 Hz, 1H), 1.54 (s, 9H); ^{13}C {^{1}H} NMR (125 MHz, $CDCl_3$): δ 196.5, 159.4 (d, $J_{F\text{-}C}$ = 251.25 Hz), 153.0, 142.2, 135.3, 134.11, 132.7 (d, $J_{F\text{-}C}$ = 7.5 Hz), 130.1 (d, $J_{F\text{-}C}$ = 2.5 Hz), 127.78 (d, $J_{F\text{-}C}$ = 15 Hz), 124.2 (d, $J_{F\text{-}C}$ = 3.75 Hz), 121.8, 120.8, 119.1, 116.2 (d, $J_{F\text{-}C}$ = 21.25 Hz), 80.7, 28.2. HRMS m/z (EI): $[M]^+$ Calcd for $C_{18}H_{18}FNO_3$ 315.1271; Found 315.1274.

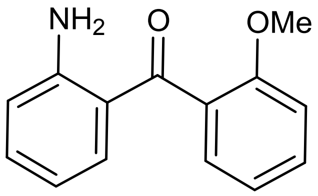

4e

The Boc protected crude **4e** was synthesized according to general procedure A. The mixture was treated with TFA (neat, 30 mins) to remove the Boc group. The amine was obtained as a yellow oil (155 mg, 53 %) after chromatography (CH_2Cl_2: hexane = 4:1).

^{1}H NMR (500 MHz, $CDCl_3$): δ 7.42 (td, *J* = 7.5 Hz, 1.5 Hz, 1H), 7.28-7.24 (m, 3H), 7.02 (td, *J* = 7 Hz, 0.5 Hz, 1H), 6.98 (d, J = 8.5 Hz, 1H), 6.68 (d, J = 8.5 Hz), 6.52 (td, *J* = 8 Hz, 1 Hz, 1H), 6.40-6.27 (br, 2H), 3.76 (s, 3H). The spectrum data match the values in the literature.

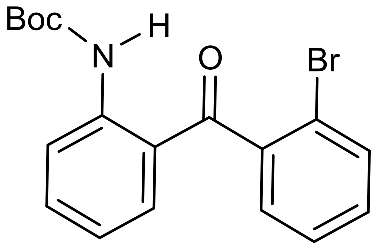

4f

4f was synthesized from general procedure A as a white powder (50 mg, 19 %) after flash chromatography (CH_2Cl_2: hexane = 1:1).

^{1}H NMR (500 MHz, $CDCl_3$): δ10.83 (s, 1H), 8.55 (dd, *J* = 8.5 Hz, 1 Hz, 1H), 7.55 (d, *J* = 8 Hz, 1H), 7.54 (td, *J* = 10 Hz, 1.5 Hz, 1H), 7.43-7.39 (m, 1H), 7.37-7.33 (m, 1H), 7.31-7.27 (m, 2H), 6.91 (td, *J* = 7.5 Hz, 0.5 Hz, 1H), 1.55 (s, 9H); ^{13}C {^{1}H} NMR (125 MHz, $CDCl_3$): δ 199.3, 153.0, 143.0, 141.1, 135.8, 134.7, 133.1,

131.0, 128.6, 127.2, 120.8, 120.5, 119.3, 119.0, 80.8, 28.3. HRMS m/z (FAB): $[M]^+$ Calcd for $C_{18}H_{18}NO_3Br$ 375.0470; Found 375.0479.

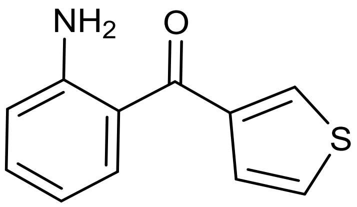

deprotected 4g

The Boc protected crude **4g** was synthesized according to general procedure A. The crude mixture was treated with TFA (neat, 30 min) to provide the amine as a brown liquid (220 mg, 50 %) after flash chromatography (CH_2Cl_2: hexane = 6:4).

^{1}H NMR (500 MHz, $CDCl_3$): δ 7.79 (dd, J = 3 Hz, 1 Hz, 1H), 7.66 (dd, J = 8 Hz, 1.5 Hz, 1H), 7.48 (dd, J = 5 Hz, 2 Hz, 1H), 7.36 (dd, J = 5 Hz, 2 Hz, 1H), 7.29 (td, J = 8 Hz, 1.5 Hz, 1H), 6.72 (dd, J = 8 Hz, 1 Hz, 1H), 6.65 (td, J = 8 Hz, 1 Hz, 1H), 5.93-5.69 (br, 2H) ; ^{13}C {^{1}H} NMR (125 MHz, $CDCl_3$): δ 192.1, 150.3, 142.4, 134.0, 133.4, 131.4, 128.6, 125.7, 119.2, 116.9, 115.7. HRMS m/z (EI): $[M]^+$ for $C_{11}H_9NOS$ 203.0405; Found 203.0402.

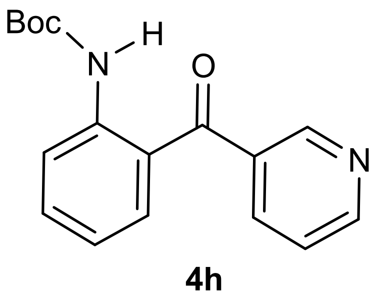

4h

4h was synthesized according to general procedure A as a dark red solid (360mg, 49 %) after flash chromatography (CH_2Cl_2).

^1H NMR (400 MHz, $CDCl_3$): δ 10.09 (s, 1H), 8.91 (d, *J* = 1.6 Hz, 1H), 8.81 (dd, *J* = 5.2 Hz, 1.6 Hz, 1H). 8.45 (d, *J* = 8.8 Hz, 1H), 8.01 (td, *J* = 7.6 Hz, 2 Hz, 1H), 7.58 (td, *J* = 8 Hz, 1.6 Hz, 1H), 7.49 (dd, J = 8 Hz, 1.6 Hz, 1H), 7.44 (dd, *J* = 7.6 Hz, 4.8 Hz, 1H), 7.02 (td, *J* = 7.6 Hz, 1 Hz, 1H), 1.53 (s, 9H); ^{13}C {^1H} NMR (125 MHz, $CDCl_3$): δ 197.5, 153.1, 152.8, 150.7, 141.9, 137.2, 135.1, 134.8, 133.6, 123.4, 121.1, 120.3, 81.0, 28.4. HRMS m/z (FAB): $[M+H]^+$ Calcd for $C_{17}H_{19}N_2O_3$ 299.1396; Found 299.1398.

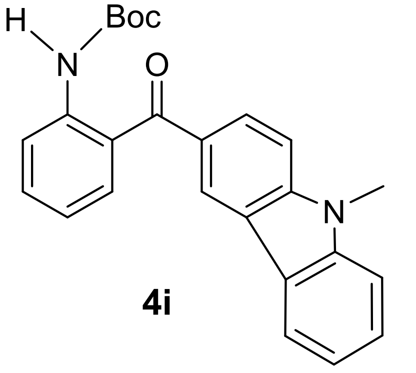

4i

4i was synthesized according to general procedure A as a yellow amorphous solid (217 mg, 42 %) after flash chromatography (CH_2Cl_2: hexane = 1:9).

^1H NMR (500 MHz, $CDCl_3$): δ 9.72 (s, 1H), 8.53 (d, *J* = 1 Hz, 1H), 8.40 (d, *J* = 8 Hz, 1H), 8.10 (dd, *J* = 7.5 Hz, 1 Hz, 1H), 7.95 (dd, *J* = 7.5 Hz, 1.5 Hz, 1H), 7.59 (dd, *J* = 8 Hz, 1.5 Hz, 1H), 7.57-7.52 (m, 2H), 7.45 (t, *J* = 7 Hz, 2H), 7.31 td, *J* = 7.5 Hz, 1 Hz, 1H), 7.05 (td, *J* = 7.5 Hz, 1.5 Hz, 1H), 3.90 (s, 3H), 1.52 (s, 9H); ^{13}C {^1H} NMR (125 MHz, $CDCl_3$): δ 198.5, 153.1, 143.5, 141.7, 140.5,

133.1, 132.9, 129.4, 128.5, 126.6, 124.7, 124.0, 122.9, 122.4, 120.8, 120.6, 120.1, 109.0, 108.0, 80.4, 29.3, 28.3. HRMS m/z (FAB): $[M]^+$ Calcd for $C_{25}H_{24}N_2O_3$ 400.1787; Found 400.1784.

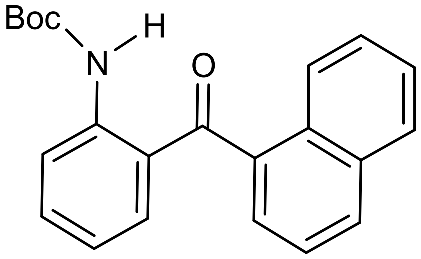

4j

4j was synthesized according to general procedure A as a white powder (105 mg, 23 %) after flash chromatography (CH_2Cl_2: hexane = 1:1).

^{1}H NMR (400 MHz, $CDCl_3$): δ 10.85 (s, 1H), 8.54 (d, J = 8.8 Hz, 1H), 7.99 (dd, J = 7.2 Hz, 1.2 Hz, 1H), 7.93 (td, J = 8.8 Hz, 1.6 Hz, 2H), 7.56-7.47 (m, 5H), 7.38 (dd, J = 8 Hz, 1.6 Hz, 1H), 6.86 (td, J = 7.6 Hz, 0.8 Hz, 1H), 1.56 (s, 9H); ^{13}C {^{1}H} NMR (125 MHz, $CDCl_3$): δ 201.8, 153.3, 142.7, 137.5, 135.4, 135.0, 133.8, 131.1, 130.8, 129.1, 128.6, 127.4, 127.1, 126.7, 125.6, 124.6, 122.9, 120.9, 119.4, 80.9, 28.5. HRMS m/z (EI): $[M]^+$ Calcd for $C_{22}H_{21}NO_3$ 347.1521; Found 347.1513.

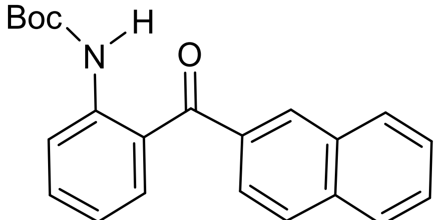

4k

4k was synthesized according to general procedure A as a white powder (235 mg, 45 %) after flash chromatography (CH_2Cl_2: hexane = 1:1).

^{1}H NMR (500 MHz, $CDCl_3$): δ 10.00 (s, 1H), 8.40 (d, *J* = 8.5 Hz, 1H), 8.17 (d, *J* = 1.5 Hz, 1H), 7.95-7.91 (m, 3H), 7.84 (dd, *J* = 8.5 Hz, 1.5 Hz, 1H), 7.62 (td, *J* = 7 Hz, 1.5 Hz, 1H), 7.61-7.55 (m, 3H), 7.02 (td, *J* = 7 Hz, 1.5 Hz, 1H), 1.53 (s, 9H); ^{13}C {^{1}H} NMR (125 MHz, $CDCl_3$): δ 199.2, 153.1, 141.3, 136.0, 135.1, 134.0, 133.5, 132.1, 131.7, 129.4, 128.4, 128.3, 127.8, 126.9, 125.6, 123.2, 120.8, 120.0, 80.6, 28.3. HRMS m/z (EI): $[M]^{+\cdot}$ Calcd for $C_{22}H_{21}NO_3$ 347.1521; Found 347.1523.

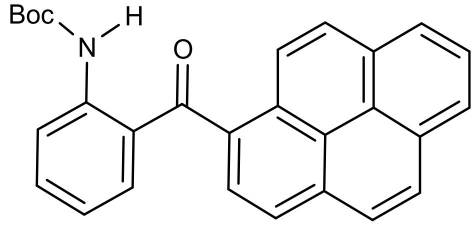

4l

4l was synthesized according to general procedure A as a yellow amorphous solid (272 mg, 31.5 %) after flash chromatography (CH_2Cl_2: hexane = 1:1).

^{1}H NMR (500 MHz, $CDCl_3$): δ 10.96 (s, 1H), 8.61 (d, *J* = 4 Hz, 1H), 8.26-8.16 (m, 5H), 8.12-8.04 (m, 3H), 7.99 (d, *J* = 8 Hz, 1H), 7.56 (td, *J* = 8 Hz, 1 Hz, 1H), 7.35 (dd, *J* = 8 Hz, 1 Hz, 1H), 6.84 (td, *J* = 7.5 Hz, 1 Hz, 1H), 1.60 (s, 9H); ^{13}C NMR {^{1}H} (125 MHz, $CDCl_3$): δ 201.9, 153.2, 142.5, 135.1, 135.1, 134.0, 132.8, 131.2, 130.6, 129.3, 129.1, 128.9, 127.1, 126.4, 126.3, 126.1, 125.9, 124.7, 124.4, 124.4, 123.8, 123.2, 120.8, 119.3, 80.8, 28.3. HRMS m/z (FAB): $[M]^+$ Calcd for $C_{28}H_{23}NO_3$ 421.1678; Found 421.1686.

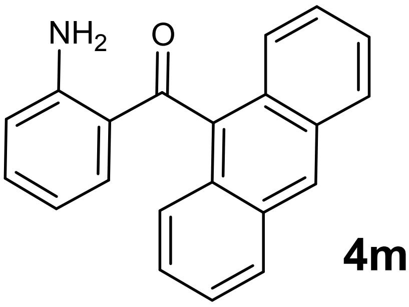

4m

9-Bromoanthracene (0.26 g, 1 mmol), 2,1-benzisoxazole (0.18 g, 1.5 mmol), potassium acetate, 196 mg), and $PdCl(C_3H_5)(DPPB)$ (12.2 mg, 0.02 mmol) were placed in a pressurized tube. The mixture was dissolved in DMA (4 mL) and the solution was deoxygenated. The reaction vessel was sealed with a Teflon screw cap. The reaction was heated at 150 ^oC for 20 hrs. After the solvent was evaporated, the crude product was purified with flash chromatography (ethyl acetate: hexane = 1: 18) to give **4m** as a yellow powder (113 mg, 38 %).

^1H NMR (500 MHz, $CDCl_3$): δ 8.54 (s, 1H), 8.05 (d, J = 8.5 Hz, 2H), 7.78 (td, J = 8.5 Hz, 1 Hz, 2H), 7.47 (td, J = 7.5 Hz, 1 Hz, 2H), 7.41(td, J = 7.5 Hz, 1 Hz, 2H), 7.28-7.25 (m, 1H), 6.93 (dd, J = 8 Hz, 1.5 Hz, 1H), 6.79 (dd, J = 7.5 Hz, 1 Hz, 1H), 6.83-6.65 (br, 2H), 6.32 (td, J = 7.5 Hz, 1.5 Hz, 1H). The spectrum data match with the literature values.

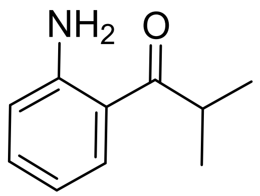

4p

To a THF solution of 2-aminobenzonitrile (200 mg, 1.69 mmol in 10 mL) was added isopropyl magnesium bromide lithium chloride complex (2.6 mmol, 1.3 mL of 2.0 M THF solution, 1.5 eq) at 0 °C. After stirred at room temperature for 2 hrs, the reaction was quenched with 1M HCl (35 mL) and the mixture was stirred at room temperature of an additional 1.5 hrs. The solution was neutralized with saturated $NaHCO_3$ before THF was removed in vacuo. The residue was extracted with CH_2Cl_2 (2 x 50 mL). The combined organic portion was dried over $MgSO_4$ and concentrated. The residue was purified with flash chromatography (CH_2Cl_2: hexane = 1:1) to give **4p** as a lightly brown oil (110 mg, 40 %)

^{1}H NMR (500 MHz, $CDCl_3$): δ 7.76 (d, *J* = 8 Hz, 1H), 7.24 (dd, *J* = 5 Hz, 1.5 Hz, 1H), 6.66-6.63 (m, 2H), 6.31-6.11 (br, 2H), 3.59 (sep, *J* = 7.5 Hz, 1H), 1.20 (d, *J* = 5 Hz, 6H); ^{13}C NMR (125 MHz, $CDCl_3$): δ 207.0, 150.9, 134.0, 130.9, 117.5, 116.8, 115.6, 35.2, 19.6. The spectrum data matches with the literature values.

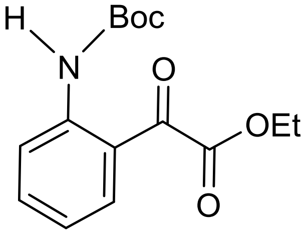

4q

4q was synthesized in two steps from isatin following a known procedure. The spectrum data match with the literature values.

^{1}H NMR (500 MHz, $CDCl_3$): δ 8.52 (d, *J* = 8.5 Hz, 1H), 7.65 (dd, *J* = 8 Hz, 1 Hz, 1H), 7.60 (td, *J* = 8.5 Hz, 1 Hz, 1H), 7.05 (t, J = 7.5 Hz, 1H), 4.45 (qd, *J* = 7 Hz, 1 Hz, 2H), 1.52 (s, 9H), 1.41 (td, J = 7 Hz, 1 Hz, 3H).

General procedure B for the synthesis of bay-substituted [13,14] dibenzo [b,j] [4,7]phenanthroline:

A dichloroethane solution (1 mmol in 7.5 mL) of carbamate (**4a-4q**, 2.8 eq) and 1,4-hexadione (0.35 mmol, 1 eq) was placed in a pressurized tube. To the mixture was added TFA (~5% the volume of dichloroethane, 0.15 mL minimum) and the container was sealed with a Teflon screw-cap. The reaction was refluxed for 20 hrs before diluted with CH_2Cl_2 (30 mL). The solution was washed with saturated $NaHCO_3$ solution (2x30 mL). The organic portion was dried over $MgSO_4$ before concentrated in vacuo. The crude product was then purified with flash chromatography.

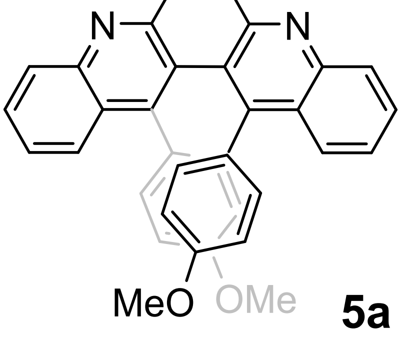

5a

5a was synthesized from **4a** and cyclohexadione according to general procedure B. **5a** was obtained as a white powder (96 mg, 61 %) after flash chromatography (ethyl acetate: CH_2Cl_2 = 1:1).

^{1}H NMR (500 MHz, $CDCl_3$): δ 8.07 (d, *J* = 8.5 Hz, 2H), 7.71 (d, *J* = 8.5 Hz, 2H), 7.65 (td, *J* = 7.5 Hz, 1.5 Hz, 2H), 7.32 (td, *J* = 7.5 Hz, 1.5 Hz, 2H), 6.79-6.63 (br, 4H), 6.46-6.26 (br, 4H), 3.78 (s, 6H), 3.42 (d, *J* = 10 Hz, 2H), 3.31 (d, *J* = 10. Hz, 2H); ^{13}C {^{1}H} NMR (125 MHz, $CDCl_3$): δ 162.4, 159.0, 147.1, 145.8, 132.4, 129.3, 128.7, 128.0, 126.3, 126.1, 125.7, 125.0, 113.7, 55.3, 35.0. HRMS m/z (EI): $[M]^+$ Calcd for $C_{34}H_{26}N_2O_2$ 494.1994; Found 494.1984.

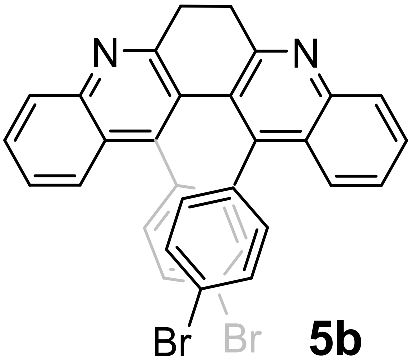

5b

5b was synthesized from **4b** and cyclohexadione according to general procedure B. **4b** was obtained as an white solid (200 mg, 65 %) after flash chromatography (ethyl acetate: CH_2Cl_2 = 1:1).

^{1}H NMR (500 MHz, $CDCl_3$): δ 8.09 (d, *J* = 8.5 Hz, 2H), 7.70 (td, *J* = 7 HZ, *J* = 1.5 HZ, 2H), 7.63 (d, *J* = 8.5 Hz, 2H), 7.36 (td, *J* = 8 Hz, *J* = 1.5 Hz, 2H), 7.46-7.03 (br, 4H), 6.60-6.30 (br, 4H), 3.44 (d, *J* = 10 Hz, 2H), 3.29 (d, *J* = 10. Hz, 2H); ^{13}C {^{1}H} NMR (125 MHz, $CDCl_3$): δ 162.4, 147.2, 144.7, 134.3, 132.8 (br), 131.7, 129.8, 129.0, 126.2, 125.7, 125.4, 124.4, 122.1, 34.9. HRMS m/z (EI): $[M+H]^+$ Calcd for $C_{32}H_{21}Br_2N_2$ 591.0071; Found 591.0079.

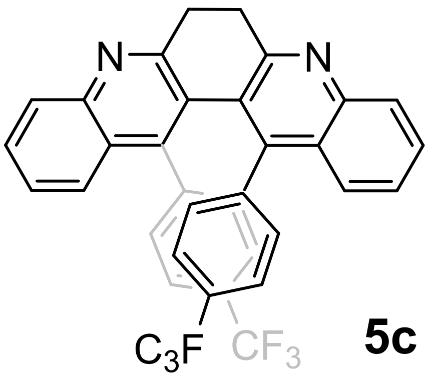

5c

5c was synthesized from **4c** and cyclohexadione according to general procedure B. **5c** was obtained as a greenish solid (67 mg, 63 %) after flash chromatography (ethyl acetate: CH_2Cl_2 = 1:1).

^{1}H NMR (500 MHz, $CDCl_3$): δ 8.11 (dd, *J* = 8 Hz, 1 Hz, 2H), 7.72 (td, J = 7.5 Hz, 1 Hz, 2H), 7.62 (dd, *J* = 8 Hz, 1 Hz, 2H), 7.37 (td, J = 7.5 Hz, 1 Hz, 2H), 7.40-7.29 (br, 4H), 6.94-6.20 (br, 4H), 3.48 (d, *J* = 10 Hz, 2H), 3.32 (d, *J* = 10. Hz, 2H); ^{13}C {^{1}H} NMR (125 MHz, $CDCl_3$): δ 162.4, 147.3, 144.2, 139.2, 131.3

(br), 130.1, 129.8 (q, $J_{F\text{-}C}$ = 32.5 Hz), 129.1, 126.5, 125.6, 125.5, 125.5, 125.2, 124.3, 123.9 (q, $J_{F\text{-}C}$ = 268.75 Hz), 35.0. HRMS m/z (EI): $[M]^+$ Calcd for $C_{34}H_{20}F_6N_2$ 570.1531; Found 570.1521.

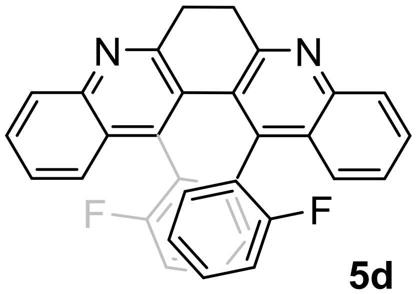

5d

5d was synthesized from **4d** and cyclohexadione according to general procedure B. **5d** was obtained as a white powdery solid (22 mg, 21 %) after flash chromatography with neutral silica (CH_2Cl_2: hexane = 7:3).

^{1}H NMR (500 MHz, $CDCl_3$): δ 8.12-8.07 (m, 2H), 7.68 (td, J = 7 Hz, 1 Hz, 2H), 7.61-7.54 (m, 1H), 7.41-7.24 (m, 5H), 7.05-6.92 (m, 2H), 6.85-6.77 (m, 2H), 6.64-6.60 (m, 1H), 6.61-6.35 (m, 1H), 3.48-3.26 (m, 4H); ^{13}C {^{1}H} NMR (125 MHz, $CDCl_3$): δ 162.7, 162.4, 162.2, 161.8, 160.4 (d, $J_{F\text{-}C}$ = 248.75 Hz), 160.2 (d, $J_{F\text{-}C}$ = 247.5 Hz), 158.7 (d, $J_{F\text{-}C}$ = 248.75 Hz), 158.5 (d, $J_{F\text{-}C}$ = 250 Hz), 147.4, 147.4, 146.64, 146.58, 142.1, 140.4, 139.1, 137.5, 134.0 (d, $J_{F\text{-}C}$ = 2.5 Hz), 133.1 (d, $J_{F\text{-}C}$ = 3.75 Hz), 132.8 (d, $J_{F\text{-}C}$ = 2.5 Hz), 132.6 (d, $J_{F\text{-}C}$ = 3.75 Hz), 130.9 (d, $J_{F\text{-}C}$ = 8.75 Hz), 130.0 (d, $J_{F\text{-}C}$ = 7.5 Hz), 129.9, 129.8, 129.7, 129.5,

129.4, 129.1, 128.9, 128.7, 127.7, 126.8, 126.30 (d, $J_{F\text{-}C}$ = 3.75 Hz), 126.23 (d, $J_{F\text{-}C}$ = 2.5 Hz), 126.15 (d, $J_{F\text{-}C}$ = 5 Hz), 126.04 (d, $J_{F\text{-}C}$ = 2.5 Hz), 125.86 (d, $J_{F\text{-}C}$ = 3.75 Hz), 125.77, 125.56, 125.36, 125.29, 125.13, 124.68 (d, $J_{F\text{-}C}$ = 3.75 Hz), 124.38, 124.25 (d, $J_{F\text{-}C}$ = 2.5 Hz), 124.20, 124.16, 124.15, 123.0 (d, $J_{F\text{-}C}$ = 13.75 Hz), 122.4 (d, $J_{F\text{-}C}$ = 13.75 Hz), 116.85 (d, $J_{F\text{-}C}$ = 21.25 Hz), 116.39 (d, $J_{F\text{-}C}$ = 21.25 Hz), 116.22 (d, $J_{F\text{-}C}$ = 21.25 Hz), 116.14 (d, $J_{F\text{-}C}$ = 21.25 Hz), 35.06, 34.79, 34.67, 34.46. HRMS m/z (EI): $[M]^+$ Calcd for $C_{32}H_{20}F_2N_2$ 470.1595; Found 470.1605.

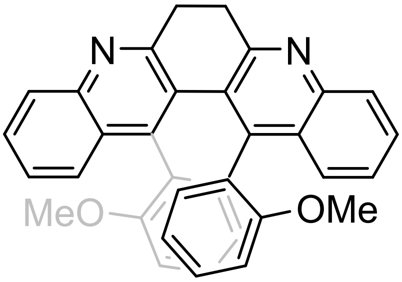

5e

5d was synthesized from **4d** and cyclohexadione according to general procedure B. **5d** was obtained as an white solid (82 mg. 75 %) after flash chromatography with neutral silica (ethyl acetate: CH_2Cl_2 = 7:3). Single crystal suitable for x-ray crystallography was grown by slowly from a solution of dichloromethane by slowly

^{1}H NMR (400 MHz, $CDCl_3$): δ 8.06-8.13 (m, 2H), 7.68-7.53 (m, 3.5 H), 7.31-7.17 (m, 4.5 H), 6.84-6.75 (m, 2 H), 6.60-6.50 (m, 2.3 H), 6.60-6.50 (m, 2.3H), 6.28-6.22 (m, 1.7 H, 3.42-3.24 (m, 4H, overlapping with -**OMe** peak), 3.31 (s, 1H), 3.18 (s, 1H), 3.07 (s, 4H) (Non-integer integrations are due to rotamers.); ^{13}C {^{1}H} NMR (125 MHz, $CDCl_3$): δ 163.09, 162.10, 161.49, 157.4, 155.6, 155.1, 147.35, 147.19, 146.27, 139.3, 134.0, 133.0, 132.2, 129.60, 129.45, 129.11, 129.07, 128.96, 128.82, 128.75, 128.65, 128.60, 128.47, 127.78, 127.53, 127.14, 126.69, 126.29, 125.95, 125.76, 125.53, 125.40, 125.33, 125.08, 124.75, 124.69, 123.98, 123.5, 120.8, 120.2, 119.7, 111.4, 110.93, 110.75, 54.91, 54.69, 54.16, 35.08, 34.79, 34.51. HRMS m/z (EI): $[M]^+$ Calcd for $C_{34}H_{26}N_2O_2$ 494.1994; Found 494.1990.

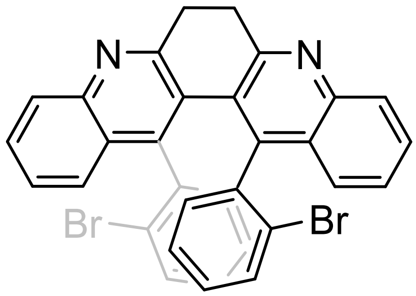

5f

5f was synthesized from **4f** and cyclohexadione according to general procedure B. **4f** was obtained as an white powdery solid (17 mg, 6.7 %) after flash chromatography with neutral silica (CH_2Cl_2: hexane = 7:3).

^{1}H NMR (400 MHz, $CDCl_3$): δ 8.12 (d, *J* = 8.4 Hz, 2H), 7.70 (m, 2H), (dd, *J* = 7.6 Hz, 1.6 Hz, 2H), 7.33-7.30 (m, 4H), 7.16-7.08 (m, 4H), 6.29 (dd, *J* = 7.2 Hz, 2.0 Hz, 2H), 3.48-3.22 (m, 4H); δ ^{13}C {^{1}H} NMR (125 MHz, $CDCl_3$): δ 162.8, 147.2, 142.9, 135.9, 134.8, 133.6, 129.6, 129.1, 128.9, 127.56, 127.29, 126.1, 125.91, 125.80, 123.1, 34.3. HRMS m/z (EI): $[M]^+$ Calcd for $C_{32}H_{20}Br_2N_2$ 589.9993; Found 590.0000.

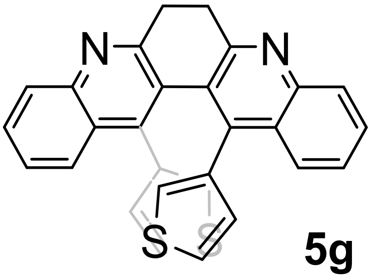

5g

5g was synthesized from **4g** and cyclohexadione according to general procedure B. **5g** was obtained as a dark powdery solid (99 mg, 94 %) after flash chromatography (ethyl acetate: CH_2Cl_2 = 1:1).

^{1}H NMR (500 MHz, $CDCl_3$): δ 8.07 (d, *J* = 8 Hz, 2H), 7.92 (d, *J* = 8 Hz, 2H), 7.67 (td, *J* = 7.5 Hz, *J* = 1 Hz, 2H), 7.37 (dd, *J* = 7.5 Hz, *J* = 1 Hz, 2H), 7.04 (dd, *J* =

2.5 Hz, J = 1.5 Hz, 2H), 7.03 (d, J = 1.5 Hz, 2H), 6.31 (dd, J = 3.5 Hz, J = 1 Hz, 2H), 3.26-3.45 (m, 4H); $^{13}C\{^1H\}$ NMR (125 MHz, $CDCl_3$): δ 162.0, 147.0, 140.7, 135.5, 129.5, 129.4, 128.8, 126.2, 125.97, 125.88, 125.73, 125.13, 124.6, 36.4. HRMS m/z (EI): $[M]^+$ Calcd for $C_{28}H_{18}N_2S_2$ 446.0911: Found 446.0910.

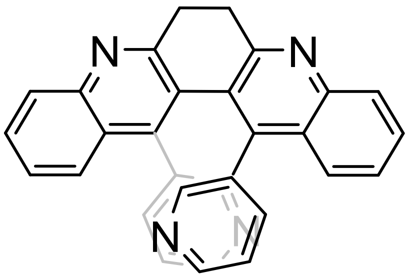

5h

5h was synthesized from **4h** and cyclohexadione according to general procedure B. **5h** was obtained as a dark red amorphous solid (29 mg, 31 %) after flash chromatography (ethyl acetate: CH_2Cl_2 = 9:1).

^1H NMR (500 MHz, $CDCl_3$): δ 8.53 (bs, 2H), 8.11 (d, J = 7.5 Hz, 2H), 7.93-7.48 (br, 4H), 7.72 (t, J = 7.5 Hz, 2H), 7.21-6.65 (br, m, 4H), 3.41 (d, J = 10 Hz, 2H), 3.29 (d, J = 10. Hz, 2H); $^{13}C\{^1H\}$ NMR (125 MHz, $CDCl_3$): δ 162.3, 150.7, 148.8, 147.2, 142.3, 137.8, 131.8, 130.2, 129.2, 126.7, 125.34, 125.0, 124.5, 123.6, 34.7. HRMS m/z (FAB): $[M]^{+\cdot}$ Calcd for $C_{30}H_{21}N_4$ 437.1766; Found 437.1757.

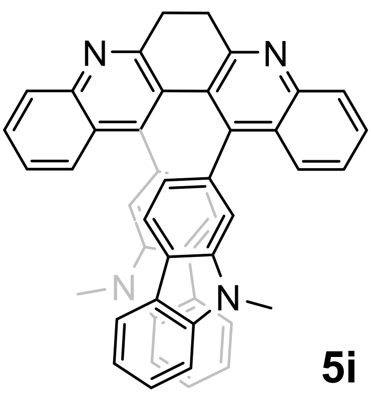

5i

5i was synthesized from **4i** and cyclohexadione according to general procedure B. **5i** was obtained as a yellow amorphous solid (27 mg, 42 %) after flash chromatography (ethyl acetate: CH_2Cl_2 = 9:1)

^{1}H NMR (500 MHz, $CDCl_3$, 258K): δ 8.11-8.04 (m, 2 H), 7.73-7.55 (m, 6 H), 7.54 (m, 4H), 7.30 (t, *J* = 7.5 Hz, 1H), 7.24-7.03 (m, 5H), 6.78-6.67 (m, 2H), 6.59-6.52 (m, 1H), 6.34 (d, *J* = 8.5 Hz, 0.5 H), 5.88 (d, *J* = 8.5 Hz, 0.5 H), 3.85-3.69 (m, 6H), 3.57-3.37 (m, 4H); ^{13}C {^{1}H} NMR (125 MHz, $CDCl_3$): δ 162.60, 162.38, 147.08, 147.04, 146.96, 146.92, 146.74, 141.08, 140.72, 140.49, 139.91, 139.78, 139.73, 129.40, 129.18, 128.43, 128.37, 128.30, 128.17, 127.7, 126.75, 126.69, 126.64, 126.24, 126.00, 125.97, 125.80, 125.60, 125.52, 125.48, 125.39, 125.36, 125.31, 125.26, 125.03, 123.77, 123.29, 122.80, 122.54, 122.39, 122.29, 122.14, 121.97, 121.36, 120.47, 120.09, 118.83, 118.81, 118.72, 108.75, 108.37, 108.02, 107.79, 107.66, 35.02, 35.00, 34.93,

29.25, 29.11, 29.03. HRMS m/z (FAB): $[M+H]^+$ Calcd for $C_{46}H_{33}N_4$ 641.2705; Found 641.2698.

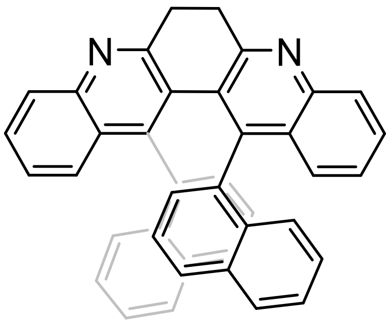

5j

5j was synthesized from **4j** and cyclohexadione according to general procedure B. **5j** was obtained as a white amorphous solid (21 mg, 27 %) after flash chromatography (ethyl acetate: CH_2Cl_2 = 7:3)

^1H NMR (500 MHz, $CDCl_3$): δ 8.11-8.07 (m, 1H), 8.00-7.96 (m, 2H), 7.85-7.63 (m, 3H), 7.57-7.50 (m, 2H), 7.38-7.28 (m, 2H), 7.23-7.01 (m, 6H), 6.94-6.74 (m, 3H), 6.61-6.59 (m, 1H), 6.13-6.05 (m, 1H), 5.73-5.80 (m, 1H) , 3.65-3.30(m, 4H); ^{13}C {^1H} NMR (125 MHz, $CDCl_3$): δ 163.22, 162.75, 162.05, 161.5, 147.49, 147.36, 146.55, 146.45, 145.0, 144.0, 134.83, 134.74, 133.52, 133.44, 133.30, 133.10, 133.00, 132.49, 132.21, 131.49, 131.34, 130.03, 129.68, 129.64, 129.59, 129.48, 129.43, 129.46, 129.30, 129.15, 128.98, 128.66, 128.57, 128.54, 128.45, 128.33, 128.27, 128.09, 127.88, 127.79, 127.76, 127.68,

127.56, 127.23, 127.07, 127.02, 127.00, 126.48, 126.45, 126.35, 126.31, 126.16, 125.98, 125.80, 125.69, 125.66, 125.58, 125.49, 125.41, 125.36, 125.33, 125.23, 125.00, 124.99, 124.85, 123.75, 123.41, 123.24, 35.78, 35.72, 35.16, 35.04; HRMS m/z (EI): $[M]^+$ Calcd for $C_{40}H_{26}N_2$ 534.2096; Found: 534.2100.

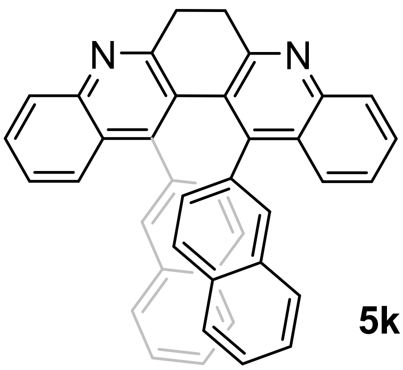

5k

5k was synthesized from **4k** and cyclohexadione according to general procedure B. **5k** was obtained as a white powder (97 mg, 90 %) after flash chromatography (ethyl acetate: CH_2Cl_2 = 7:3)

^1H NMR (500 MHz, $CDCl_3$, 233K): δ 8.12-8.06 (m, 2H), 7.77-7.64 (m, 5H), 7.63-7.38 (m, 6H), 7.33-7.17 (m, 4H), 6.93-6.72 (m, 1H), 6.67-6.64 (m, 2H), 6.42-6.37 (m, 1.5H), 5.99-5.80 (m, 0.5H), 3.51-3.41 (m, 4H) (Non-integer integrations are due to rotamers); ^{13}C {^1H} NMR (125 MHz, $CDCl_3$, 233K): δ 162.43, 162.40, 162.35, 146.89, 146.73, 146.56, 146.27, 146.16, 145.98, 133.26, 132.99,

132.63, 132.37, 132.14, 132.09, 131.94, 131.83, 131.74, 130.76, 129.72, 129.69, 129.66, 129.60, 129.11, 128.83, 128.75, 128.45, 128.32, 128.23, 128.11, 127.92, 127.70, 127.61, 127.55, 127.49, 127.44, 127.39, 127.31, 127.25, 126.96, 126.81, 126.75, 126.54, 126.47, 126.33, 126.25, 125.98, 125.89, 125.81, 125.80, 125.76, 125.73, 125.59, 124.86, 124.81, 124.59, 124.54, 34.83, 34.73, 34.60. HRMS m/z (EI): $[M]^+$ Calcd for $C_{40}H_{26}N_2$ 534.2096; Found: 534.2102.

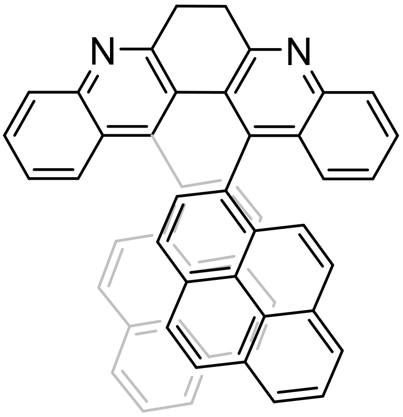

5l

5l was synthesized from **4l** and cyclohexadione according to general procedure B. **5l** was obtained as a yellow amorphous solid (27 mg, 31 %) after flash chromatography (ethyl acetate: CH_2Cl_2 = 7: 3)

^1H NMR (500 MHz, $CDCl_3$): δ 8.32-8.09 (m, 2.6H), 8.18-7.85 (m, 8H), 7.74-7.37 (m, 5.9H), 7.07-6.62 (m, 5.7H), 6.37-5.90 (m, 1.8H), 5.82-5.35 (m, 2H), 3.77-

3.48 (m, 4H) (Non-integer integrations are due to rotamers); ^{13}C {^1H} NMR (125 MHz, $CDCl_3$): δ 163.35, 162.97, 162.27, 161.76, 147.57, 147.36, 146.75, 146.70, 146.53, 145.24, 144.35, 143.52, 131.52, 131.42, 131.27, 131.12, 131.09, 131.03, 130.98, 130.81, 130.69, 130.62, 130.54, 130.50, 130.45, 130.37, 130.16, 130.10, 130.04, 129.61, 129.55, 129.47, 129.43, 129.38, 128.96, 128.72, 128.59, 128.55, 128.15, 127.98, 127.74, 127.64, 127.36, 127.30, 127.21, 127.14, 127.10, 126.93, 126.85, 126.74, 126.56, 126.50, 126.39, 126.08, 125.97, 125.84, 125.69, 125.60, 125.54, 125.42, 125.33, 125.29, 125.11, 124.90, 124.77, 124.64, 124.57, 124.54, 124.42, 123.40, 123.29, 123.15, 123.10, 122.7, 35.74, 35.37, 35.33, 35.18. HRMS m/z (FAB): $[M+H]^+$ Calcd for $C_{52}H_{31}N_2$ 683.2487; Found: 683.2484.

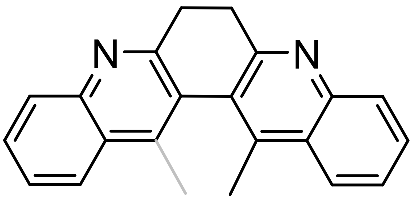

5n

5n was synthesized from **4n** (commercially available) and cyclohexadione according to general procedure B. **5n** was obtained as a white powder (85 mg, 94 %) after flash chromatography (ethyl acetate: CH_2Cl_2 = 7:3)

^{1}H NMR (500 MHz, $CDCl_3$): δ 8.09 (dd, *J* = 5.5 Hz, 1 Hz, 2H), 8.07 (dd, *J* = 5.5 Hz, 1 Hz, 2H), 7.74 (td, *J* = 7.5 Hz, 1.5 Hz, 2H), 7.59 (td, *J* = 7.5 Hz, 1.5 Hz, 2H), 3.35 (d, *J* = 10.5 Hz, 2H), 3.20 (d, *J* = 10.5 Hz, 2H),, 2.56 (s, 6H); ^{13}C {^{1}H} NMR (125 MHz, $CDCl_3$): δ 161.6, 146.1, 141.6, 129.5, 129.0, 127.7, 126.3, 126.1, 124.4, 34.3, 17.4. HRMS m/z (EI): $[M]^+$ Calcd for $C_{22}H_{18}N_2$ 310.1478; Found: 310.1470.

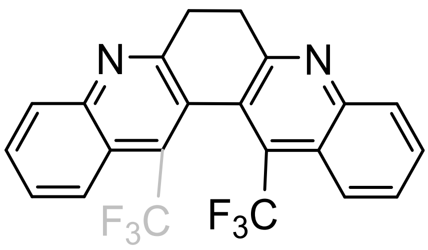

5o

5o was synthesized from **4o** (commercially available) and cyclohexadione according to general procedure B. **5o** was obtained as a white powder (56 mg, 89 %) after flash chromatography (ethyl acetate: CH_2Cl_2 = 1:1)

^{1}H NMR (500 MHz, $CDCl_3$): δ 8.20 (d, *J* = 8.5 Hz, 2H), 8.16 (dd, *J* = 8.5Hz, 0.5Hz, 2H), 7.84 (td, *J* = 7.5Hz, 1Hz, 2H), 7.68 (td, *J* = 8Hz, 1Hz, 2H), 3.44 (d, *J* = 10.5 Hz, 2H), 3.20 (d, *J* = 11 Hz, 2H); ^{13}C {^{1}H} NMR (125 MHz, $CDCl_3$): δ 160.9, 147.5, 133.9 (d, $J_{CF3\text{-}C}$ = 32Hz), 130.9, 129.2, 127.7, 125.1, 123.5 (q,

$J_{F\text{-}C}$ = 278.75Hz), 123.2, 34.0; ^{19}F NMR (470 MHz, $CDCl_3$): δ -54.1. HRMS m/z (EI): $[M]^+$ Calcd for $C_{22}H_{18}F_6N_2$ 418.0905; Found: 418.0903.

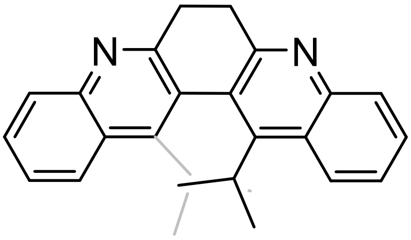

5p

5p was synthesized from **4p** and cyclohexadione according to general procedure B. **5p** was obtained as a white fine powder (14 mg, 19 %) after flash chromatography (ethyl acetate: CH_2Cl_2 = 3:7)

^{1}H NMR (400 MHz, $CDCl_3$): δ 8.42 (d, *J* = 8.4 Hz, 2H), 8.08 (d, *J* = 8.4 Hz, 2H), 7.70 (td, *J* = 7.2 Hz, *J* = 1.2 Hz, 2H), 7.50 (td, *J* = 7.2 Hz, *J* = 1.2 Hz, 2H), 3.57 (sep, *J* = 7.2 Hz, 2H), 3.26-2.94 (m, 4H), 1.87 (d, *J* = 7.2 Hz, 6H), 1.14 (d, *J* = 7.2 Hz, 6H); ^{13}C {^{1}H} NMR (125 MHz, $CDCl_3$): δ 162.3, 150.8, 147.5, 129.8, 129.0, 126.1, 125.9, 125.5, 125.0, 35.5, 30.7, 24.0, 23.3. HRMS m/z (EI): $[M]^+$ Calcd for $C_{26}H_{26}N_2$ 366.2096; Found 366.2089.

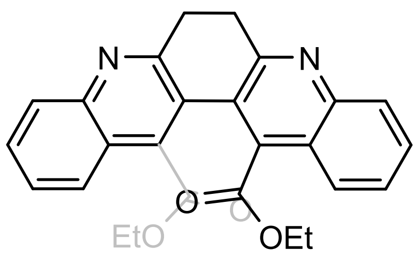

5q

5q was synthesized from **4q** and cyclohexadione according to general procedure B yet with higher concentration (1.5 mL/mmol). **5q** was obtained as a yellow powder (2.0 mg, 2 %) after flash chromatography (ethyl acetate: CH_2Cl_2 = 1:1)

^{1}H NMR (500 MHz, $CDCl_3$): δ 8.37 (d, *J* = 8.5 Hz, 2H), 8.12 (d, *J* = 8.5 Hz, 2H), 7.84 (t, *J* = 8Hz, 2H), 7.60 (t, *J* = 8Hz, 2H), 4.29-4.14 (m, 4H), 3.45 (d, *J* = 10 Hz, 2H), 3.29 (d, *J* = 10.5 Hz, 2H), 0.88 (t, *J* = 7.5 Hz, 6H); ^{13}C {^{1}H} NMR (125 MHz, $CDCl_3$): δ 166.0, 160.8, 147.2, 136.8, 130.4, 129.0, 127.4, 125.9, 125.5, 124.1, 62.3, 33.6, 13.5. HRMS m/z (EI): $[M]^+$ Calcd for $C_{26}H_{22}N_2O_4$ 426.1580; Found 426.1589.

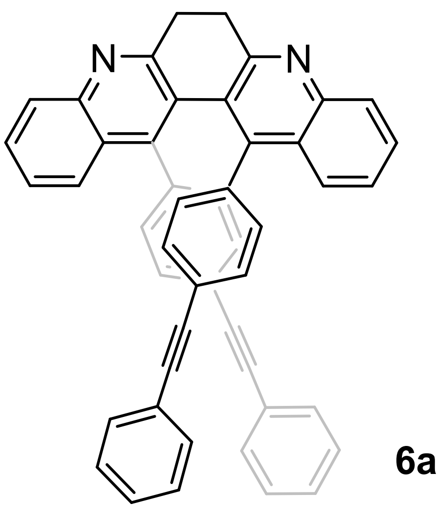

6a

5b (100 mg, 017 mmol, 1 eq)**,** phenyl acetylene (69 mg, 0.68 mmol, 4 eq), copper iodide (5 mg), and $Pd(PPh_3)_4$ (20 mg, 0.017 mmol, 0.1 eq) was placed in a pressurized tube. Et_3N (1 mL) was added under nitrogen and the solution was deoxygenated for 10 min. The tube was sealed with a Teflon screw-cap and heated at 70 ^oC for 20 hrs. After cooled to room temperature, the mixture was filtered through a pad of Celite. The filtrate was partition between water and CH_2Cl_2. The organic fraction was dried over $MgSO_4$ and concentrated in vacuo. The crude product wa purified with flash chromatography (ethyl acetate: CH_2Cl_2 = 3:7) to furnish **6a** as a yellow amorphous solid (170 mg, 83 %).

^{1}H NMR (500 MHz, $CDCl_3$): δ 8.10 (d, *J* = 8 Hz, 2H), 7.71-7.68 (m, 4H), 7.55-7.53 (m, 4H), 7.36-7.34 (m, 4H), 7.48-7.08 (br, 8H), 6.78-6.35 (br, 4H),

3.47 (d, J = 10 Hz, 2H), 3.33 (d, J = 10. Hz, 2H); ^{13}C {^{1}H} NMR (125 MHz, $CDCl_3$): δ 162.3, 147.2, 145.4, 135.3, 131.6, 131.5, 131.3 (br), 129.7, 128.9, 128.47, 128.36, 126.08, 125.81, 125.53, 124.7, 122.88, 122.59, 90.6, 88.0, 34.9. HRMS m/z (EI): $[M+H]^+$ Calcd for $C_{48}H_{31}N_2$ 635.2487; Found 635.2482.

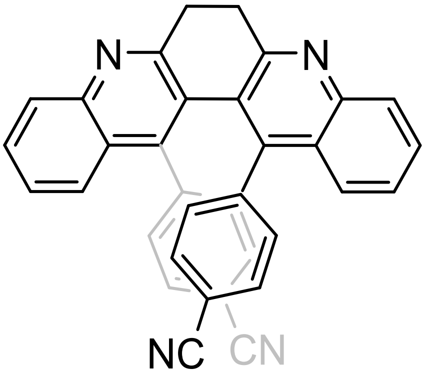

6b

Dibromide **5b** (100 mg, 0.17 mmol), zinc cyanide (79 mg, 0.68 mmol, 4 eq.) were suspended in DMF (5 mL). To this solution was added $Pd(PPh_3)_4$ (39 mg, 0.34 mmol, 0.2 eq) before the mixture was deoxyganted for 10 min. The reaction was heated at 100 °C for 12 hrs. The solution was filtered through a pad of Celite before the solvent was removed in vacuo. The residue was partitioned in CH_2Cl_2 and water and the mixture was extracted with CH_2Cl_2 (3X20 mL). The combined organic portion was dried over $MgSO_4$ before

concentrated. The crude product was purified with chromatography (ethyl acetate: hexane = 1:1) to furnish **6b** as a red powder (25 mg, 30 %).

^{1}H NMR (400 MHz, $CDCl_3$): δ 8.12 (dd, *J* = 8 Hz, 0.8 Hz, 2H), 7.75 (td, *J* = 7.2 Hz, 1.2 Hz, 2H), 7.52 (dd, *J* = 8 Hz, 0.8 Hz, 2H),7.51-7.35 (br, 4H, overlap with other peaks), 7.41 (td, *J* = 7.2 Hz, 1.2 Hz, 2H), 6.87-6.45 (br, 4H), 3.54-3.27 (m, 4H); ^{13}C {^{1}H} NMR (100 MHz, $CDCl_3$): δ 162.3, 147.3, 143.8, 140.2, 132.2, 132.0, 130.4, 129.3, 126.8, 125.0, 124.72, 124.20, 118.1, 111.7, 34.8. HRMS m/z (EI): $[M]^+$ Calcd for $C_{34}H_{20}N_4$ 484.1688; Found 484.1690.

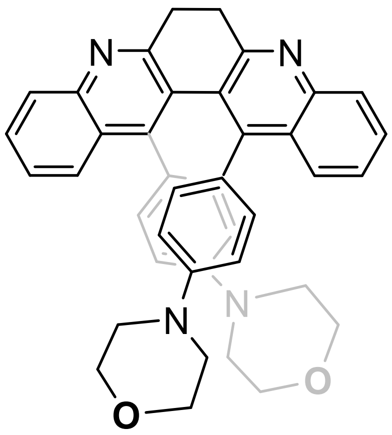

6c

A toluene (2 mL) suspension of KO*t*-Bu (49 mg, 0.44 mmol, 4 eq) was placed in a pressurized tube. A toluene (3 mL) solution of **5b** (70 mg, 0.11 mmol, 1 eq), morpholine (40 mg, 0.44 mmol, 4 eq), and Pd(P*t*-Bu$_3$)$_2$ (6 mg, 0.03 mmol, 0.07 eq) was added to the tube under nitrogen. The reaction mixture

deoxygenated for 10 min before the tube was sealed with a Teflon screw-cap. The tube was heated at 120 °C for 16 hrs. The reaction was quenched with saturated NH_4Cl solution and the mixture was extracted with ethyl acetate (3 X 20 mL). The combined organic fraction was dried over $MgSO_4$ and concentrated in vacuo. The residue was purified with flash chromatography (ethyl acetate: CH_2Cl_2 = 1: 4) to furnish **6c** as a white amorphous solid (20 mg, 34 %).

^1H NMR (400 MHz, $CDCl_3$): δ 8.07 (d, J = 8 Hz, 2H), 7.74 (d, J = 8 Hz, 2 H), 7.65 (td, J = 8 Hz, 1.2 Hz, 2H), 7.30 (td, J = 8 Hz, 1.2 Hz), 3.86 (m, 8 H), 3.43 (d, J = 10.4 Hz, 2H), 3.34 (d, J = 10.4 Hz, 2H), 3.14 (m, 8H); ^{13}C {^1H} NMR (100 MHz, $CDCl_3$): δ 162.5, 150.4, 147.1, 146.1, 132.3, 132.1, 131.4, 129.3, 128.7, 127.1, 126.4, 126.3, 125.6, 125.0, 115.0, 66.8, 48.9, 34.9; HRMS m/z (EI): $[M+H]^+$ Calcd for $C_{40}H_{37}N_4O_2$ 605.2917; Found 605.2910.

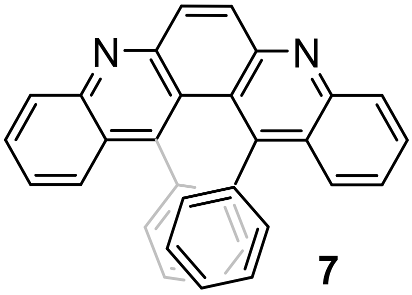

7

In a sealed pressurized tube was placed a dichlorobenzene (10 mL) solution of **2** (70 mg, 0.16 mmol), DDQ (73 mg, 0.32 mmol). The reaction was refluxed for 3 hrs. After cooled to room temperature, the precipitation was filtered and the solvent was removed in vacuo. The crude product was purified with flash chromatography (ethyl acetate: CH_2Cl_2 = 1:1) to furnish **7** as a dark solid (23 mg, 34%). Single crystal of **7** suitable for x-ray crystallography was grown from chloroform solution by slow evaporation.

^{1}H NMR (500 MHz, $CDCl_3$): δ 8.24 (dd, *J* = 8.5 Hz, 0.5 Hz, 2H), 7.93 (s, 2H), 7.83 (dd, *J* = 8.5 Hz, 0.5 Hz, 2H), 7.44 (td, *J* = 7.5Hz, 1 Hz, 2H), 7.39 (td, *J* = 7.5Hz, 1 Hz, 2H), 7.25 (t, J = 7 Hz, 2H), 7.21-6.97 (br, 4H), 6.52 (dd, *J* = 8.5 Hz, 1 Hz, 4H) (a signal from grease was observed near 1.3 ppm); ^{13}C {^{1}H} NMR (125 MHz, $CDCl_3$): δ 151.1, 147.8, 147.5, 136.6, 133.5, 131.5, 129.9, 129.2, 128.6, 127.6, 126.6, 126.0, 124.2, 122.0. HRMS m/z (FAB): $[M+H]^+$ Calcd for $C_{32}H_{21}N_2$ 433.1705; Found 433.1706.

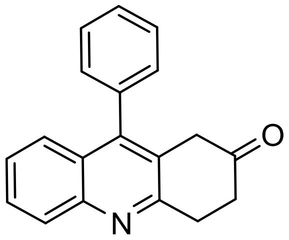

8

2-aminobenzophenone (**1**, 1 eq.) and cyclohexadione (3 eq.) was dissolved in dichloroethane (8 mL/mmol). To the solution was added the trifluoroacetic acid (2.5 % volume of the dichloroethane) and the mixture was placed in a pressurized tube. The sealed vessel (Teflon screw-cap) was refluxed for 16 hrs before diluted with ethyl acetate (30 mL). The solution was washed with brine (2x 30 mL) and the organic fraction was dried over $MgSO_4$ and concentrated in vacuo. The crude product was purified with flash chromatography (silica gel neutralized with 1% Et_3N, CH_2Cl_2: hexane = 7:3) to give **8** as a lightly brown solid (485 mg, 76 %).

^{1}H NMR (500 MHz, $CDCl_3$): δ 8.09 (d, *J* = 8 Hz, 1H), 7.69 (td, *J* = 7.5 Hz, 1 Hz, 1H),7.55-7.39 (m, 5H), 7.24-7.22 (m, 2H), 3.55 (s, 2H), 3.50 (t, *J* = 7 Hz , 2H), 2.75 (t, *J* = 7 Hz, 2H); ^{13}C {^{1}H} NMR (125 MHz, $CDCl_3$): δ 208.8, 157.8, 146.7, 146.7, 135.4, 129.3, 129.2, 128.8, 128.8, 128.4, 126.8, 126.3, 126.2, 123.6, 42.8, 37.6, 32.4. HRMS m/z (FAB): $[M+H]^+$ Calcd for $C_{19}H_{16}NO$ 274.1232; Found 274.1235.

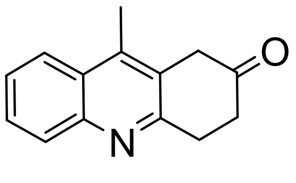

9

2-aminoacetophenone (500 mg, 3.7 mmol, 1 eq) and cyclohexadione (854 mg, 11 mmol, 3 eq) was dissolved in dichloroethane/TFA mixed solvent (28 mL/ 0.75 mL). The solution was placed in a sealed tube (Teflon screw-cap) and the reaction was refluxed for 16 hrs. After cooled to room temperature, the mixture was diluted with CH_2Cl_2 (30 mL) and washed with saturated $NaHCO_3$ solution (2x30 mL). The organic fraction was dried over $MgSO_4$ and concentrated in vacuo. The crude product was purified with flash chromatography (silica gel neutralized with 1% Et_3N, CH_2Cl_2: hexane = 7:3) to furnish **9** as a lightly brown solid. (430 mg, 55 %).

^{1}H NMR (500 MHz, $CDCl_3$): δ 8.02 (t, *J* = 7.5 Hz, 2H), 7.69 (td, *J* = 7.5 Hz, 1 Hz, 1H), 7.55 (td, *J* = 7.5 Hz, 1 Hz, 1H), 3.81 (s, 2H), 3.42 (t, *J* = 7 Hz, 2H), 2.76 (t, *J* = 7 Hz, 2H), 2.60 (s, 3H); ^{13}C {^{1}H} NMR (125 MHz, $CDCl_3$): δ 208.9, 157.1, 146.3, 141.3, 129.3, 129.0, 127.0, 126.1, 124.1, 123.6, 41.9, 37.8, 32.50, 13.7. HRMS m/z (FAB): $[M+H]^+$ Calcd for $C_{14}H_{14}NO$ 212.1075; Found 212.1076.

Synthesis of bay-region unsymmetrically substituted dibenzo[b,f][4,7]phenanthroline (general procedure C):

Benzophenone *ortho* carbamate (**4g, 4h, 4k, 4l**, …., 1 mmol, 1 eq.) and **8** or **9** (1.1 mmol, 1.1 eq) was dissolved in dichloroethane (7.5 mL). The solution was

placed in a pressurized tube before TFA (5% the volume of dichloroethane, 0.15 mL minimum) was added. The condensation reaction was refluxed in the sealed tube for 12 hrs before diluted with CH_2Cl_2 (30 mL). The mixture was washed with saturated $NaHCO_3$ solution. The organic fraction was then dried over $MgSO_4$ and concentrated in vacuo. The crude product was purified with flash chromatography to give pure compound.

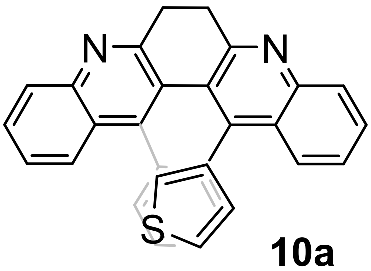

10a

10a was synthesized from **4g** and **8** according to general procedure C. **10a** was obtained as a brown solid (48 mg, 50 %) after flash chromatography (ethyl acetate: CH_2Cl_2 = 1:1)

^{1}H NMR (400 MHz, $CDCl_3$): δ 8.11-8.06 (m, 2H), 7.86 (d, J= 8.8 Hz, 1H), 7.75 (d, J= 8.4 Hz, 1H), 7.70-7.64 (m, 2H), 7.36-7.32 (m, 2H), 7.23-6.88 (m + br, 4H), 6.60 (d, J= 8.4 Hz, 2H), 6.48 (d, J= 2.8 Hz, 1H), 6.22 (d, J= 4.8 Hz. 1H), 3.48-3.26 (m, 4H); ^{13}C {^{1}H} NMR (125 MHz, $CDCl_3$): δ 162.26, 162.20, 147.08, 146.98, 146.38, 140.8, 135.65, 135.25, 131.17, 130.71, 129.98, 129.54, 129.52,

128.72, 128.21, 127.4, 126.93, 126.14, 125.92, 125.85, 125.66, 125.22, 124.94, 124.66, 34.76, 34.75. HRMS m/z (EI): $[M]^+$ Calcd for $C_{30}H_{20}N_2S$ 440.1347; Found 440.1342.

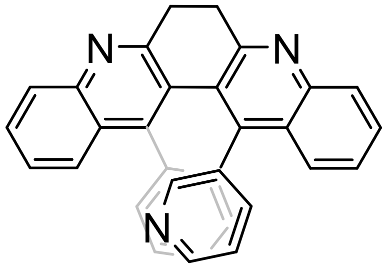

10b

10b was synthesized from **4h** and **8** according to general procedure C. **10b** was obtained as a red amorphous solid (42 mg, 57 %) after flash chromatography (ethyl acetate: CH_2Cl_2 = 4:1)

^{1}H NMR (500 MHz, $CDCl_3$): δ 8.45 (d, *J* = 4 Hz, 1H), 8.11-8.07 (m, 2H), 7.70-7.55 (m, 5H), 7.37-7.32 (m, 3H), 7.13-6.95 (br, 4H), 6.68-6.27 (br, 2H), 3.52-3.26 (m, 4H); ^{13}C {^{1}H} NMR (125 MHz, $CDCl_3$): δ 162.39, 162.25, 148.31, 147.23, 147.00, 146.10, 137.8, 135.4, 131.8, 131.7, 130.9, 129.80, 129.00, 128.99, 128.85, 128.65, 127.98, 126.35, 126.04, 125.92, 125.91, 125.75, 125.74, 125.40, 125.30, 125.09, 124.2, 123.2, 34.87, 34.80. HRMS m/z (EI): $[M]^+$ Calcd for $C_{31}H_{21}N_3$ 435.1735; Found 435.1731.

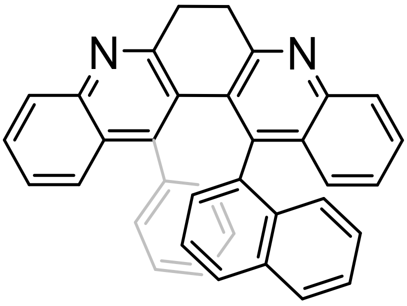

10c

10c was synthesized from **4k** and **8** according to general procedure C. **10c** was obtained as a white amorphous solid (19 mg, 24.5 %) after flash chromatography (ethyl acetate: CH_2Cl_2 = 1:1)

^1H NMR (400 MHz, $CDCl_3$): δ 8.15-8.10 (m 1H), 8.07-7.90 (m, 1H), 7.93-7.90 (m,1H), 7.81-7.67 (m, 2H), 7.64-7.47 (m, 3H), 7.40-7.12 (m, 6H), 7.01-6.87 (m, 2H), 6.86-6.69 (m, 2H), 6.45 (br, 1H), 6.44-6.17 (m, 1H), 6.16-5.58 (br, m, 1H), 3.55-3.35 (m, 4H); ^{13}C {^1H} NMR (100 MHz, $CDCl_3$): δ 163.0, 162.5, 162.0, 147.55, 146.99, 146.88, 146.62, 145.52, 143.9, 135.86, 135.14, 134.31, 133.42, 133.35, 133.03, 132.1, 130.77, 130.28, 129.65, 129.47, 129.39, 129.22, 129.19, 128.89, 128.66, 128.61, 128.57, 128.25, 128.07, 127.93, 127.80, 127.56, 127.33, 127.14, 126.98, 126.84, 126.54, 126.30, 126.23, 126.13, 126.00, 125.93, 125.77, 125.67, 125.62, 125.55, 125.50, 125.20, 125.13, 124.73, 123.47, 35.55, 35.08, 34.89. HRMS m/z (EI): $[M]^+$ Calcd for $C_{36}H_{24}N_2$ 484.1939; Found 484.1937.

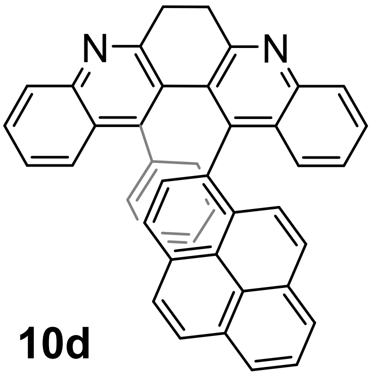

10d

10d was synthesized from **4l** and **8** according to general procedure C. **10d** was obtained as a yellow amorphous solid (91 mg, 68 %) after flash chromatography (ethyl acetate: CH_2Cl_2 = 4:1)

^{1}H NMR (500 MHz, $CDCl_3$): δ 8.25 (d, J= 7 Hz, 0.5H), 8.18-8.16 (m, 1H), 8.14-8.08 (m, 2H), 8.07-8.05 (m, 1.5H), 7.99-7.92 (m, 1H), 7.89-7.81 (m, 2H), 7.70-7.59 (m, 2H), 7,45-7.27 (m, 4H), 7.24-7.12 (m, 3H), 7.10-7.01 (m, 1.5H), 7.00-6.74 (two broad signals, 1H), 6.72-6.60 (m, 1.5H), 5.31-5.13 (two broad signals, 1H), 3.61-3.38 (m, 4H); ^{13}C {^{1}H} NMR (125 MHz, $CDCl_3$): δ 162.61, 162.12, 162.05, 147.57, 147.11, 146.95, 146.90, 146.81, 145.87, 145.62, 144.2, 135.49, 135.46, 131.55, 131.27, 131.24, 131.07, 130.94, 130.63, 130.52, 130.49, 130.28, 130.11, 129.63, 129.58, 129.58, 129.42, 129.24, 129.15, 128.78, 128.66, 128.60, 128.47, 128.28, 128.02, 127.99, 127.97, 127.83, 127.66, 127.48, 127.37, 127.32, 127.24, 127.06, 126.90, 126.74, 126.61,

126.09, 125.95, 125.90, 125.85, 125.64, 125.53, 125.50, 125.32, 125.24, 125.16, 124.80, 124.68, 124.64, 124.59, 124.54, 124.24, 123.0, 35.49, 35.22, 35.16, 35.04. HRMS m/z (EI), $[M+H]^+$ Calcd for $C_{42}H_{27}N_2$ 559.2174; Found 559.2176.

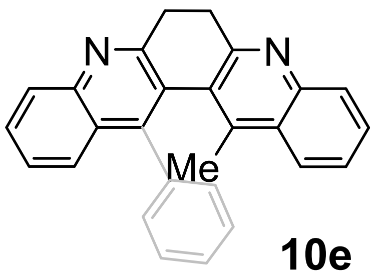

10e

10e was synthesized from **4n** and **8** according to general procedure C. **10e** was obtained as a white powder (81 mg, 91 %) after flash chromatography (ethyl acetate: CH_2Cl_2 = 1:4)

^{1}H NMR (500 MHz, $CDCl_3$): δ 8.15 (d, *J* = 8.5 Hz, 2H), 8.02 (t, *J* = 9 Hz, 2H), 7.84-7.64 (br, 1H), 7.75 (t, *J* = 7.5 Hz, 1H), 7.66-7.60 (m, 3H), 7.47 (t, *J* = 7.5 Hz, 1H), 7.40 (t, *J* = 7.5 Hz, 1H), 7.29 (t, *J* = 7.5 Hz, 1H), 7.07-6.87 (br, 1H), 6.63-6.41 (br, 1H), 3.45-3.22 (m, 4H), 1.90 (s, 3H); ^{13}C {^{1}H} NMR (125 MHz, $CDCl_3$): δ 162.65, 161.61, 147.3, 146.8, 146.2, 142.3, 137.0, 132.2, 131.7, 129.84, 129.47, 129.16, 128.83, 128.22, 127.97, 127.56, 126.34, 126.23,

126.04, 125.87, 124.44, 34.91, 34.59, 18.41. HRMS m/z (EI): $[M]^+$ Calcd for $C_{27}H_{20}N_2$ 372.1626; Found 372.1630.

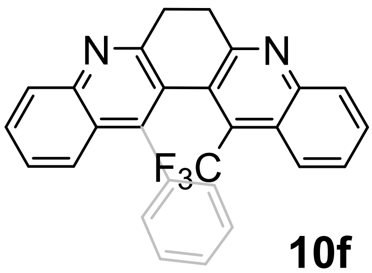

10f

10f was synthesized from **4o** and **8** according to general procedure C. **10f** was obtained as a white amorphous solid (79 mg, 69 %) after flash chromatography (ethyl acetate: CH_2Cl_2 = 1:1)

^{1}H NMR (500 MHz, $CDCl_3$): δ 8.14 (d, J = 8 Hz, 1H), 8.08 (d, J = 8.5 Hz, 1H), 7.94 (d, J = 8.5 Hz, 1H), 7.83-7.69 (m, 4H), 7.55-7.45 (m, 3H), 7.30-7.27 (m, 1H), 6.97-6.84 (br, 1H), 6.39 (d, J = 6.5 Hz, 1H), 3.46-3.20 (m, 4H); ^{13}C {^{1}H} NMR (125 MHz, $CDCl_3$): δ 161.9, 160.9, 149.4, 147.64, 146.71, 134.37, 134.09, 131.6 (q, $J_{F\text{-}C}$ = 31.25 Hz), 130.24, 130.05, 128.96, 128.85, 128.44, 127.66, 127.11, 126.80, 126.25, 125.99, 125.57, 124.64, 123.91, 123.13 (q, $J_{F\text{-}C}$ = 277.5 Hz), 123.01, 122.02, 119.8, 34.49, 34.16; ^{19}F NMR (470 MHz, $CDCl_3$) : δ -56.24. HRMS m/z (EI): $[M]^+$ Calcd for $C_{27}H_{17}F_3N_2$ 426.1344; Found 426.1352.

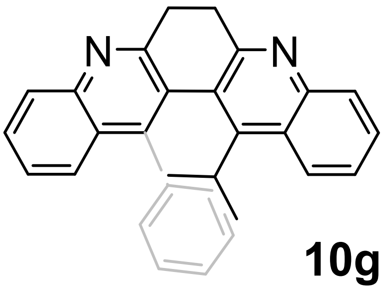

10g

10g was synthesized from **4p** and **8** according to general procedure C. **10g** was obtained as a lightly yellow solid (52 mg, 54 %) after flash chromatography (ethyl acetate: CH_2Cl_2 = 1:1).

^{1}H NMR (500 MHz, $CDCl_3$): δ 8.13 (dd, *J* = 8.5 Hz, 1 Hz, 1H), 8.11 (dd, *J* = 9 Hz, 1 Hz, 1H), 8.05 (dd, *J* = 8.5 Hz, 1 Hz, 1H), 8.00 (dd, *J* = 8.5 Hz, 1 Hz, 1H), 7.94-7.49 (br, 2H), 7.73 (td, *J* = 7.5 Hz, 1 Hz, 1H), 7.63 (td, *J* = 7.5 Hz, 1 Hz, 1H), 7.47 (td, *J* = 8 Hz, 1.5 Hz, 1H), 7.35 (td, *J* = 8.5 Hz, 1.5 Hz, 1H), 7.28 (t, *J* = 7.5 Hz, 1H), 7.21-6.45 (br, 2H), 3.46 (sep, *J* = 7 Hz, 1 H), 3.37-3.13 (m, 4H), 1.02 (d, *J* = 7.5 Hz, 3H), 0.70 (d, *J* = 7.5 Hz, 3H); ^{13}C {^{1}H} NMR (125 MHz, $CDCl_3$): δ 162.8, 161.8, 151.3, 147.16, 146.98, 146.00, 135.4, 133.2 (br), 131.7 (br), 129.58, 129.34, 129.03, 128.81, 128.69 (br), 128.56 (br), 128.32, 126.38, 126.05, 125.94, 125.92, 125.86, 125.44, 125.07, 124.8, 35.17, 35.07, 30.8, 24.0, 21.5. HRMS m/z (EI): $[M]^+$ Calcd for $C_{29}H_{24}N_2$ 400.1939; Found: 400.1930.

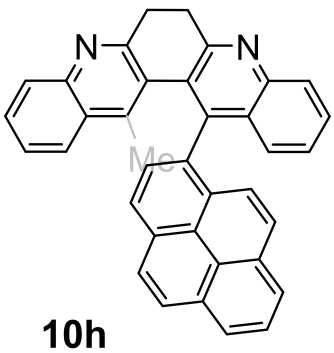

10h

10h was synthesized from **4l** and **9** according to general procedure C. **10h** was obtained as a yellow amorphous solid (132 mg, 85.5 %) after flash chromatography (ethyl acetate: CH_2Cl_2 = 7:3)

^{1}H NMR (500 MHz, $CDCl_3$): δ 8.43-8.23 (m, 3H), 8.12-8.05 (m, 3H), 7.99-7.91 (m, 2H), 7.86-7.72 (m, 3H), 7.58-7.52 (m, 1H), 7.41-7.32 (m, 1.3H), 7.25-7.00 (m, 3.7H), 3.63-3.38 (m, 4H), 1.91 (s, 1H), 1,54 (s, 2H); ^{3}C {^{1}H} NMR (125 MHz, $CDCl_3$): δ 163.0, 162.1, 161.55, 161.13, 147.7, 146.7, 146.2, 145.66, 145.59, 144.5, 143.5, 141.6, 132.1, 131.3, 131.2, 130.85, 130.81, 130.70, 130.38, 129.88, 129.80, 129.74, 129.32, 129.28, 129.01, 128.92, 128.86, 128.43, 128.33, 128.21, 128.10, 128.06, 128.01, 127.93, 127.69, 127.56, 127.41, 127.40, 127.04, 127.02, 126.97, 126.94, 126.84, 126.55, 126.44, 126.36, 126.21, 126.16, 126.00, 125.93, 125.85, 125.57, 125.52, 125.45, 125.34, 125.28, 125.18, 124.84, 124.79, 124.64, 124.35, 124.27, 123.86, 123.68, 122.7,

35.1, 34.78, 34.56, 34.53, 19.10, 18.90. HRMS m/z (FAB): $[M+H]^+$ Calcd for $C_{37}H_{25}N_2$ 497.2018; Found 497.2013.

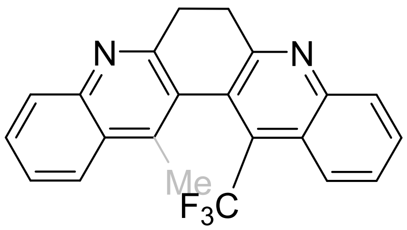

10i

10i was synthesized from **4p** and **9** according to general procedure C. **10i** was obtained as a white powder (140 mg, 94 %) after flash chromatography (ethyl acetate: CH_2Cl_2 = 1:1)

^1H NMR (500 MHz, $CDCl_3$): δ 8.22-8.20 (m, 1H), 8.14 (dd, J = 7 Hz, 1 Hz, 1H), 8.09-8.04 (m, 2H), 7.81 (td, J = 7.5 Hz, 1.5 Hz, 1H), 7.77 (td, J = 7.5 Hz, 1.5 Hz, 1H), 7.67 (td, J = 7.5 Hz, 1.5 Hz, 1H), 7.60 (td, J = 7.5 Hz, 1.5 Hz, 1H), 3.43-3.11 (m, 4H), 2.65 (s, 3H); $^{13}C\{^1H\}$ NMR (125 MHz, $CDCl_3$): δ 162.2, 160.3, 146.89, 146.41, 144.9, 131.98, 131.62 (q, $J_{F\text{-}C}$ = 30 Hz), 130.24, 130.21, 129.11, 128.96, 127.41, 127.17, 126.2, 125.3, 124.77, 124.71, 123.75 (q, $J_{F\text{-}C}$ = 276.25 Hz), 123.14, 34.04, 33.89, 16.8; ^{19}F NMR (470 MHz, $CDCl_3$) : δ -56.29. HRMS m/z (FAB): $[M+H]^+$ Calcd for $C_{22}H_{16}F_3N_2$ 365.1266, Found 365.1268.

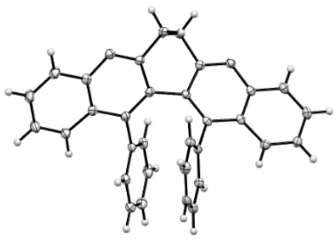

Table S1-1. Crystal data and structure refinement for **2**

Empirical formula	C32 H22 N2	
Formula weight	434.51	
Temperature	100.0(2) K	
Wavelength	0.71073 Å	
Crystal system	Monoclinic	
Space group	P 21/n	
Unit cell dimensions	a = 9.9232(3) Å	α= 90°.
	b = 14.5911(4) Å	β= 95.2810(10)°.
	c = 15.4502(4) Å	γ = 90°.
Volume	2227.55(11) $Å^3$	
Z	4	
Density (calculated)	1.296 Mg/m^3	
Absorption coefficient	0.076 mm^{-1}	
F(000)	912	
Crystal size	0.286 x 0.214 x 0.176 mm^3	
Theta range for data collection	1.924 to 27.099°.	
Index ranges	-12<=h<=12, -18<=k<=18, -19<=l<=19	
Reflections collected	59244	
Independent reflections	4931 [R(int) = 0.0547]	
Completeness to theta = 25.242°	99.9 %	
Absorption correction	Numerical	
Max. and min. transmission	1 and 0.9695	
Refinement method	Full-matrix least-squares on F^2	
Data / restraints / parameters	4931 / 0 / 307	
Goodness-of-fit on F^2	1.034	
Final R indices [I>2sigma(I)]	R1 = 0.0396, wR2 = 0.0919	
R indices (all data)	R1 = 0.0493, wR2 = 0.0986	
Extinction coefficient	n/a	
Largest diff. peak and hole	0.279 and -0.226 e.$Å^{-3}$	

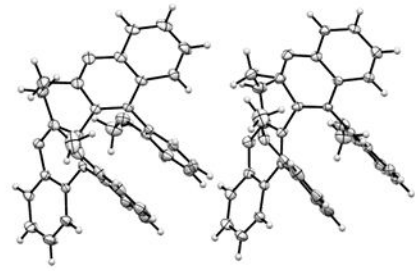

Table S1-2. Crystal data and structure refinement for **5e**

Empirical formula	C136 H104 N8 O8	
Formula weight	1978.27	
Temperature	100.0(2) K	
Wavelength	0.71073 Å	
Crystal system	Triclinic	
Space group	P -1	
Unit cell dimensions	a = 8.5277(3) Å	α= 76.9780(10)°.
	b = 17.0407(5) Å	β= 76.8890(10)°.
	c = 18.4763(5) Å	γ = 77.6480(10)°.
Volume	2509.92(13) Å^3	
Z	1	
Density (calculated)	1.309 Mg/m^3	
Absorption coefficient	0.082 mm^{-1}	
F(000)	1040	
Crystal size	0.282 x 0.243 x 0.156 mm^3	
Theta range for data collection	1.847 to 27.103°.	
Index ranges	-10<=h<=10, -21<=k<=21, -23<=l<=23	
Reflections collected	135113	
Independent reflections	11067 [R(int) = 0.1256]	
Completeness to theta = 25.242°	100.0 %	
Absorption correction	Numerical	
Max. and min. transmission	0.9977 and 0.949	
Refinement method	Full-matrix least-squares on F^2	
Data / restraints / parameters	11067 / 0 / 689	
Goodness-of-fit on F^2	1.068	
Final R indices [I>2sigma(I)]	R1 = 0.0909, wR2 = 0.2249	
R indices (all data)	R1 = 0.1327, wR2 = 0.2496	
Extinction coefficient	n/a	
Largest diff. peak and hole	1.183 and -0.373 e.Å^{-3}	

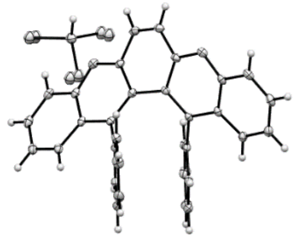

Table S1-3. Crystal data and structure refinement for **7.**

Empirical formula	C34 H22 Cl6 N2	
Formula weight	671.23	
Temperature	100.0(2) K	
Wavelength	0.71073 Å	
Crystal system	Monoclinic	
Space group	C 2/c	
Unit cell dimensions	a = 19.2458(4) Å	α= 90°.
	b = 10.6101(2) Å	β= 104.6150(10)°.
	c = 15.0894(3) Å	γ = 90°.
Volume	2981.55(10) Å^3	
Z	4	
Density (calculated)	1.495 Mg/m^3	
Absorption coefficient	0.605 mm^{-1}	
F(000)	1368	
Crystal size	0.271 x 0.176 x 0.174 mm^3	
Theta range for data collection	2.187 to 28.281°.	
Index ranges	-25<=h<=25, -14<=k<=14, -20<=l<=20	
Reflections collected	66209	
Independent reflections	3701 [R(int) = 0.0575]	
Completeness to theta = 25.242°	99.9 %	
Absorption correction	Numerical	
Max. and min. transmission	1 and 0.9491	
Refinement method	Full-matrix least-squares on F^2	
Data / restraints / parameters	3701 / 0 / 188	
Goodness-of-fit on F^2	1.059	
Final R indices [I>2sigma(I)]	R1 = 0.0672, wR2 = 0.1486	
R indices (all data)	R1 = 0.0769, wR2 = 0.1550	
Extinction coefficient	n/a	
Largest diff. peak and hole	1.799 and -1.327 e.Å^{-3}	
