# Supplementary material for: Investigating Bay-Substituted 6,7-Dihydrodibenzo[b,j][4,7]phenanthroline, a Class of Tunable Hindered Rotors: Synthesis and Molecular Dynamics
Source: J Org Chem. 2025 Jun 3;90(23):7547–54. doi: 10.1021/acs.joc.4c01476 (PMC12172051; doi:10.1021/acs.joc.4c01476)
Supplement: Supplementary file 2 [file jo4c01476_si_002.pdf]

Title: Investigating bay-substituted 6,7-dihydrodibenzo[b,j][4,7]phenanthroline, a class of tunable hindered rotors: Synthesis and molecular dynamics

Authors: Yen-Cheng Lu[b], Jhih-Syong Jhang[a], Chih-Hsiu Lin[a] *

a. Institute of Chemistry, Academia Sinica, Taipei, Taiwan, Republic of China, 115024

b. Department of Chemistry, National Central University Taoyuan, Taiwan, Republic of China

Email of corresponding author: chemopera@gate.sinica.edu.tw

Supporting information 2: ^{1}H and ^{13}C NMR spectrum for new compounds and variable temperature NMR experiments

Table of Contents

^{1}H and ^{13}C NMR spectrum of **2**……………………………………………………………………**pS2-2-pS2-3**

^{1}H and ^{13}C NMR spectrum of **4a-4q**……………………………………………………………**pS2-4-pS2-30**

^{1}H and ^{13}C NMR spectrum of **5a-5q**…………………………………………………………….**pS2-31-pS2-65**

^{1}H and ^{13}C NMR spectrum of **6a, 6b, 6c** and **7**…………………………………………………**pS2-66-pS2-73**

^{1}H and ^{13}C NMR spectrum of **8** and **9**………………………………………………………………**pS2-74-pS2-77**

^{1}H and ^{13}C NMR spectrum of **10a-10i**…………………………………………………………..**pS2-78-pS2-97**

Varible temperature NMR experiment at high temperatures (figure S2-1-S2-8, table S2-1)……..**pS2-98-pS2-107**

Varible temperature NMR experiment at low temperatures (figure S2-9-S2-19, table S2-2)………..**pS2-108-pS2-119**

^{1}H NMR signal assignment and chiral HPLC analysis of **5l** (figure S2-20 and S2-21)…………………**pS2-120-pS2-121.**

^{1}H NMR signal assignment of **5k** (figure S2-22)……………………………………………………**pS2-122**

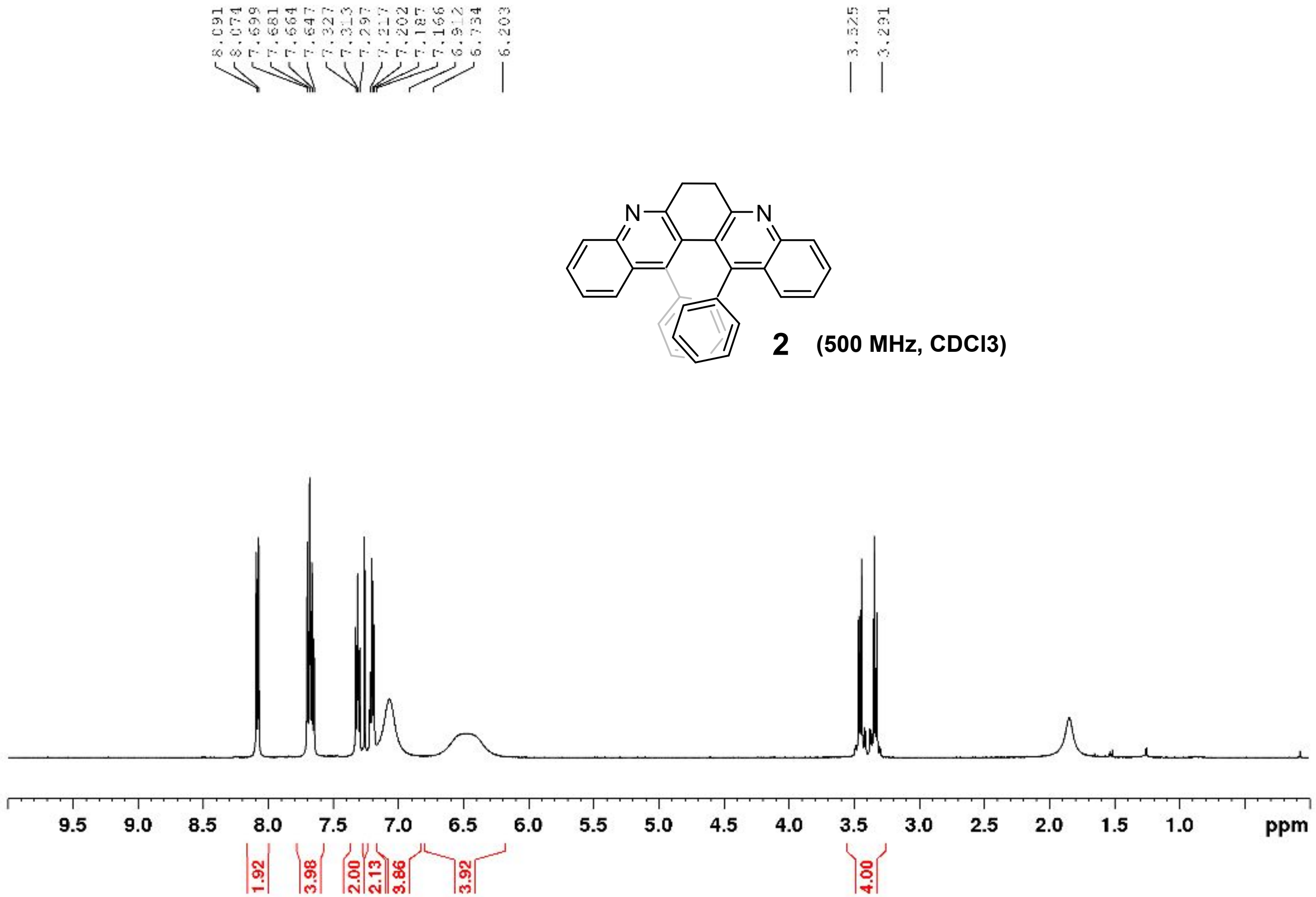
8.091
8.074
7.699
7.681
7.664
7.647
7.327
7.313
7.297
7.217
7.202
7.187
7.166
6.912
6.734
6.203
3.525
3.291
N
N
2 (500 MHz, CDCl3)
9.5 9.0 8.5 8.0 7.5 7.0 6.5 6.0 5.5 5.0 4.5 4.0 3.5 3.0 2.5 2.0 1.5 1.0 ppm
1.92
3.98
2.00
2.13
3.86
3.92
4.00

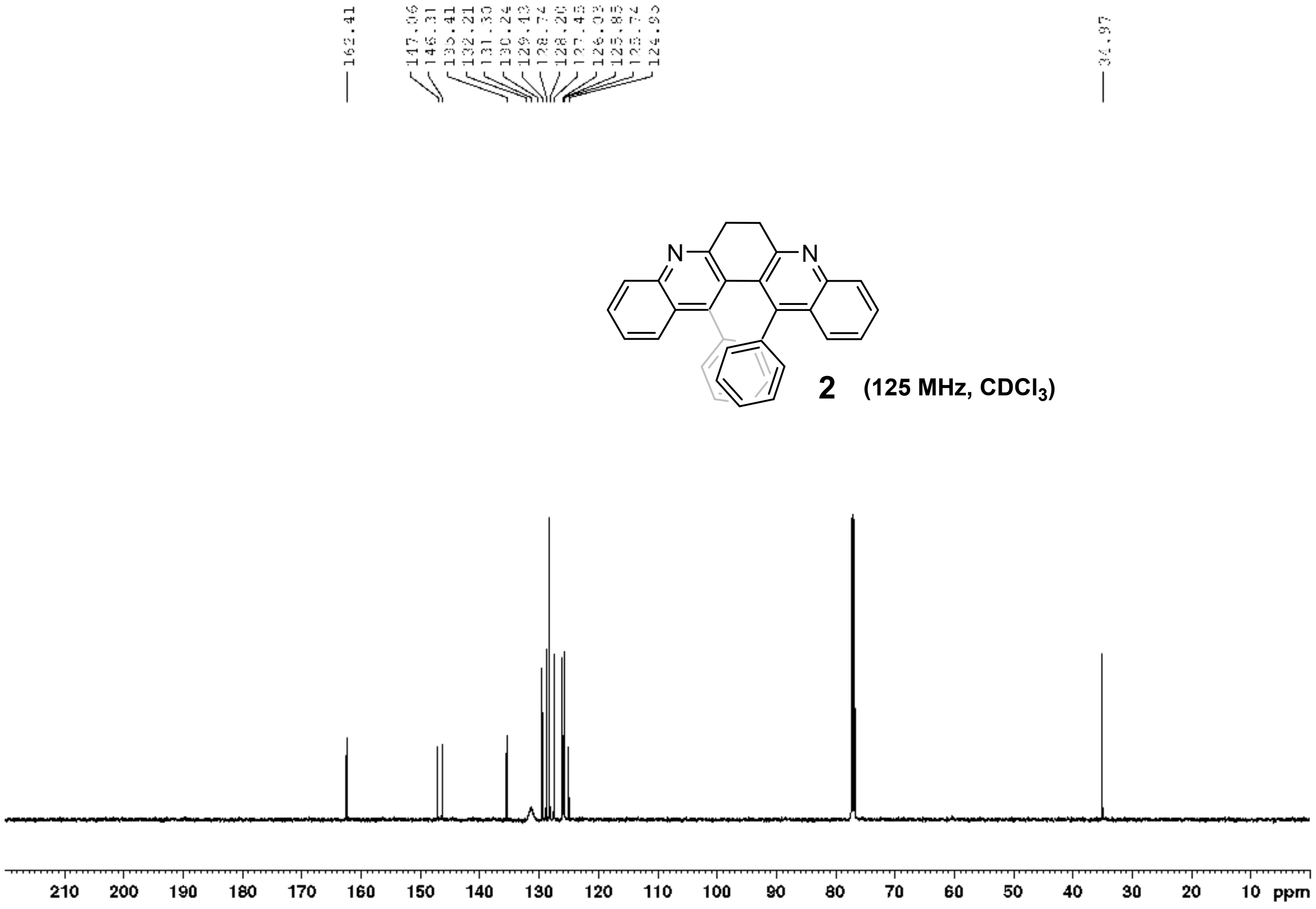
162.41
147.06
146.31
135.41
132.21
131.30
130.24
129.43
128.74
128.20
127.45
126.03
125.85
125.74
124.95
34.97
N
N
2 (125 MHz, $CDCl_3$)
210 200 190 180 170 160 150 140 130 120 110 100 90 80 70 60 50 40 30 20 10 ppm

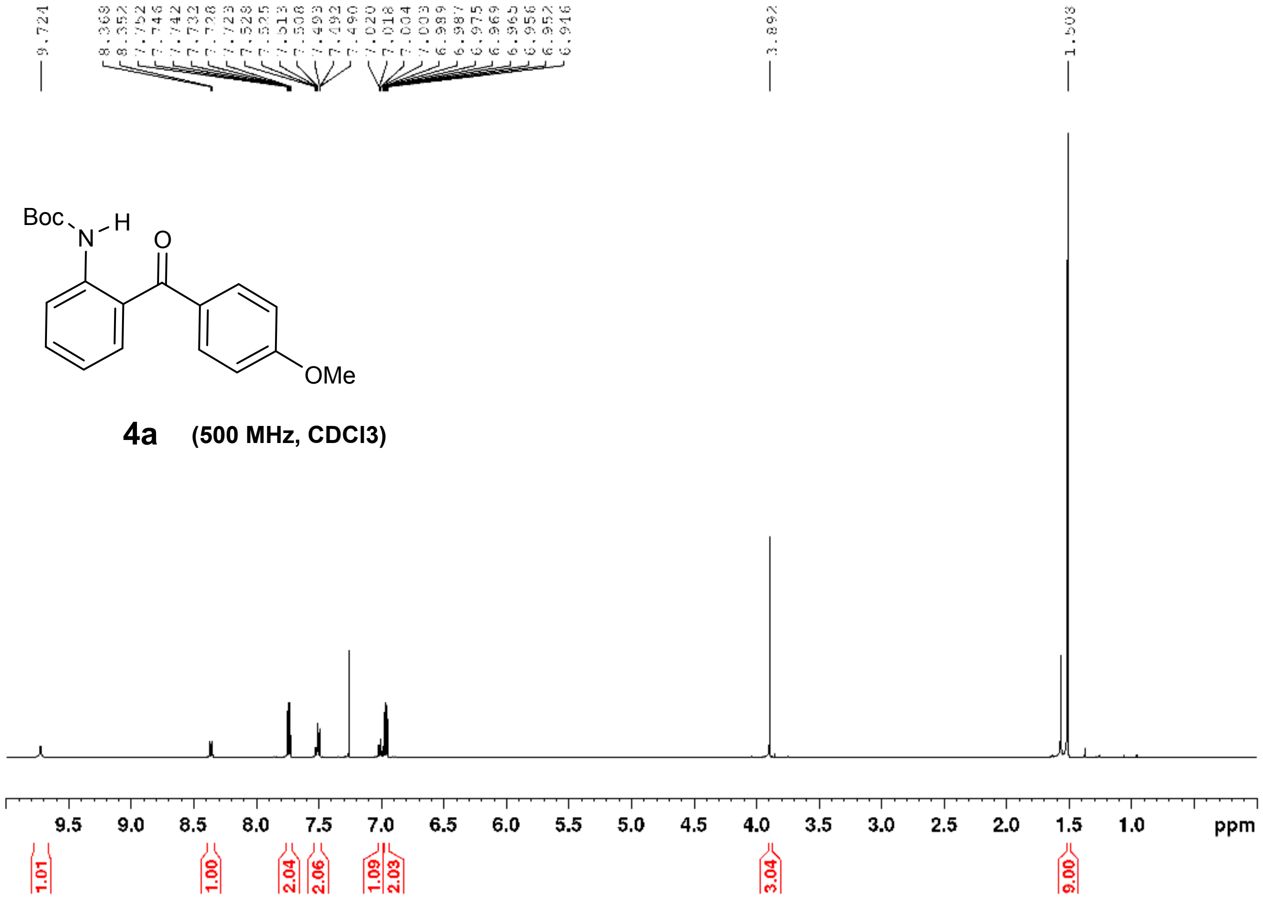
9.724
8.368
8.352
7.752
7.746
7.742
7.732
7.728
7.723
7.528
7.525
7.513
7.508
7.493
7.492
7.490
7.020
7.018
7.004
7.003
6.989
6.987
6.975
6.969
6.965
6.956
6.952
6.946
3.892
1.503
Boc
N
H
O
OMe
4a (500 MHz, CDCl3)
9.5
9.0
8.5
8.0
7.5
7.0
6.5
6.0
5.5
5.0
4.5
4.0
3.5
3.0
2.5
2.0
1.5
1.0
ppm
1.01
1.00
2.04
2.06
1.09
2.03
3.04
9.00

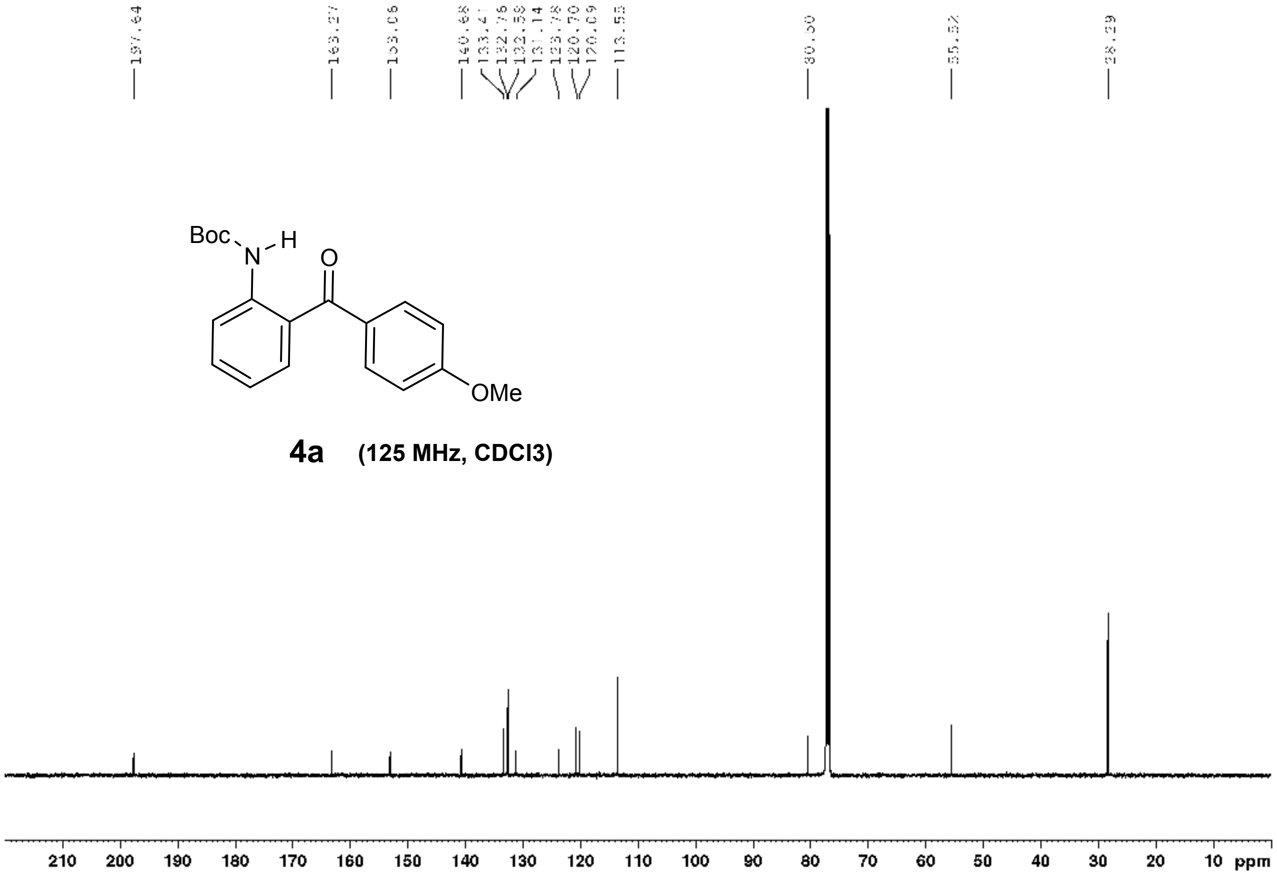

Boc
N
H
O
OMe
4a (125 MHz, CDCl3)
210 200 190 180 170 160 150 140 130 120 110 100 90 80 70 60 50 40 30 20 10 ppm

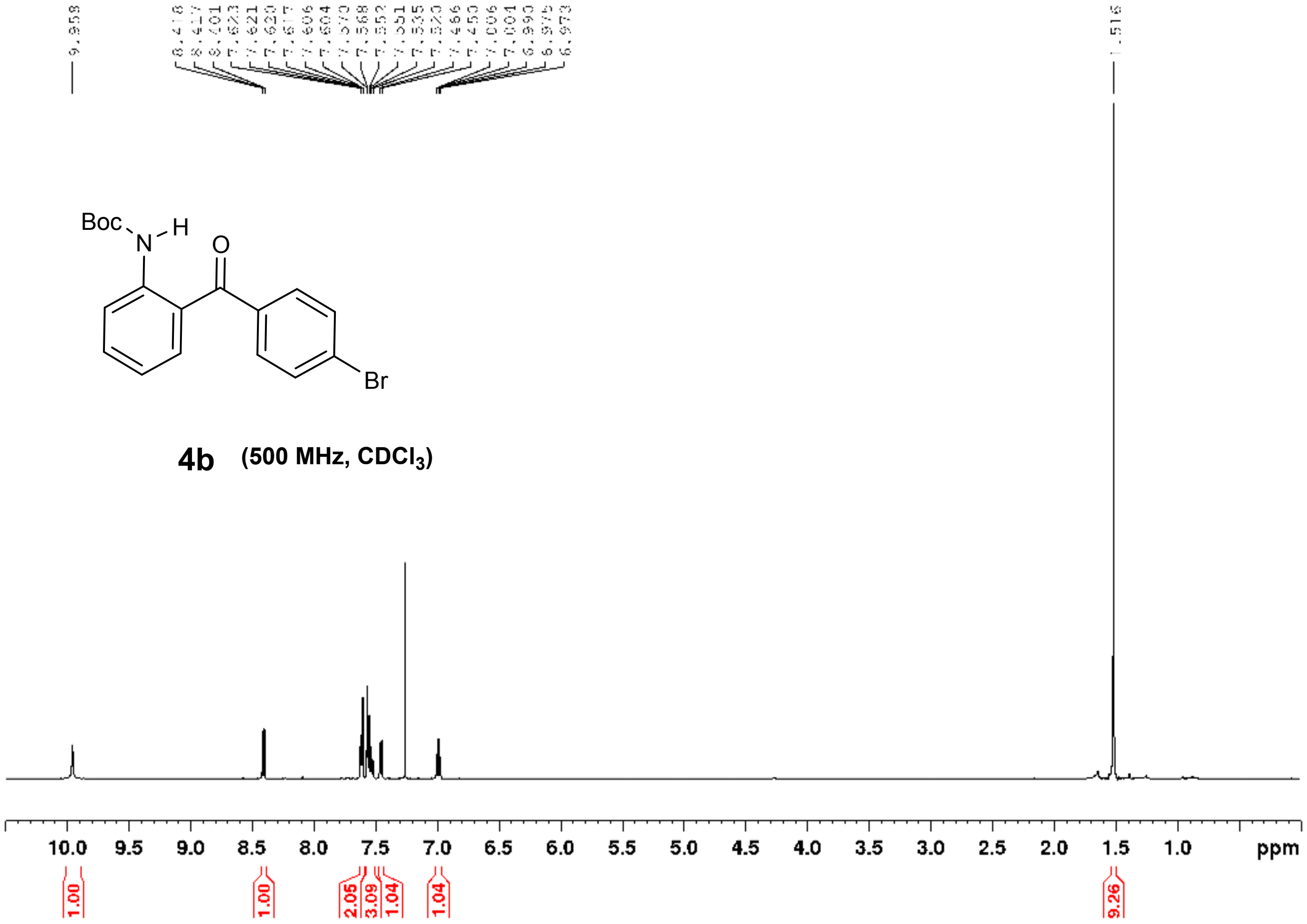

4b **(500 MHz, $CDCl_3$)**

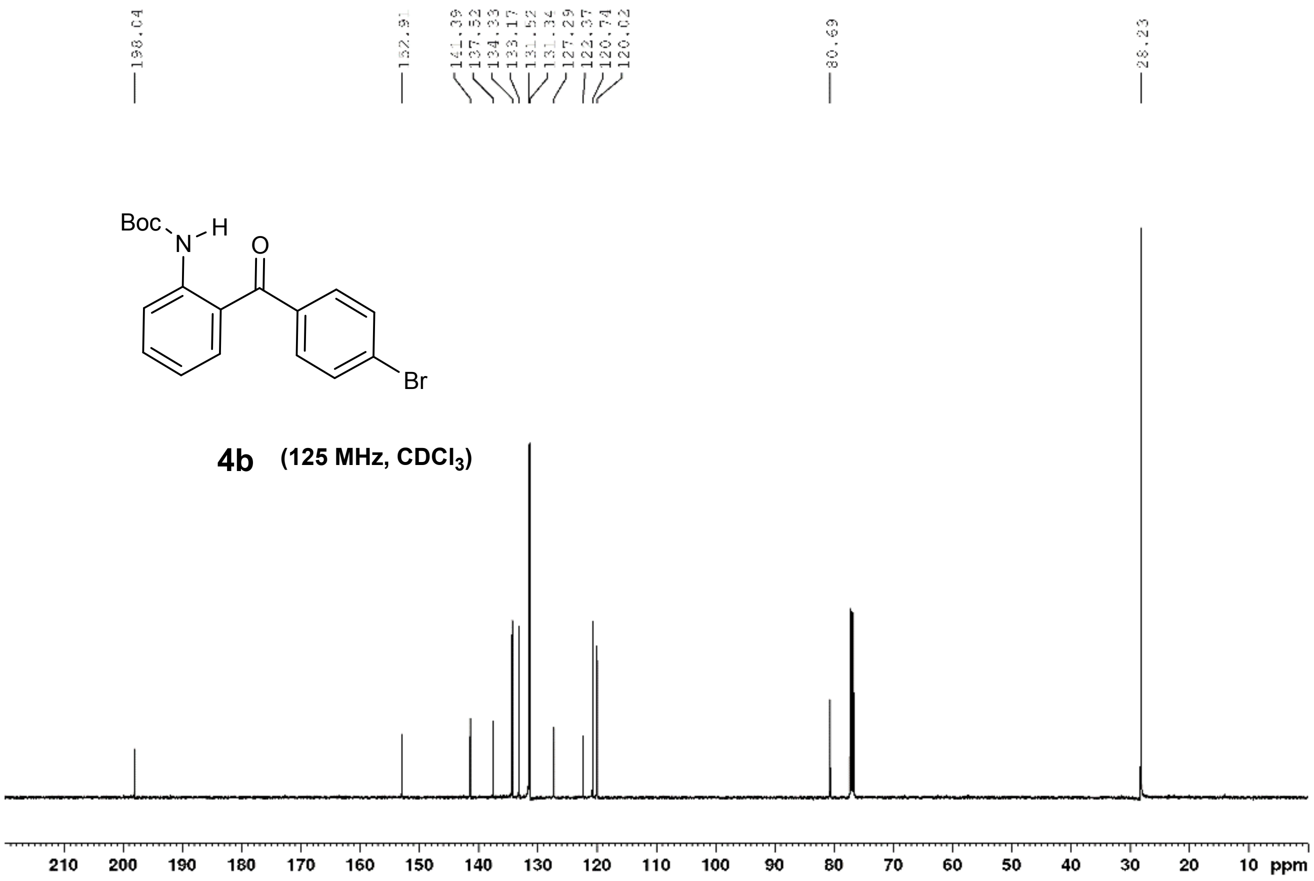

4b **(125 MHz, $CDCl_3$)**

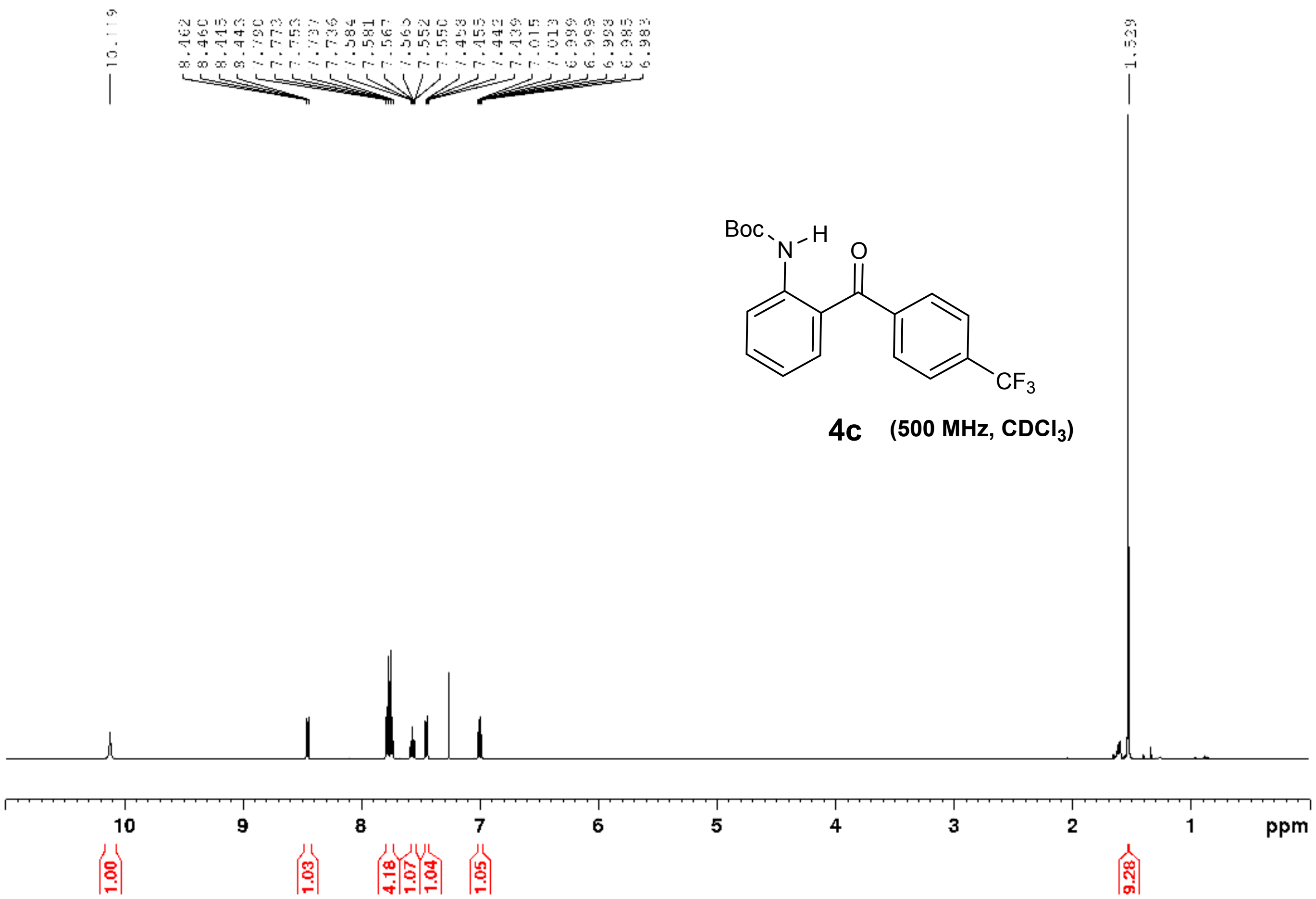
10.119
8.462
8.460
8.445
8.443
7.790
7.773
7.753
7.737
7.736
7.584
7.581
7.567
7.565
7.552
7.550
7.458
7.455
7.442
7.439
7.015
7.013
6.999
6.998
6.985
6.983
1.529
Boc
N
H
O
CF_3
4c
(500 MHz, $CDCl_3$)
10
9
8
7
6
5
4
3
2
1
ppm
1.00
1.03
4.18
1.07
1.04
1.05
9.28

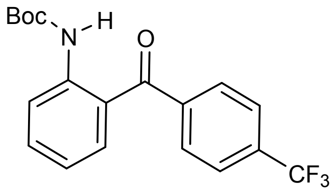

4c **(125 MHz, $CDCl_3$)**

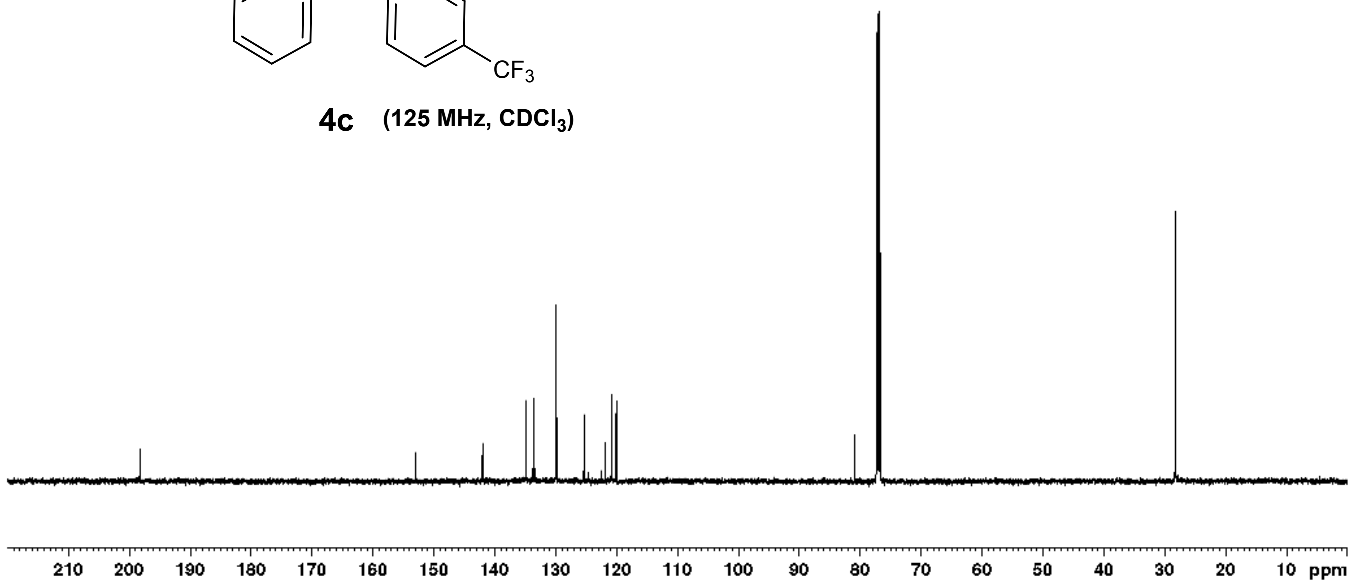

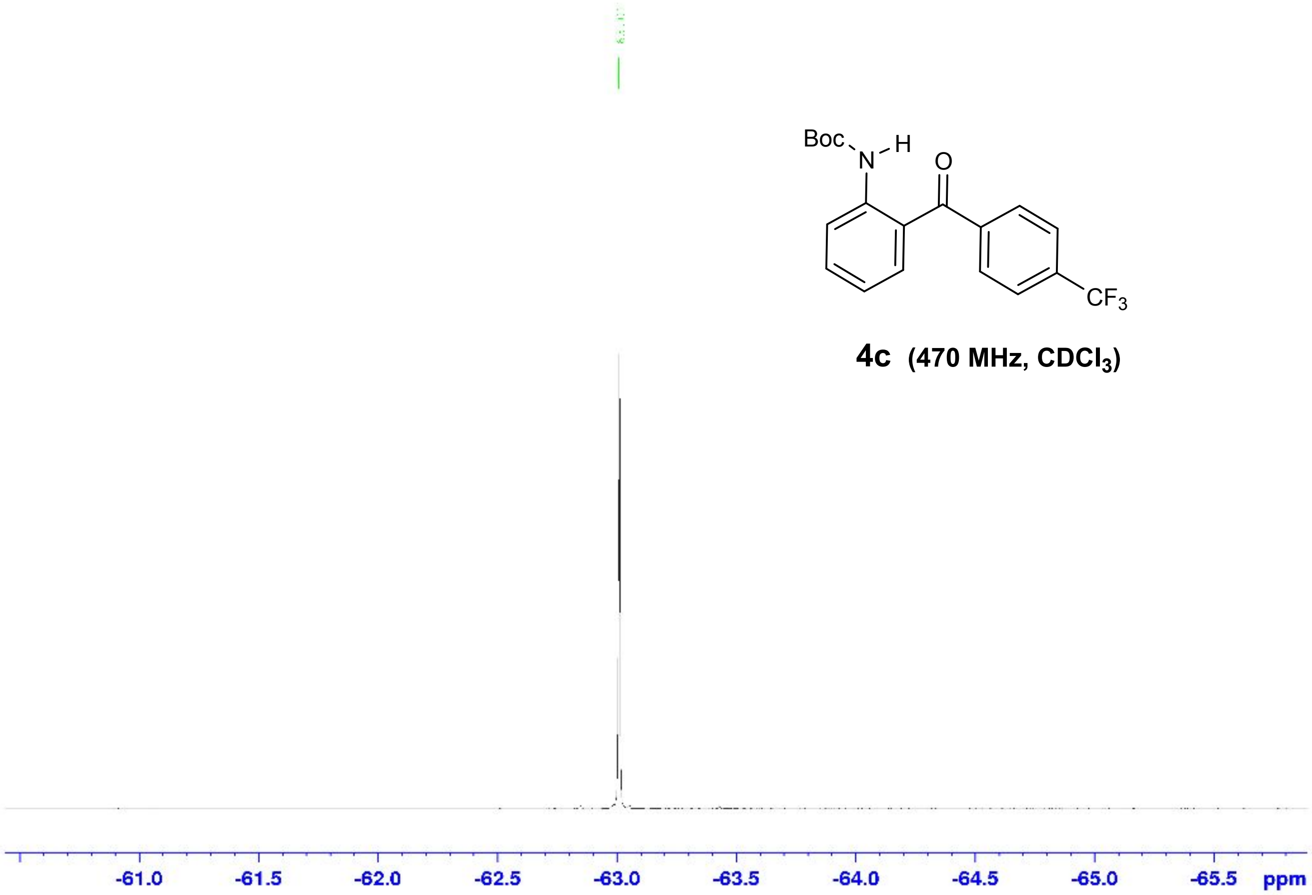

4c **(470 MHz, $CDCl_3$)**

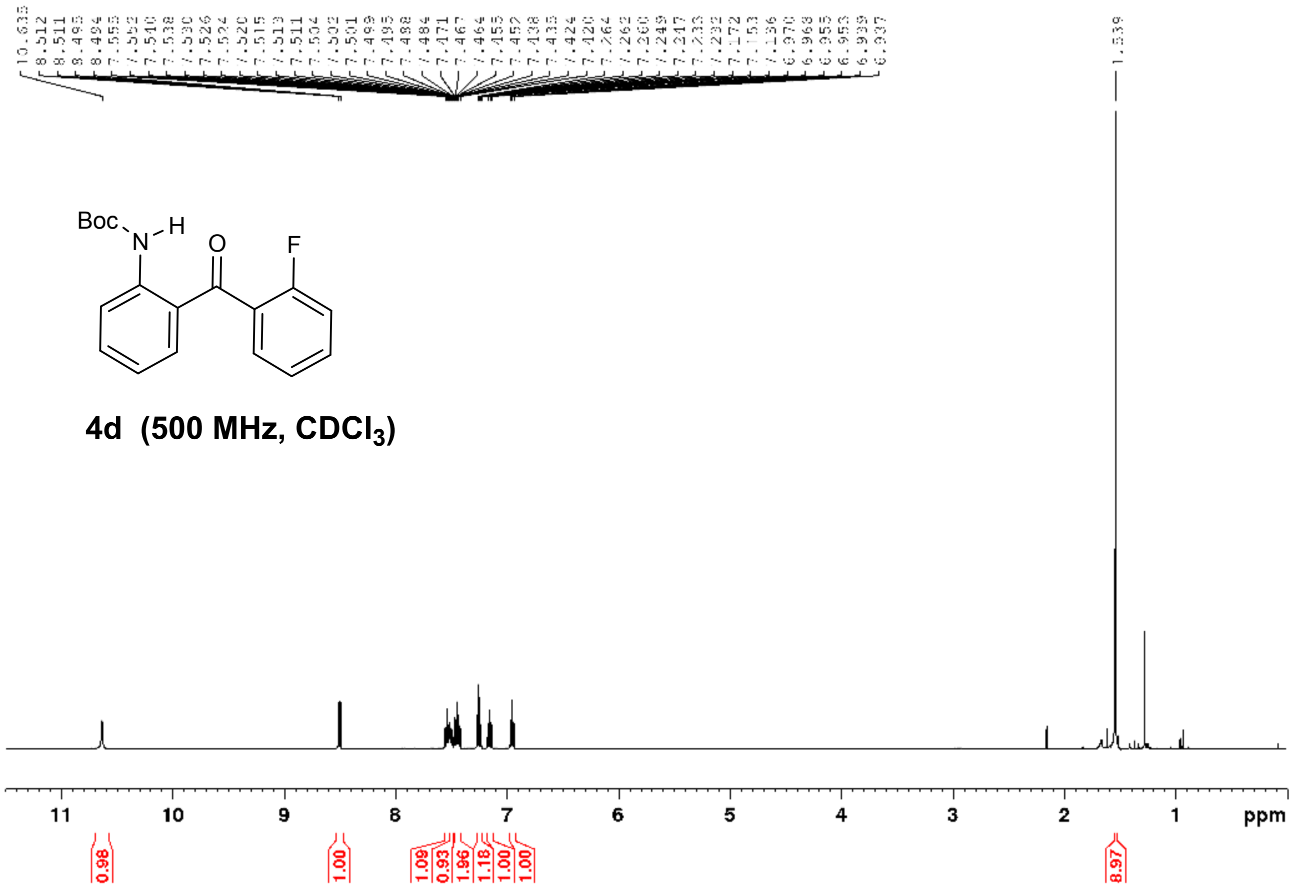

4d (500 MHz, $CDCl_3$)

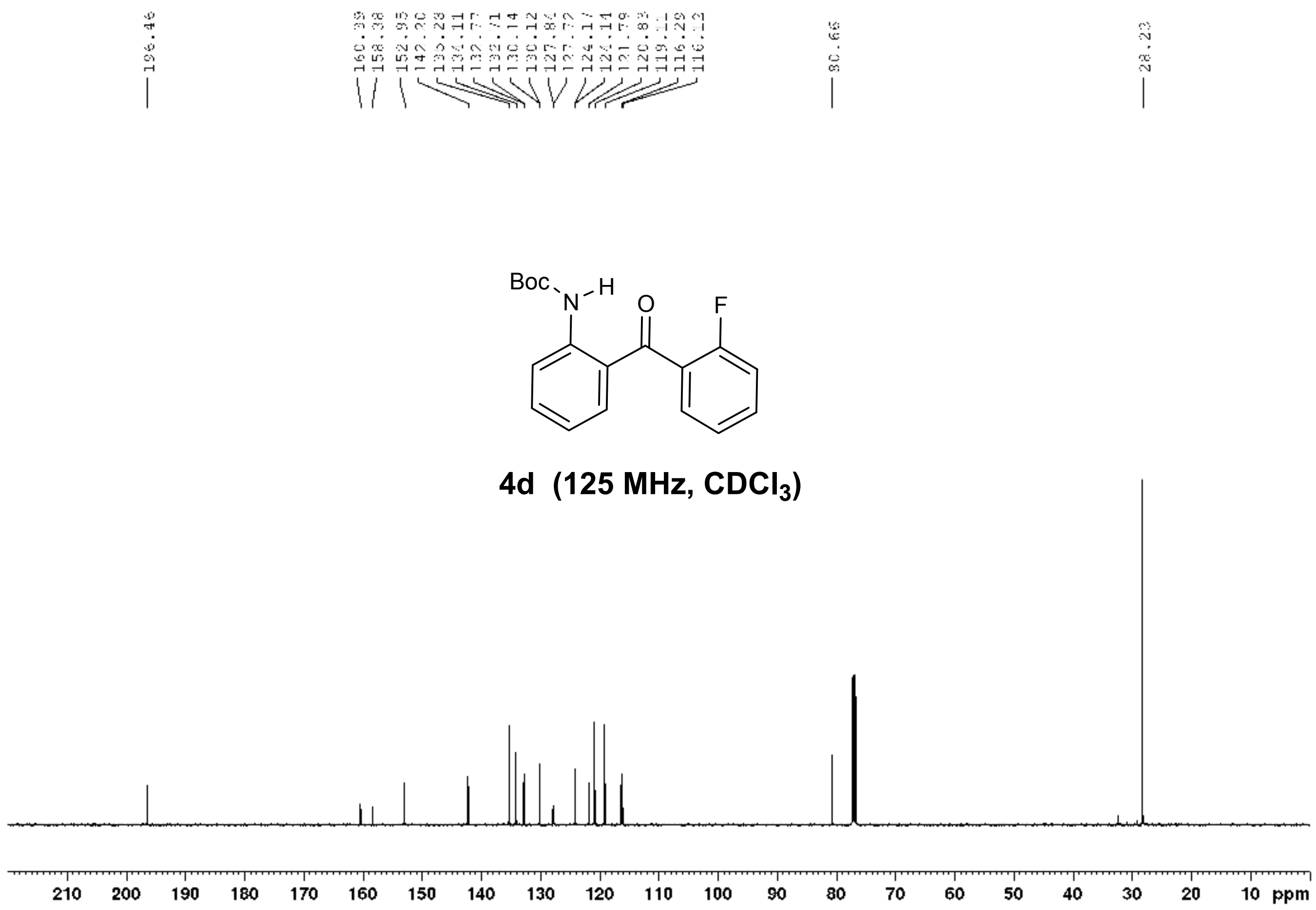

4d (125 MHz, $CDCl_3$)

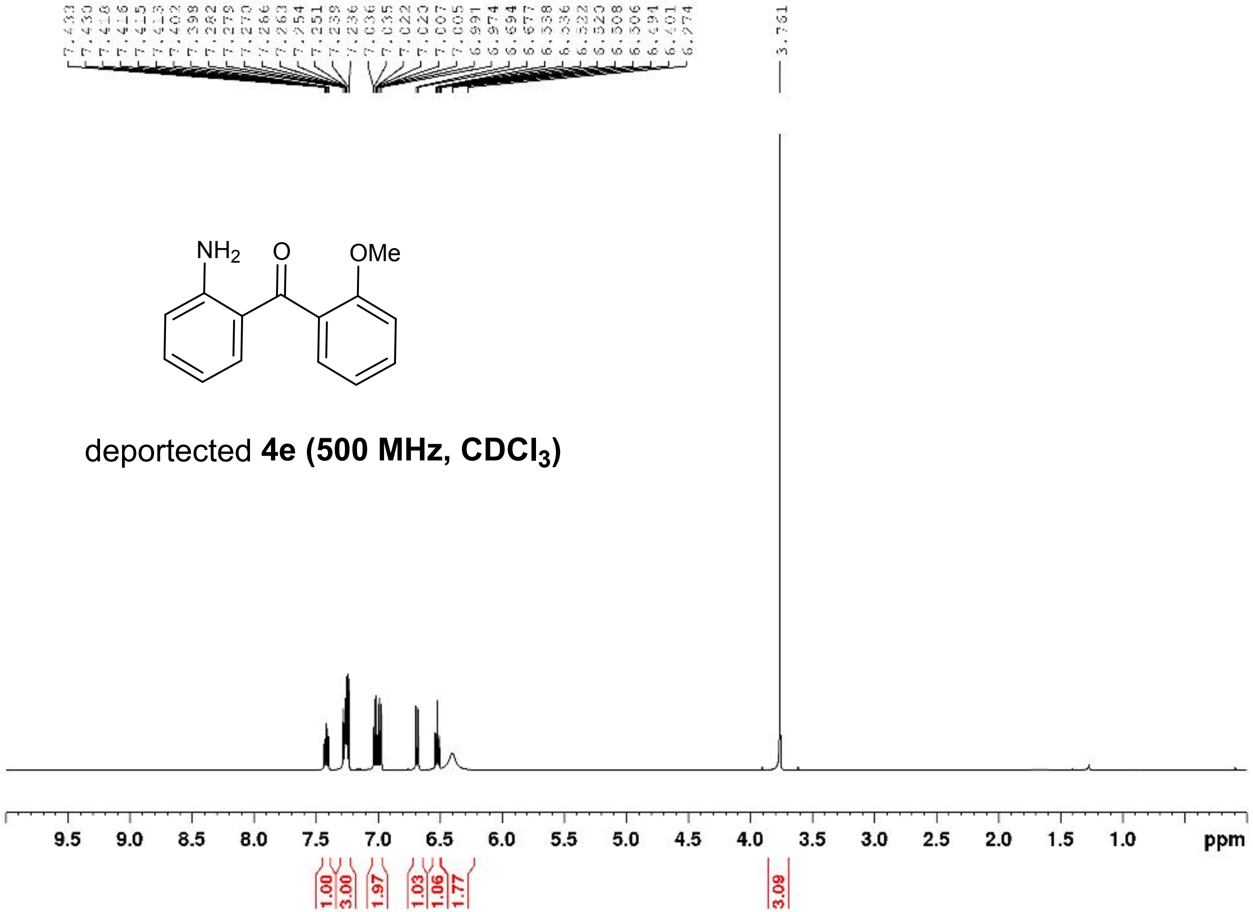

deportected **4e (500 MHz, $CDCl_3$)**

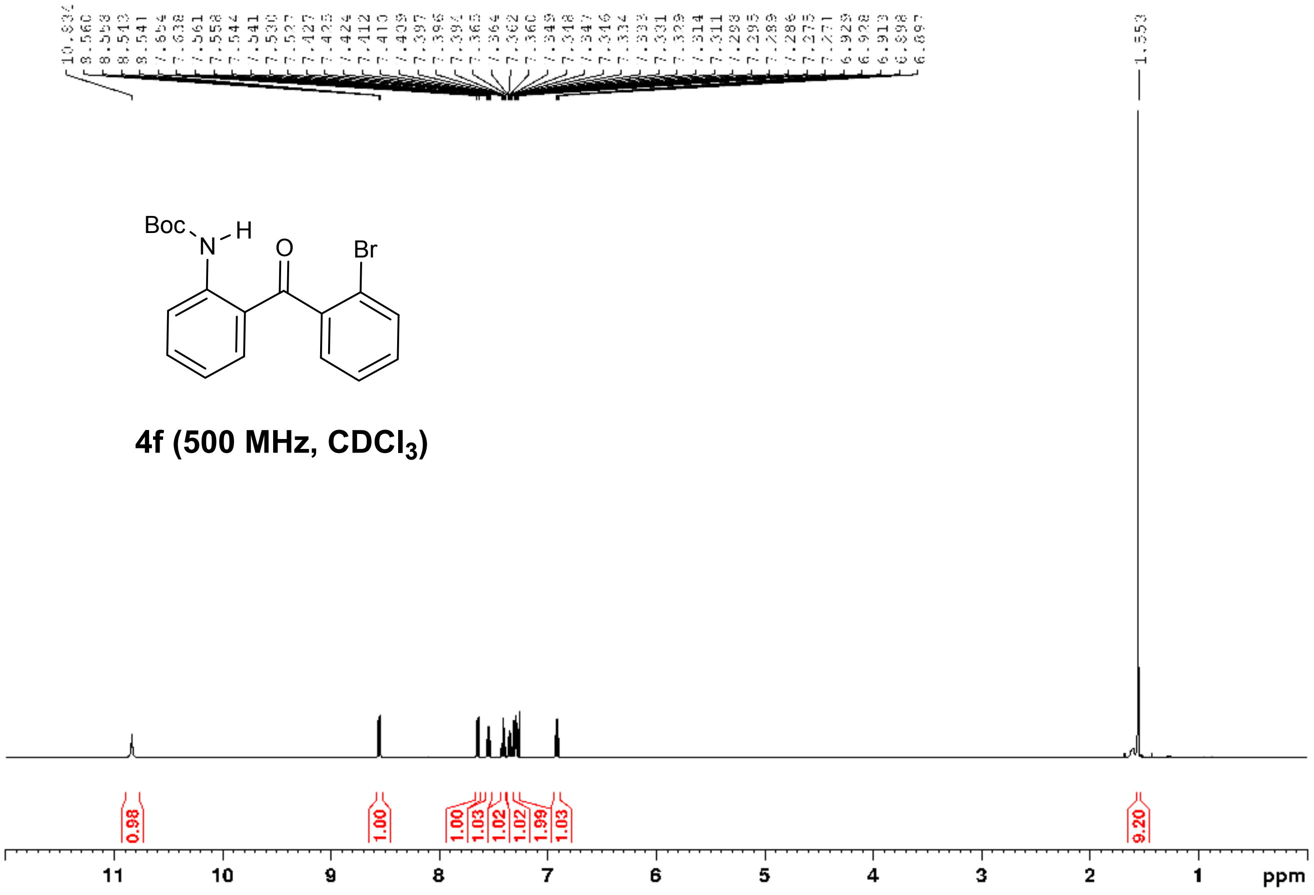

4f (500 MHz, $CDCl_3$)

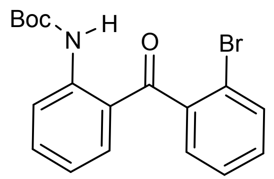

4f (125 MHz, $CDCl_3$)

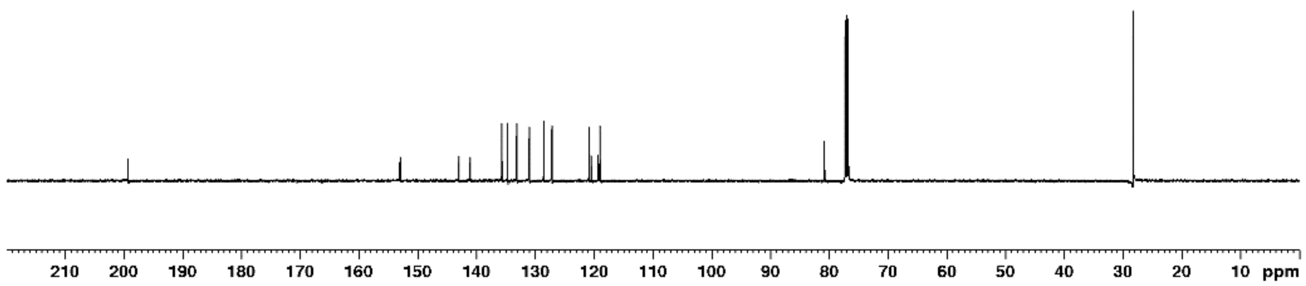

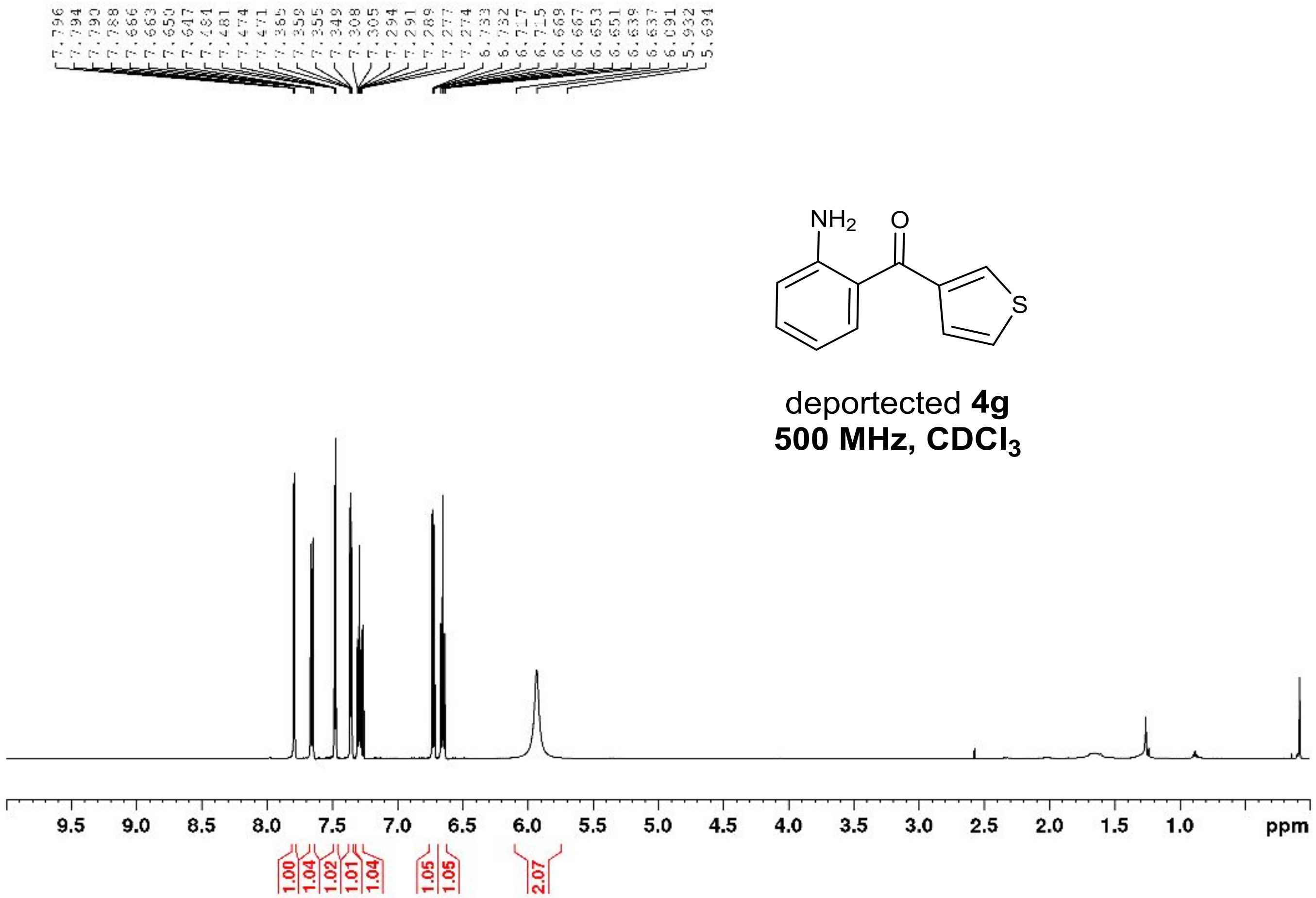

deportected **4g**
500 MHz, $CDCl_3$

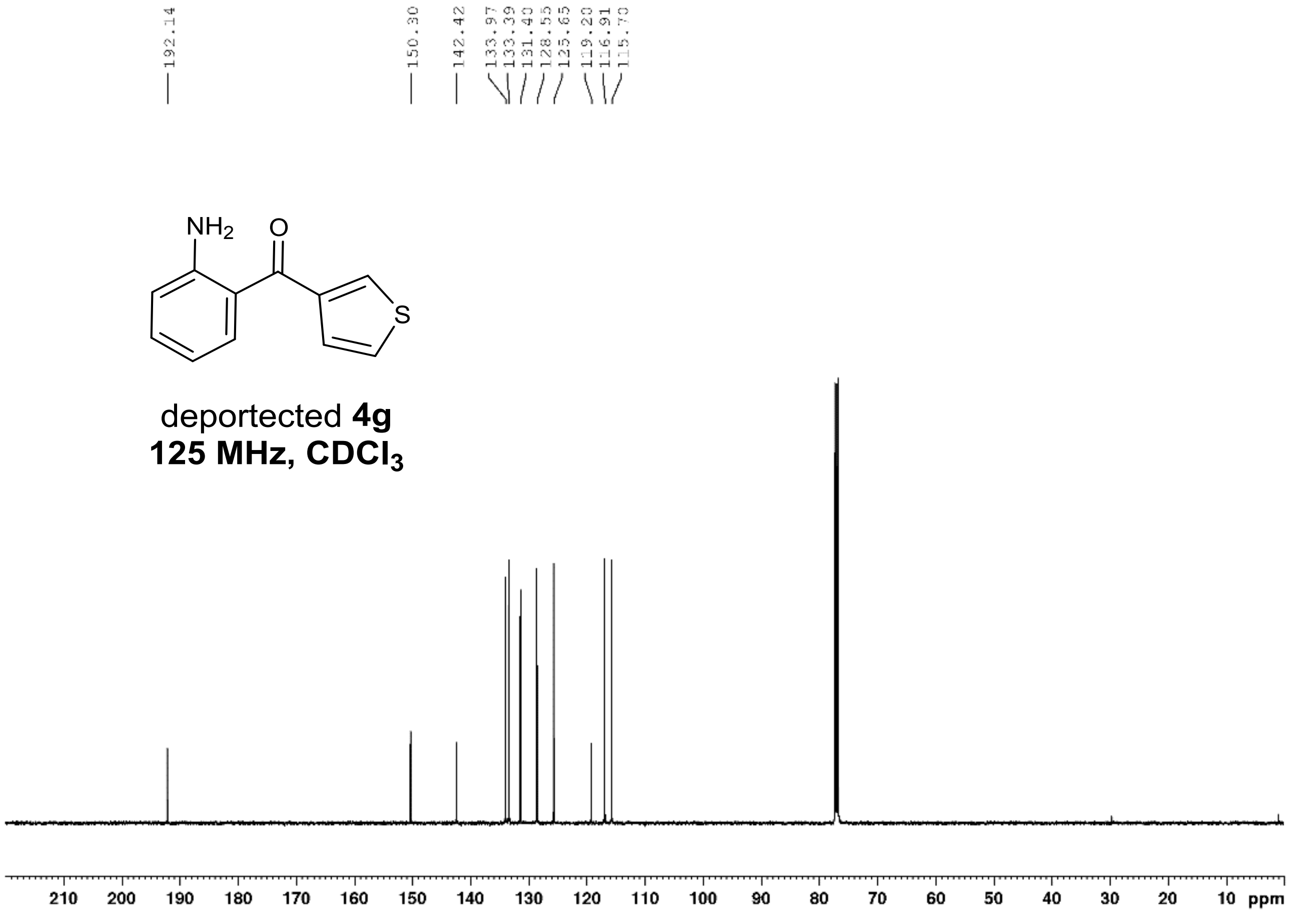

deportected **4g**

125 MHz, $CDCl_3$

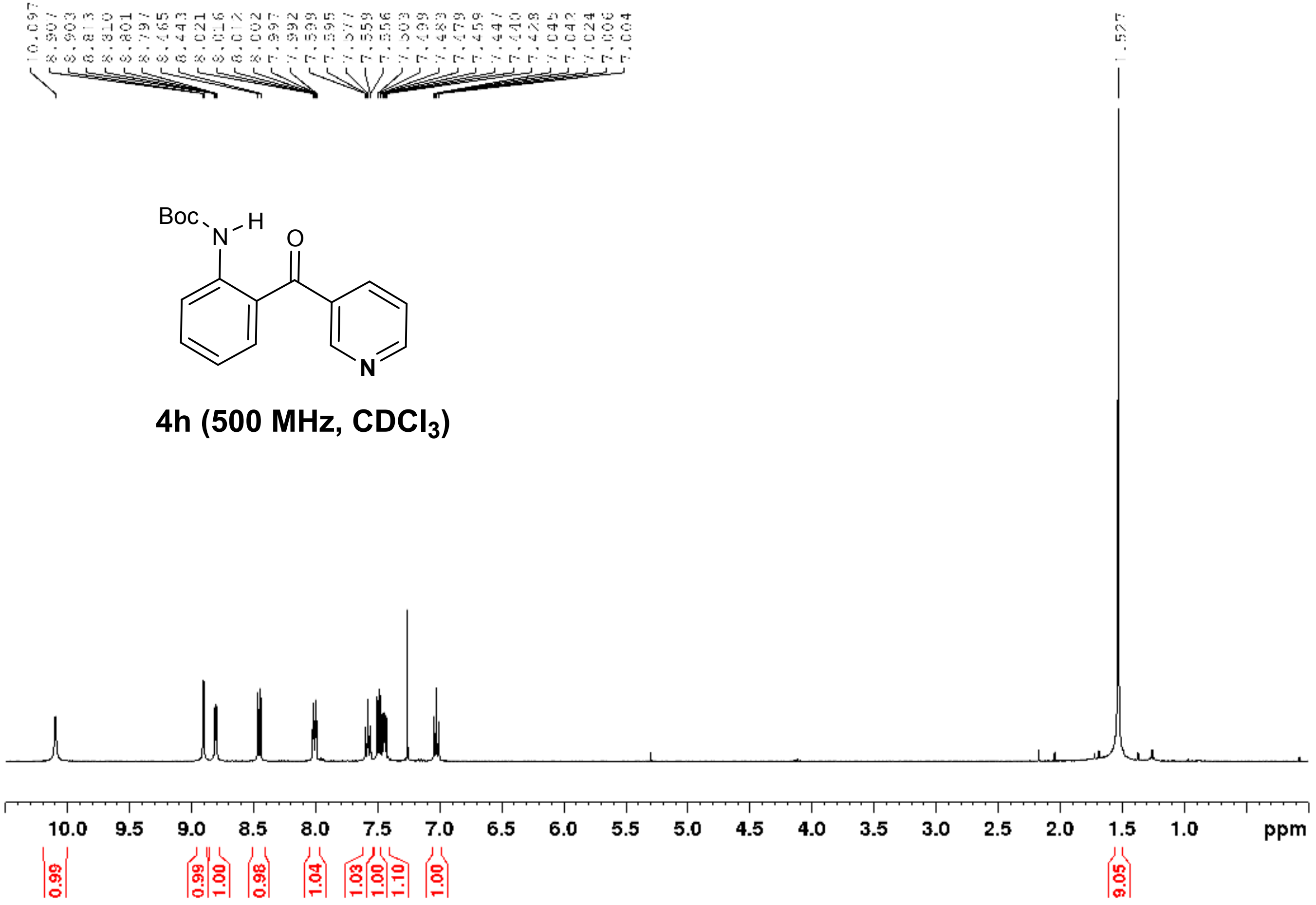

4h (500 MHz, $CDCl_3$)

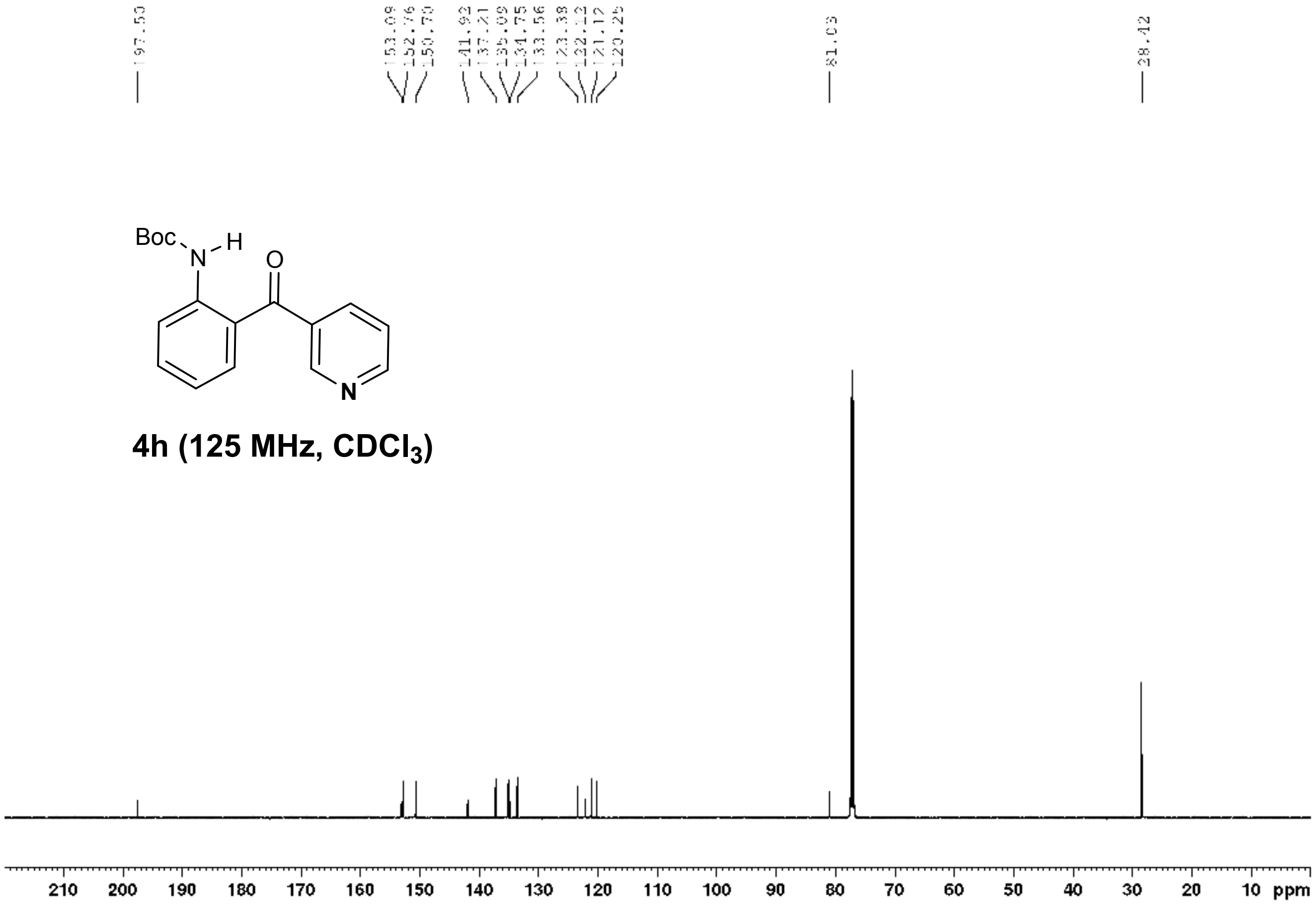

4h (125 MHz, $CDCl_3$)

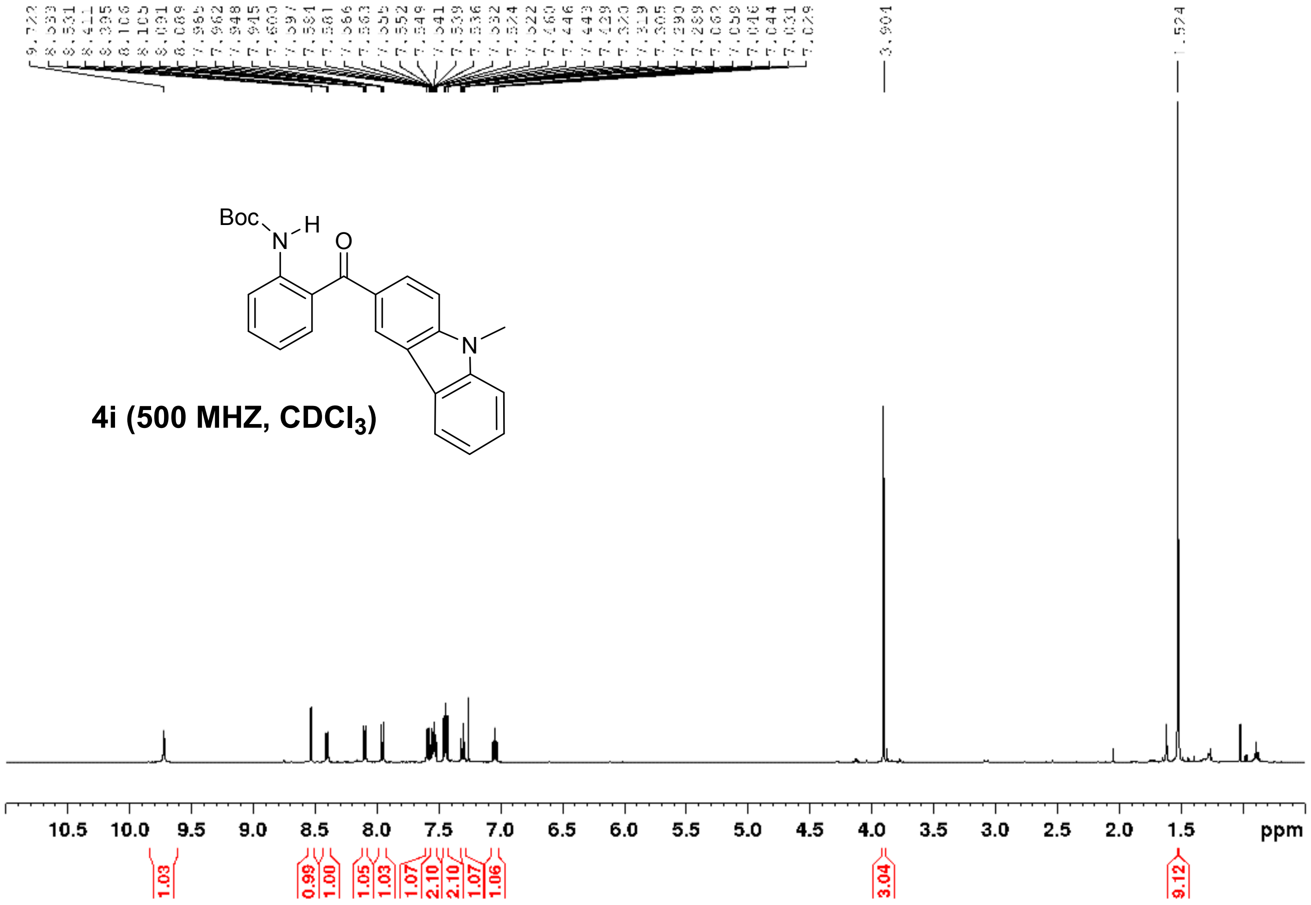

4i (500 MHZ, CDCl$_3$)

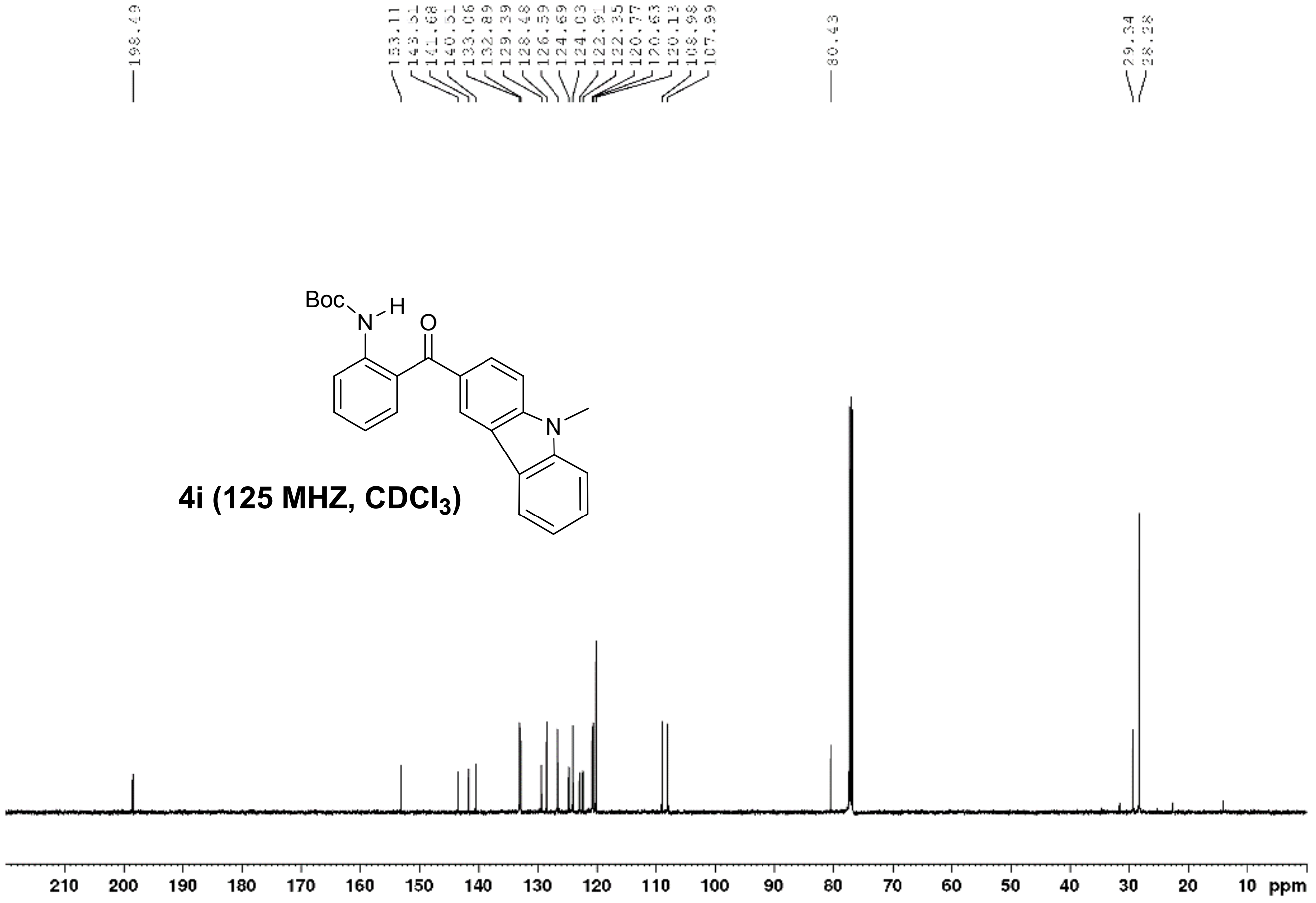

4i (125 MHZ, $CDCl_3$)

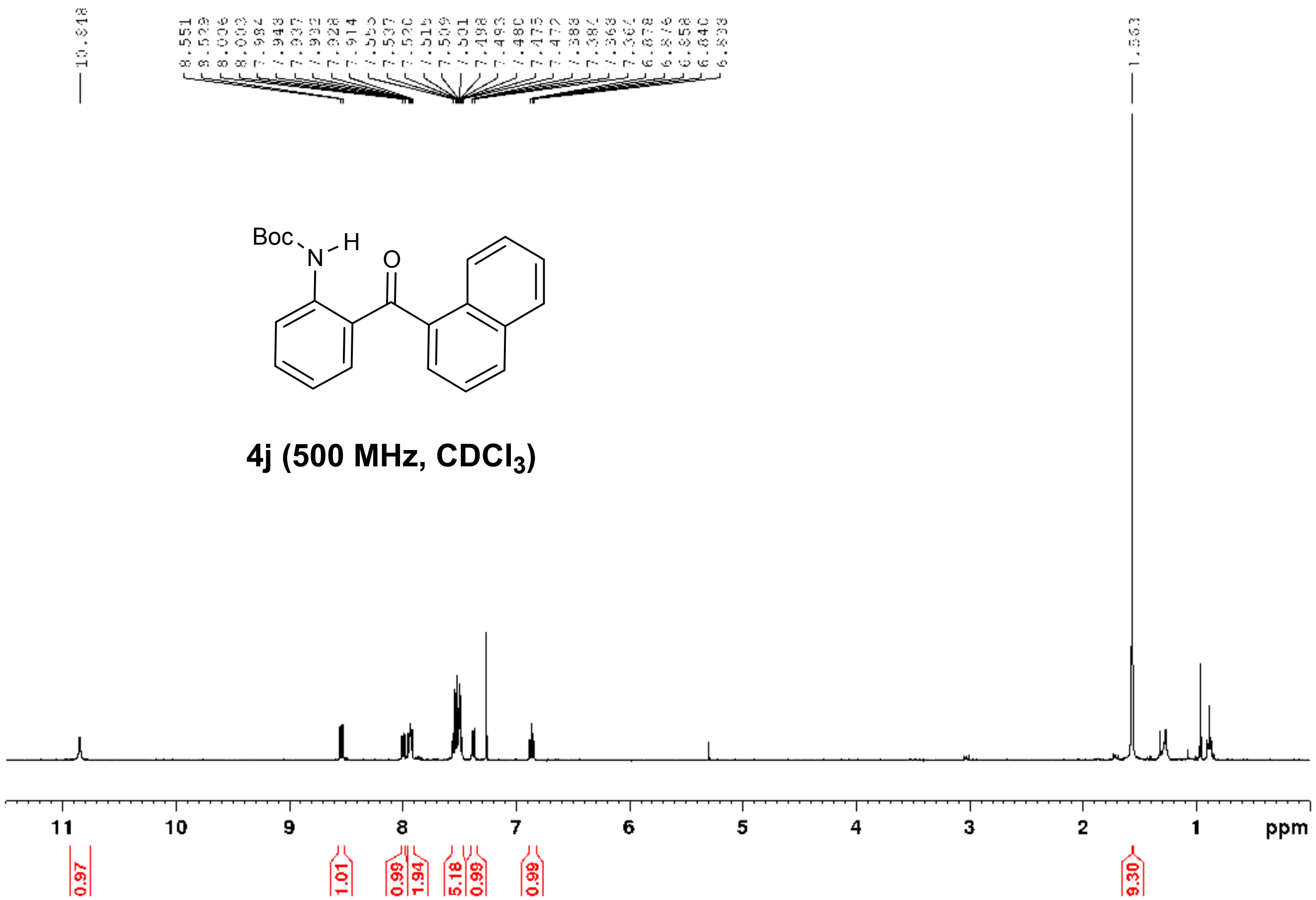
Boc
N
H
O
4j (500 MHz, CDCl3)
10.848
8.551
8.529
8.006
8.003
7.984
7.943
7.937
7.932
7.928
7.914
7.555
7.537
7.520
7.515
7.509
7.501
7.498
7.493
7.480
7.475
7.472
7.388
7.384
7.368
7.364
6.878
6.876
6.858
6.840
6.838
1.563
11
10
9
8
7
6
5
4
3
2
1
ppm
0.97
1.01
0.99
1.94
5.18
0.99
0.99
9.30

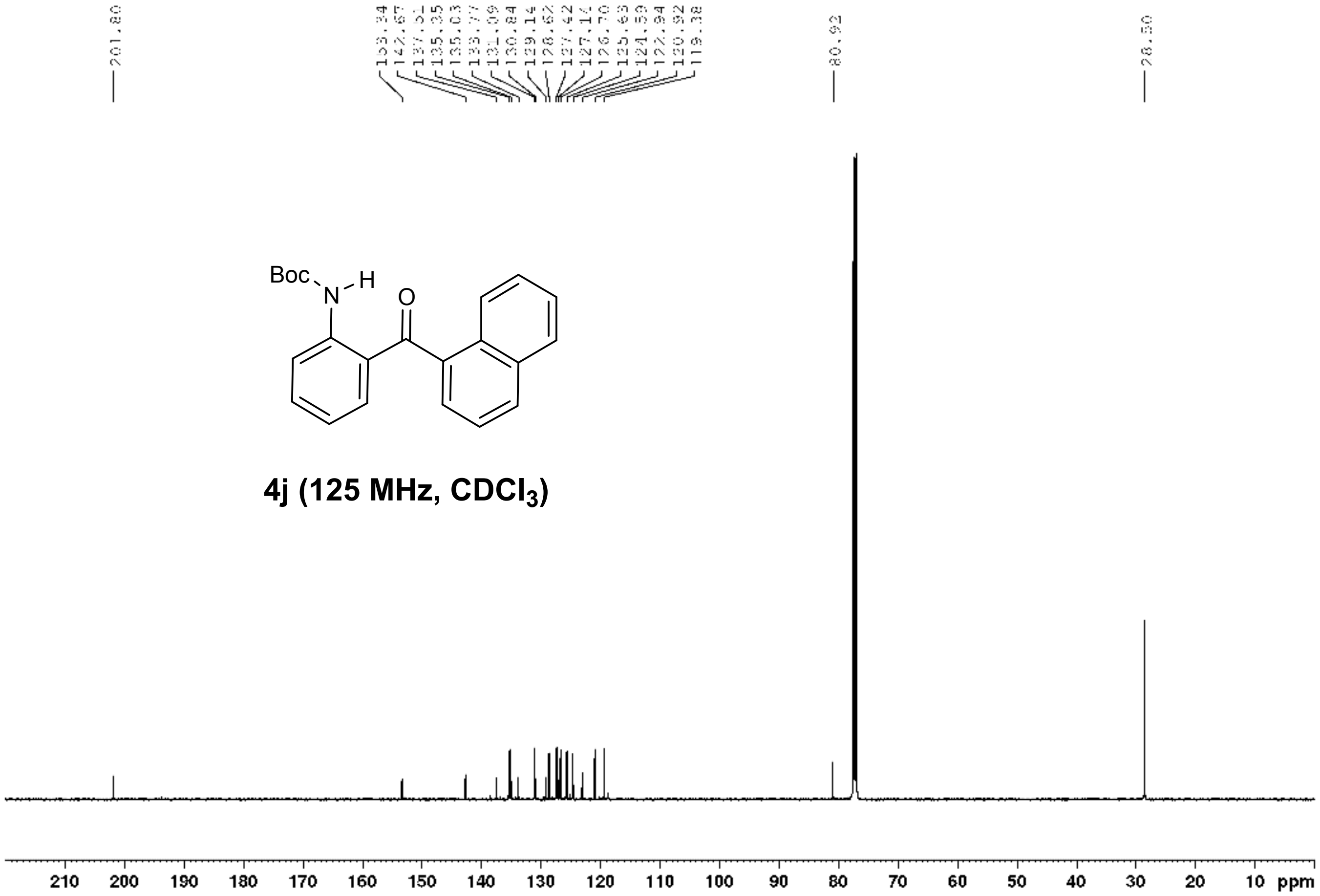

4j (125 MHz, $CDCl_3$)

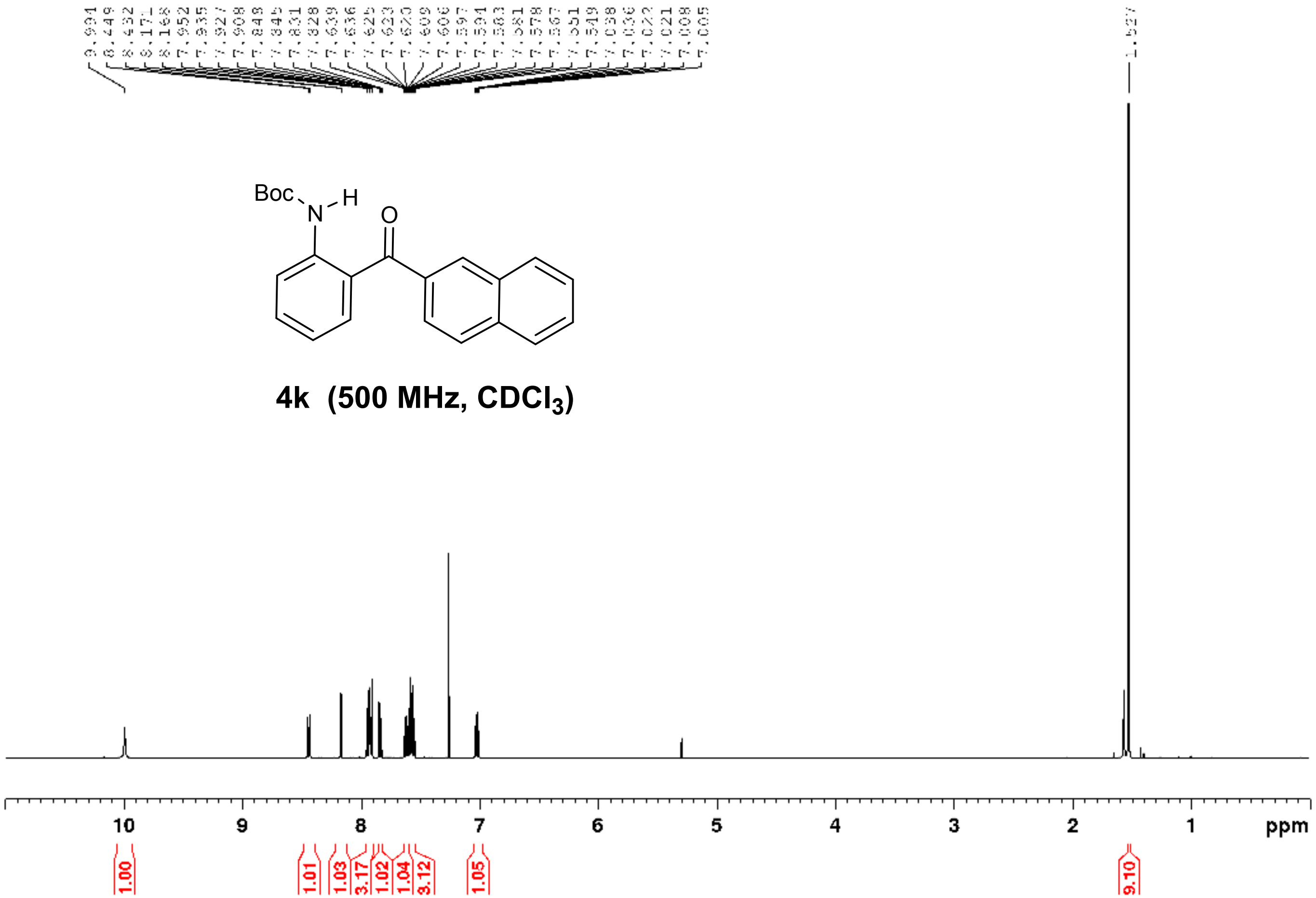

4k (500 MHz, $CDCl_3$)

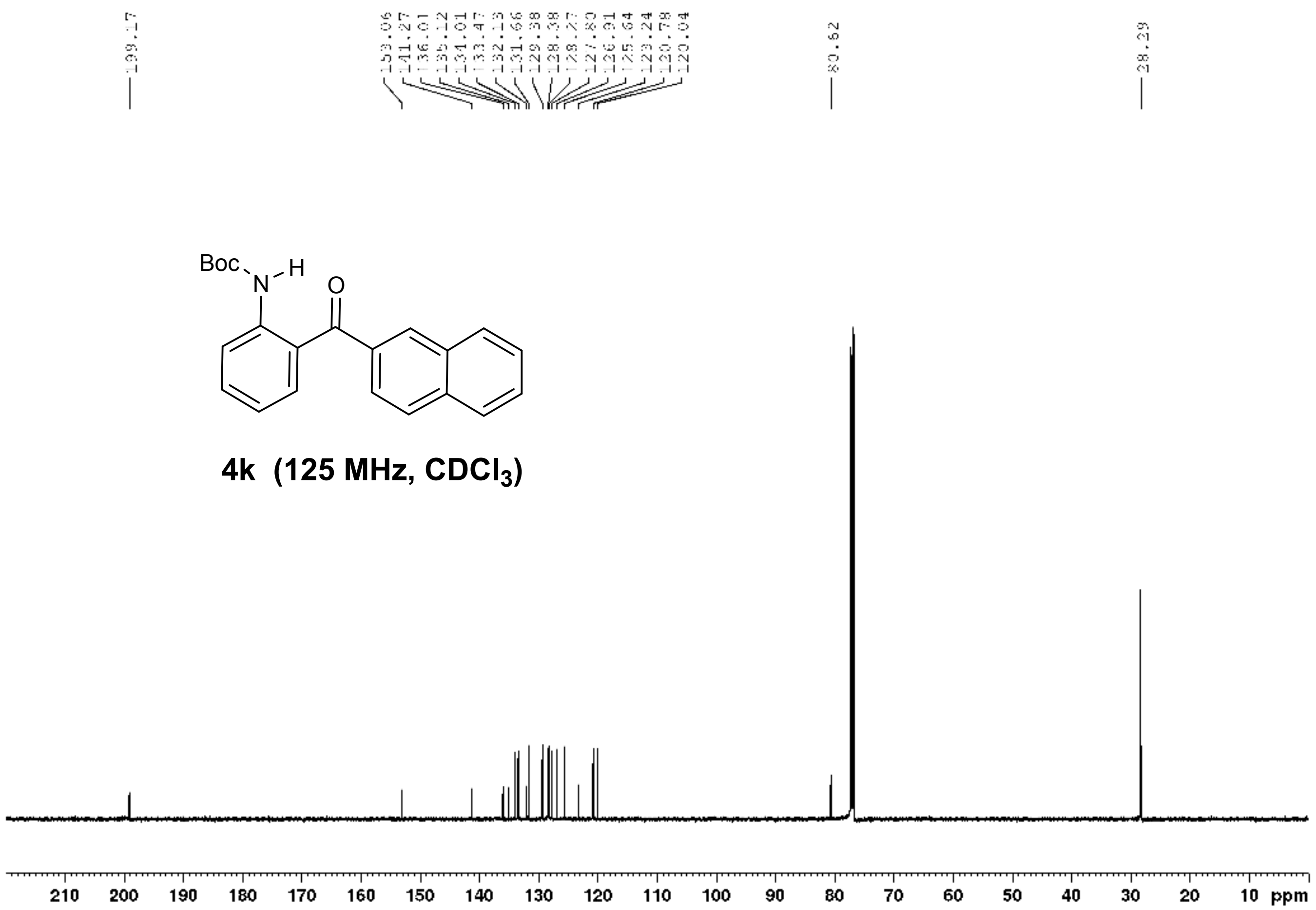
199.17
153.06
141.27
136.01
135.12
134.01
133.47
132.13
131.66
129.38
128.38
128.27
127.80
126.91
125.64
123.24
120.78
120.04
80.62
28.29
Boc
N
H
O
4k (125 MHz, CDCl3)
210 200 190 180 170 160 150 140 130 120 110 100 90 80 70 60 50 40 30 20 10 ppm

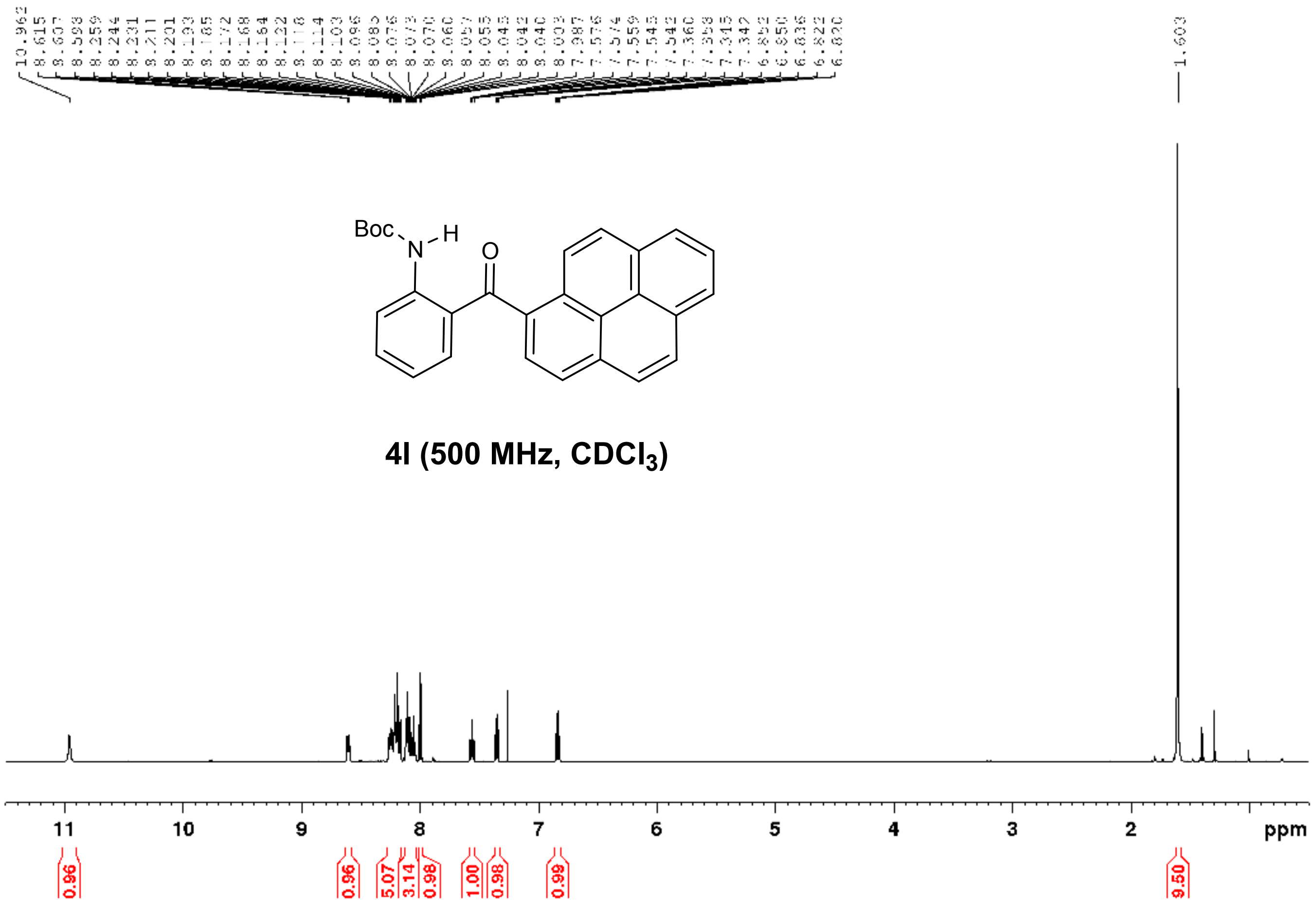
10.962
8.615
8.607
8.598
8.259
8.244
8.231
8.211
8.201
8.193
8.185
8.172
8.168
8.164
8.122
8.118
8.114
8.103
8.096
8.085
8.076
8.073
8.070
8.060
8.057
8.055
8.043
8.042
8.040
8.003
7.987
7.576
7.574
7.559
7.545
7.542
7.360
7.358
7.345
7.342
6.852
6.850
6.836
6.822
6.820
1.603
Boc
N
H
O
4l (500 MHz, CDCl3)
11
10
9
8
7
6
5
4
3
2
ppm
0.96
0.96
5.07
3.14
0.98
1.00
0.98
0.99
9.50

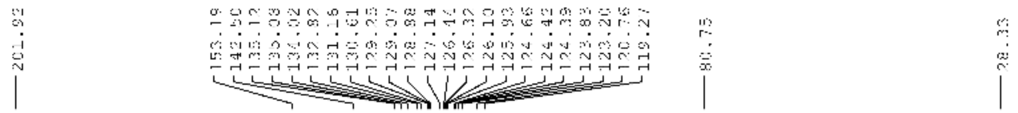

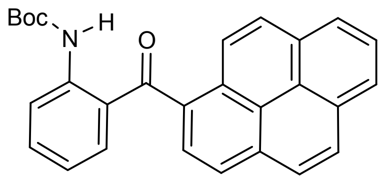

4l (125 MHz, CDCl$_3$)

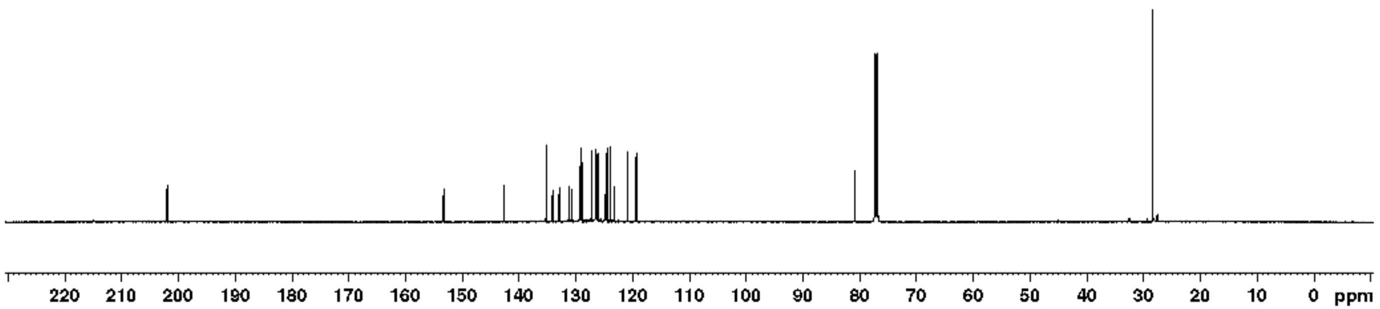

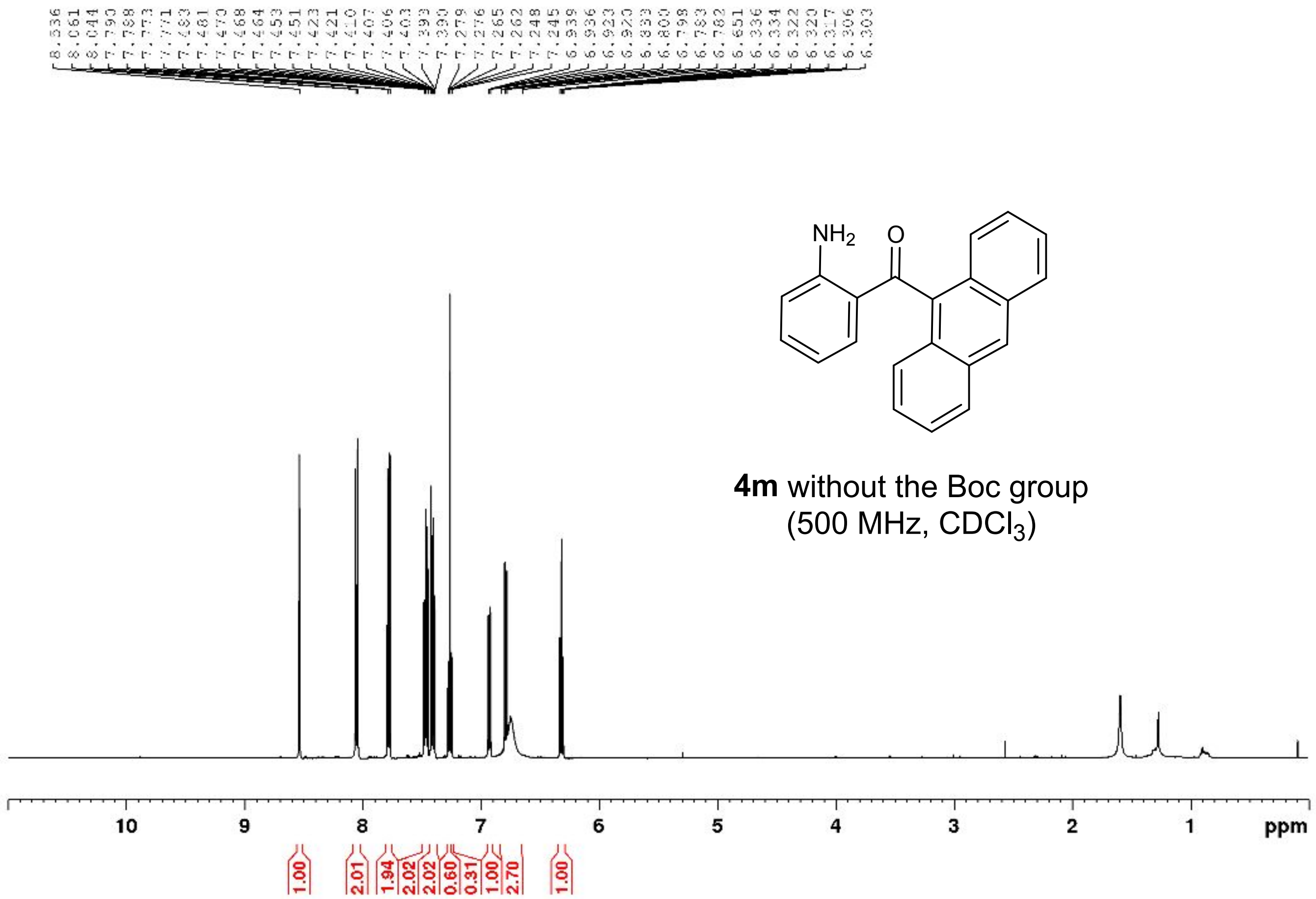

4m without the Boc group
(500 MHz, $CDCl_3$)

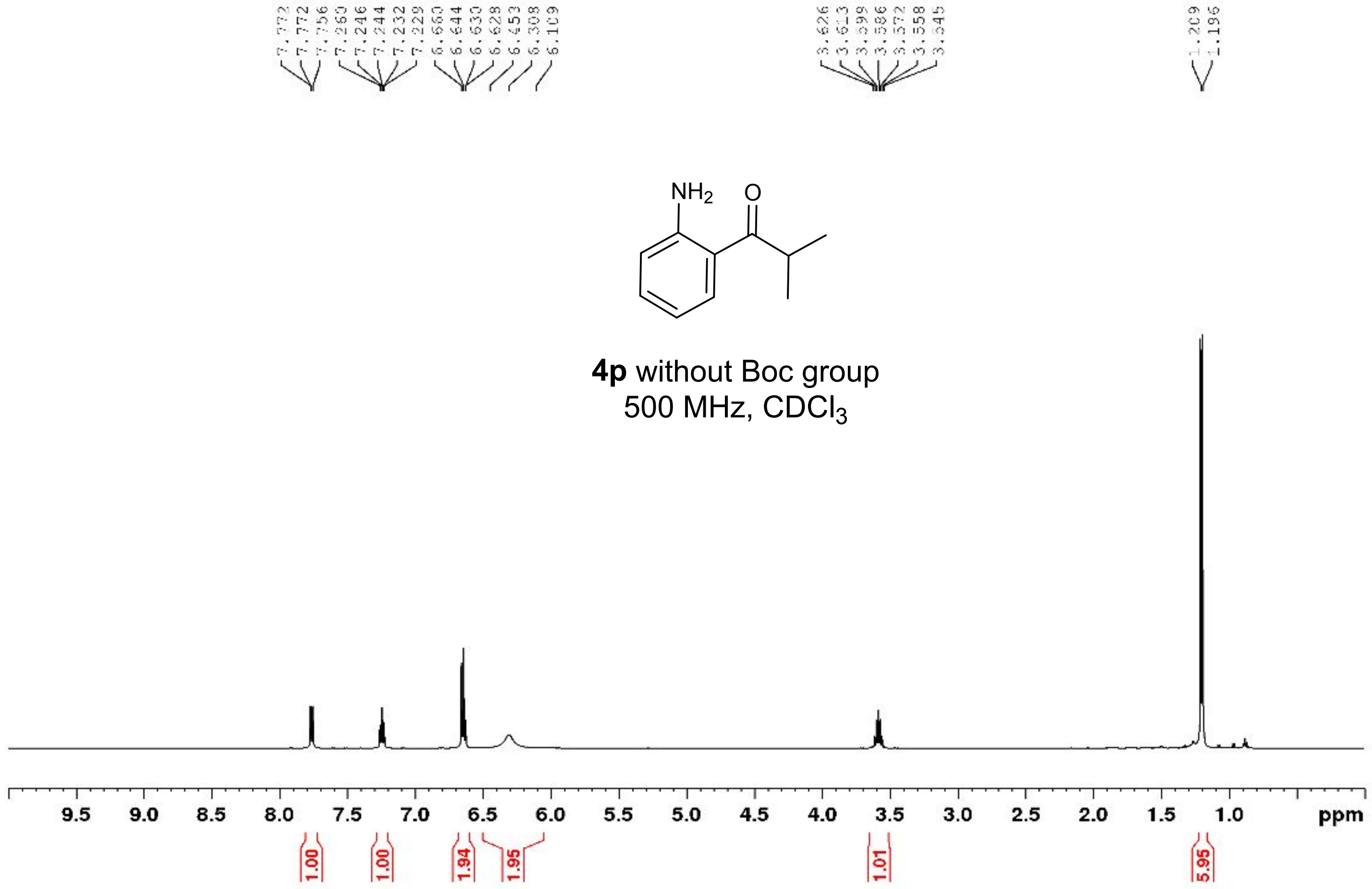

NH2
O
4p without Boc group
500 MHz, CDCl3
ppm

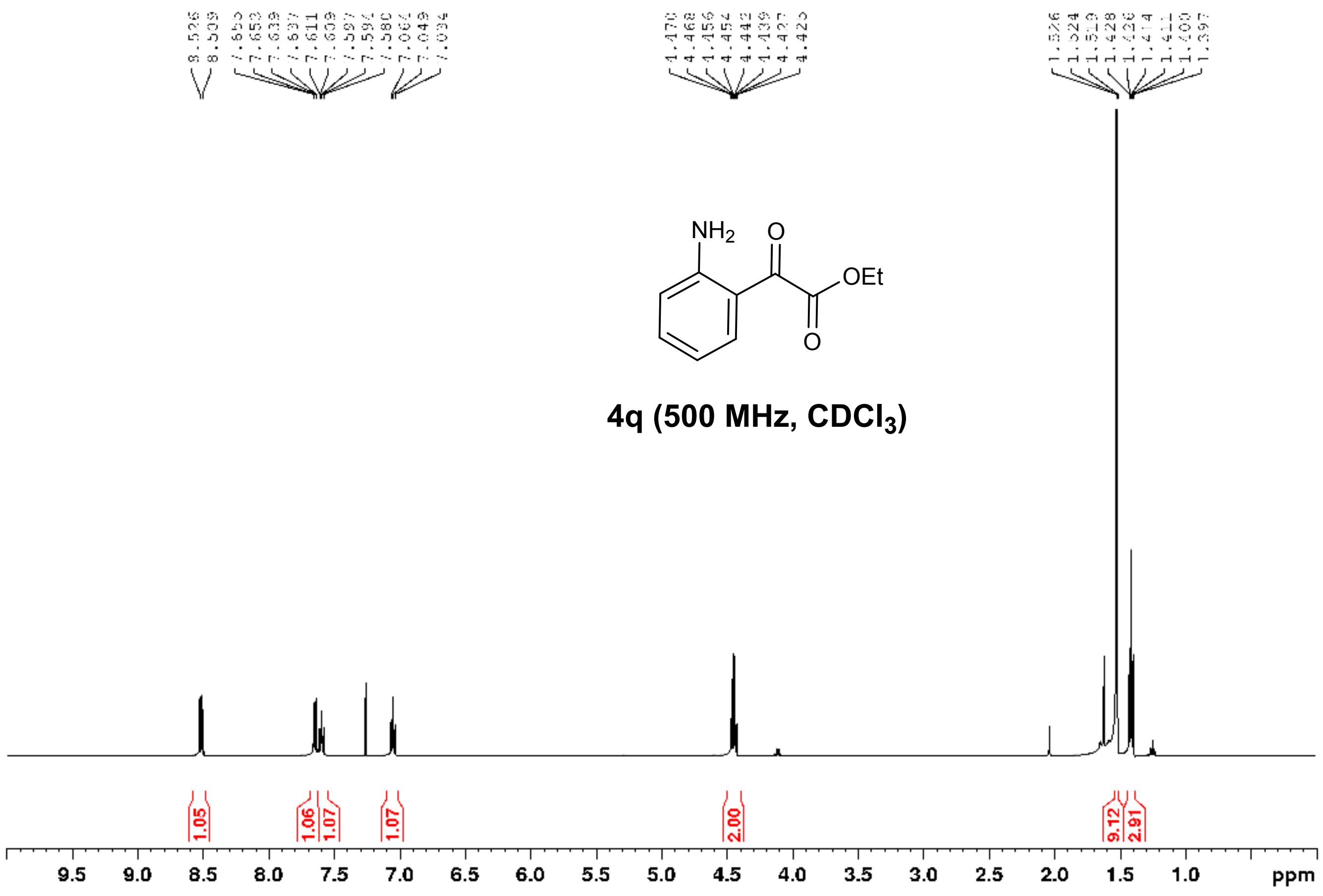

4q (500 MHz, CDCl$_3$)

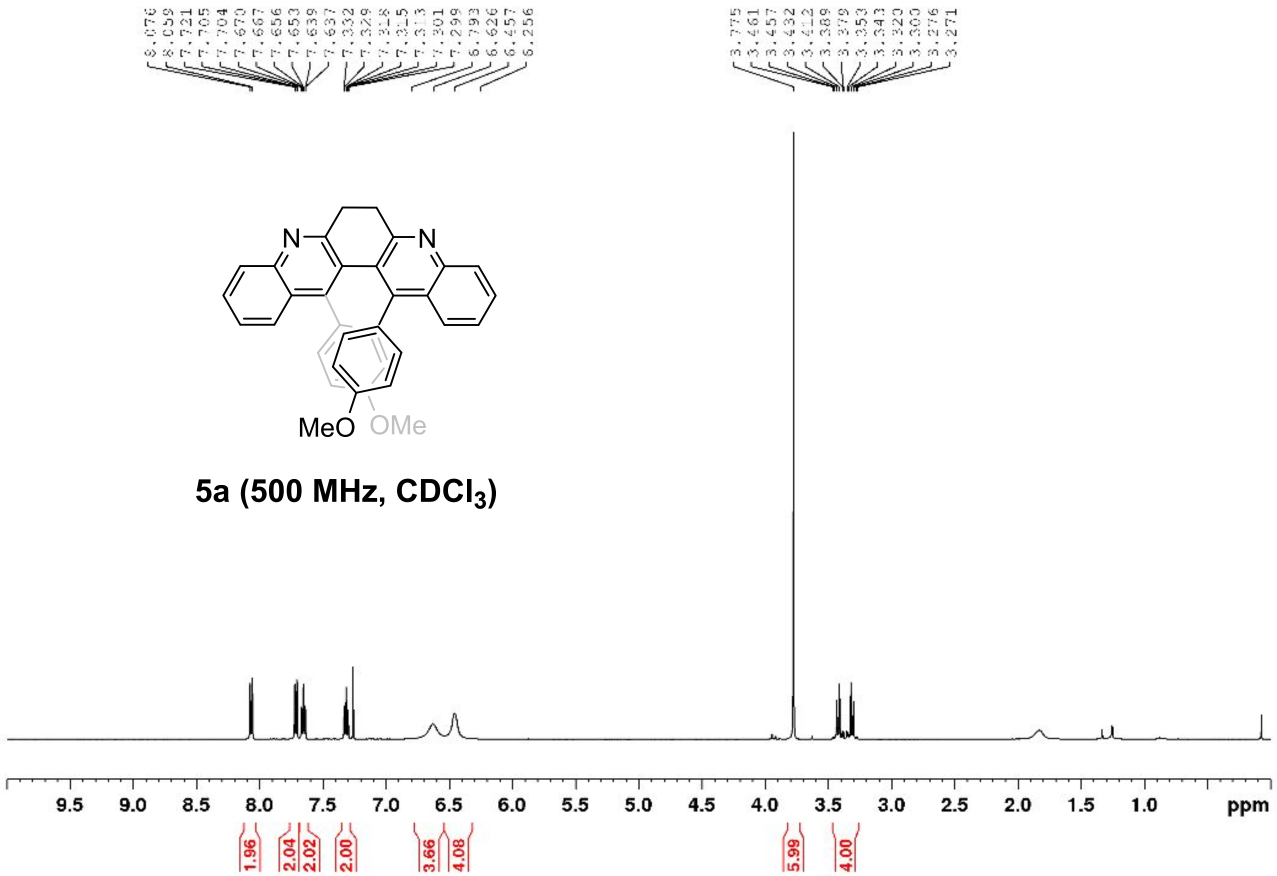

5a (500 MHz, $CDCl_3$)

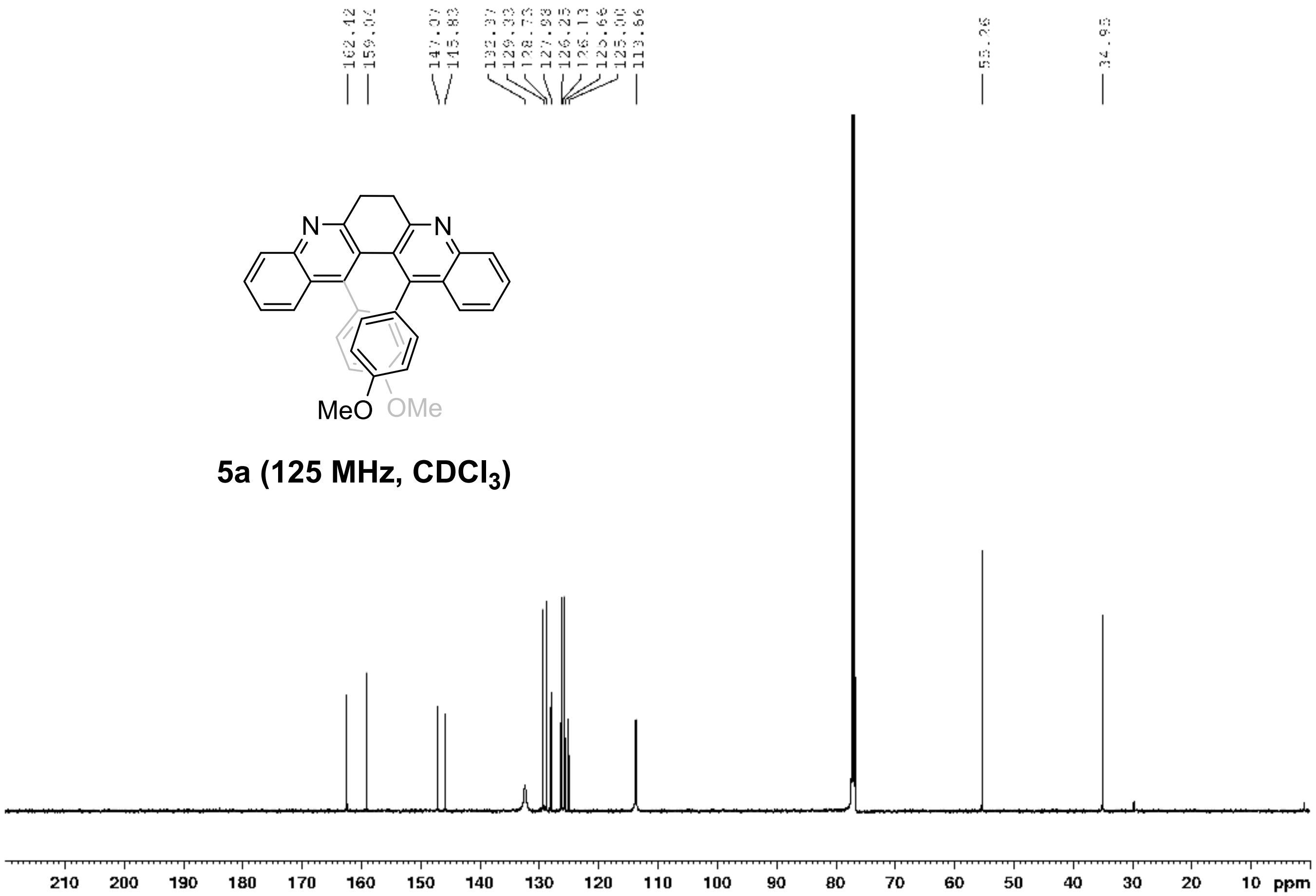

5a (125 MHz, $CDCl_3$)

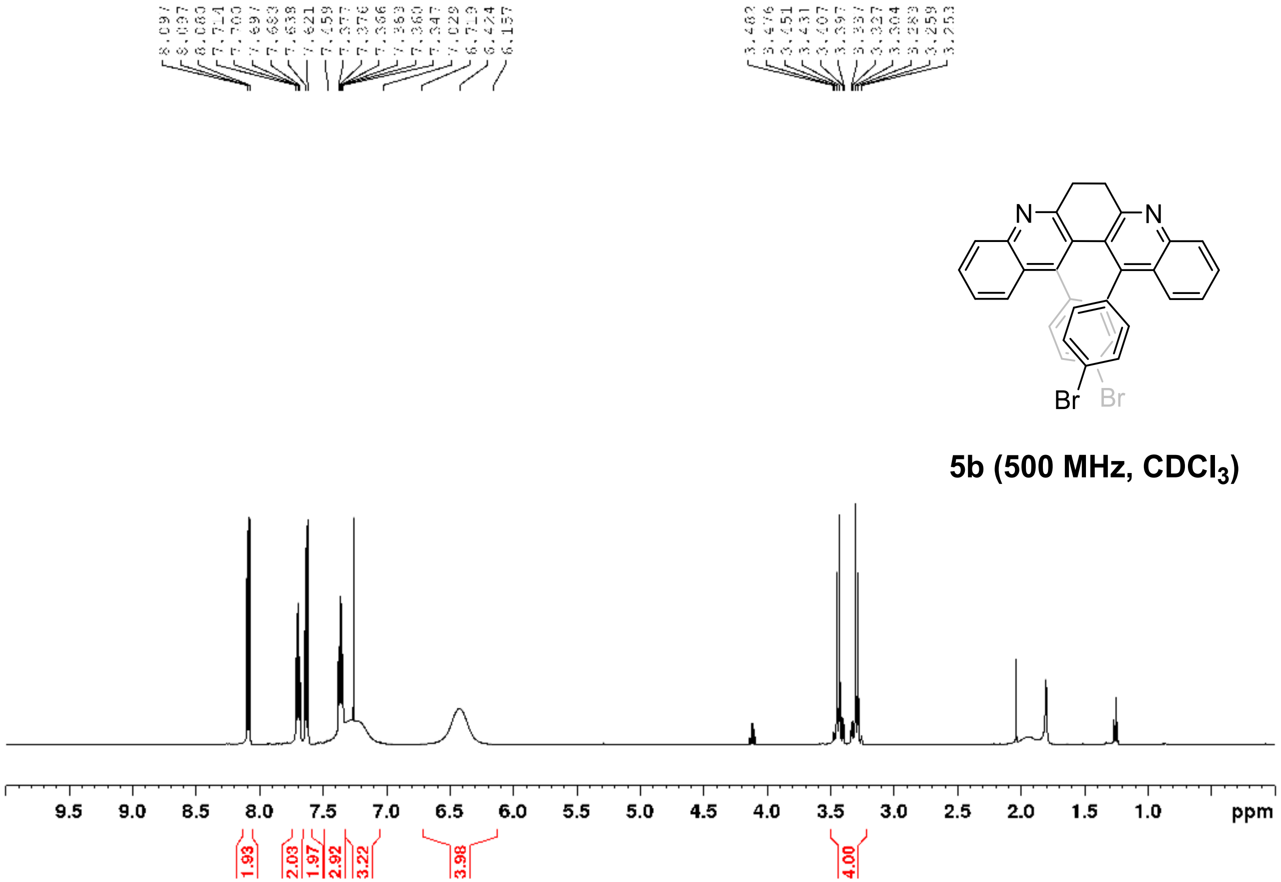

N
N
Br
Br
5b (500 MHz, CDCl$_3$)
9.5
9.0
8.5
8.0
7.5
7.0
6.5
6.0
5.5
5.0
4.5
4.0
3.5
3.0
2.5
2.0
1.5
1.0
ppm
1.93
2.03
1.97
2.92
3.22
3.98
4.00

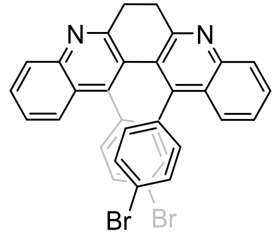

5b (125 MHz, $CDCl_3$)

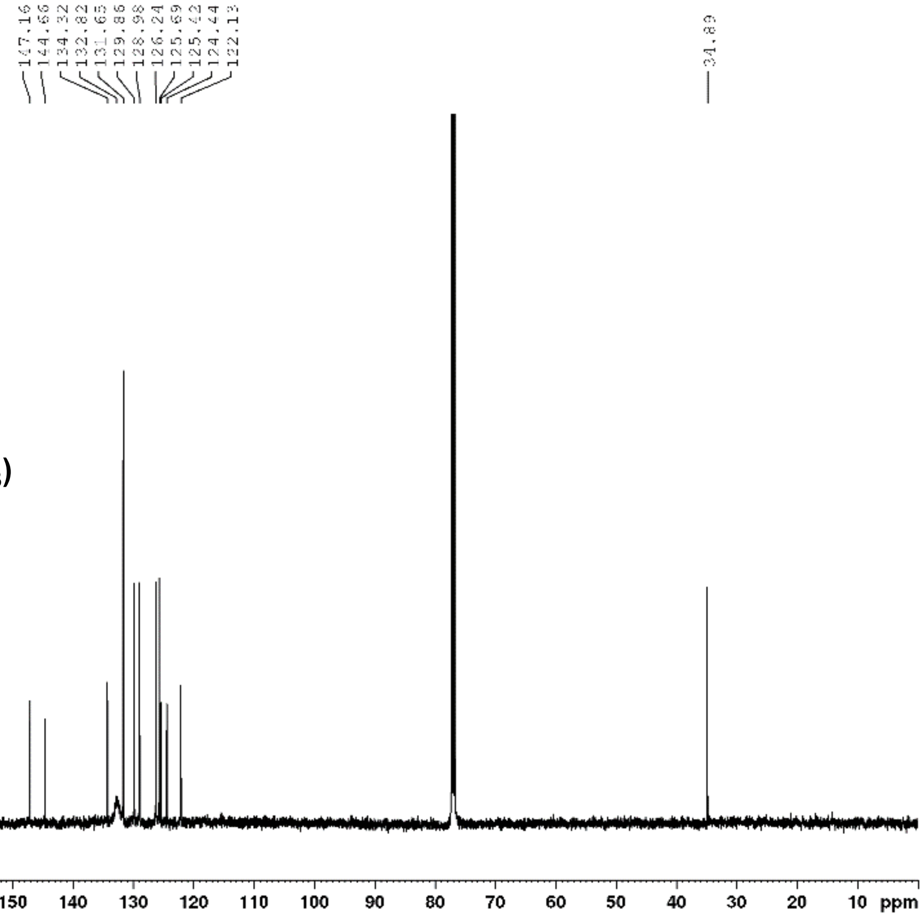

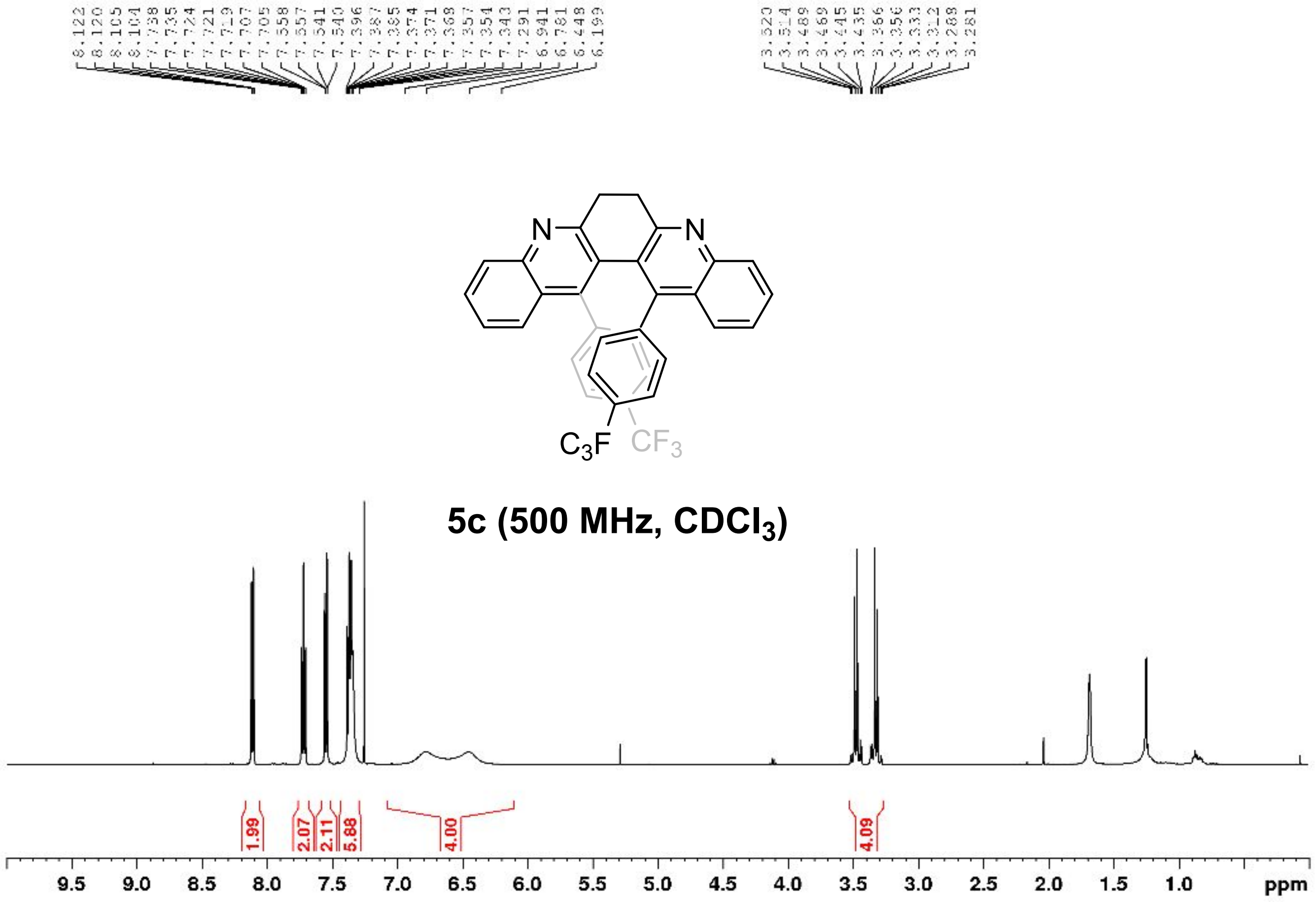

5c (500 MHz, CDCl$_3$)

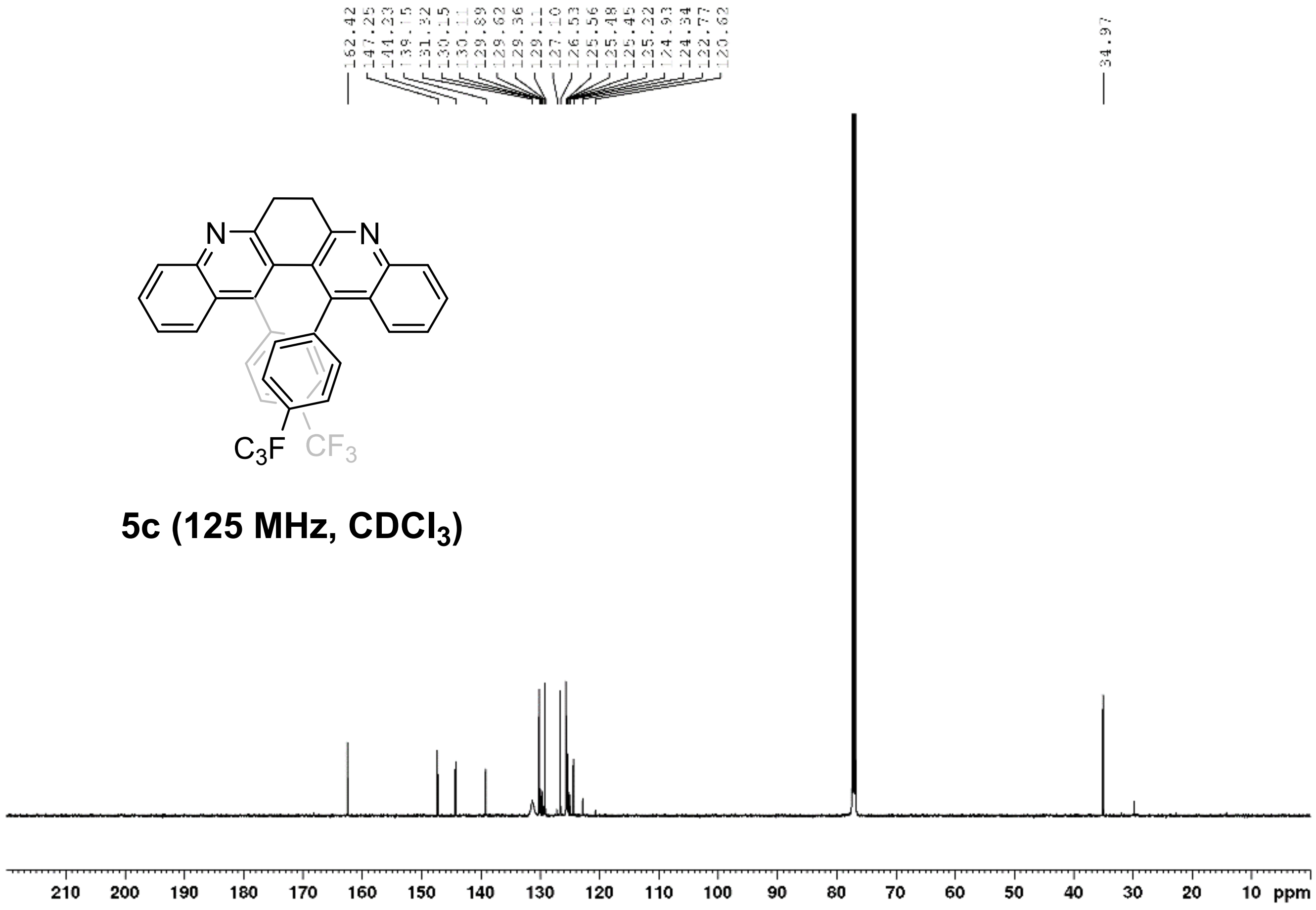

5c (125 MHz, CDCl$_3$)

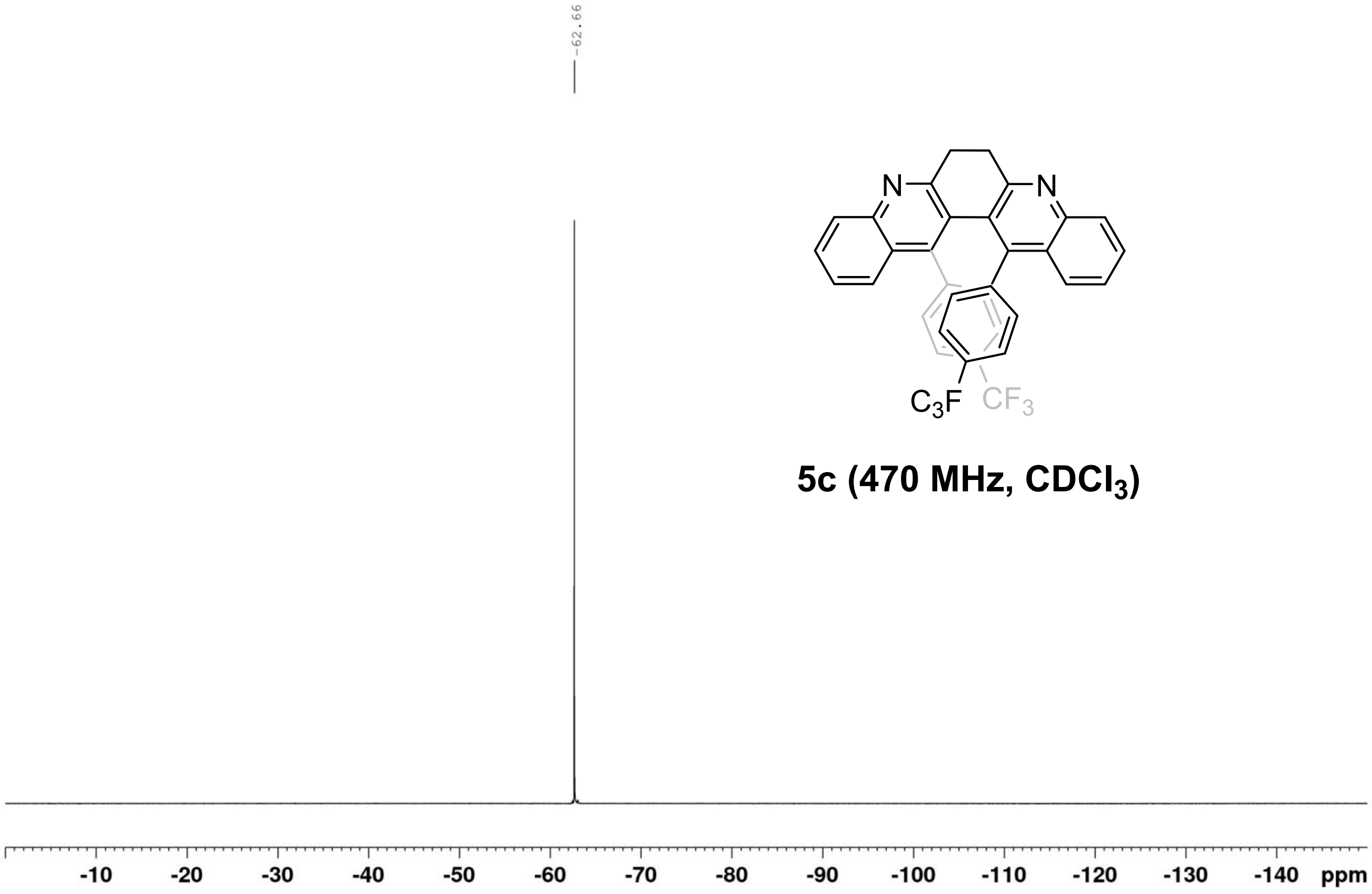

5c (470 MHz, $CDCl_3$)

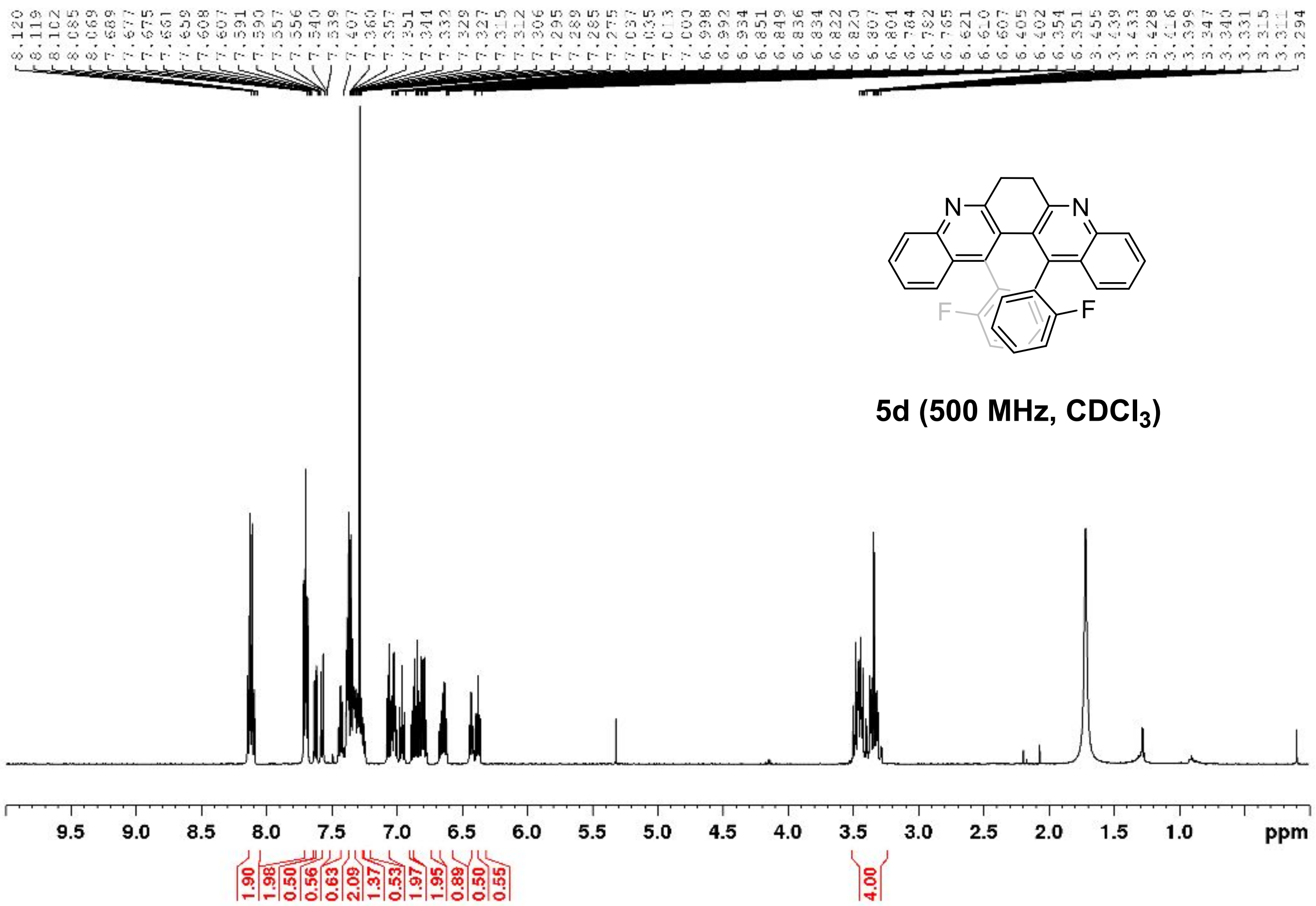

5d (500 MHz, $CDCl_3$)

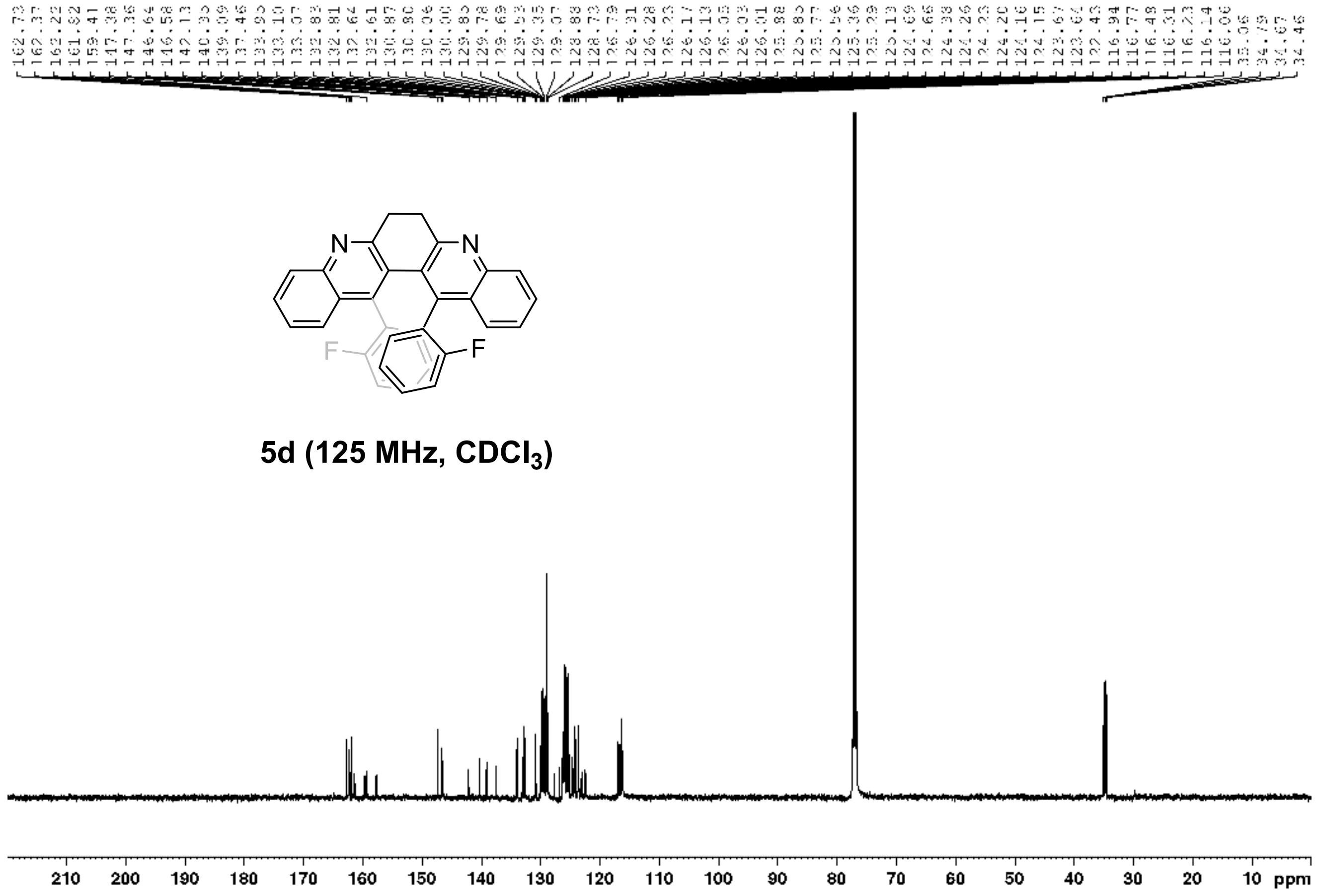
5d (125 MHz, CDCl3)
210 200 190 180 170 160 150 140 130 120 110 100 90 80 70 60 50 40 30 20 10 ppm

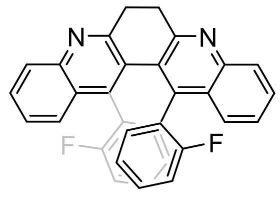

5d (470 MHz, $CDCl_3$)

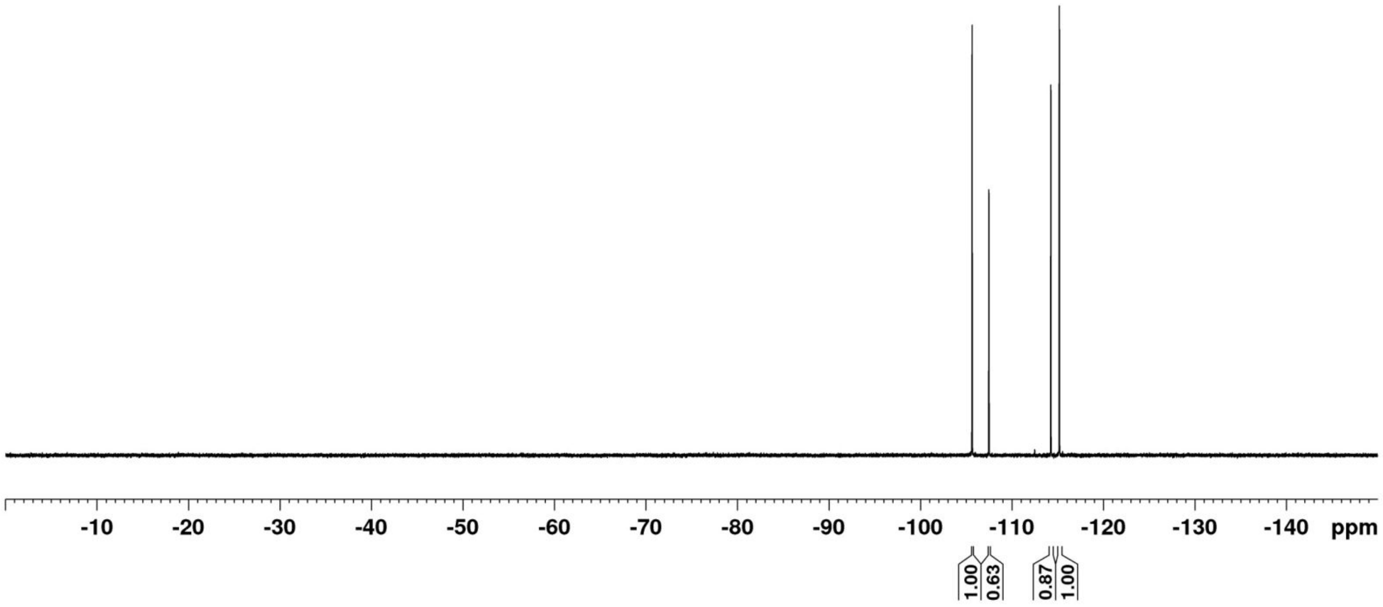

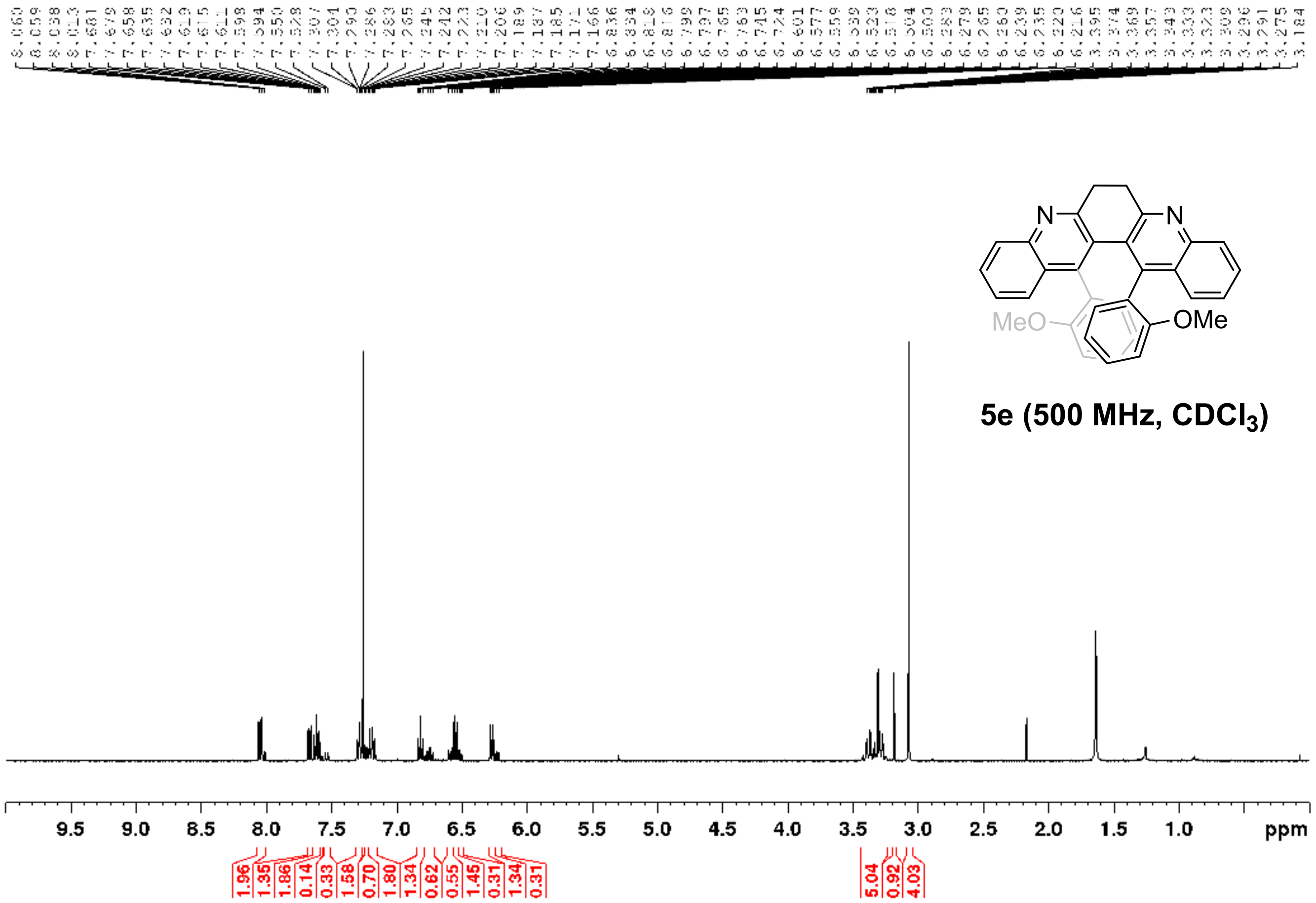

5e (500 MHz, $CDCl_3$)

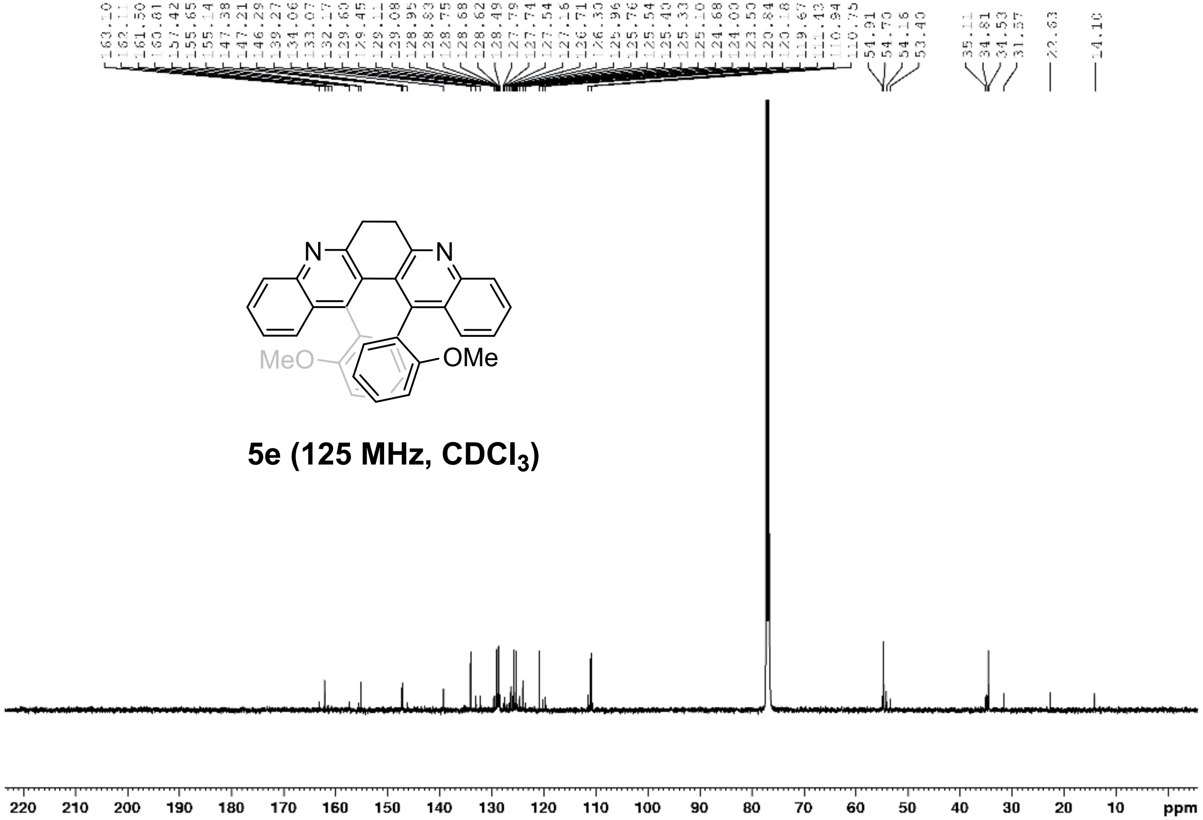

5e (125 MHz, $CDCl_3$)

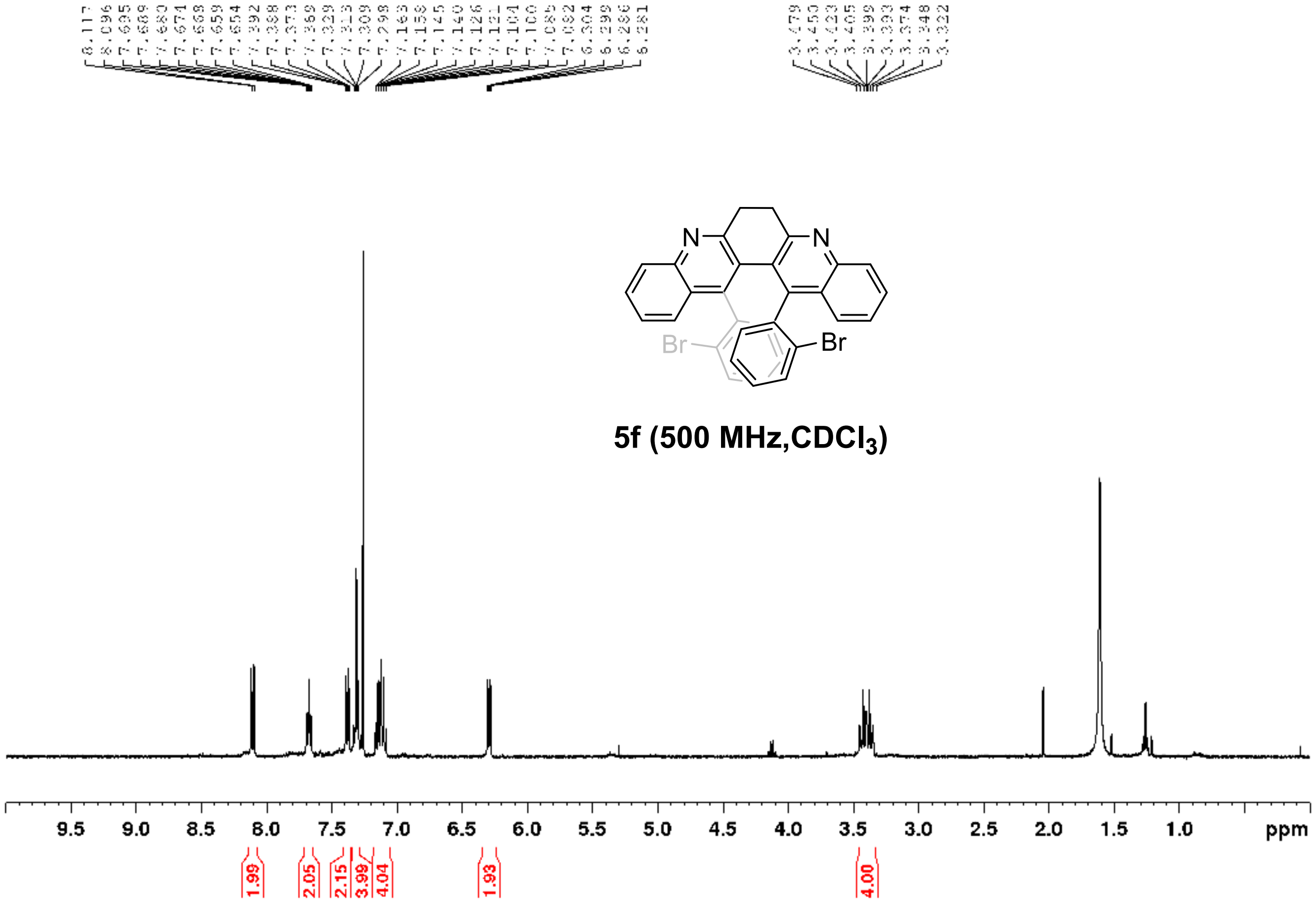
8.117
8.096
7.695
7.689
7.680
7.671
7.668
7.659
7.654
7.392
7.388
7.373
7.369
7.329
7.313
7.309
7.298
7.163
7.158
7.145
7.140
7.126
7.121
7.104
7.100
7.085
7.082
6.304
6.299
6.286
6.281
3.479
3.450
3.423
3.405
3.399
3.393
3.374
3.348
3.322
N
N
Br
Br
5f (500 MHz,CDCl$_3$)
9.5 9.0 8.5 8.0 7.5 7.0 6.5 6.0 5.5 5.0 4.5 4.0 3.5 3.0 2.5 2.0 1.5 1.0 ppm
1.99
2.05
2.15
3.99
4.04
1.93
4.00

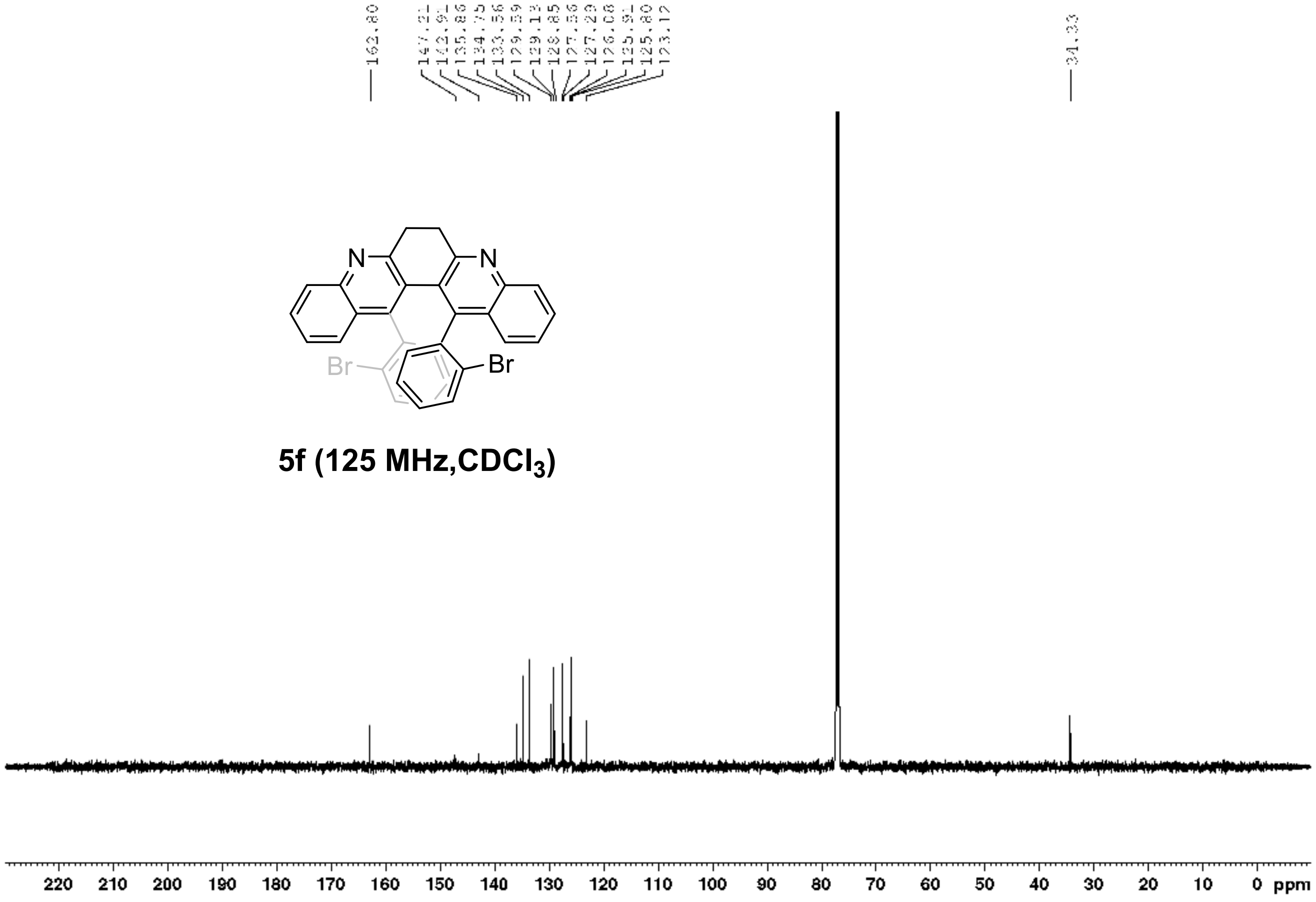

5f (125 MHz,$CDCl_3$)

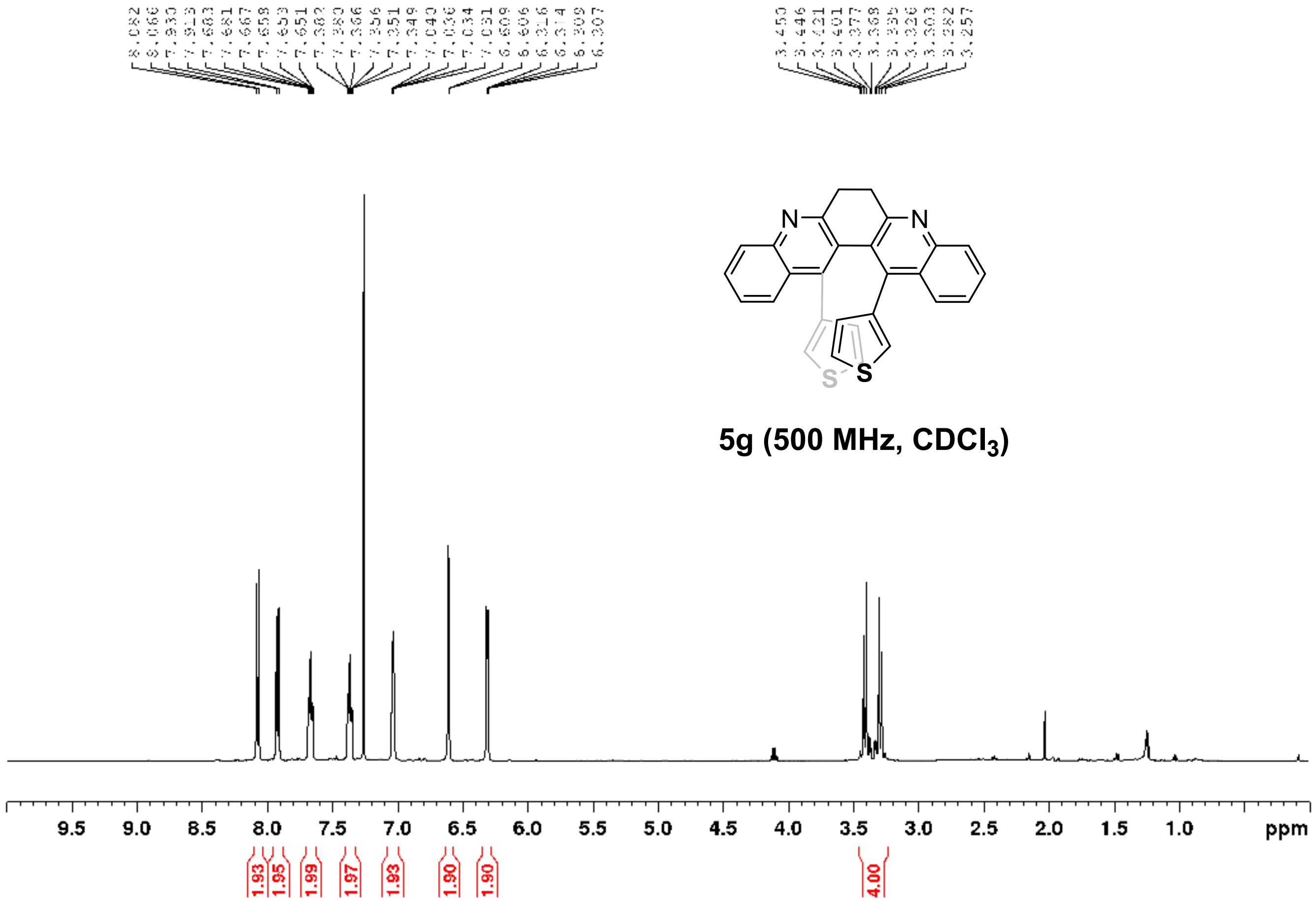

5g (500 MHz, CDCl3)
ppm

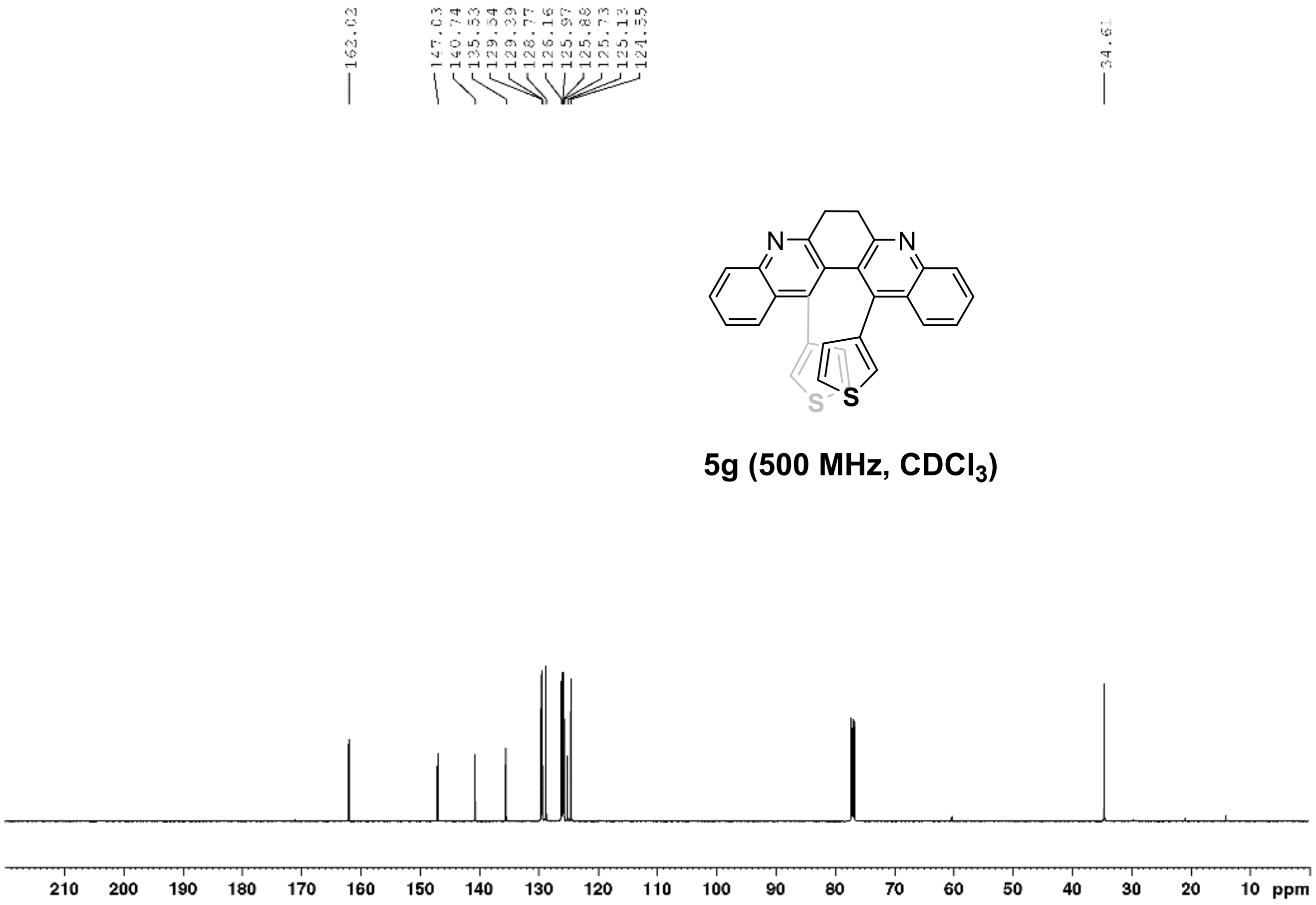

5g (500 MHz, $CDCl_3$)

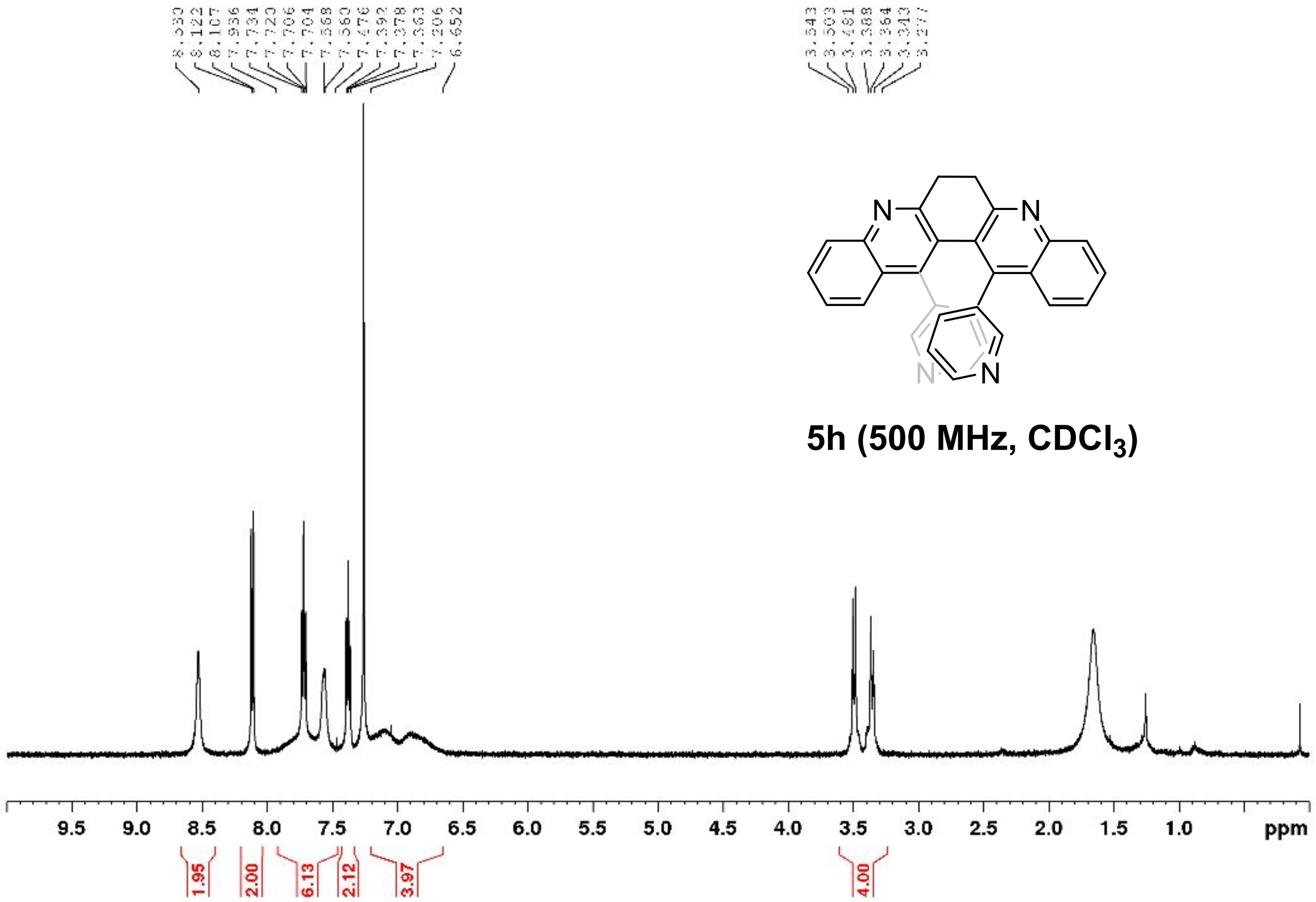

5h (500 MHz, $CDCl_3$)

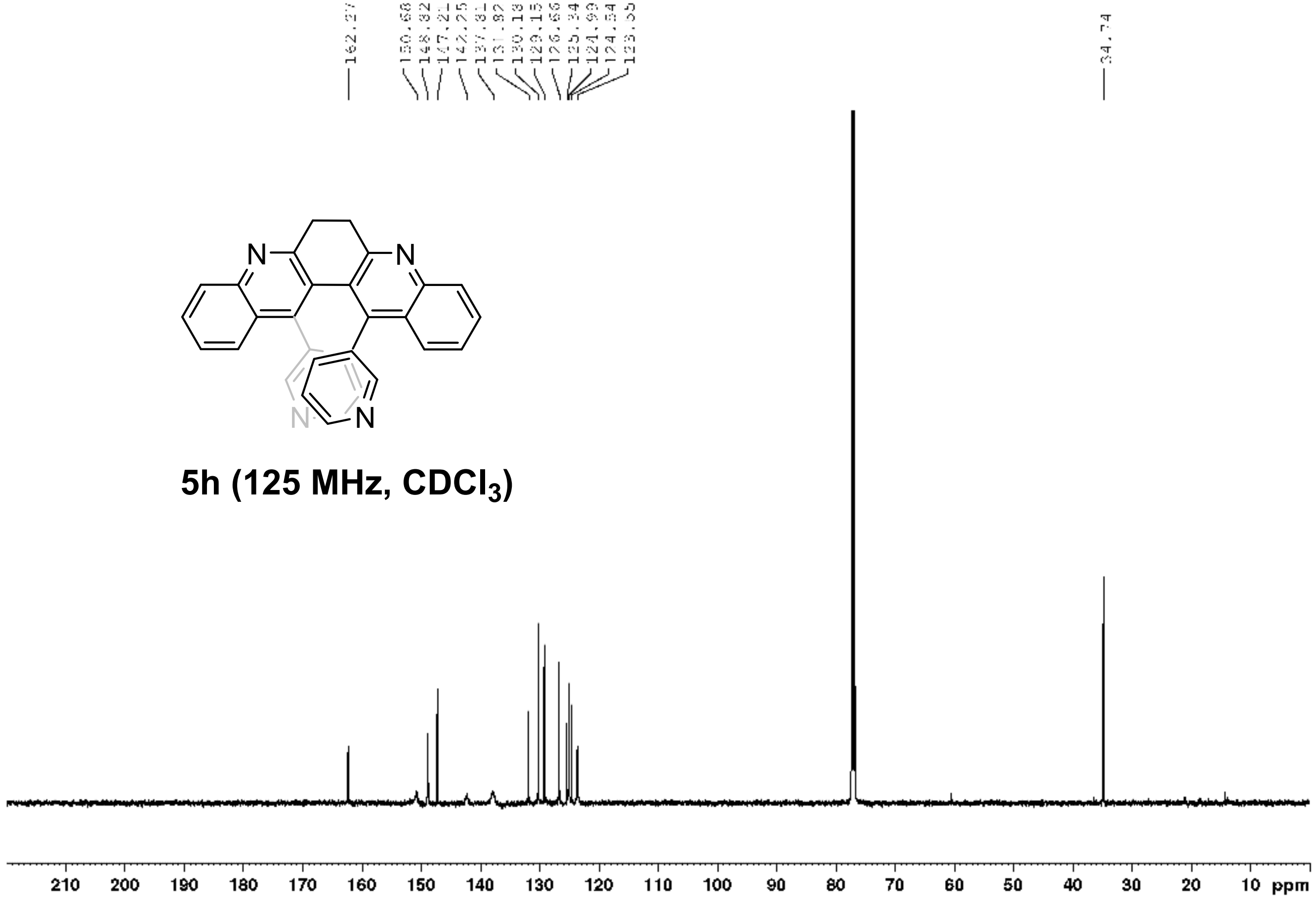

5h (125 MHz, $CDCl_3$)

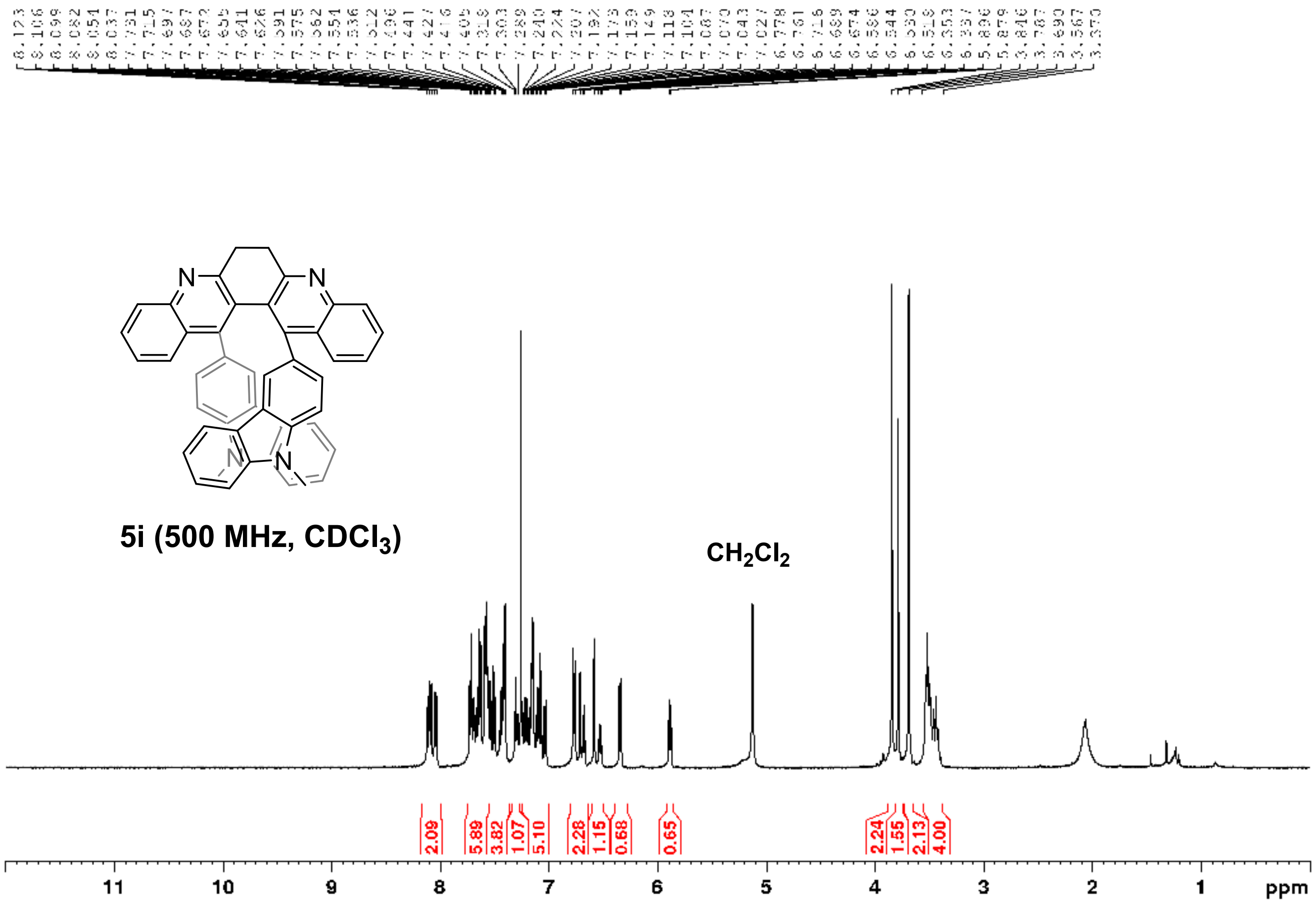
5i (500 MHz, CDCl$_3$)
CH$_2$Cl$_2$
2.09
5.89
3.82
1.07
5.10
2.28
1.15
0.68
0.65
2.24
1.55
2.13
4.00
ppm

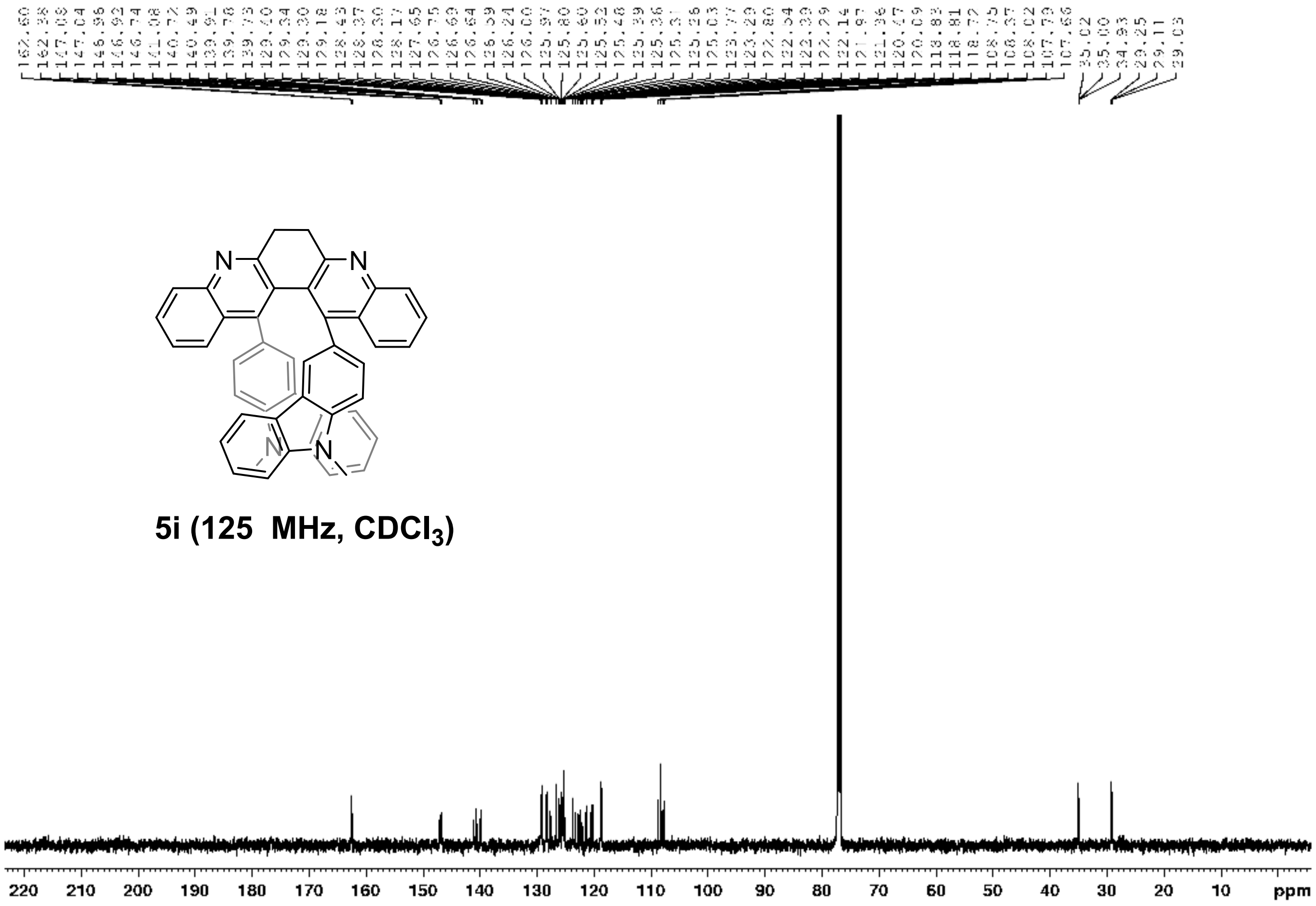

5i (125 MHz, $CDCl_3$)

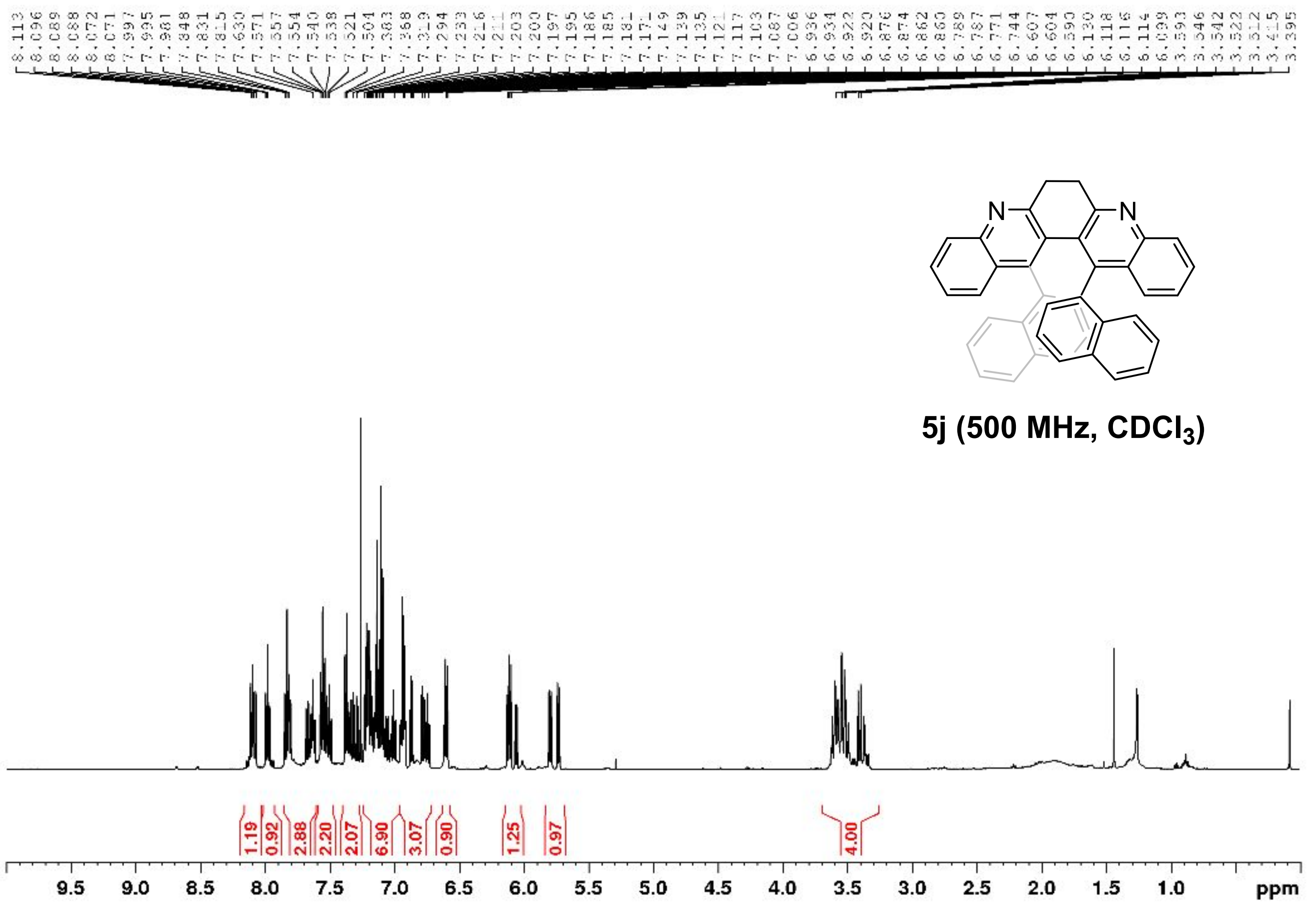

5j (500 MHz, $CDCl_3$)

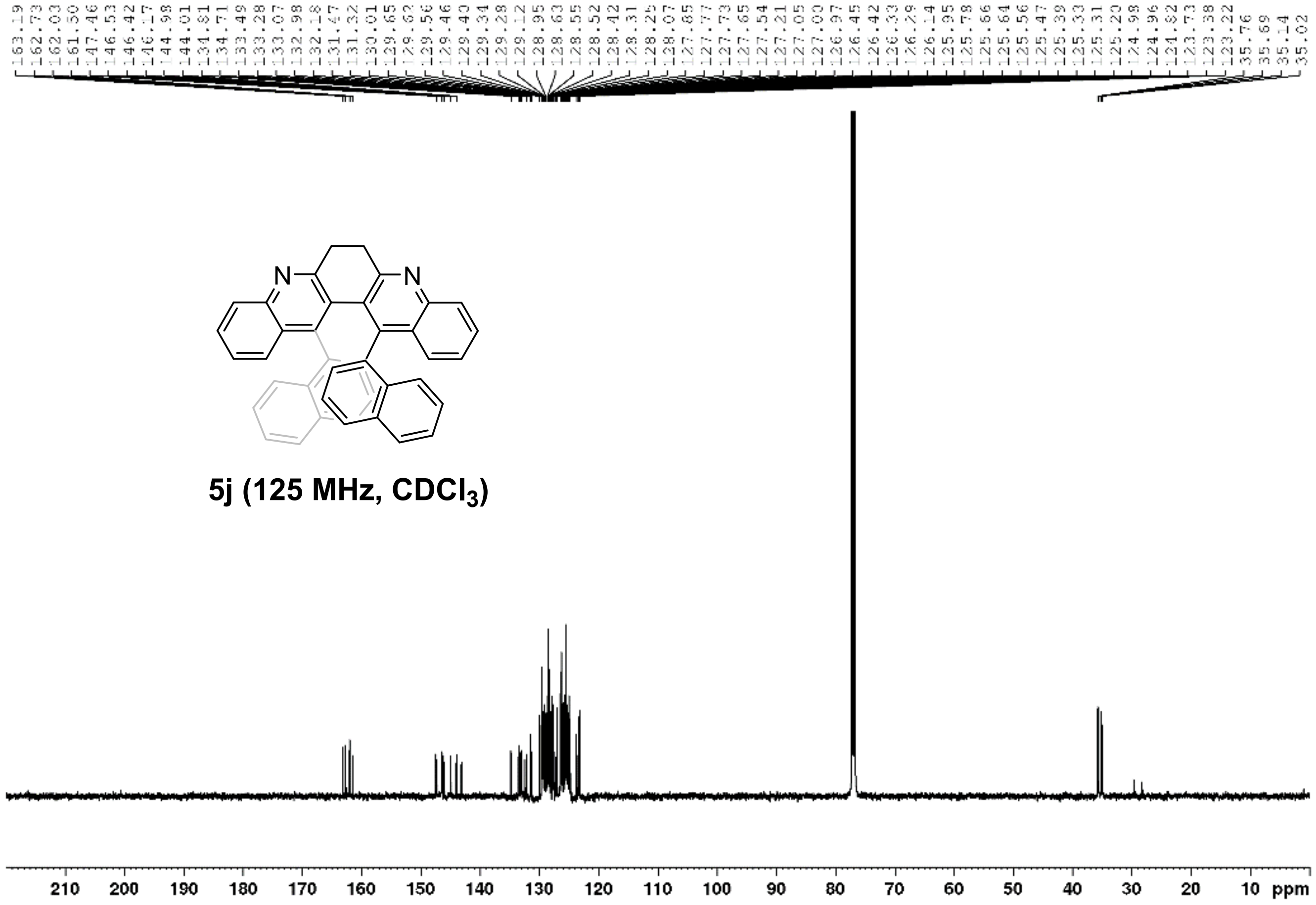

5j (125 MHz, $CDCl_3$)

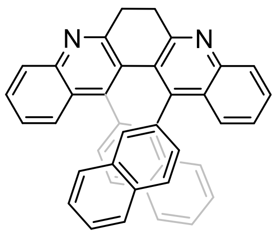

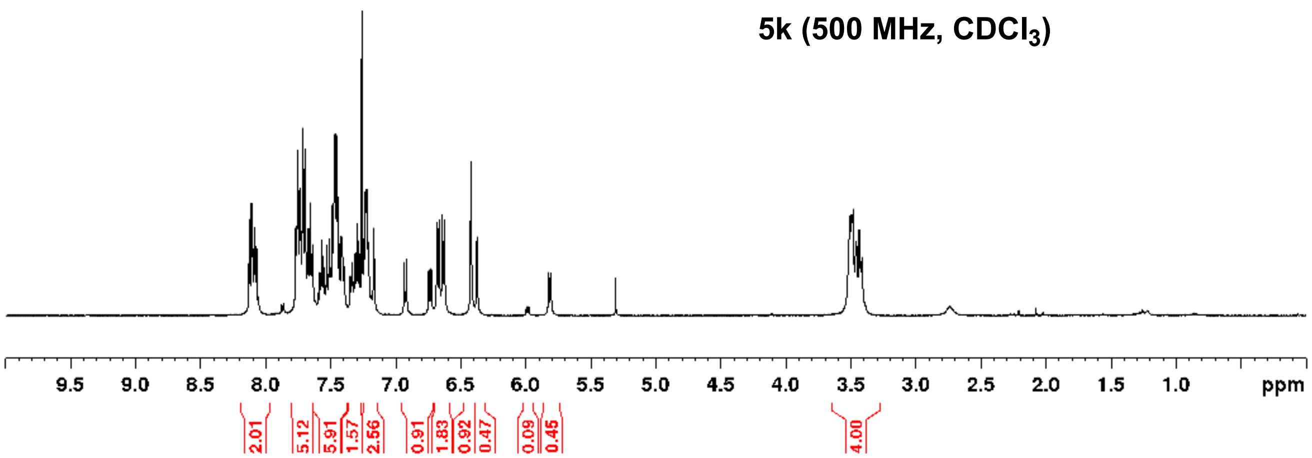

5k (500 MHz, $CDCl_3$)

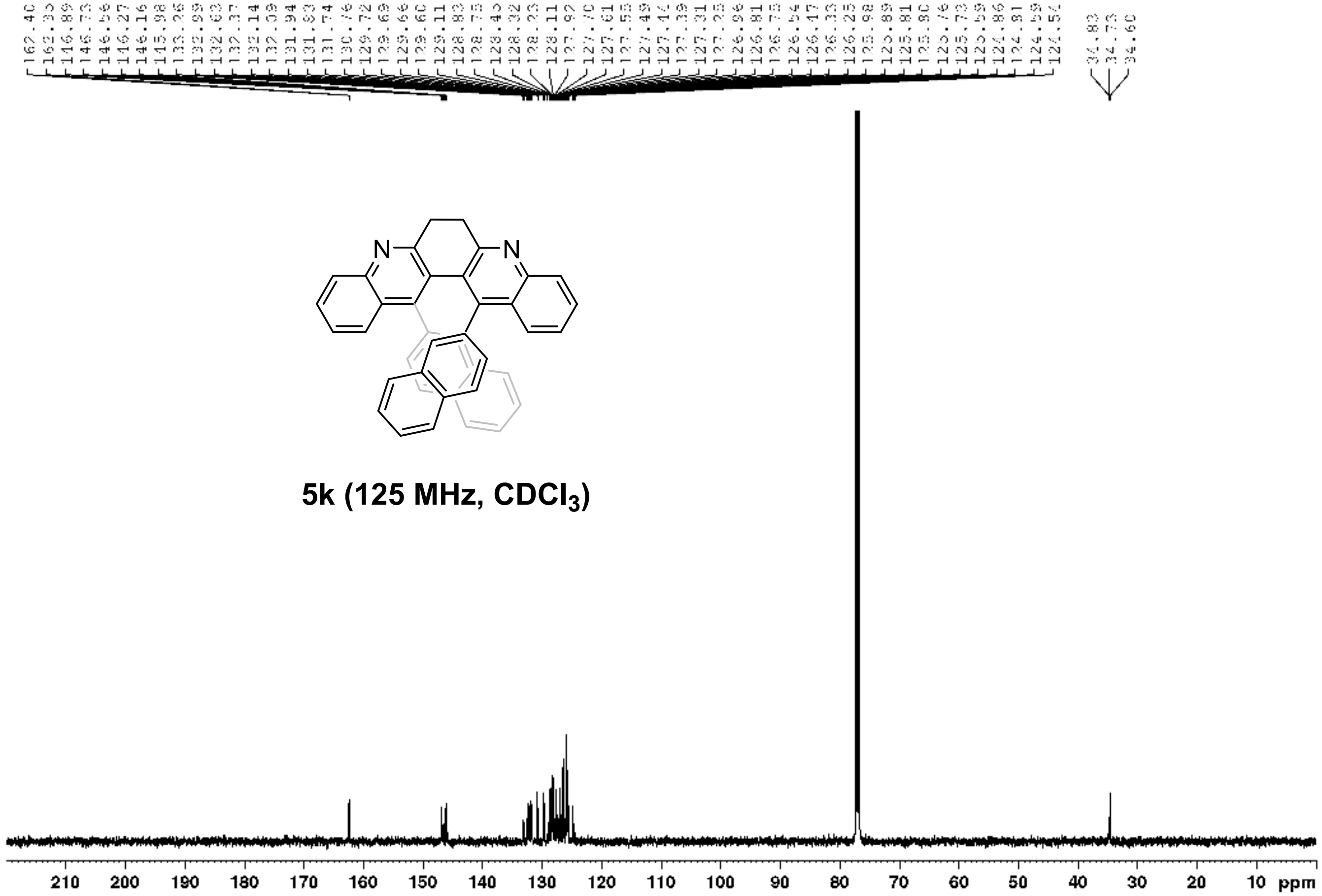

5k (125 MHz, $CDCl_3$)

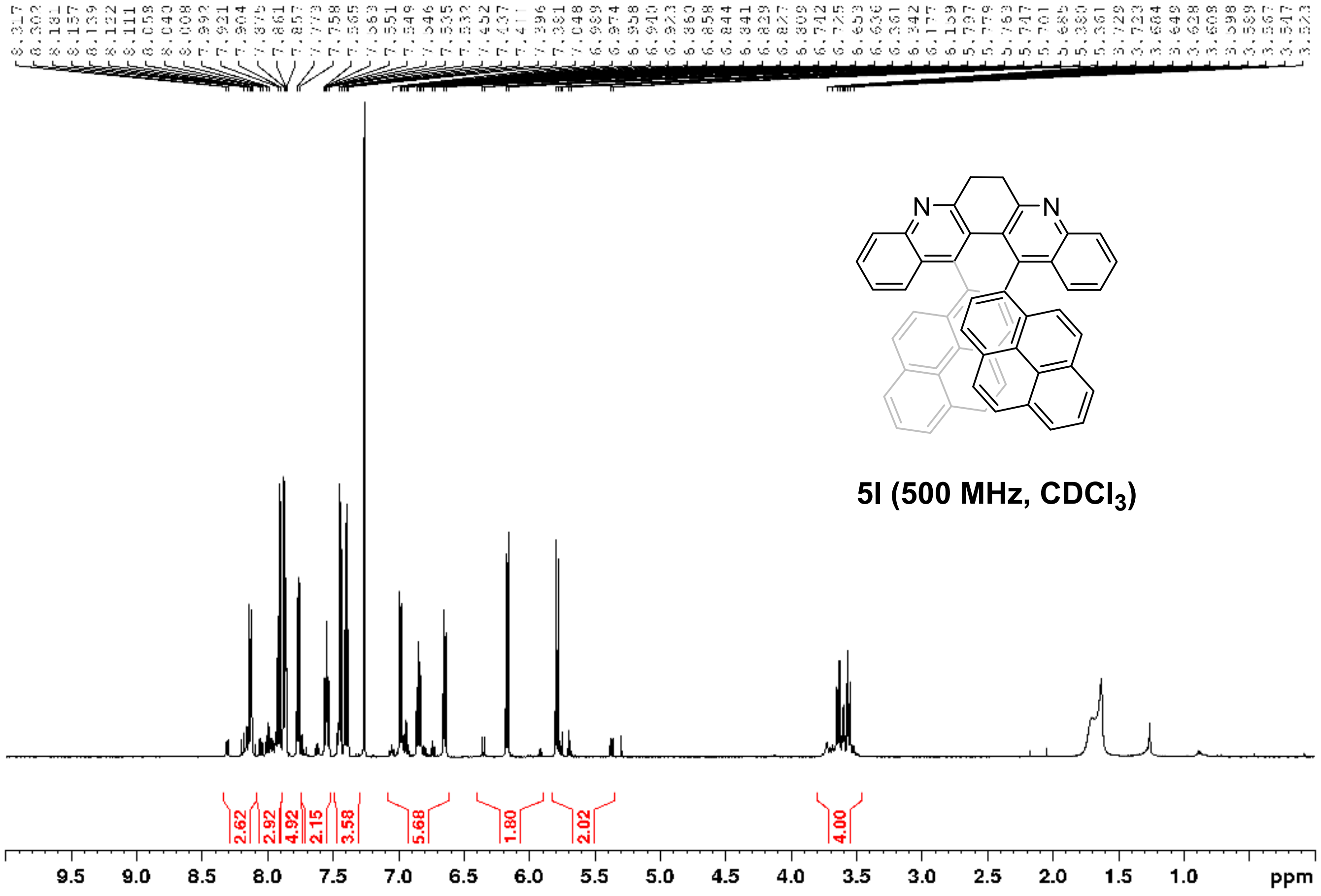
5l (500 MHz, CDCl3)
N
N
2.62
2.92
4.92
2.15
3.58
5.68
1.80
2.02
4.00
9.5 9.0 8.5 8.0 7.5 7.0 6.5 6.0 5.5 5.0 4.5 4.0 3.5 3.0 2.5 2.0 1.5 1.0 ppm

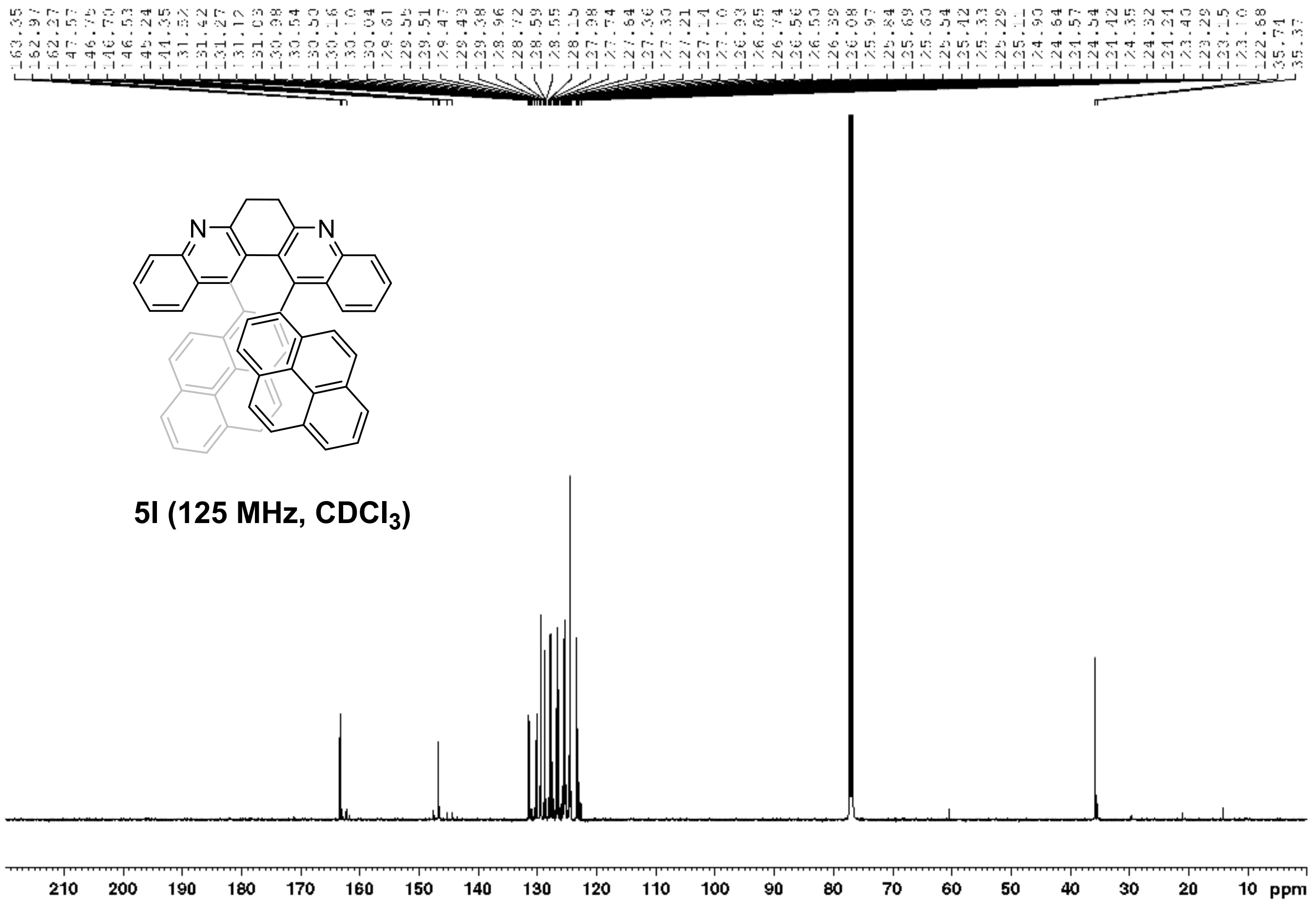
5l (125 MHz, CDCl3)
210 200 190 180 170 160 150 140 130 120 110 100 90 80 70 60 50 40 30 20 10 ppm

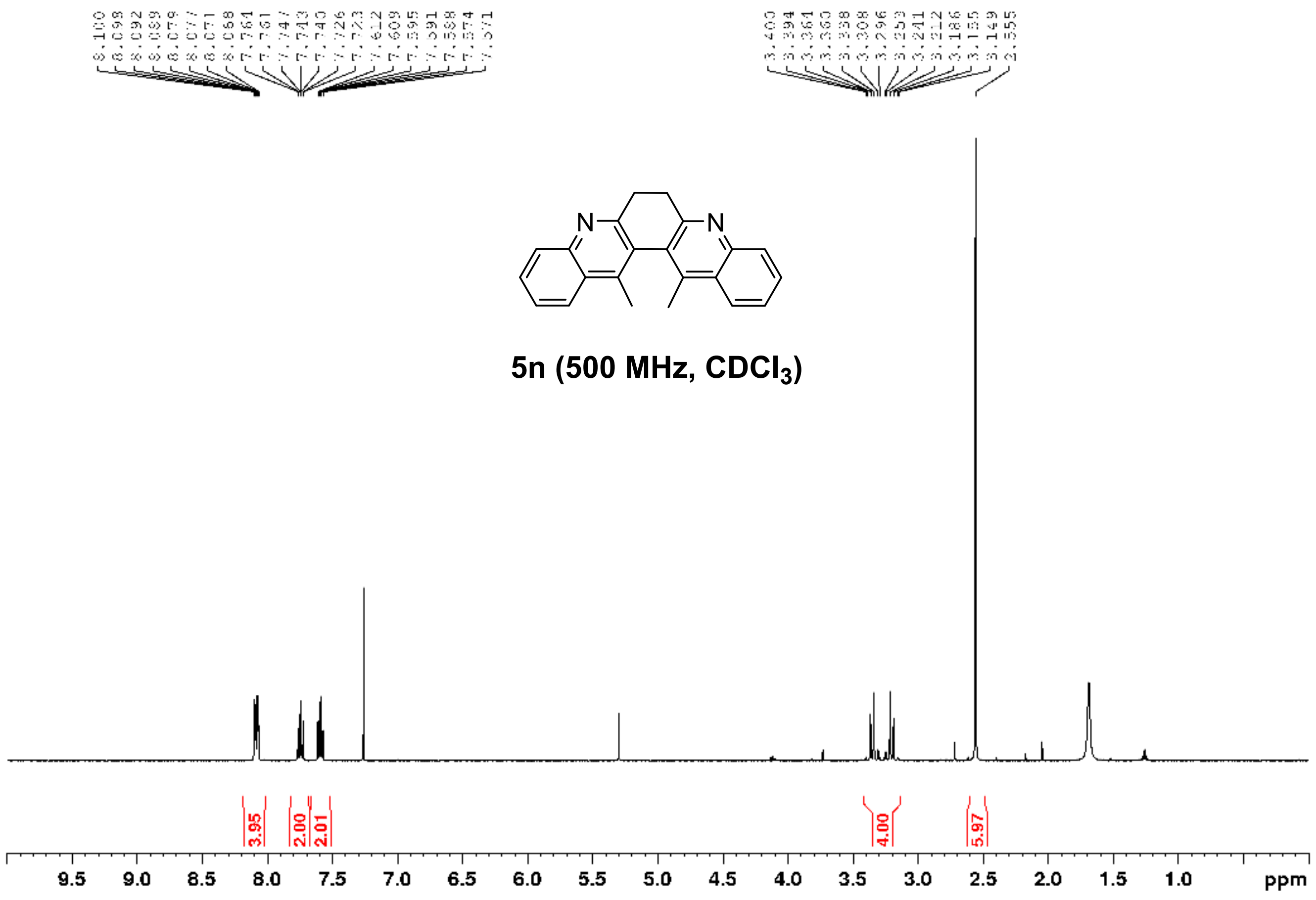

5n (500 MHz, $CDCl_3$)

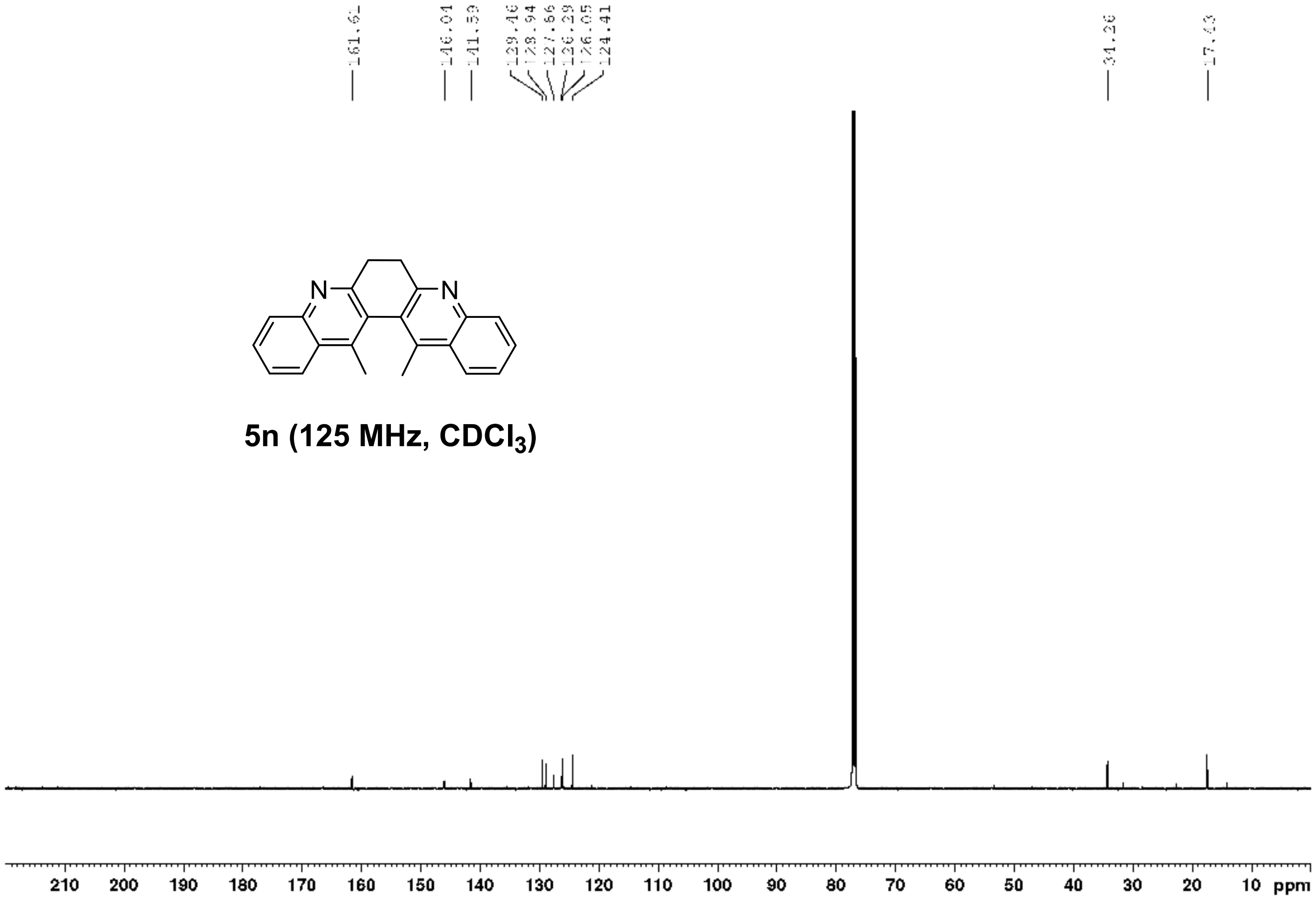
161.62
146.04
141.59
129.46
128.94
127.86
126.29
126.05
124.41
34.26
17.43
N
N
5n (125 MHz, CDCl3)
210 200 190 180 170 160 150 140 130 120 110 100 90 80 70 60 50 40 30 20 10 ppm

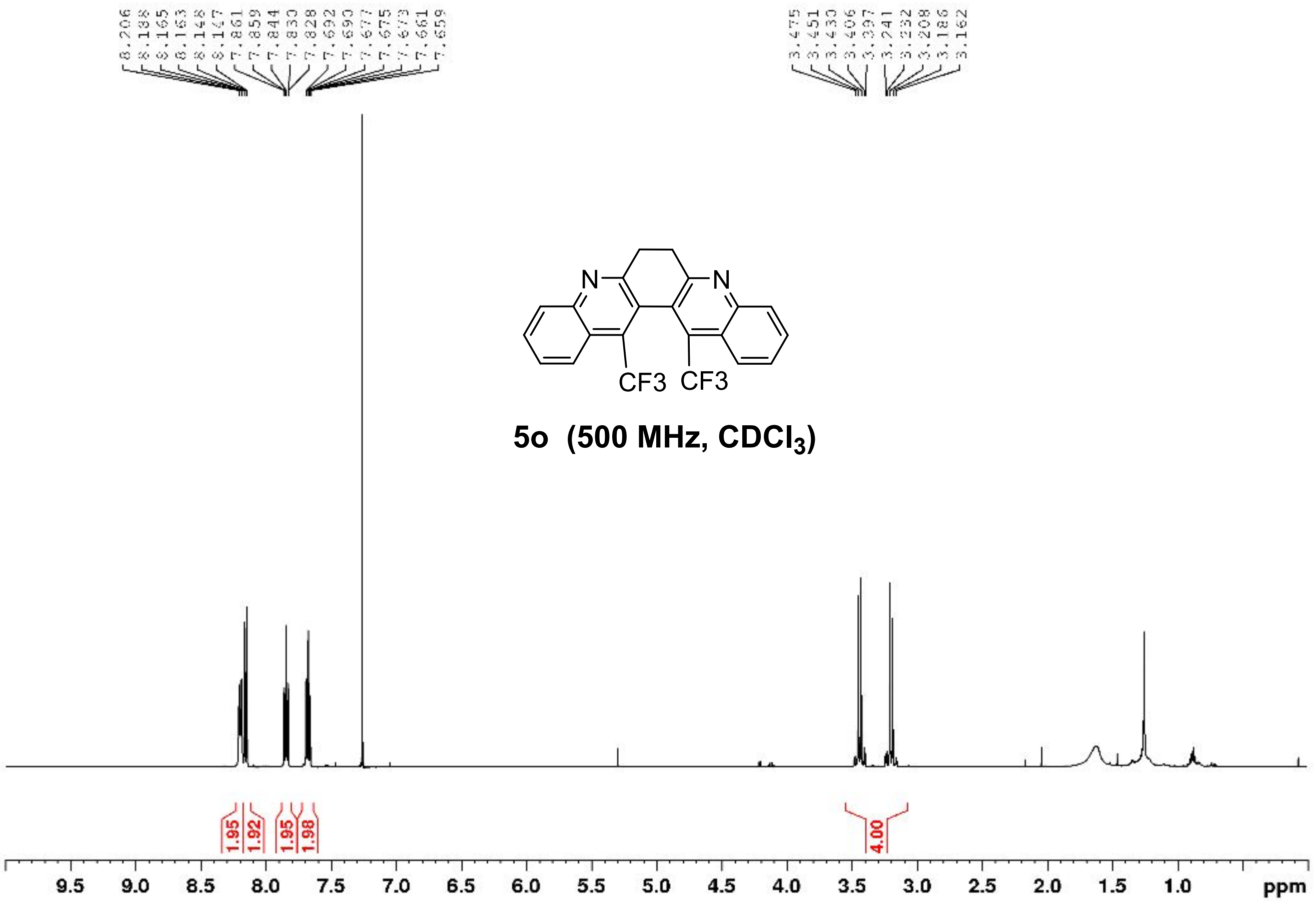

5o (500 MHz, $CDCl_3$)

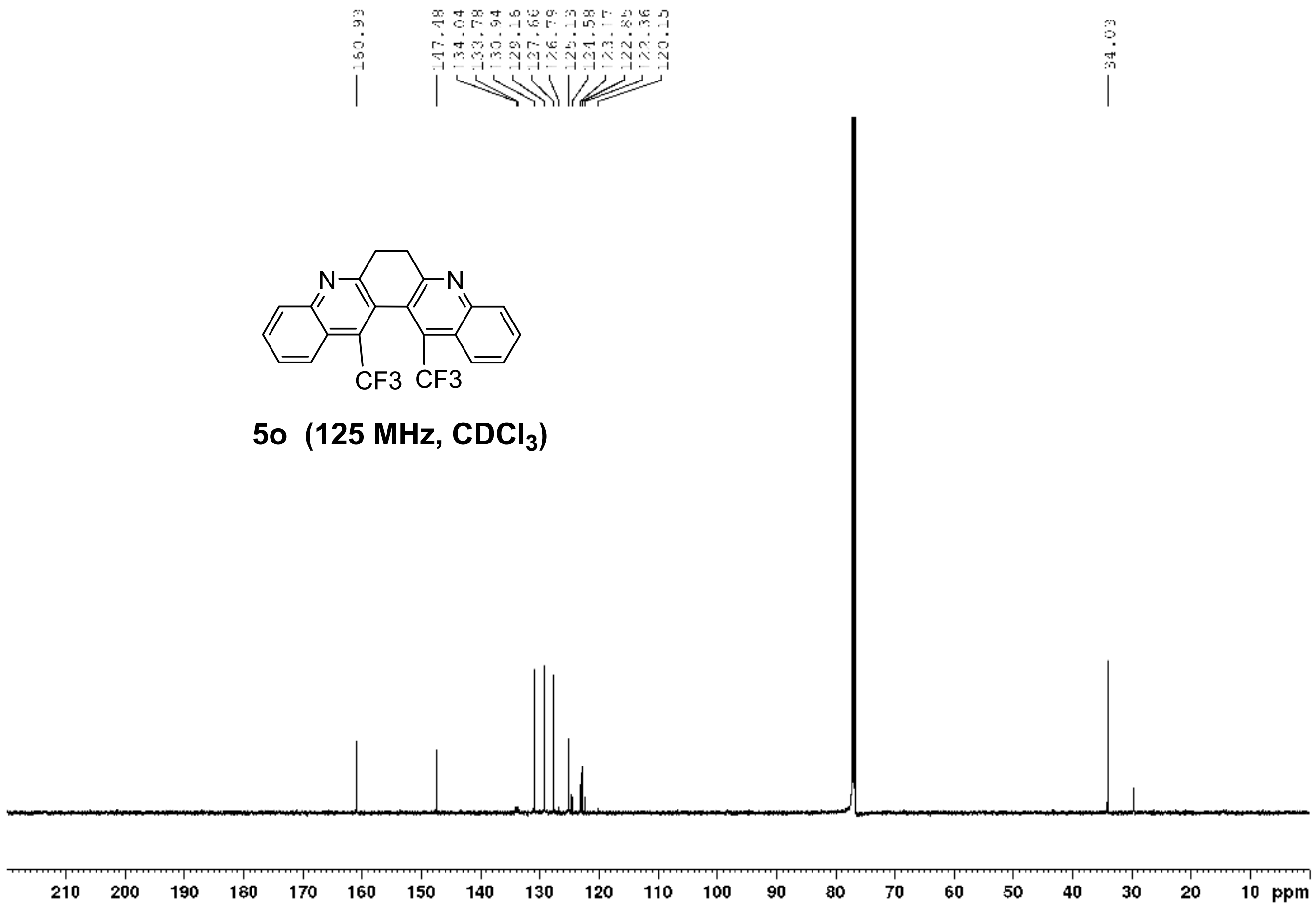

5o (125 MHz, $CDCl_3$)

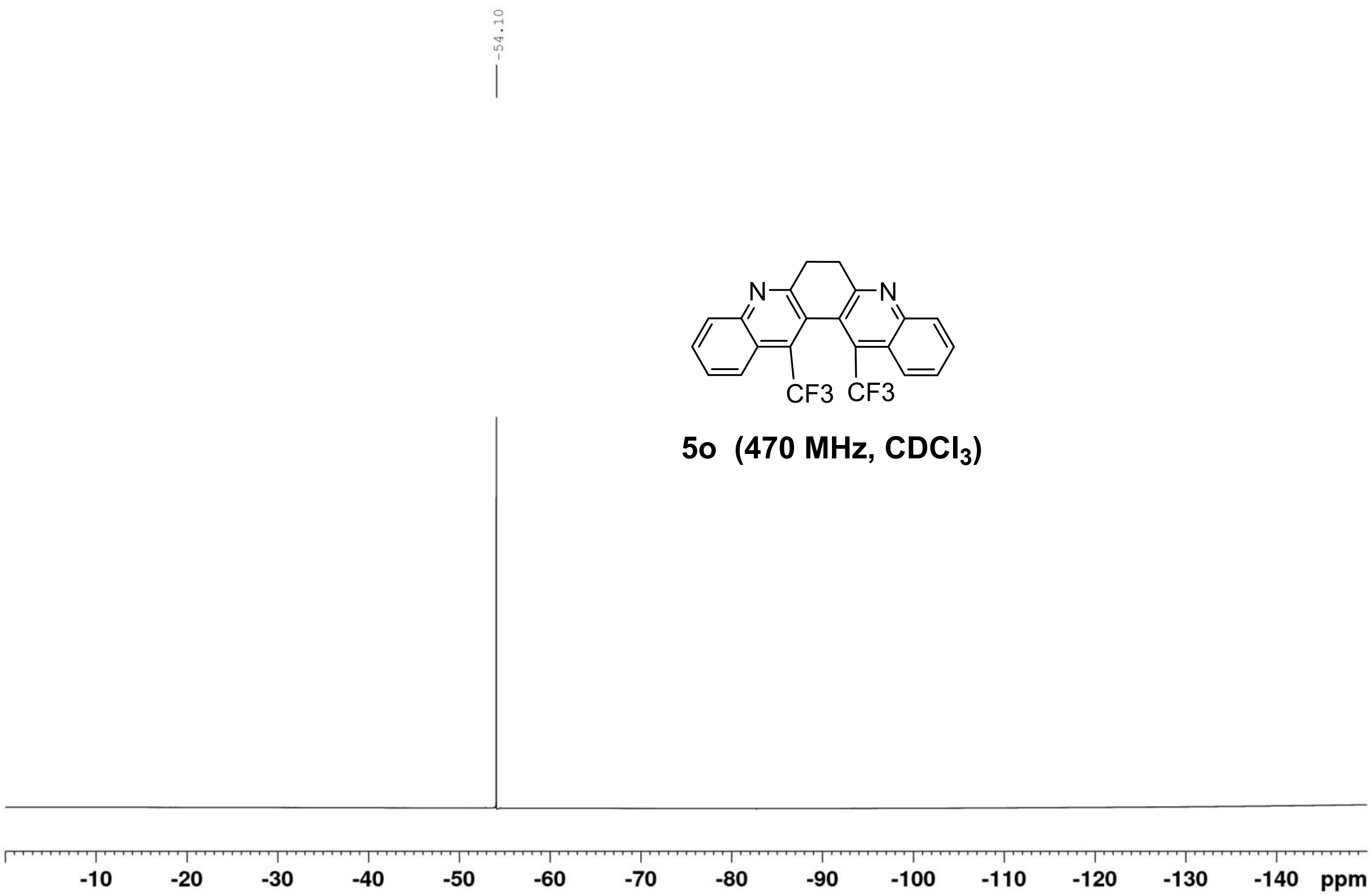

5o (470 MHz, $CDCl_3$)

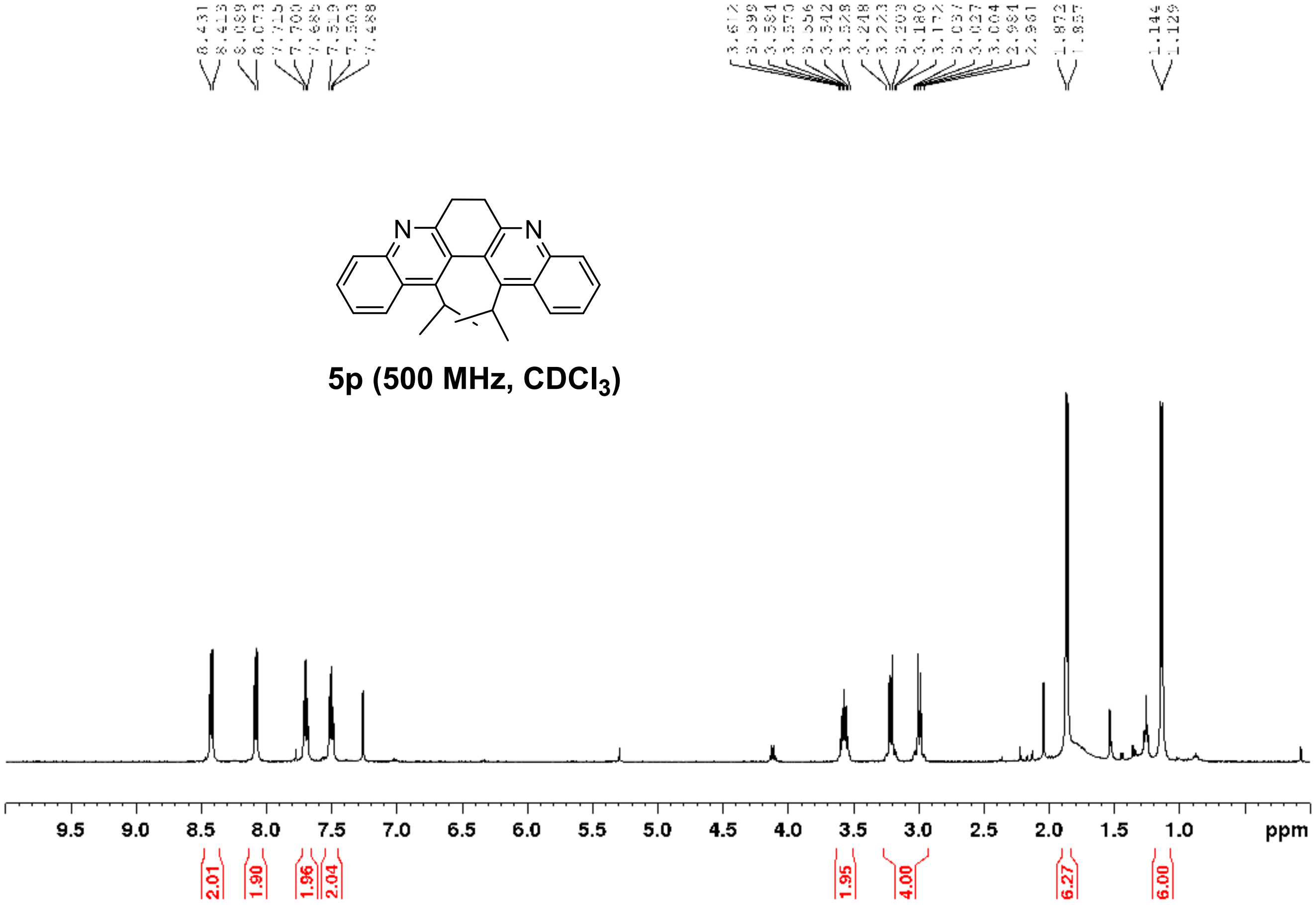

5p (500 MHz, CDCl$_3$)

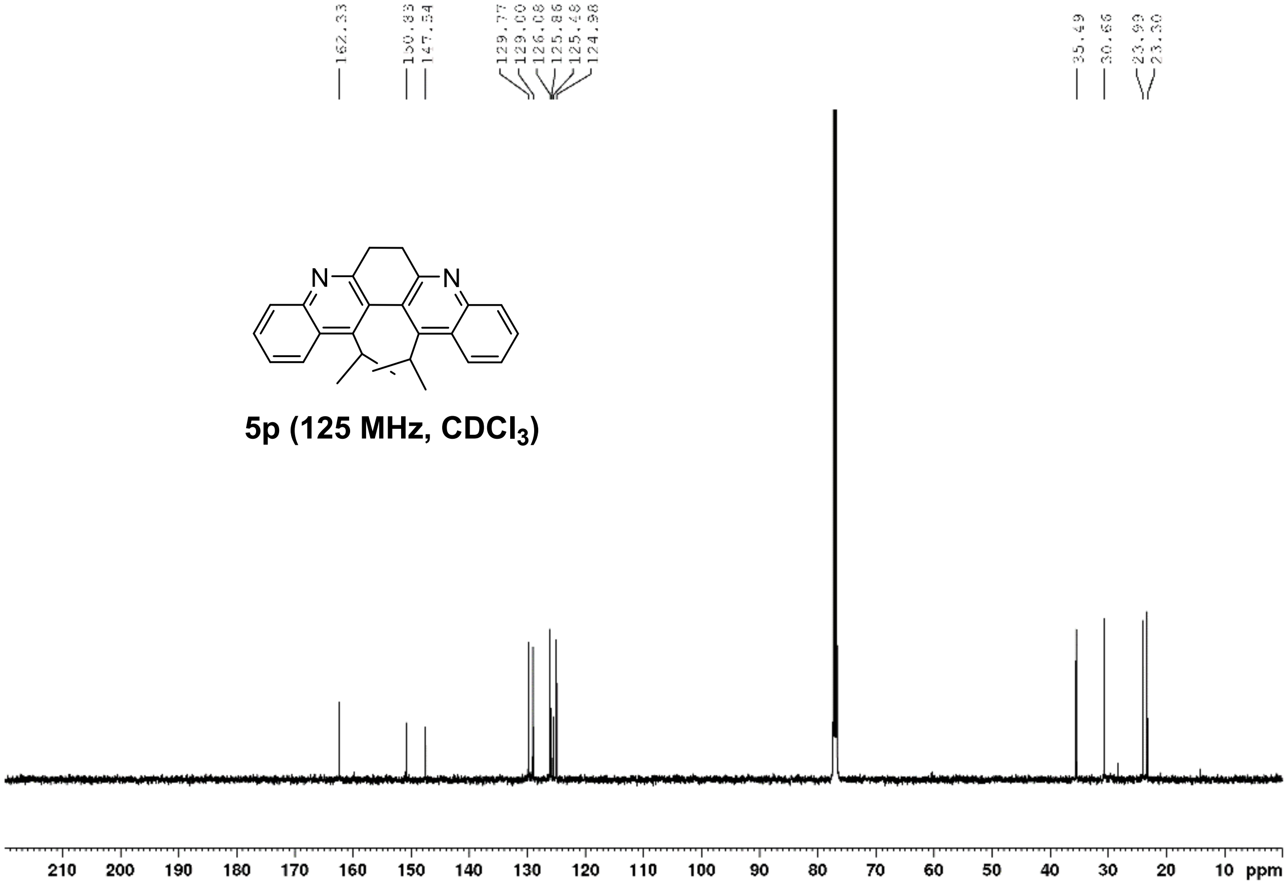

5p (125 MHz, $CDCl_3$)

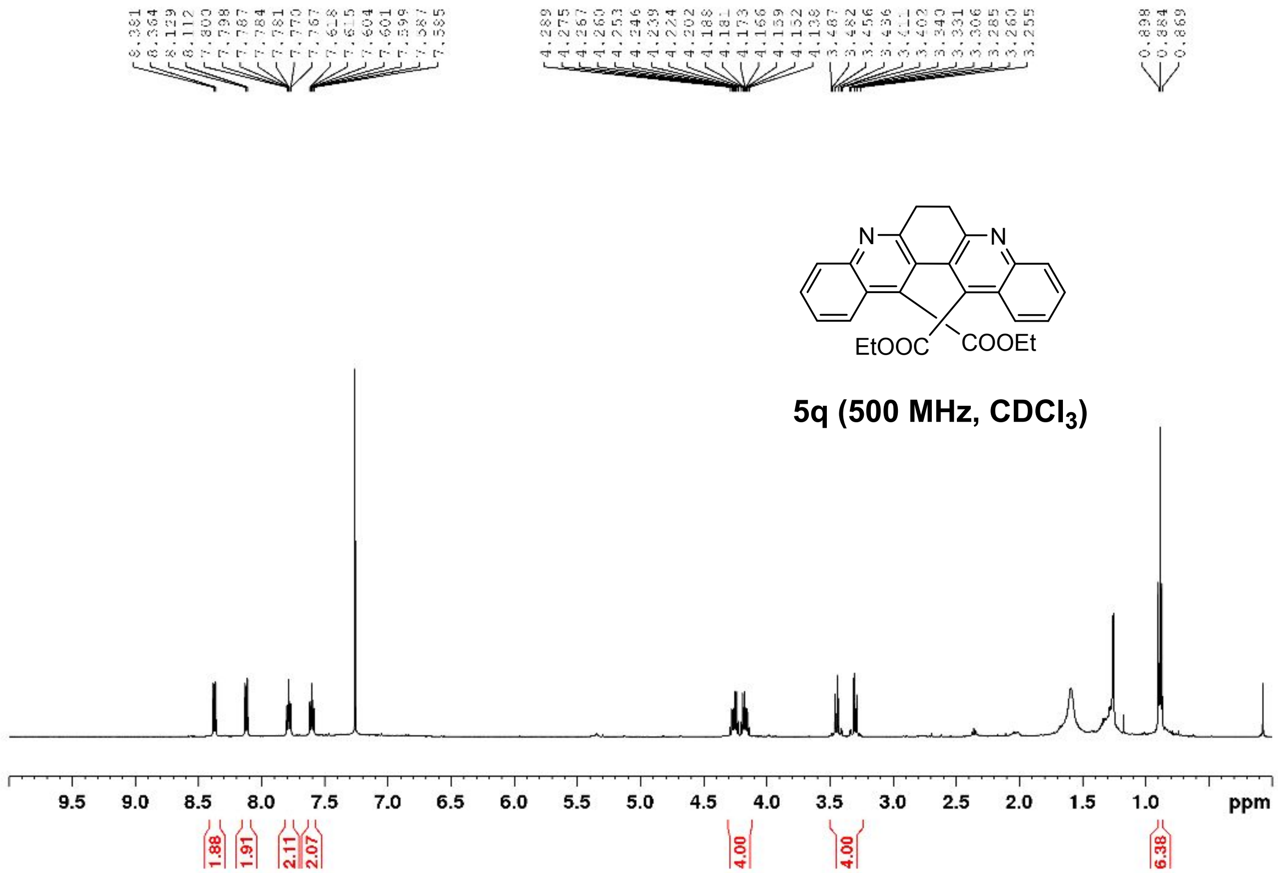

5q (500 MHz, $CDCl_3$)

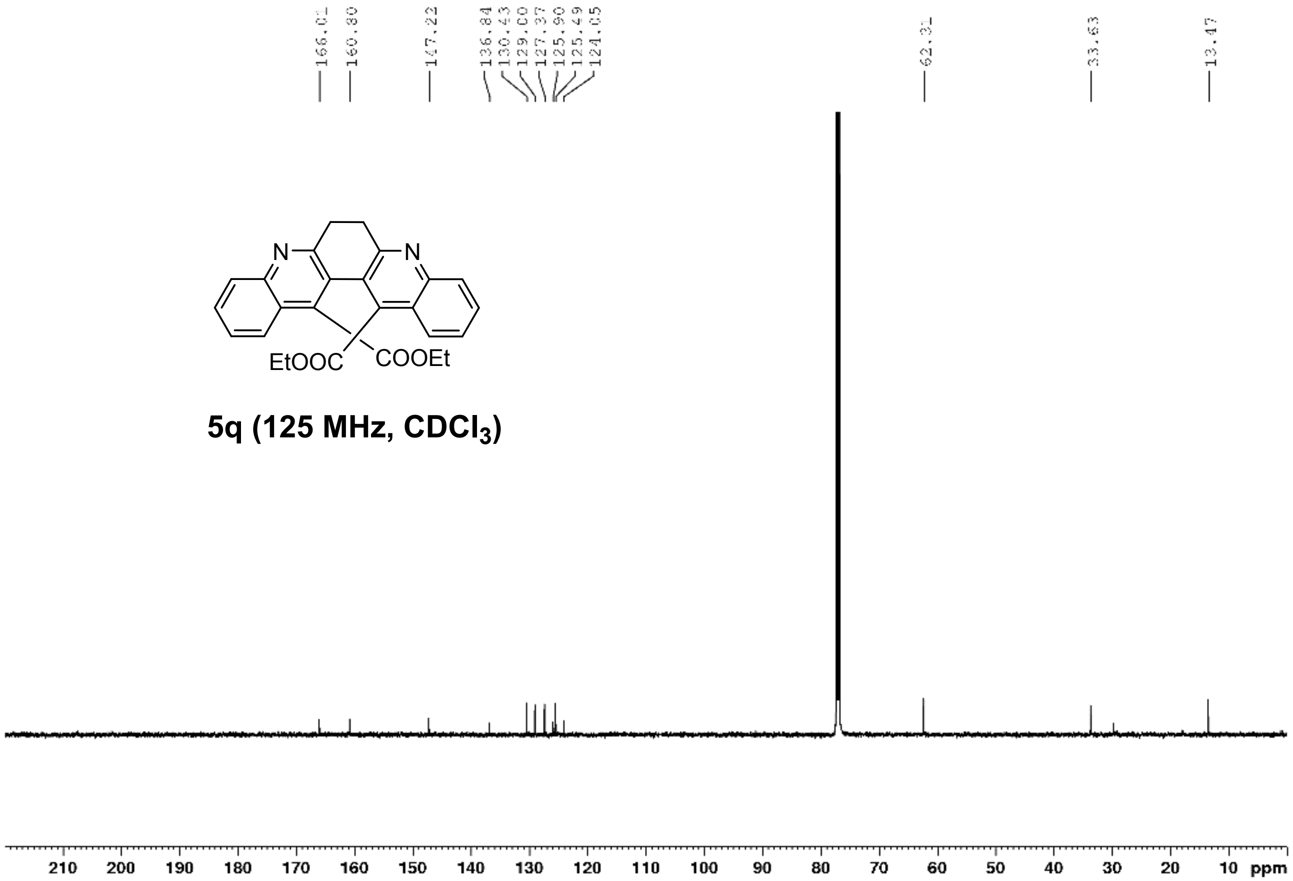

5q (125 MHz, $CDCl_3$)

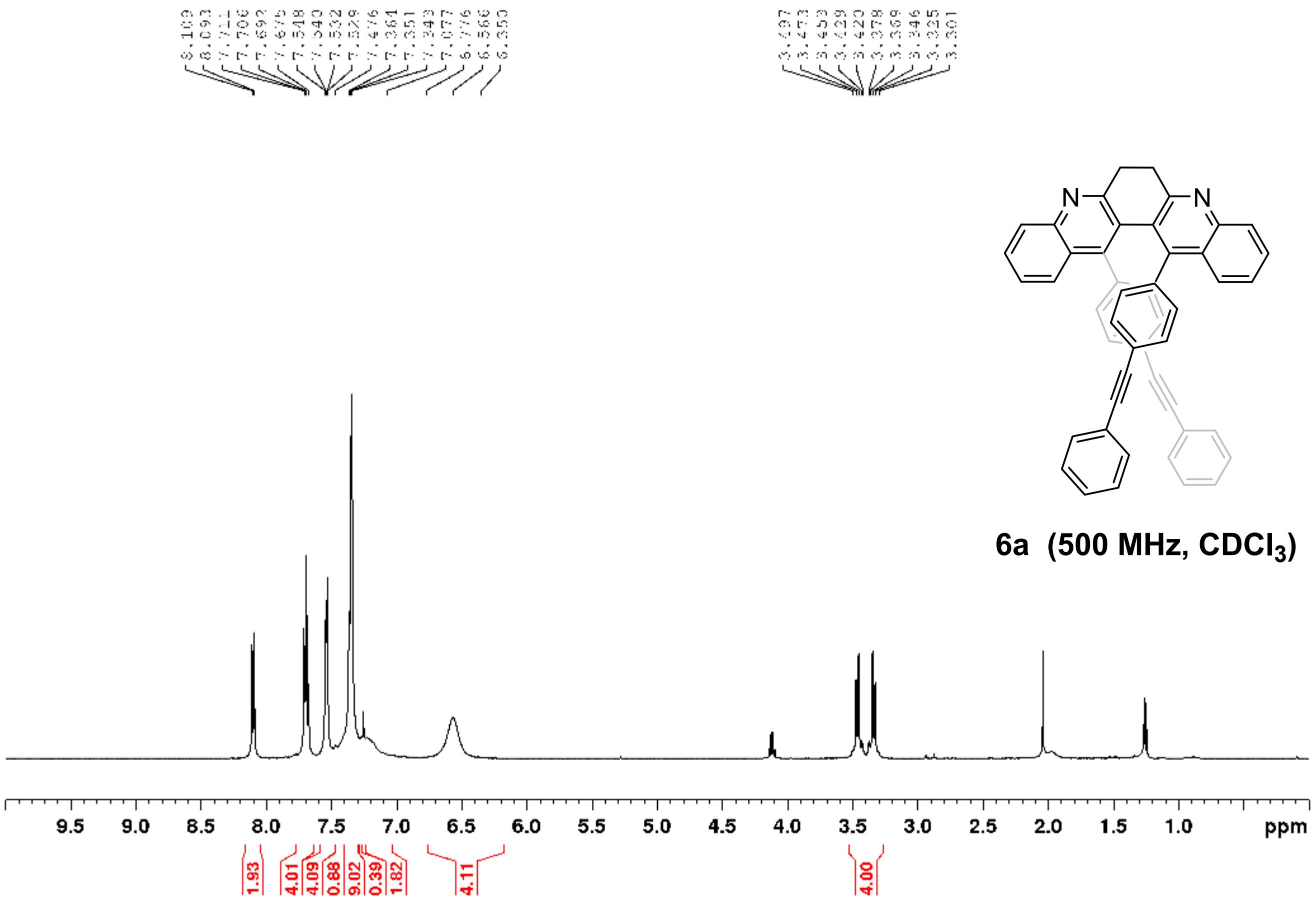

6a (500 MHz, $CDCl_3$)

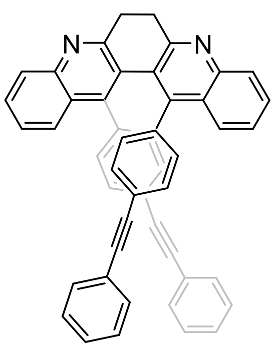

6a (125 MHz, $CDCl_3$)

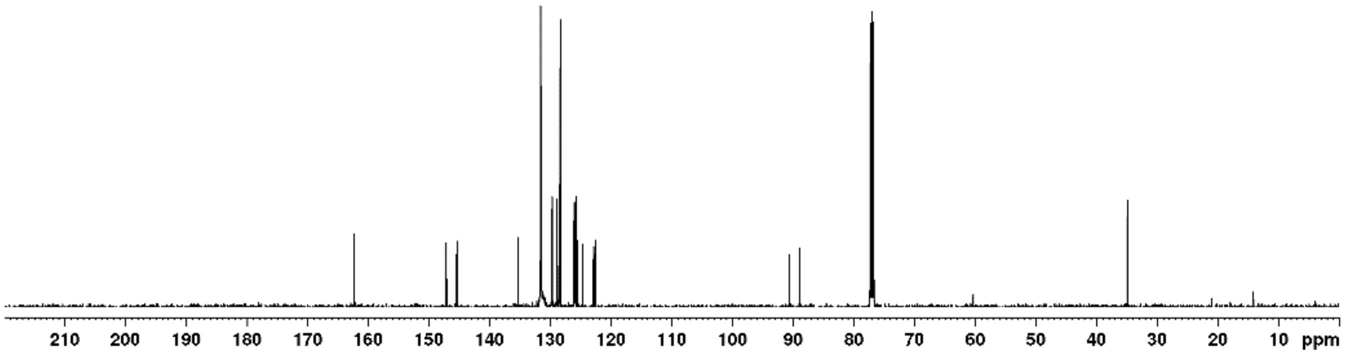

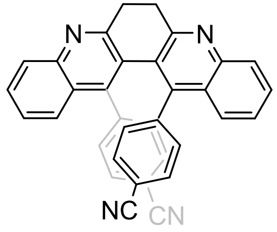

6b (500 MHz, CDCl$_3$)

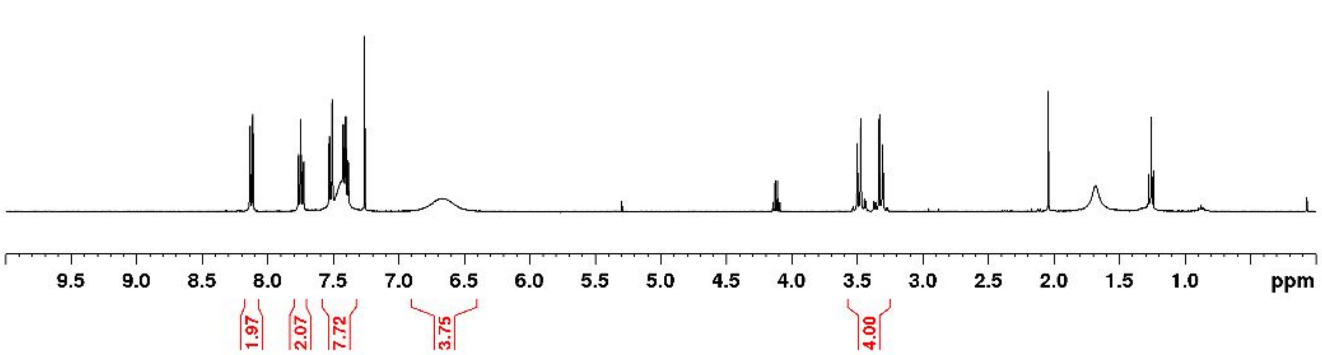

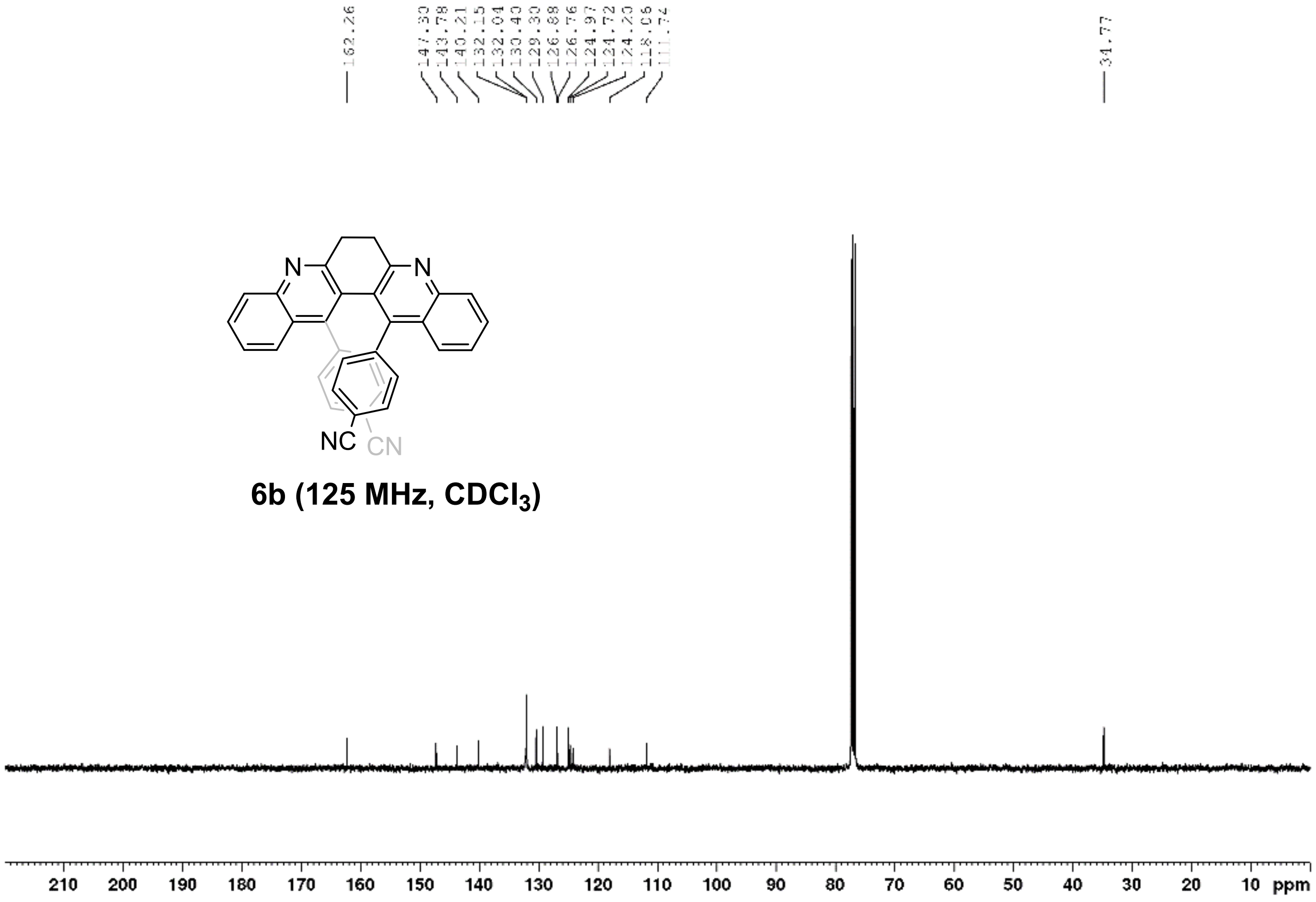

6b (125 MHz, $CDCl_3$)

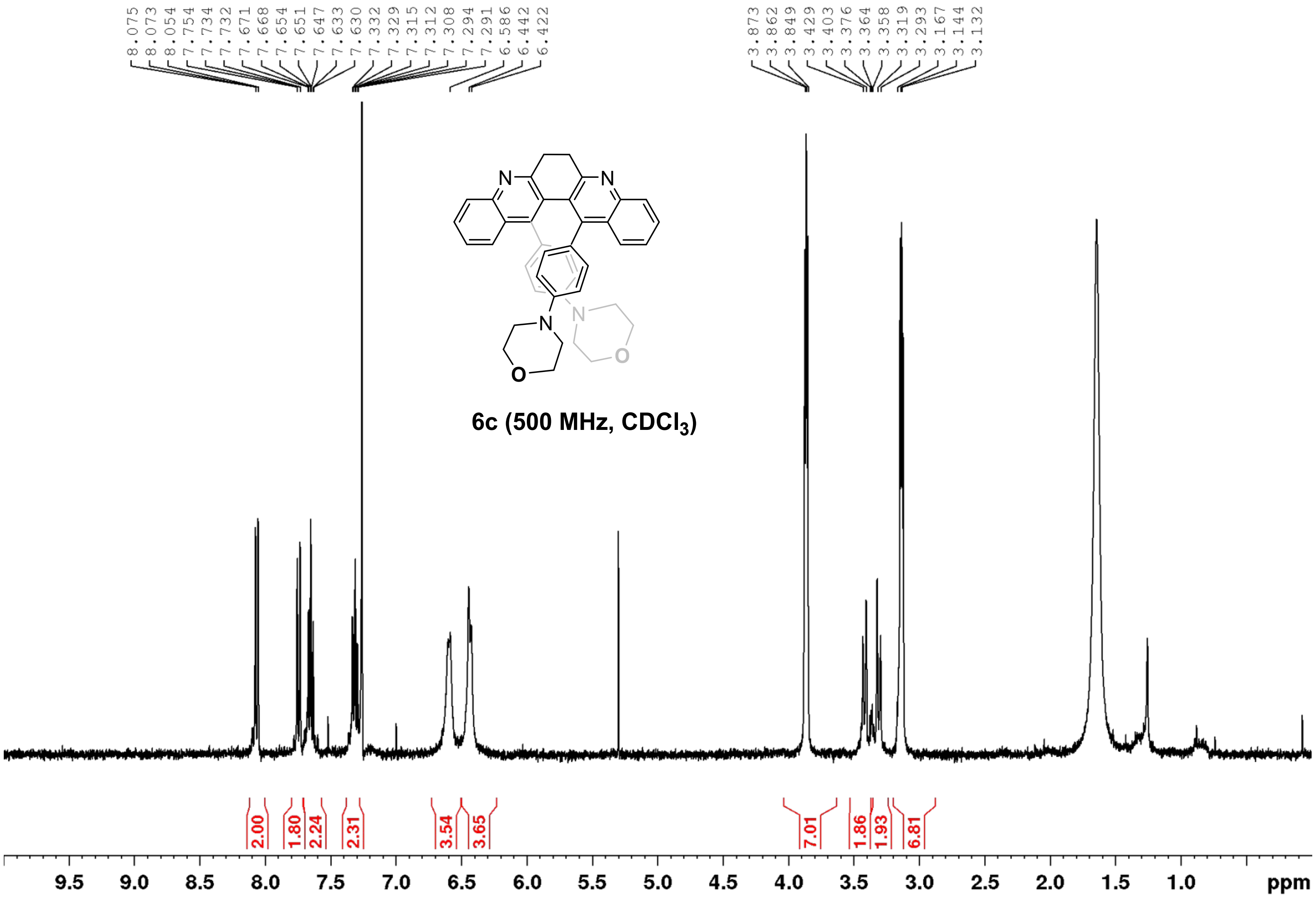
8.075
8.073
8.054
7.754
7.734
7.732
7.671
7.668
7.654
7.651
7.647
7.633
7.630
7.332
7.329
7.315
7.312
7.308
7.294
7.291
6.586
6.442
6.422
3.873
3.862
3.849
3.429
3.403
3.376
3.364
3.358
3.319
3.293
3.167
3.144
3.132
6c (500 MHz, CDCl3)
2.00
1.80
2.24
2.31
3.54
3.65
7.01
1.86
1.93
6.81
9.5
9.0
8.5
8.0
7.5
7.0
6.5
6.0
5.5
5.0
4.5
4.0
3.5
3.0
2.5
2.0
1.5
1.0
ppm

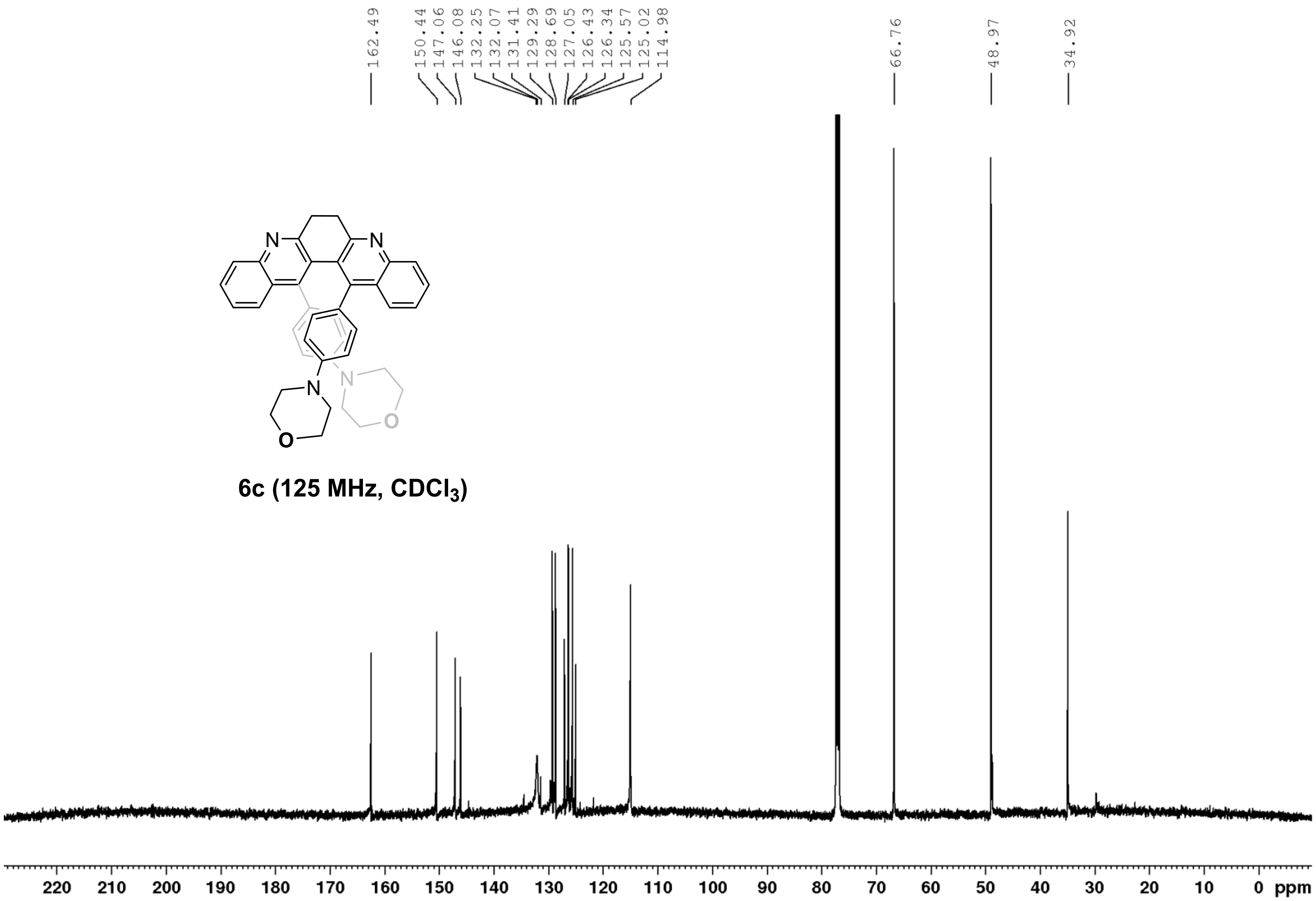

6c (125 MHz, $CDCl_3$)

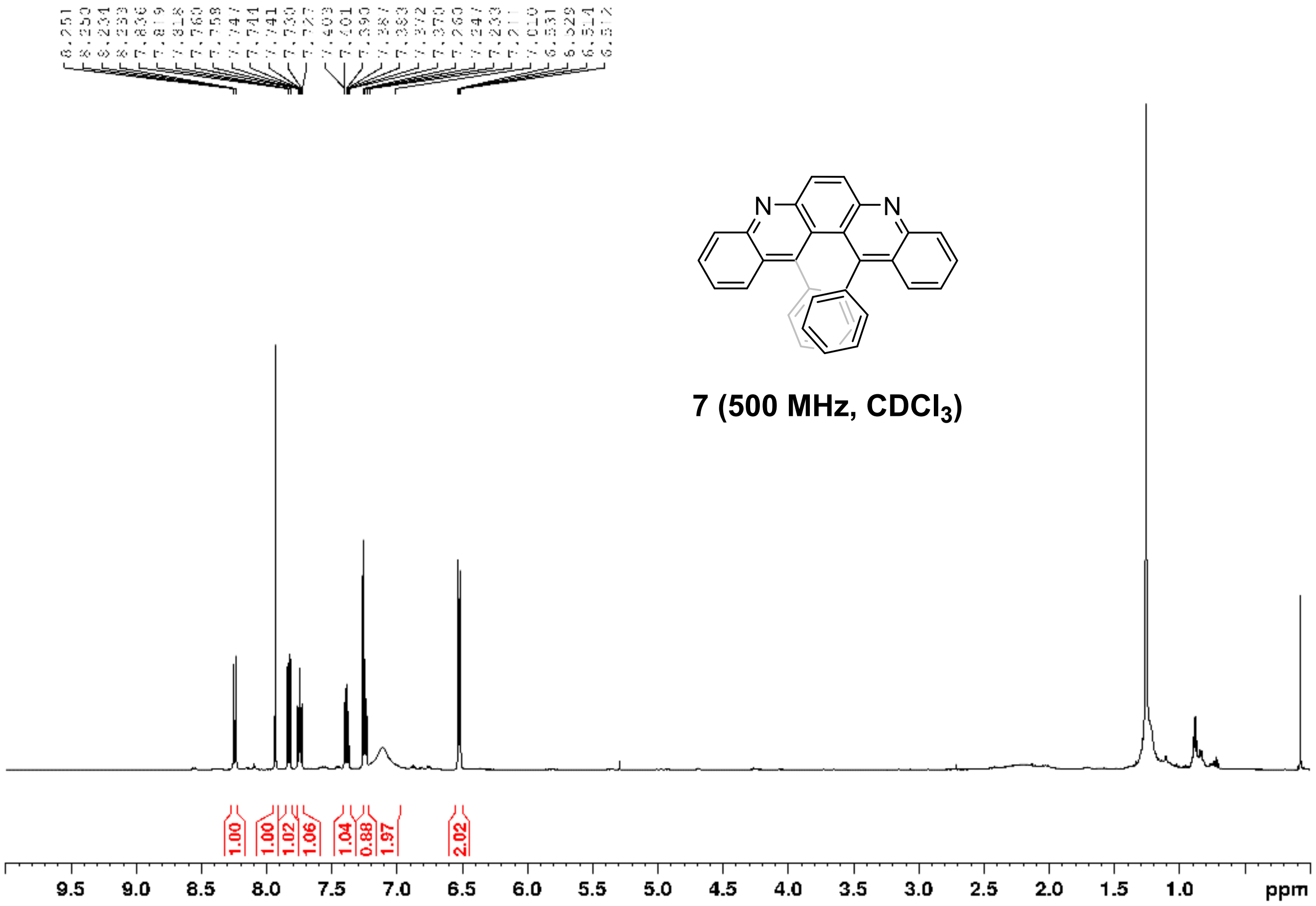

7 (500 MHz, $CDCl_3$)

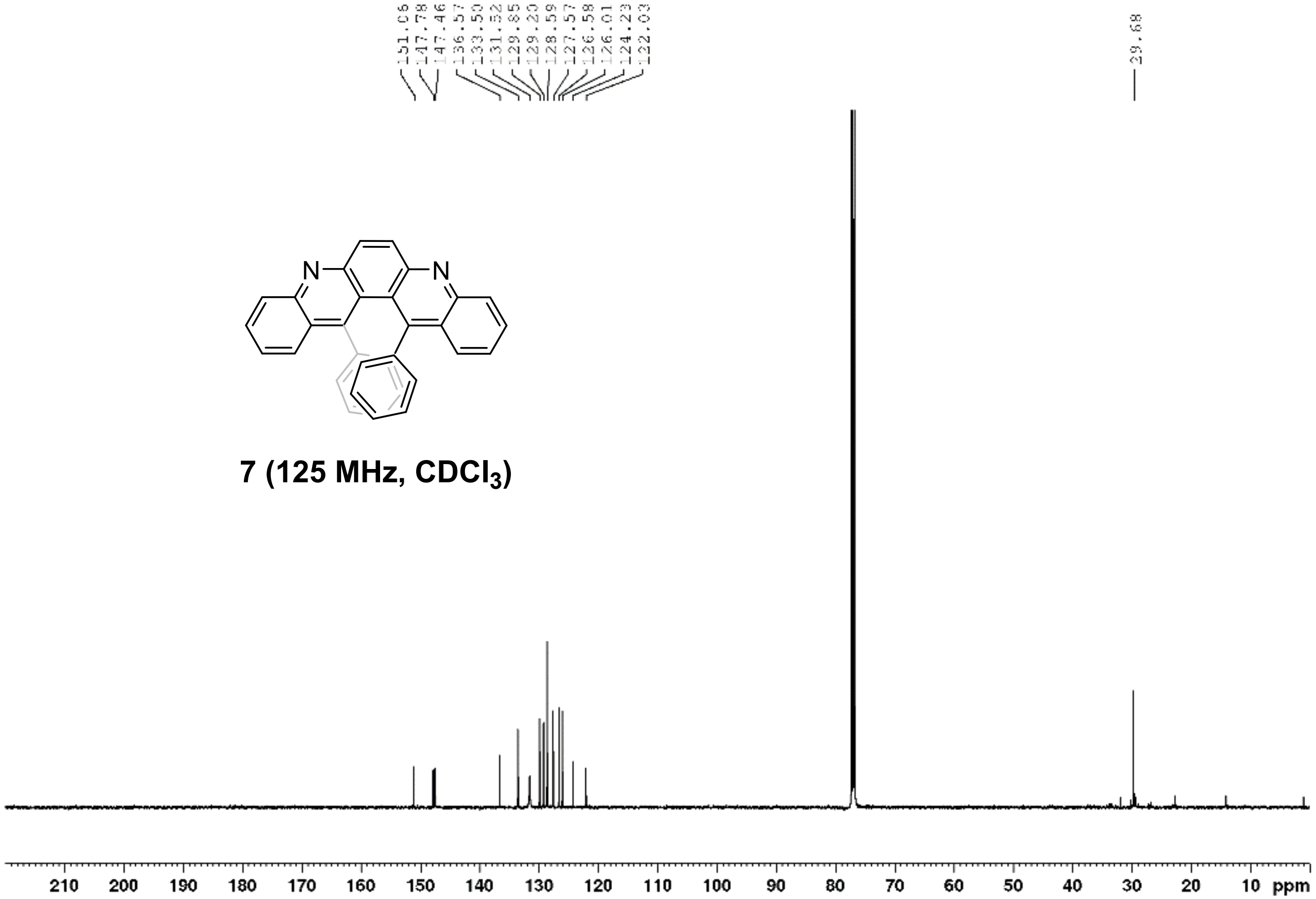

7 (125 MHz, $CDCl_3$)

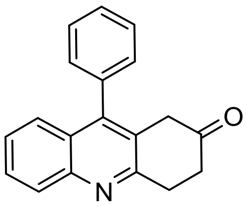

8 (500 MHz, $CDCl_3$)

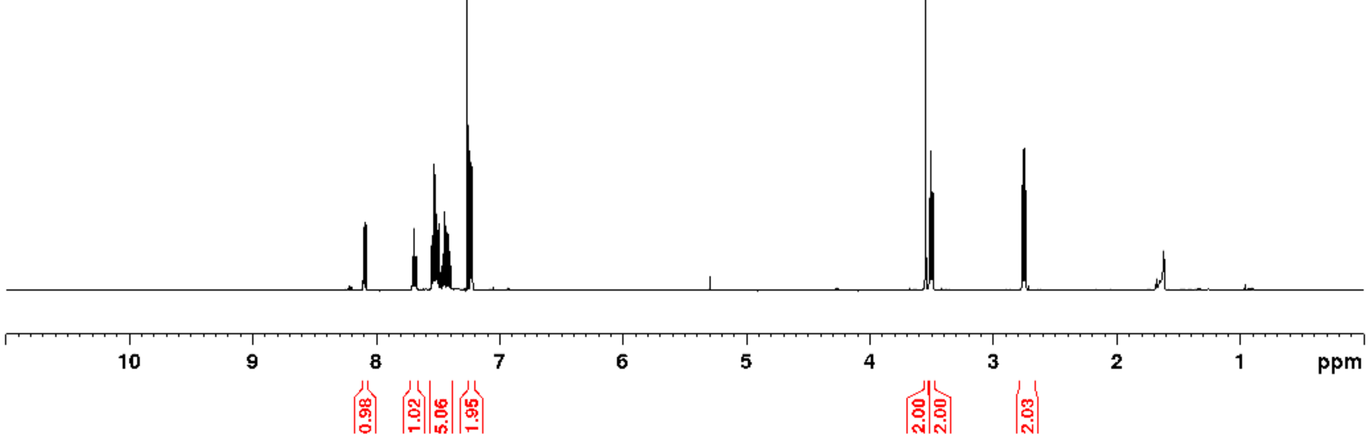

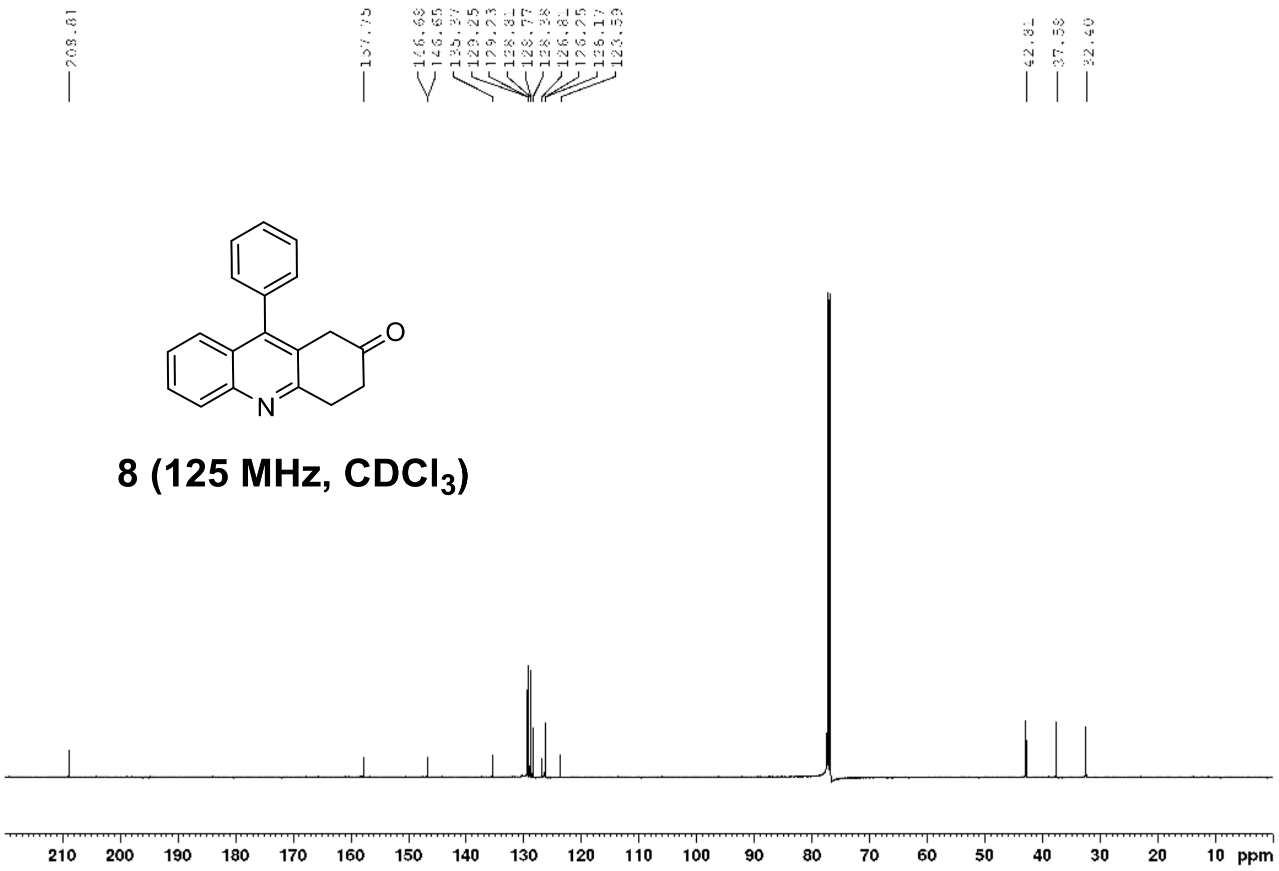

8 (125 MHz, $CDCl_3$)

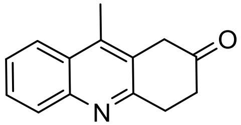

9 (500 MHz, $CDCl_3$)

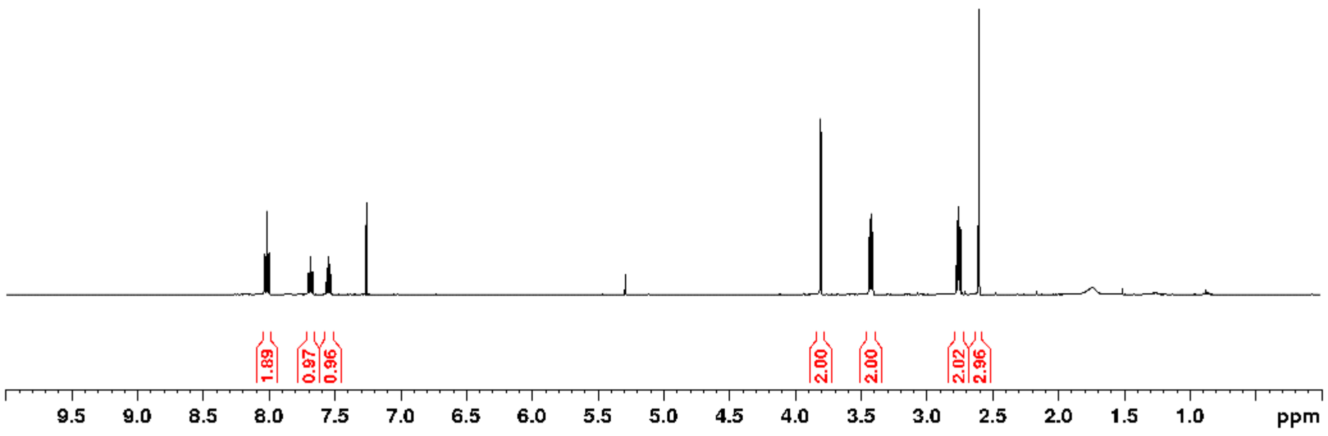

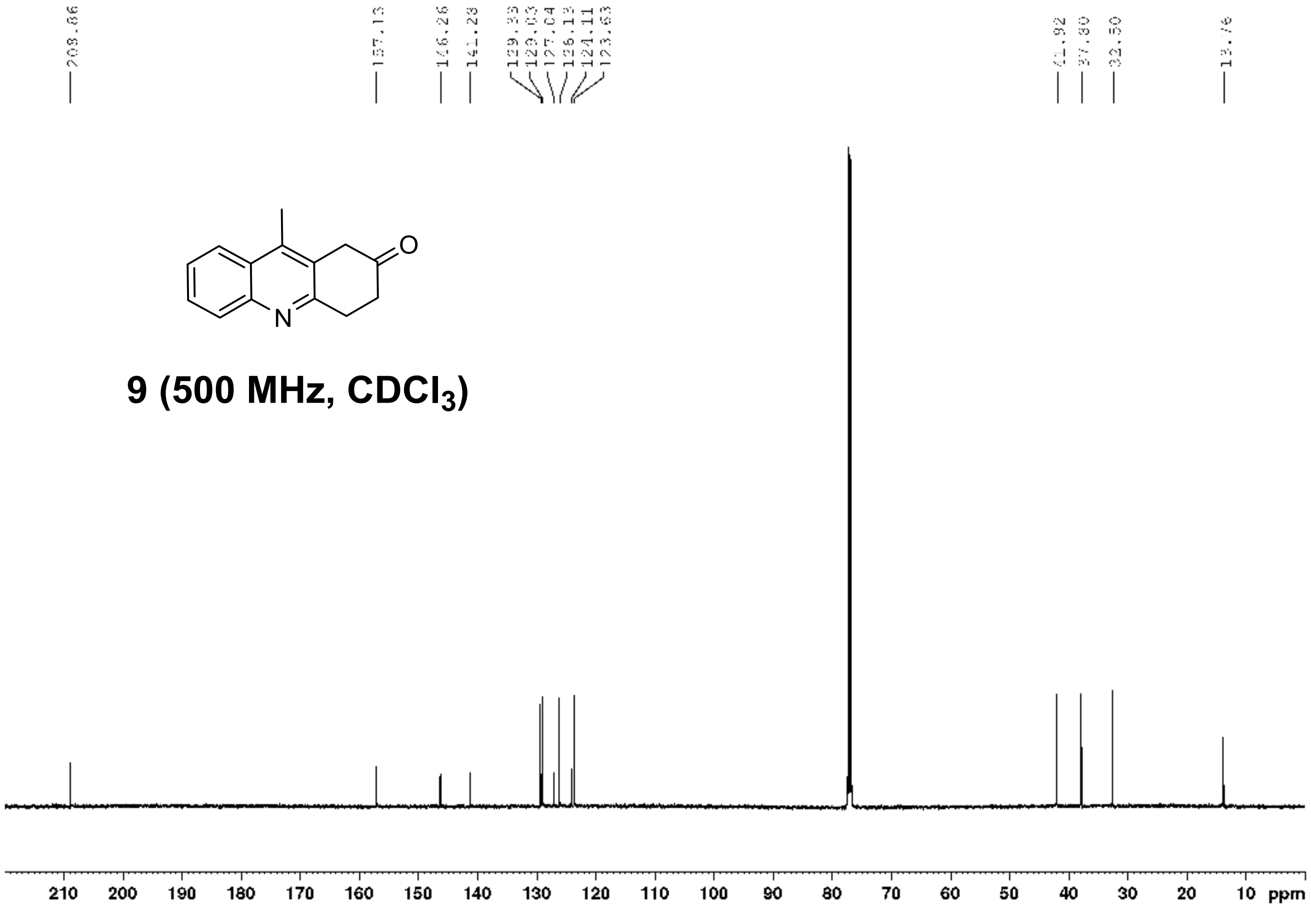

208.86
157.13
146.26
141.28
129.33
129.03
127.04
126.13
124.11
123.63
41.92
37.80
32.50
13.76
N
O
9 (500 MHz, CDCl3)
210 200 190 180 170 160 150 140 130 120 110 100 90 80 70 60 50 40 30 20 10 ppm

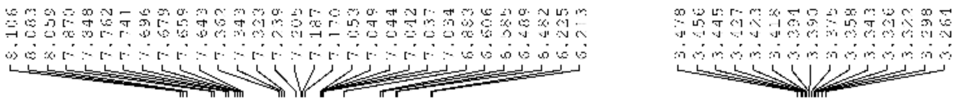

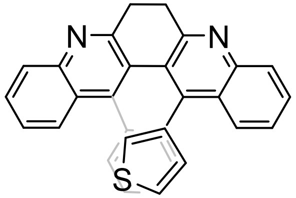

10a (500 MHz, $CDCl_3$)

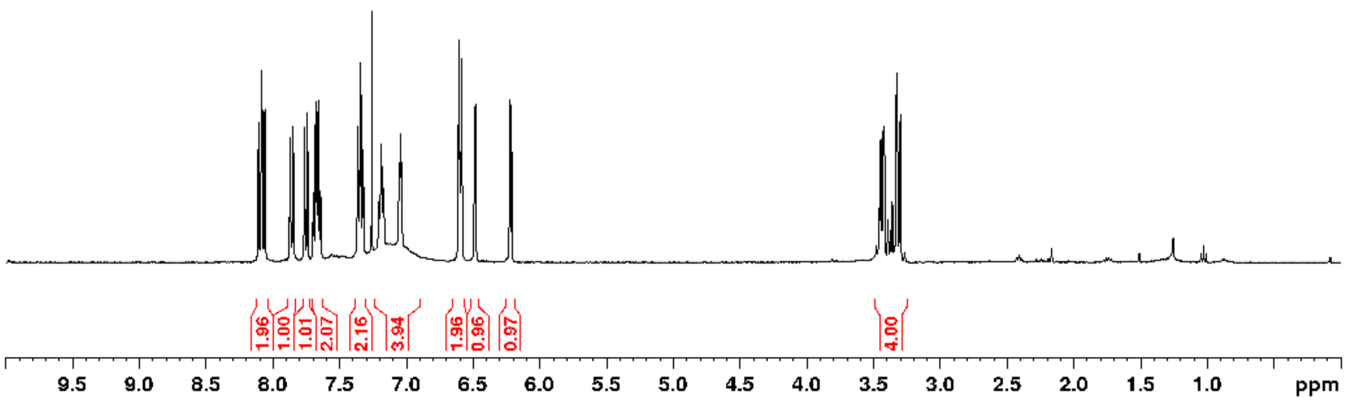

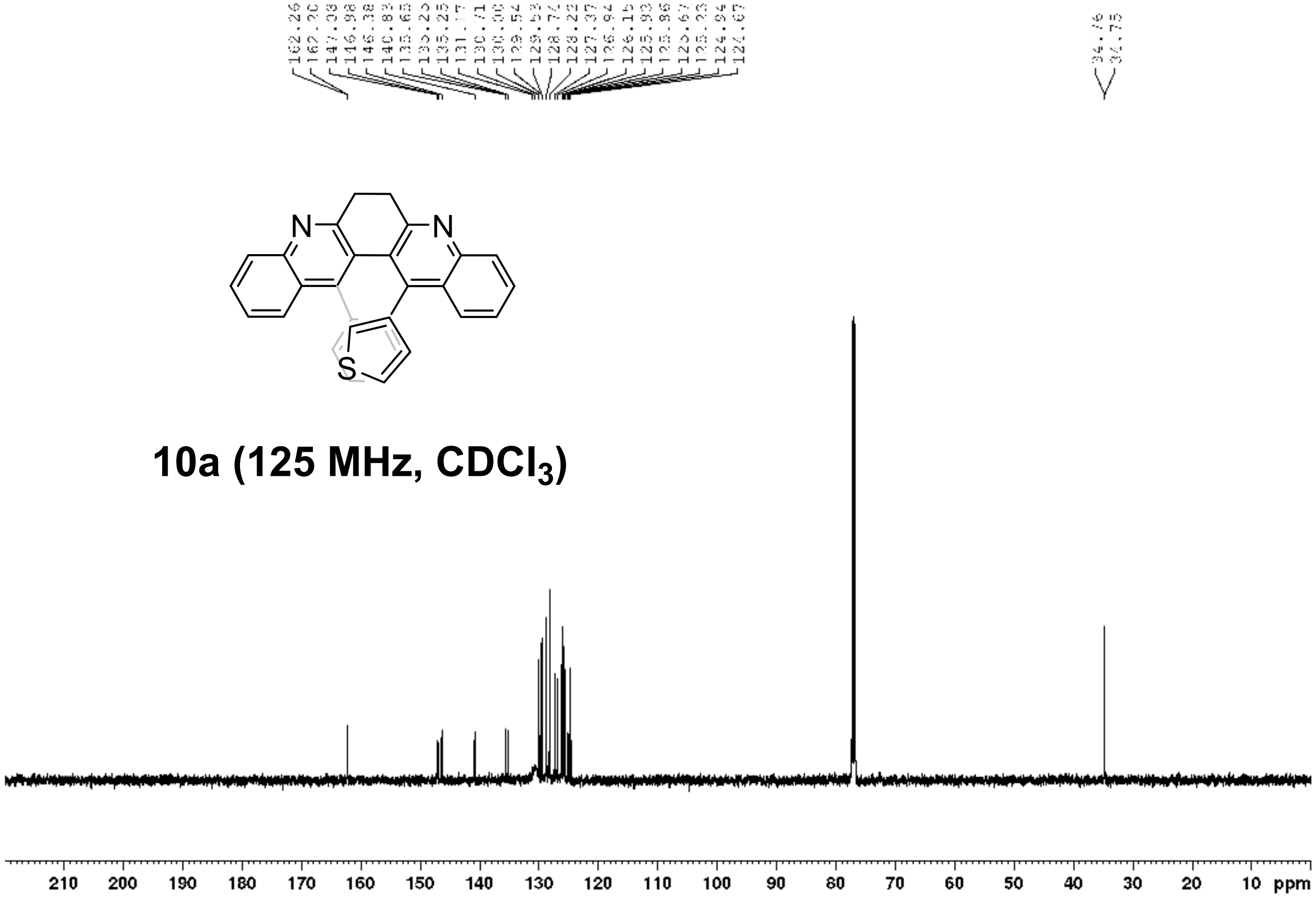

10a (125 MHz, $CDCl_3$)

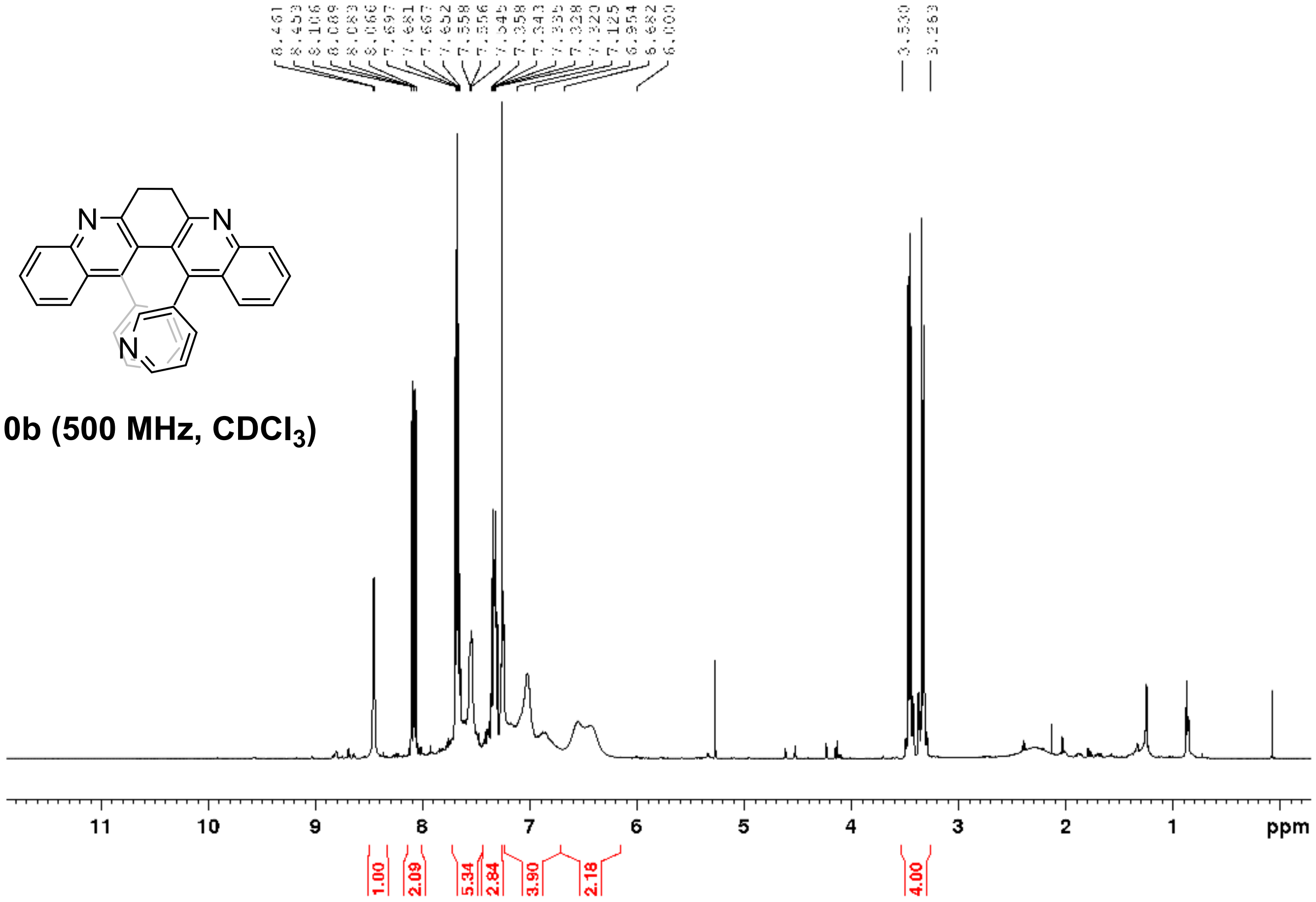

10b (500 MHz, $CDCl_3$)

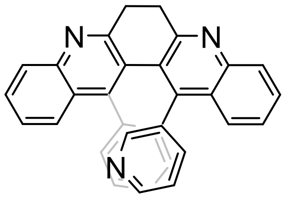

10b (125 MHz, $CDCl_3$)

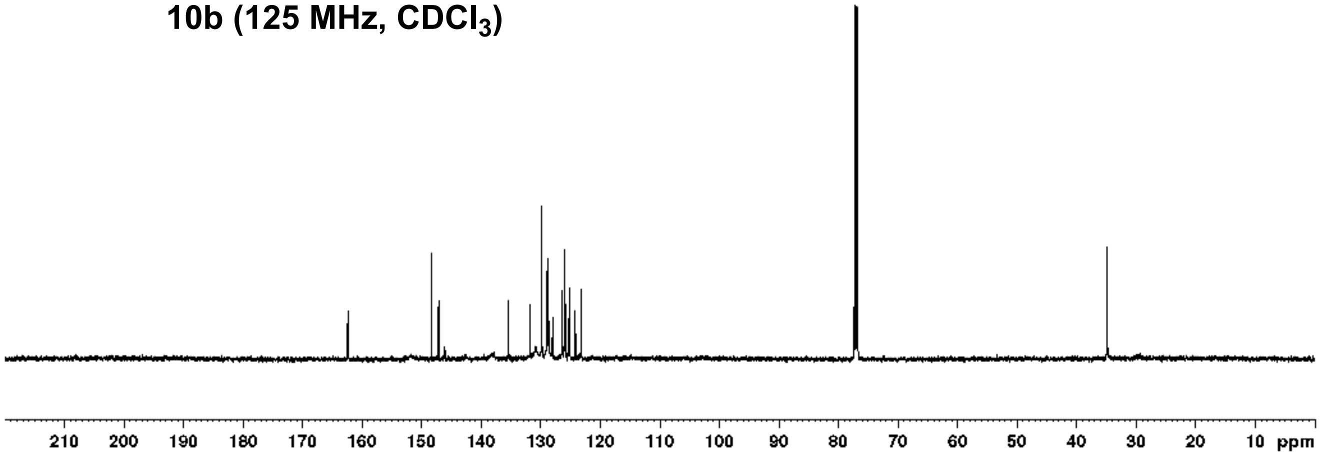

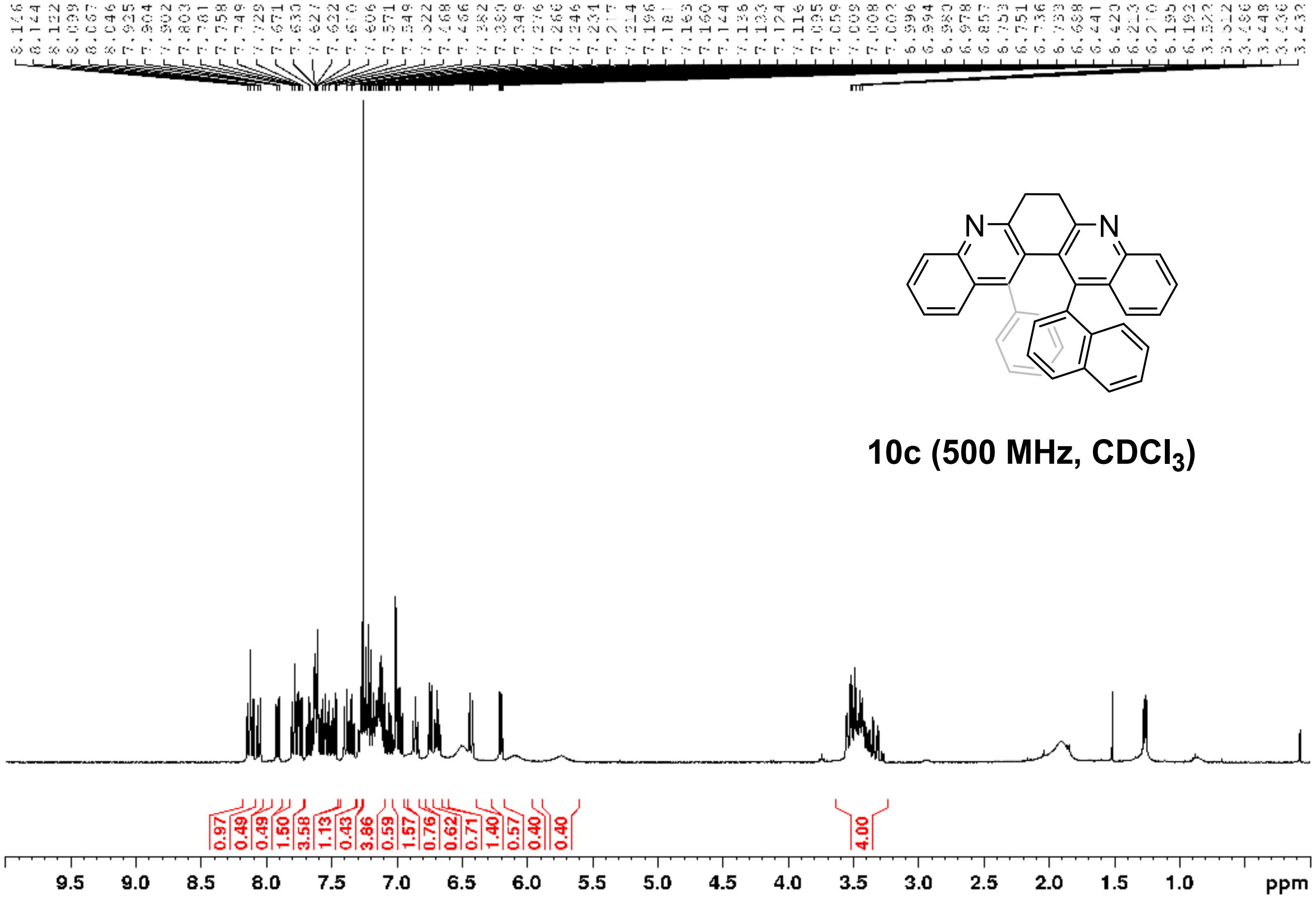

10c (500 MHz, $CDCl_3$)

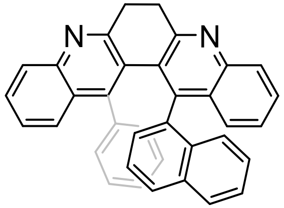

10c (125 MHz, $CDCl_3$)

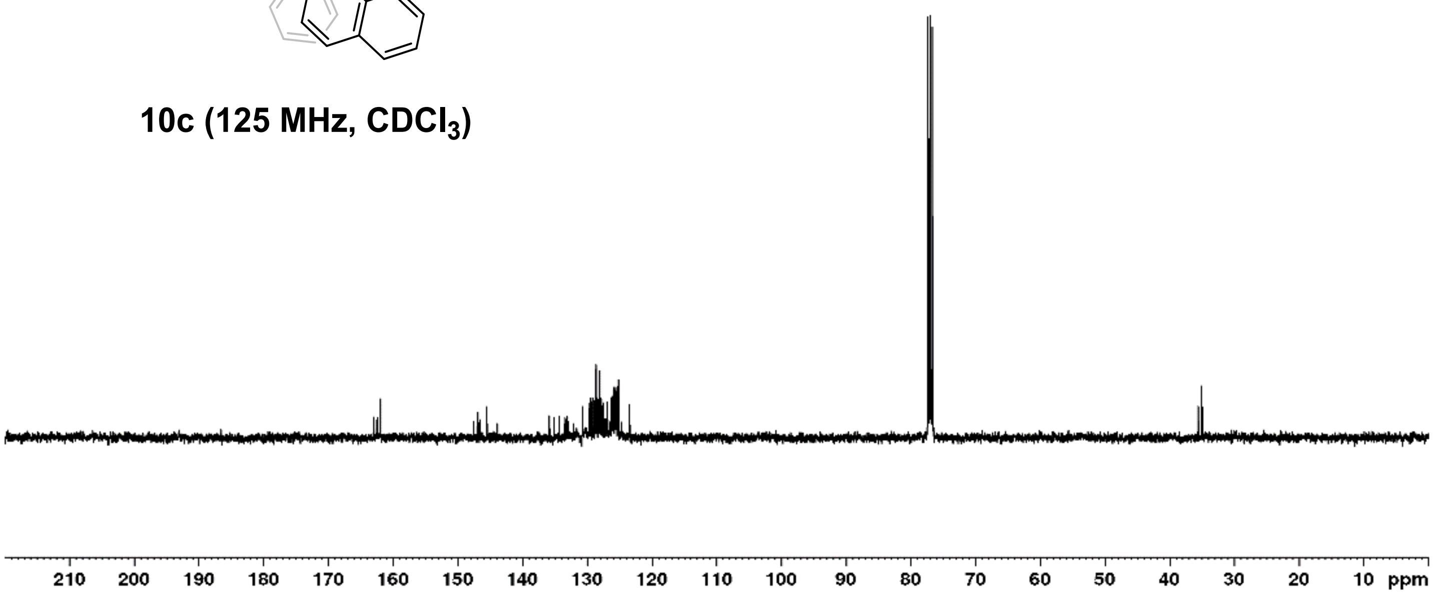

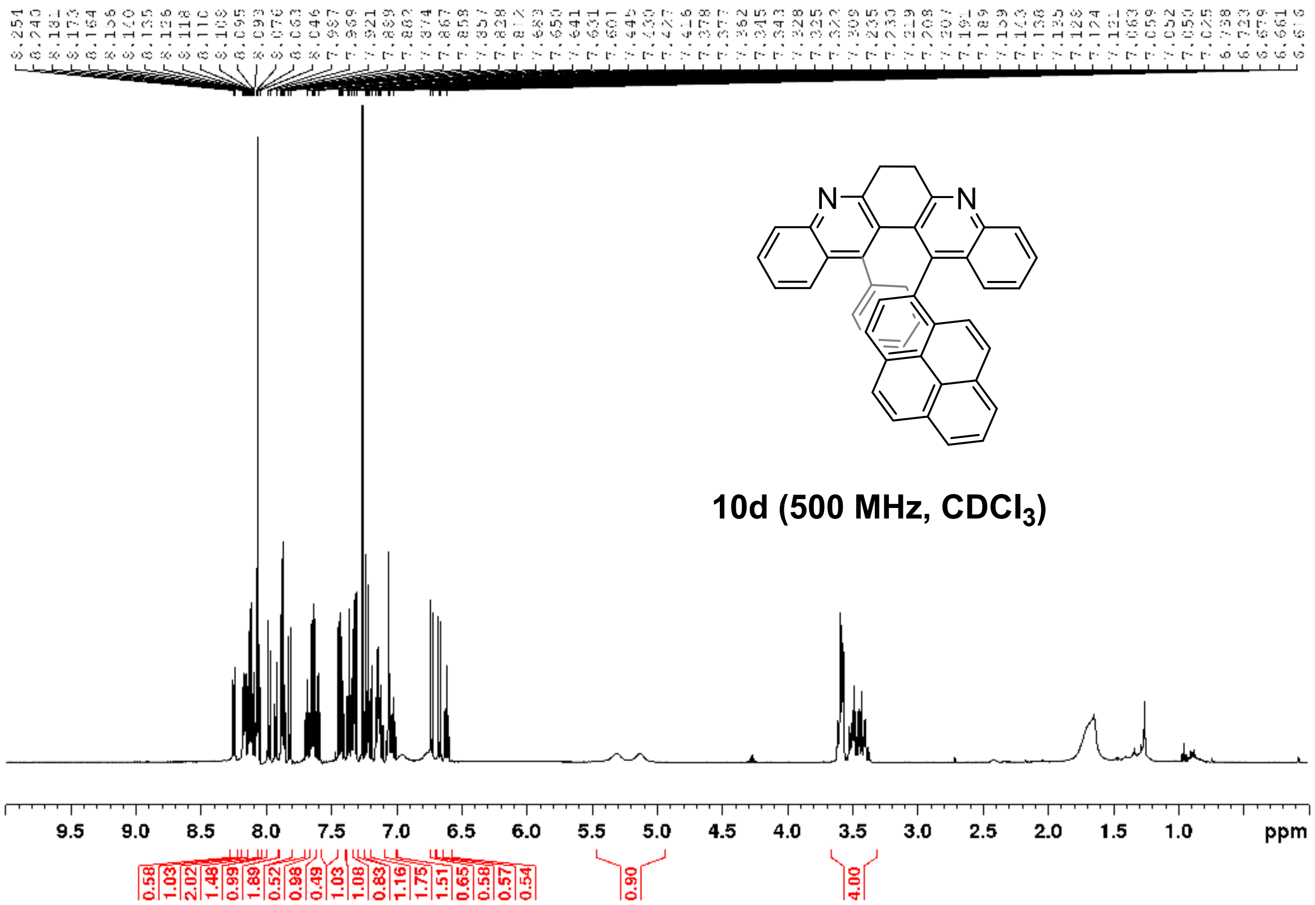

10d (500 MHz, $CDCl_3$)

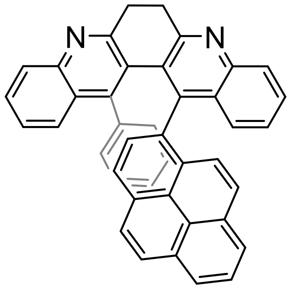

10d (125 MHz, $CDCl_3$)

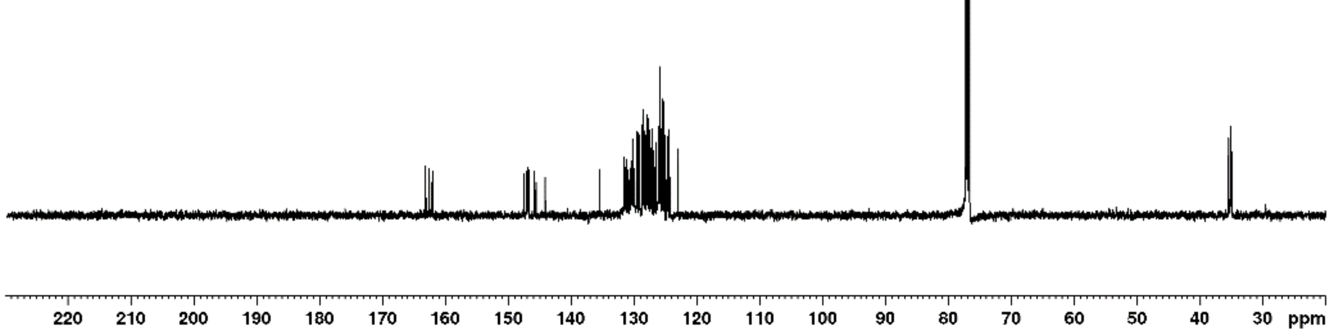

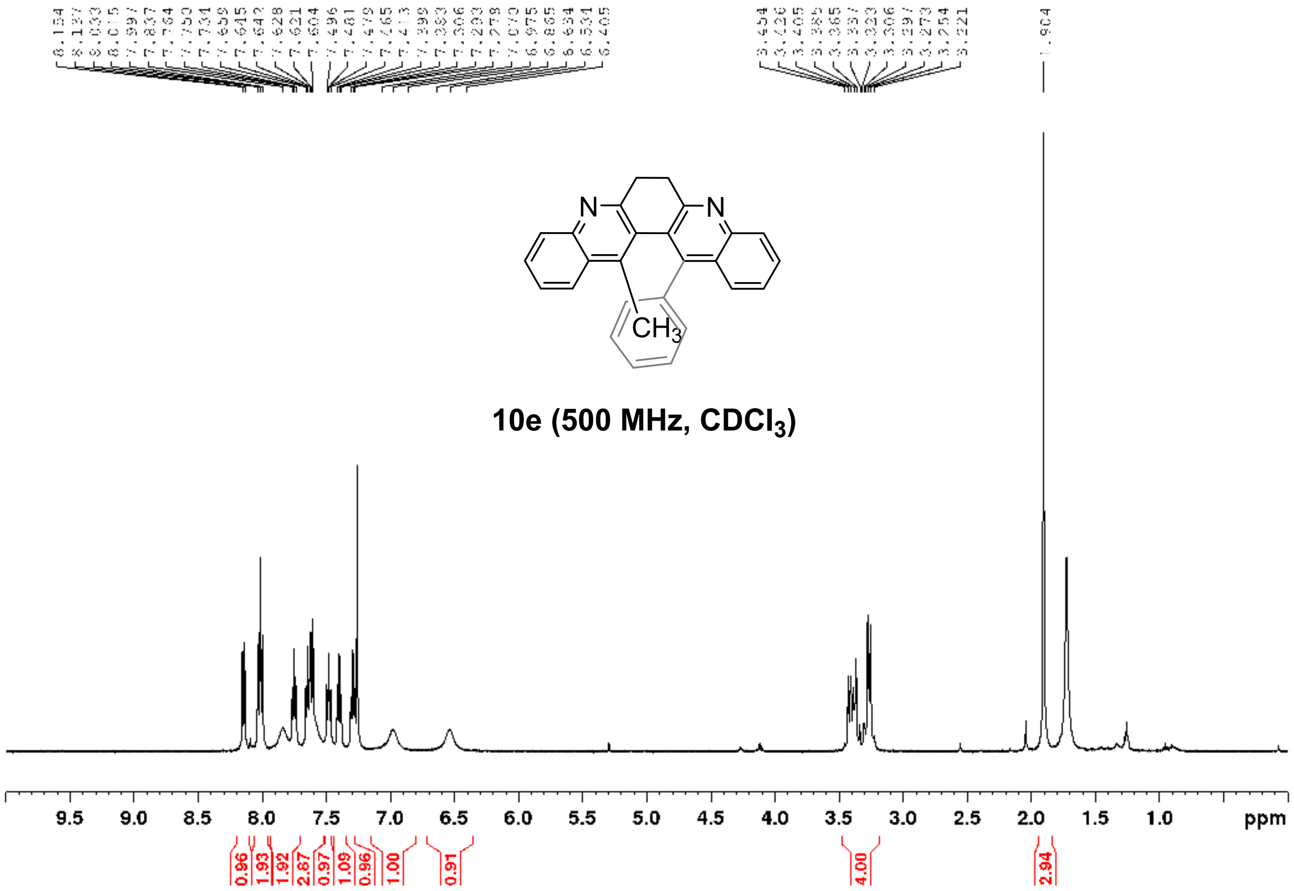

10e (500 MHz, CDCl$_3$)

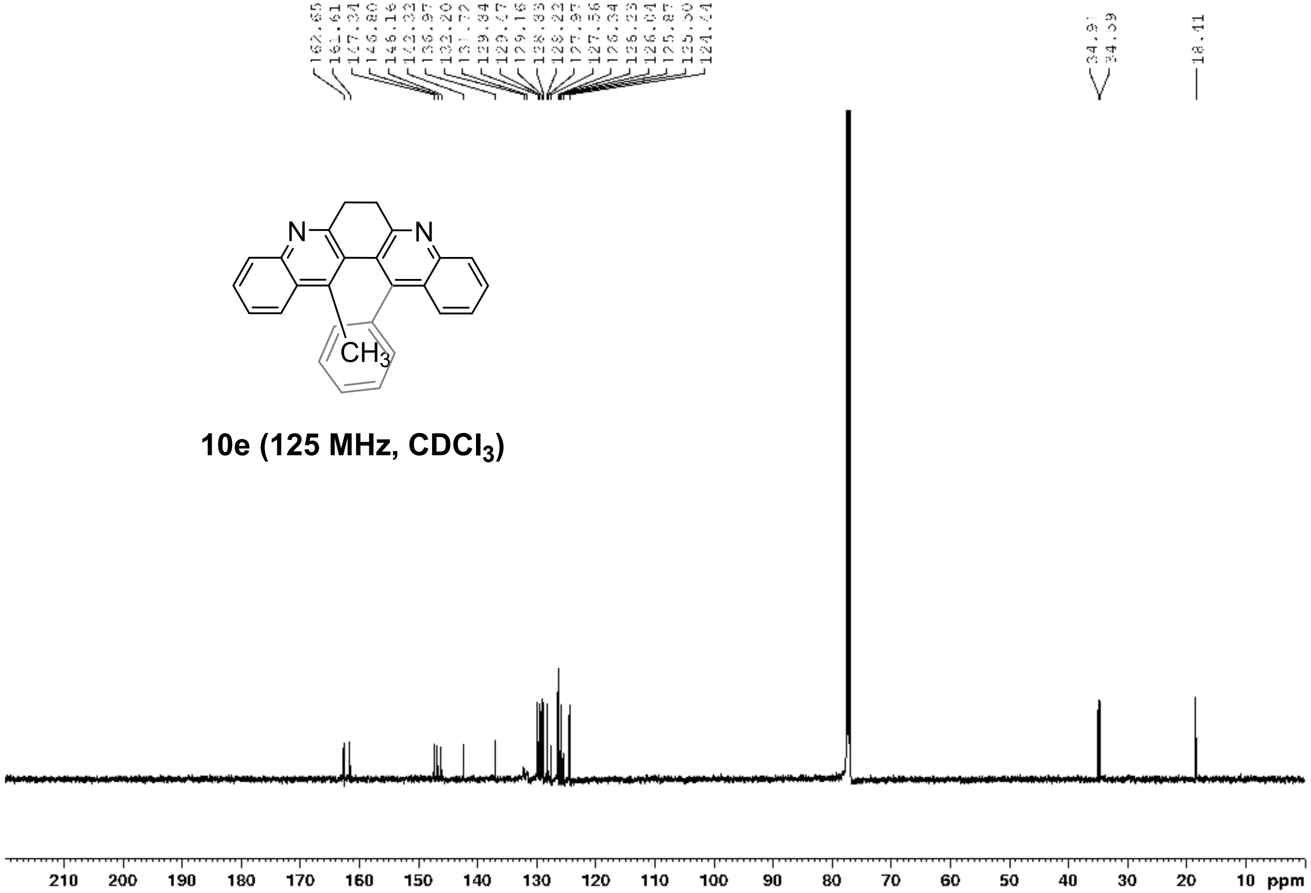

10e (125 MHz, $CDCl_3$)

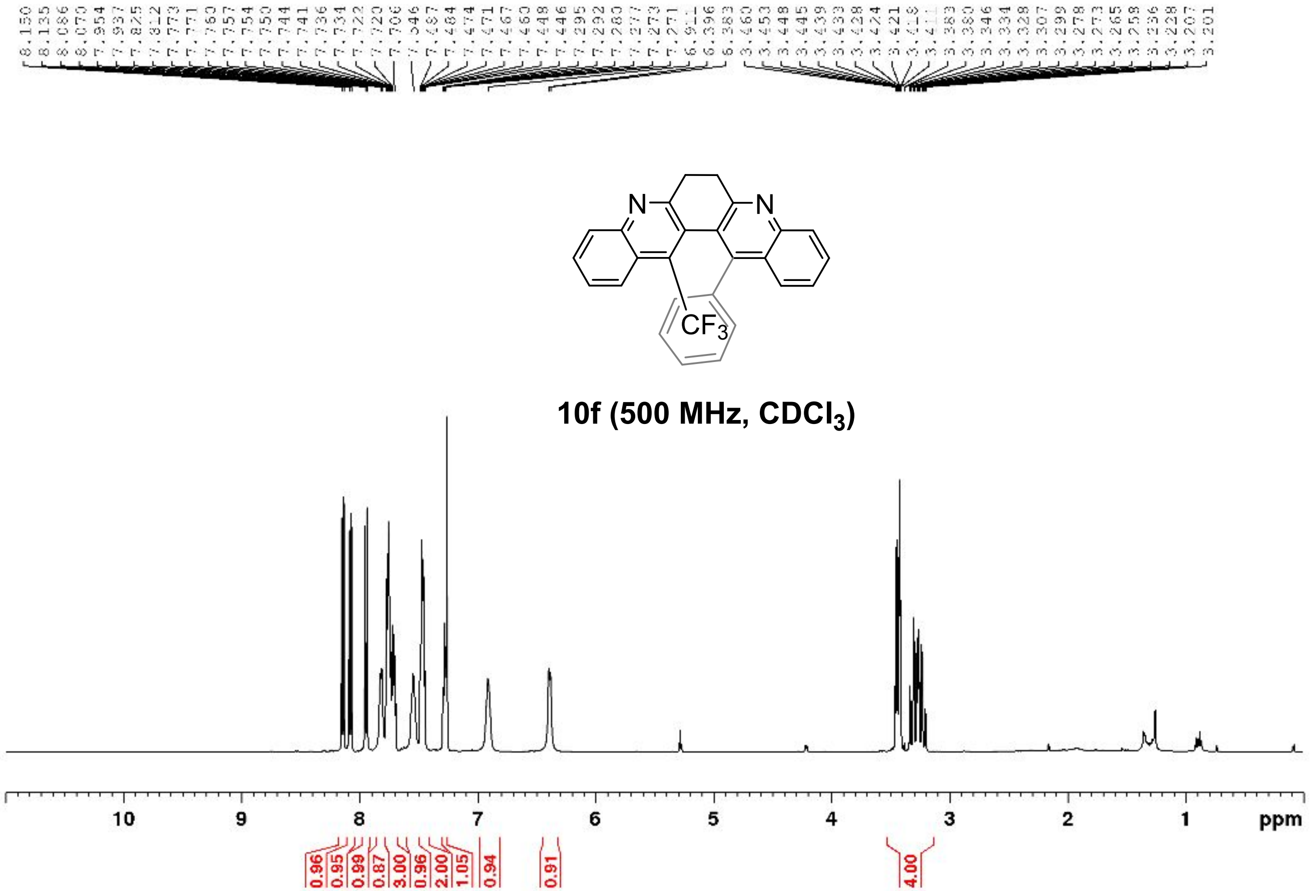

10f (500 MHz, $CDCl_3$)

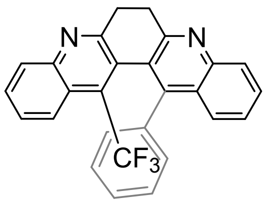

10f (125 MHz, $CDCl_3$)

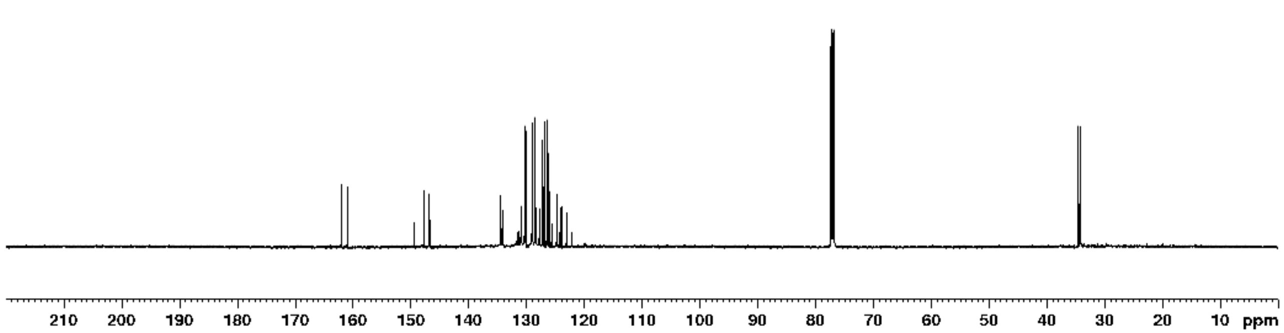

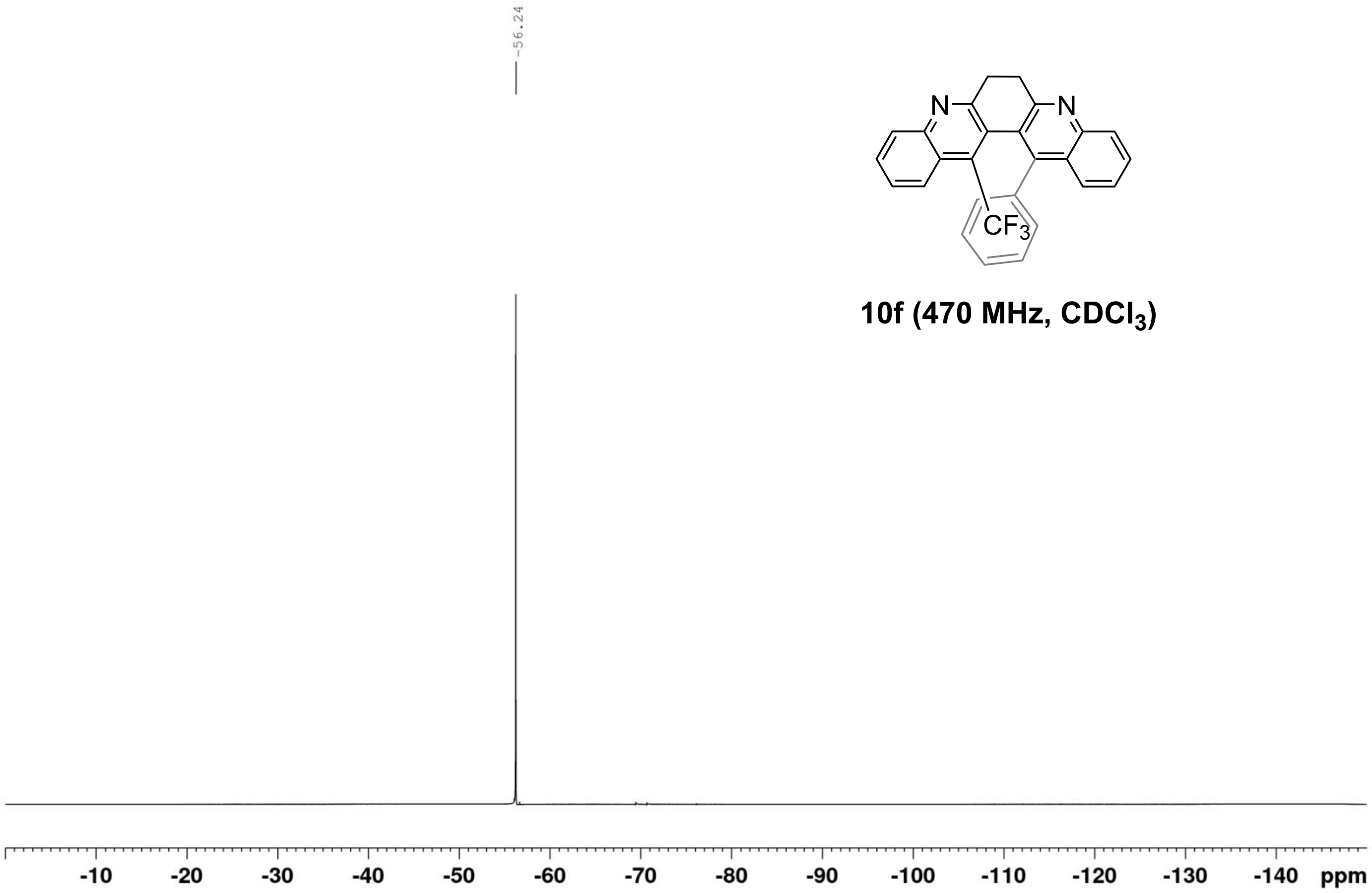

10f (470 MHz, $CDCl_3$)

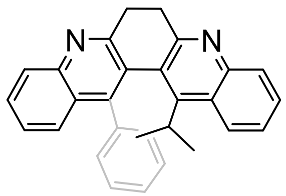

10g (500 MHz, $CDCl_3$)

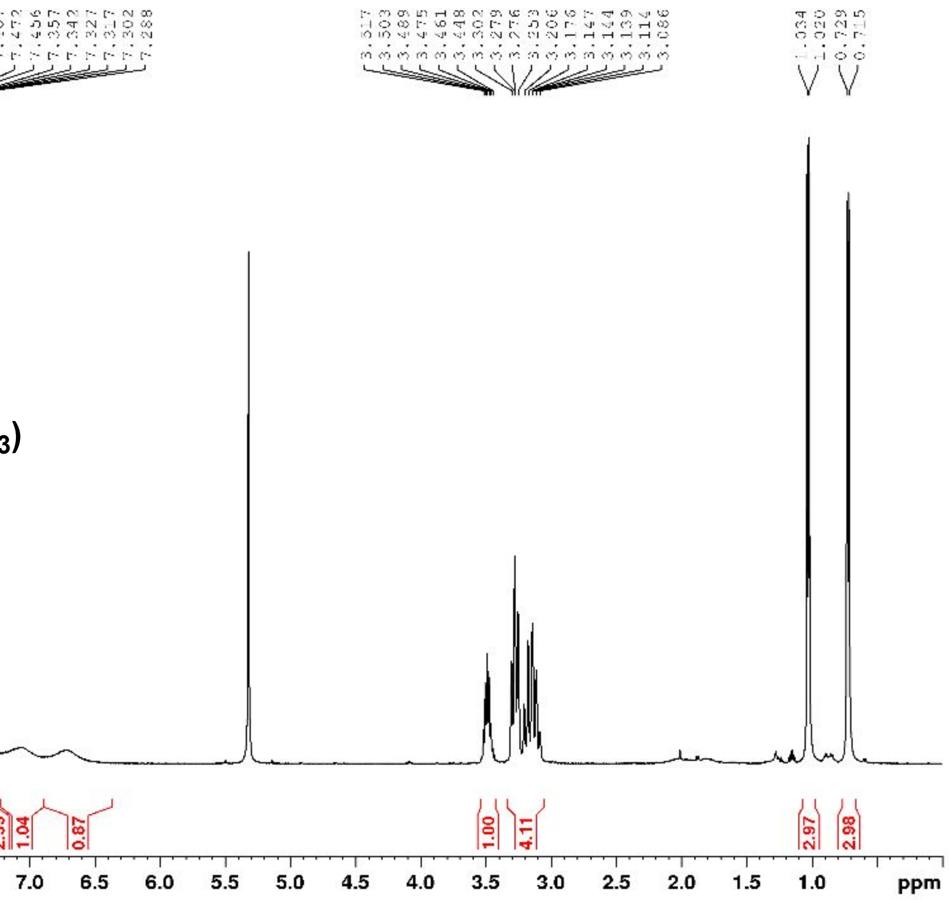

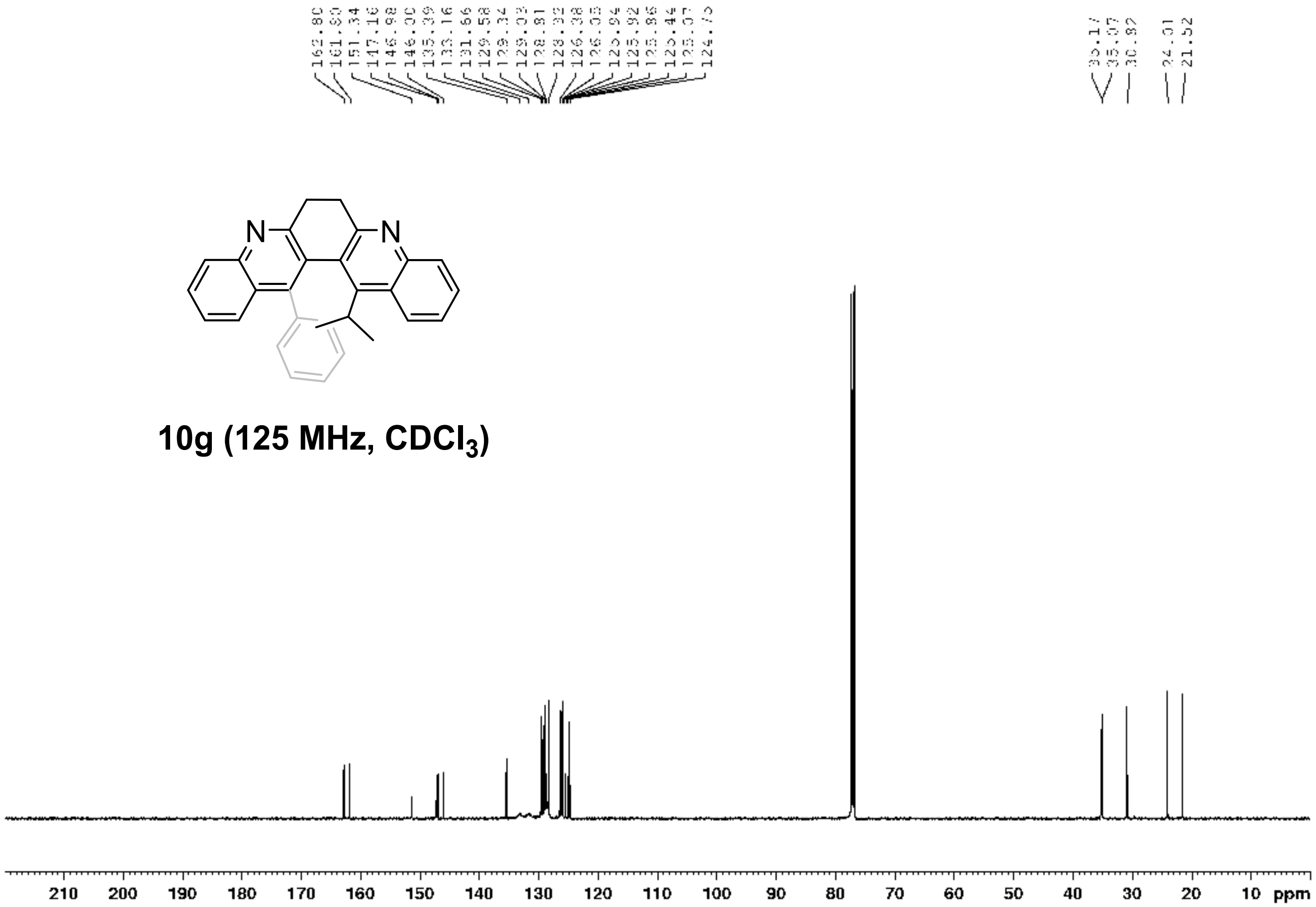

10g (125 MHz, $CDCl_3$)

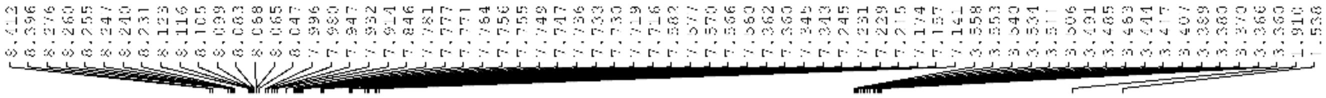

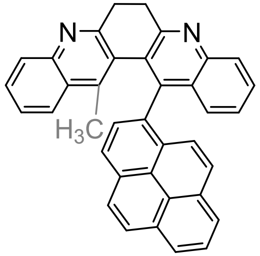

10h (500 MHz, $CDCl_3$)

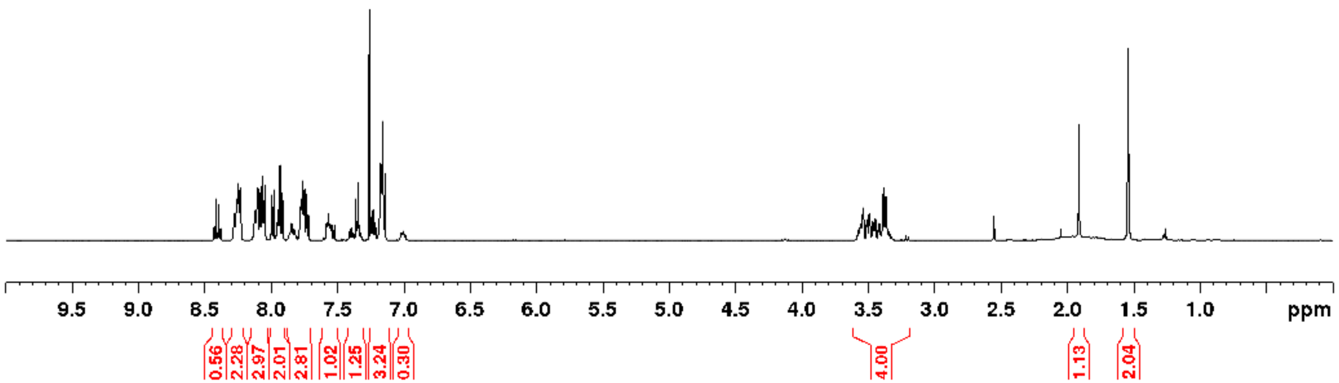

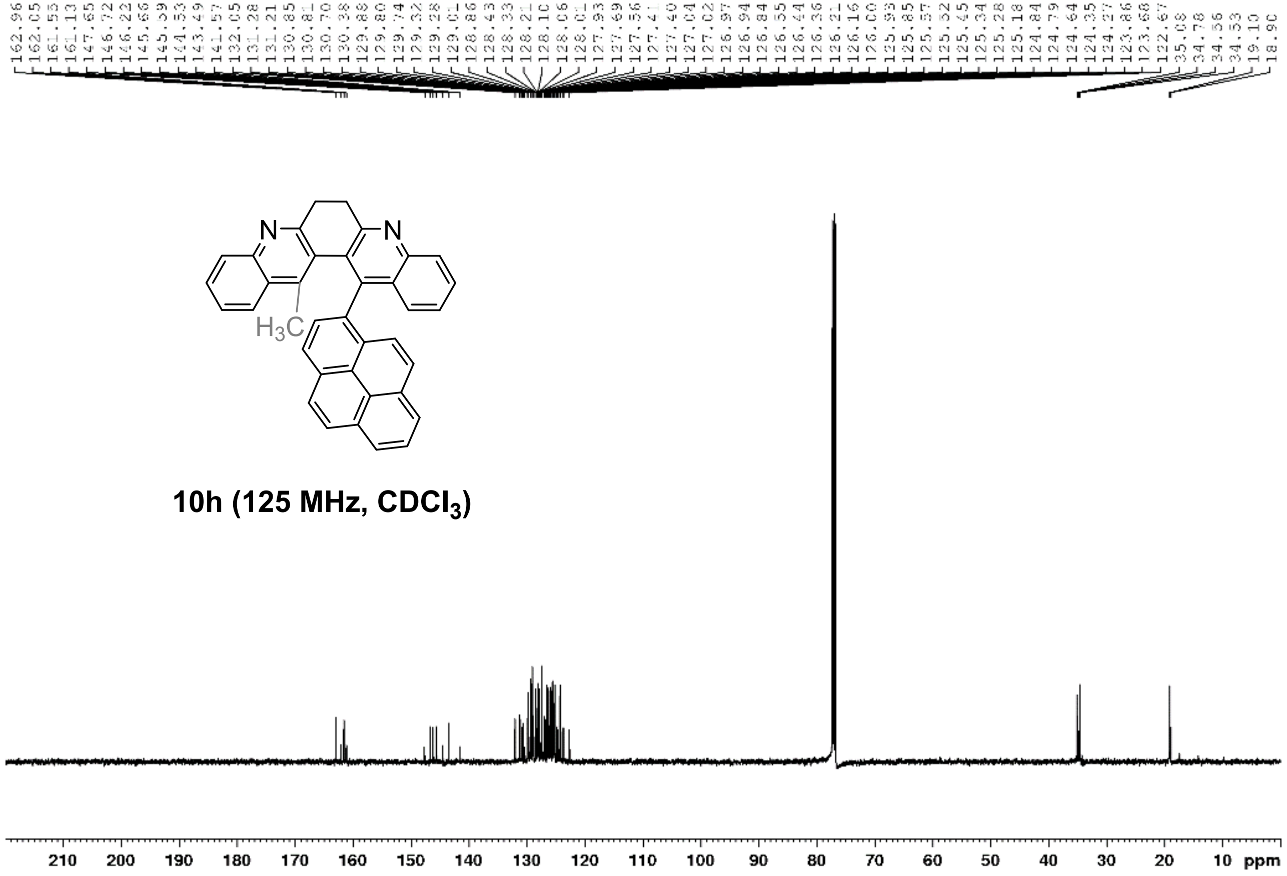

10h (125 MHz, $CDCl_3$)

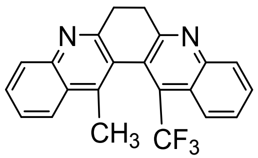

10i (500 MHz, CDCl$_3$)

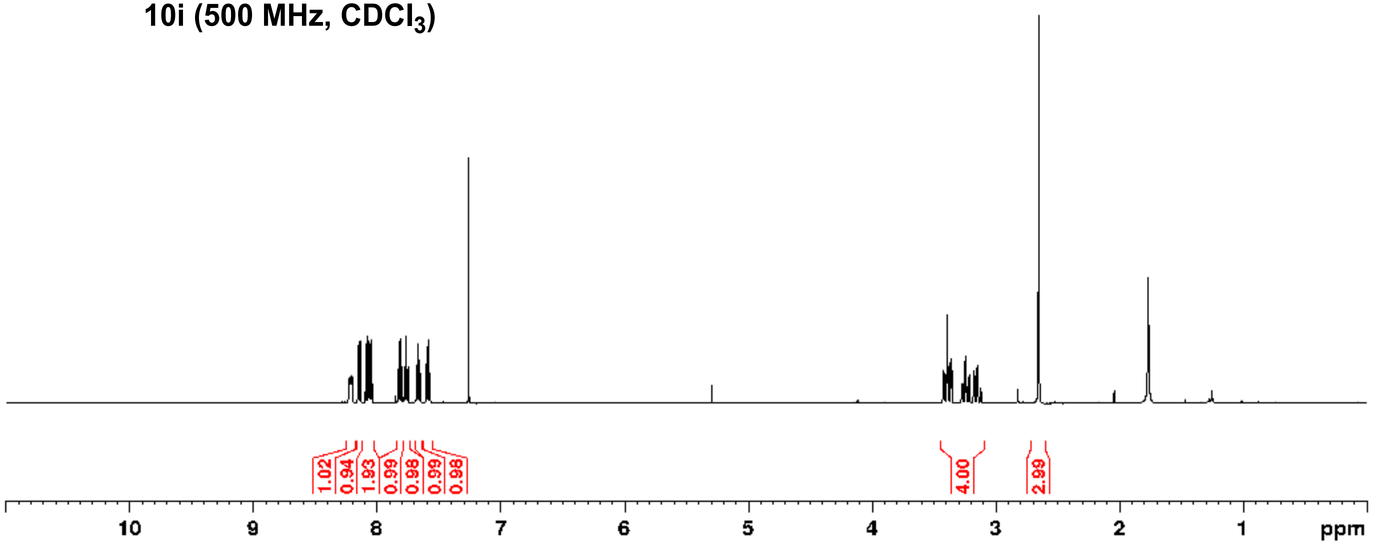

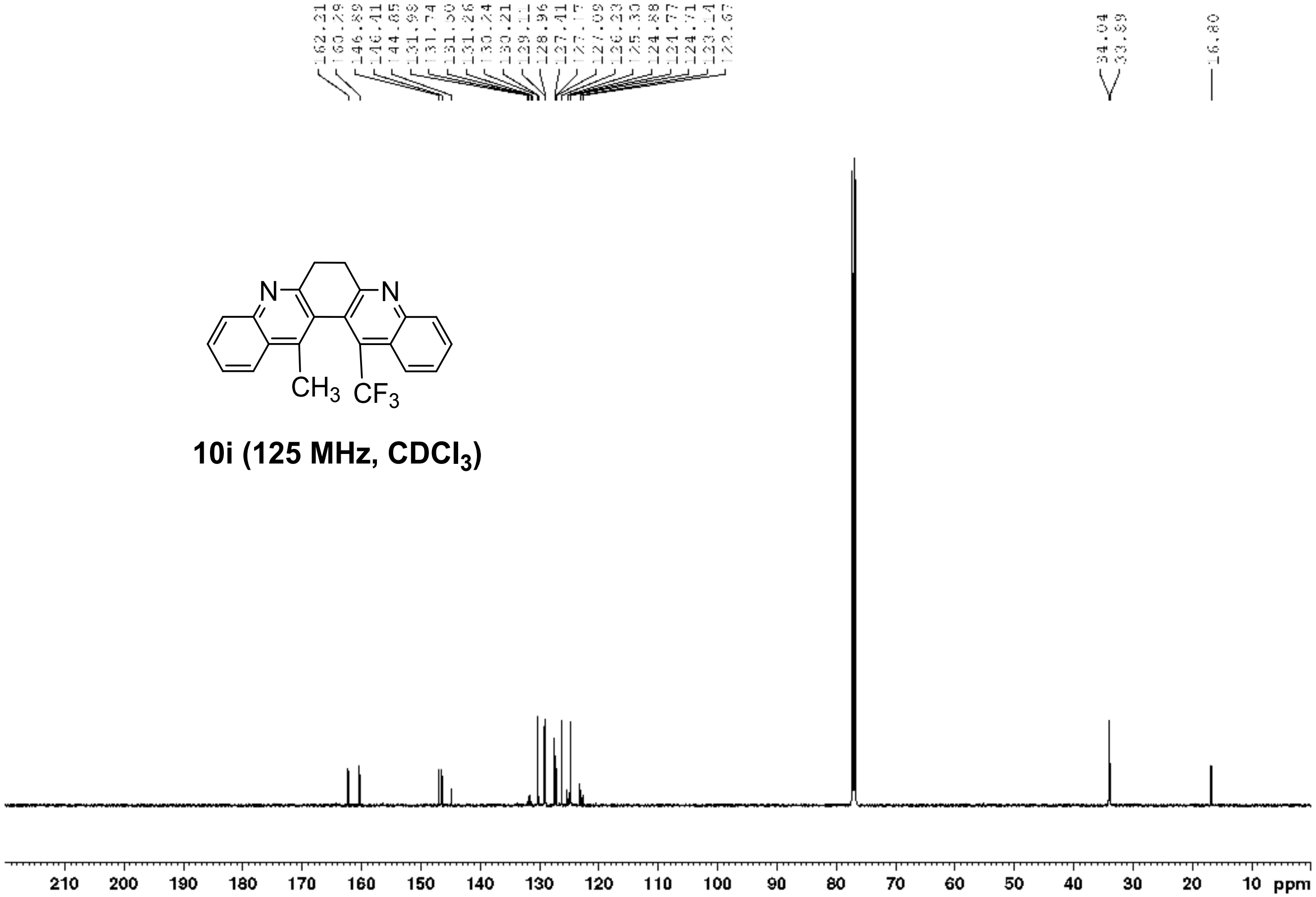

10i (125 MHz, $CDCl_3$)

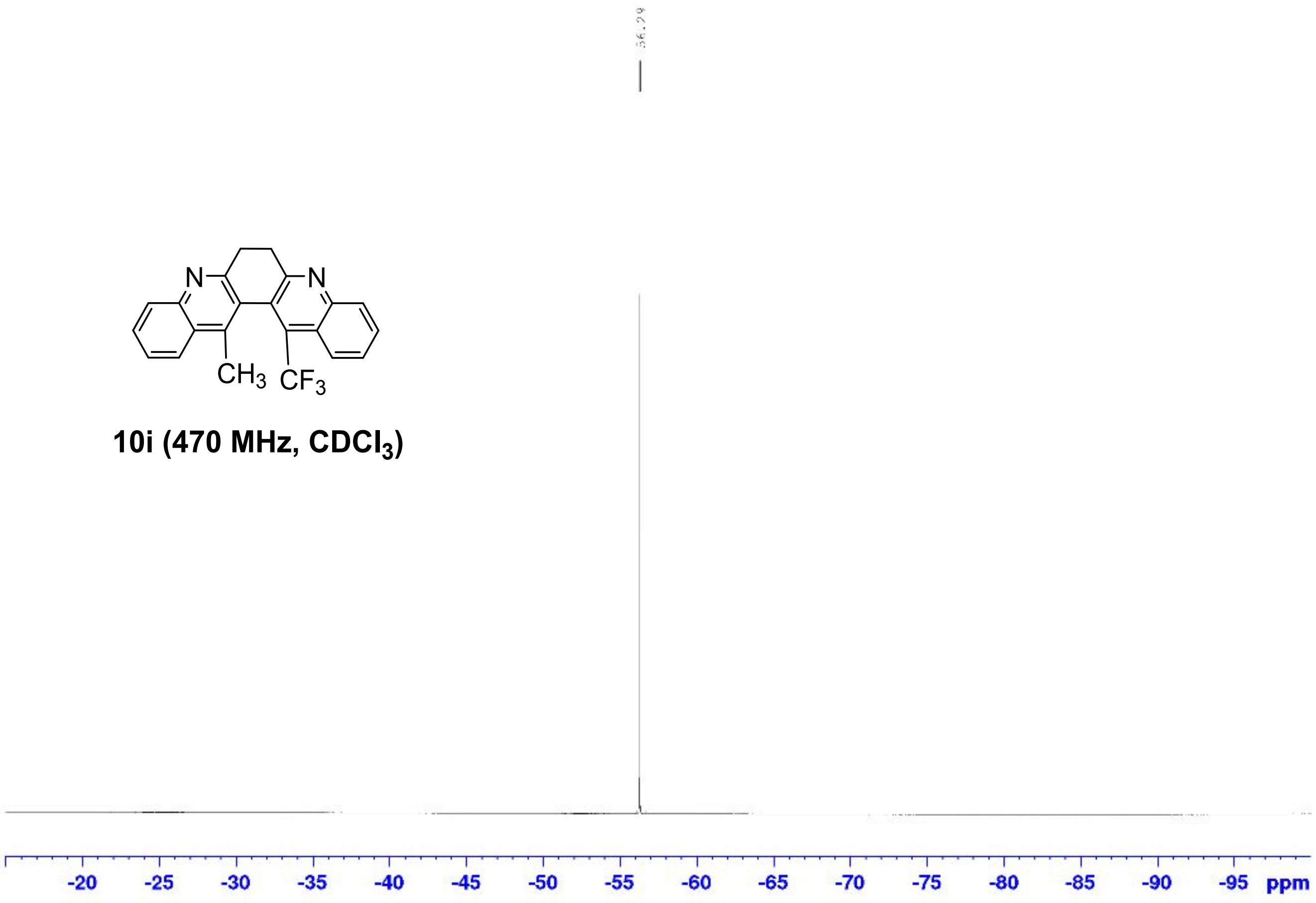

10i (470 MHz, $CDCl_3$)

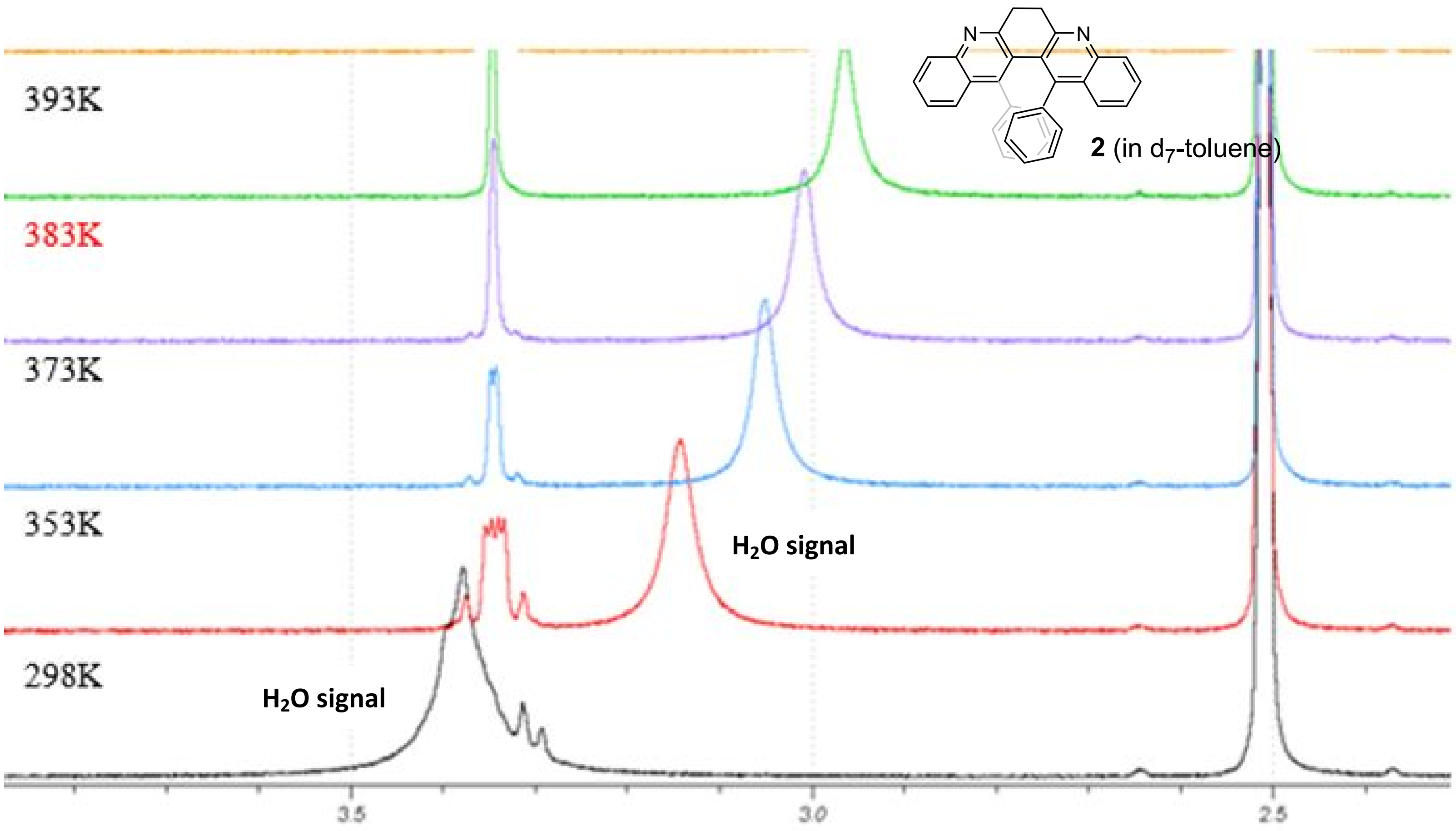

Figure S2-1: Variable temperature experiment to investigate the interconversion between enantiomers of **2**

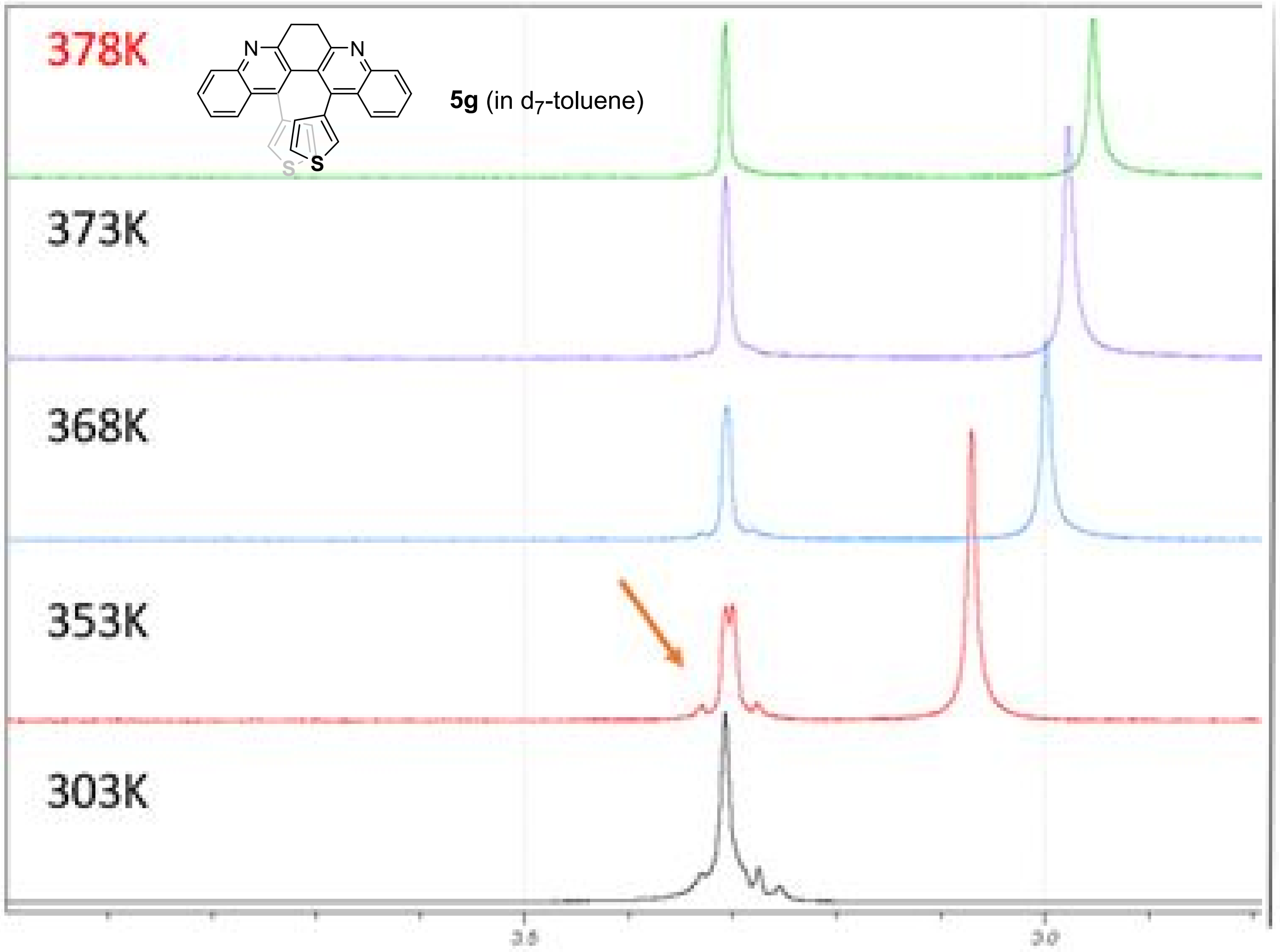

Figure S2-2: Variable temperature experiment to investigate the interconversion between enantiomers of **5g**

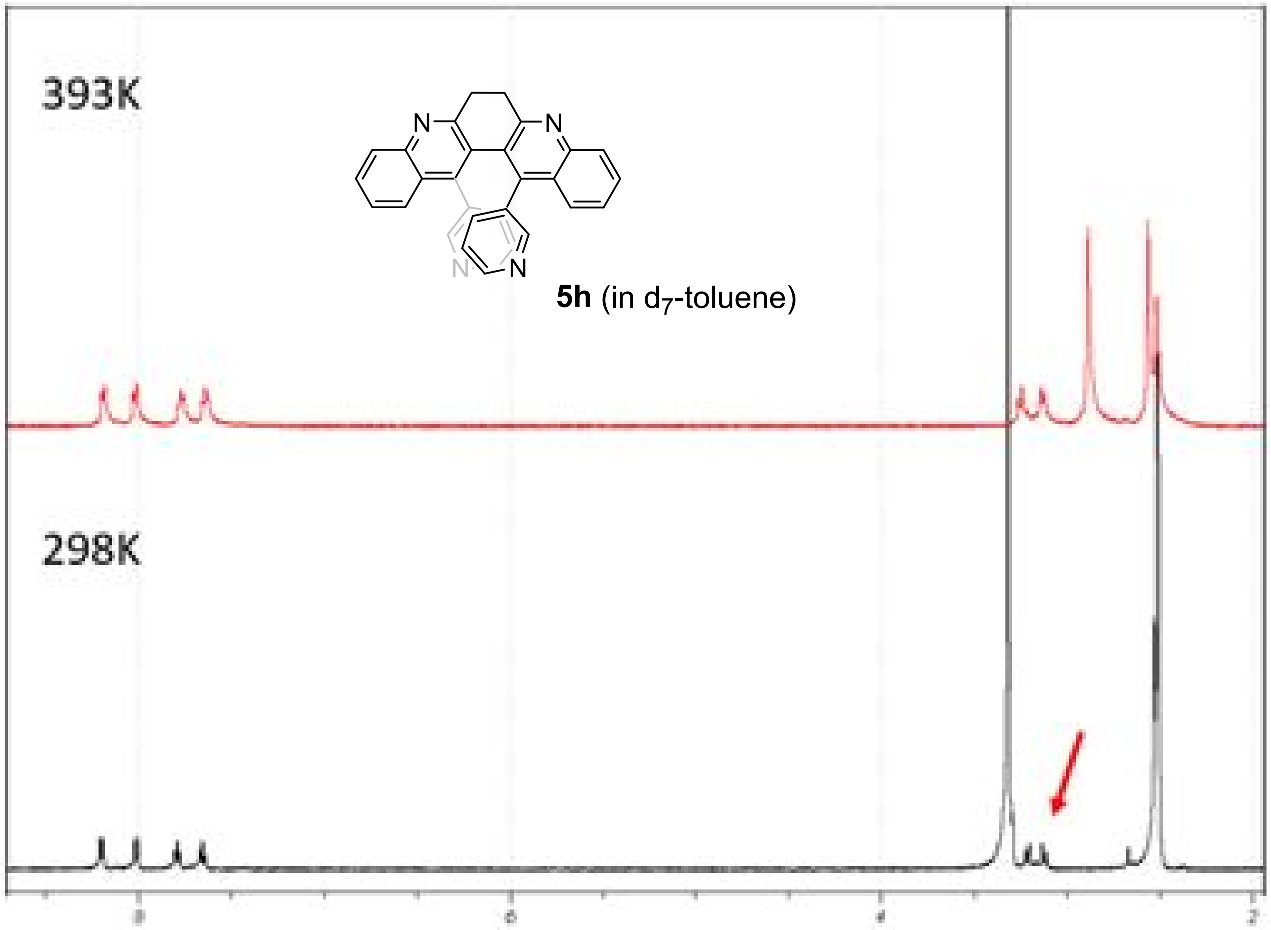

Figure S2-3: Variable temperature experiment to investigate the interconversion between enantiomers of **5h**

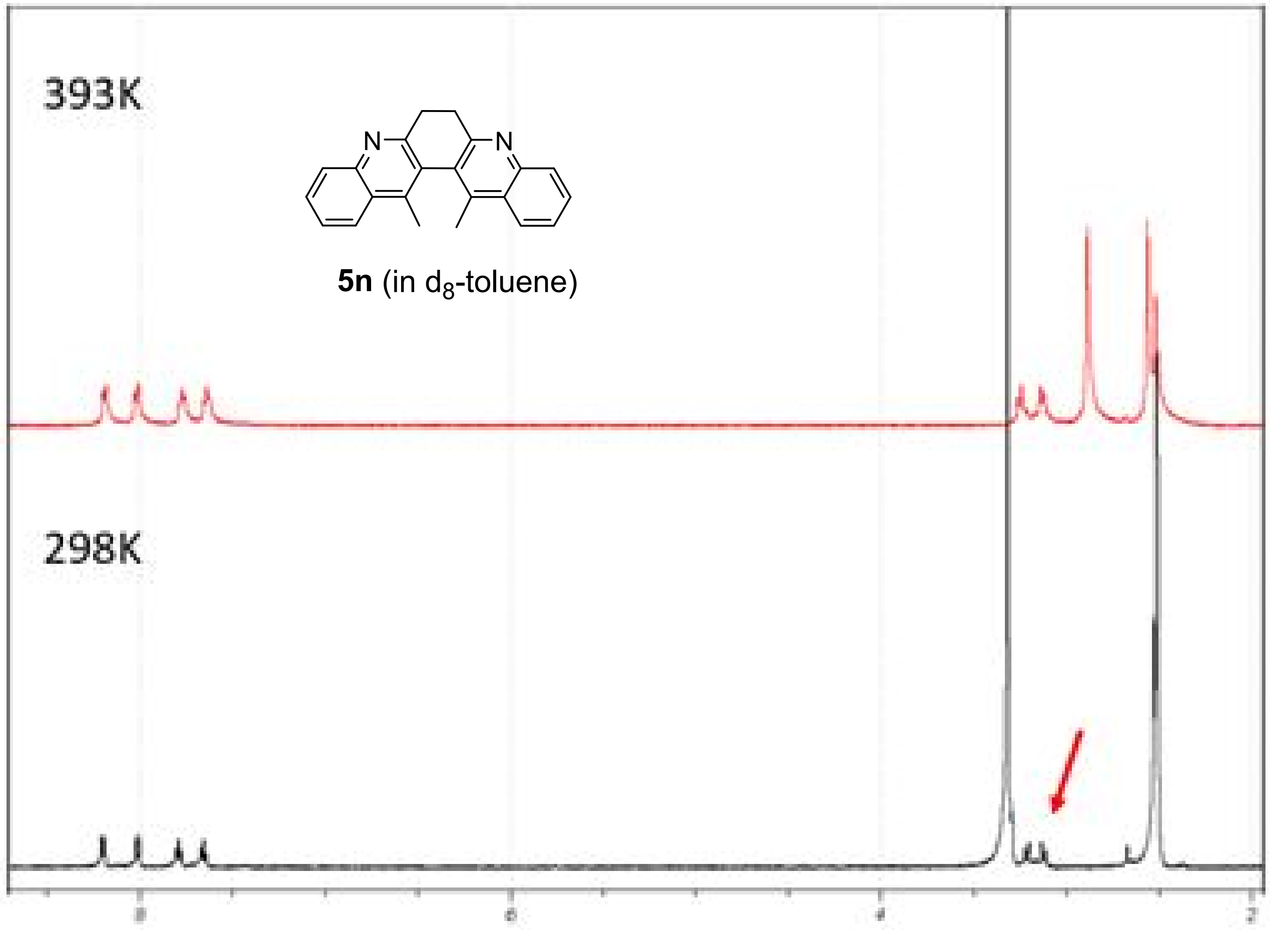

Figure S2-4: Variable temperature experiment to investigate the interconversion between enantiomers of **5n**

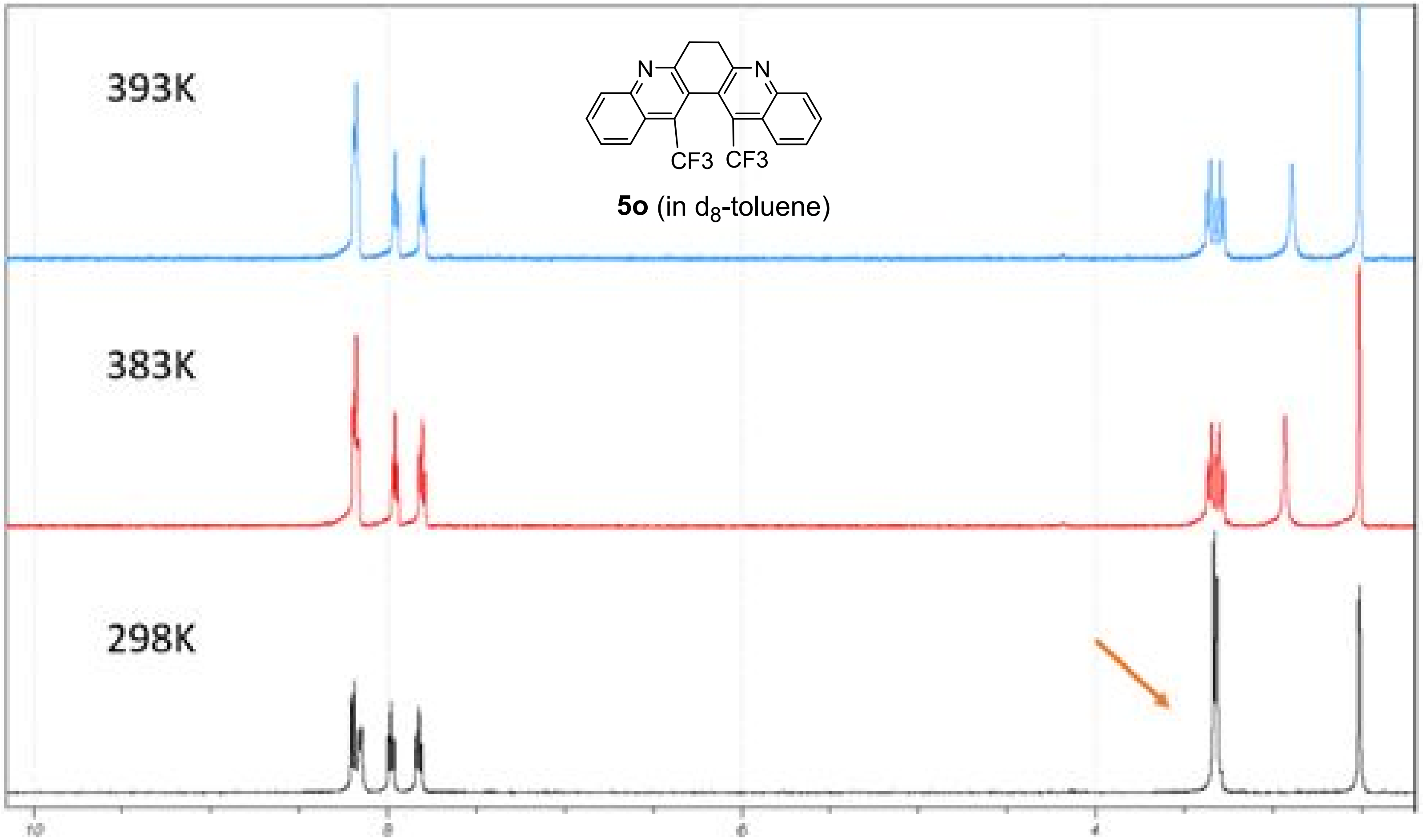

Figure S2-5: Variable temperature experiment to investigate the interconversion between enantiomers of **5o**

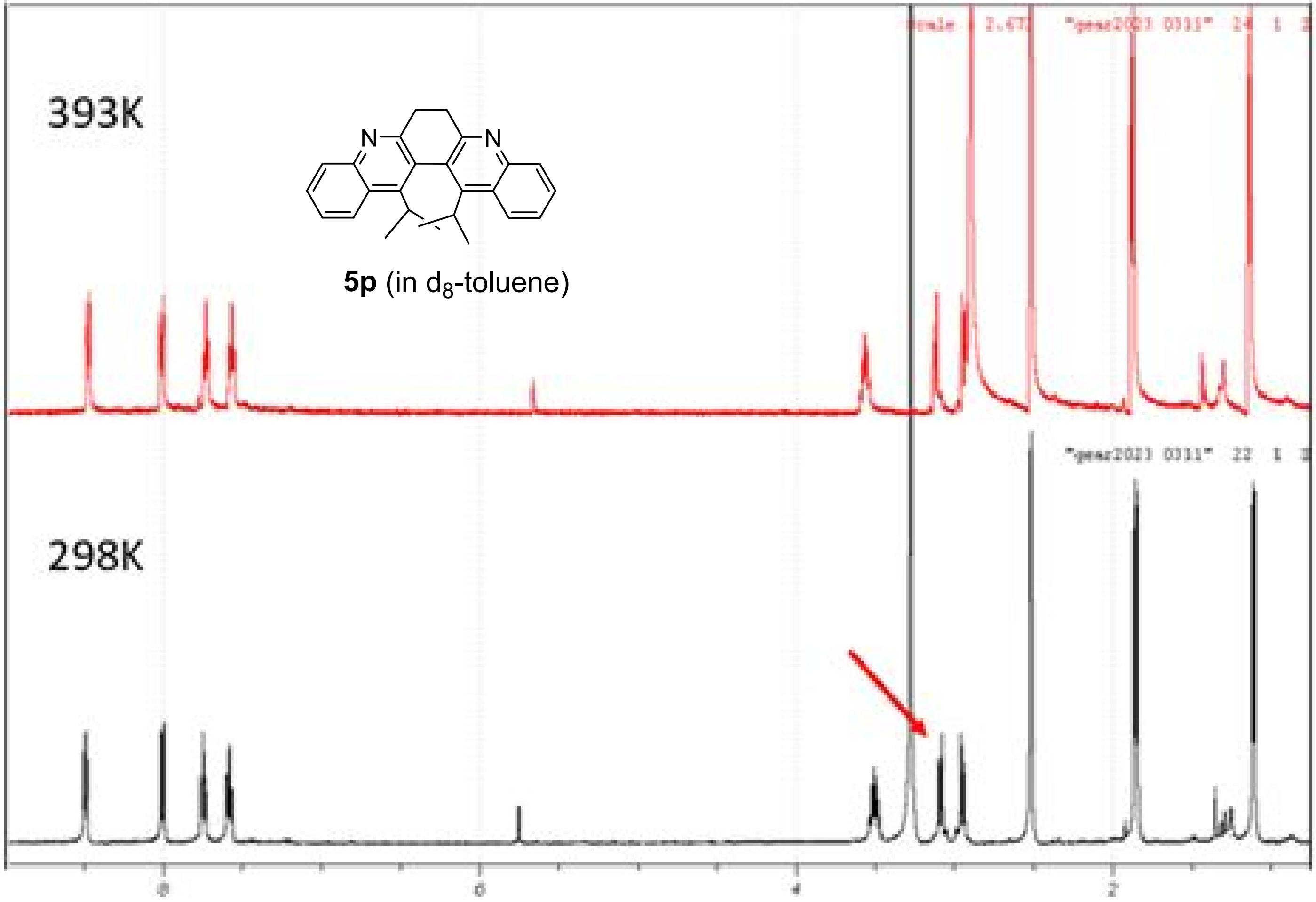

Figure 2S-6: Variable temperature experiment to investigate the interconversion between enantiomers of **5p**

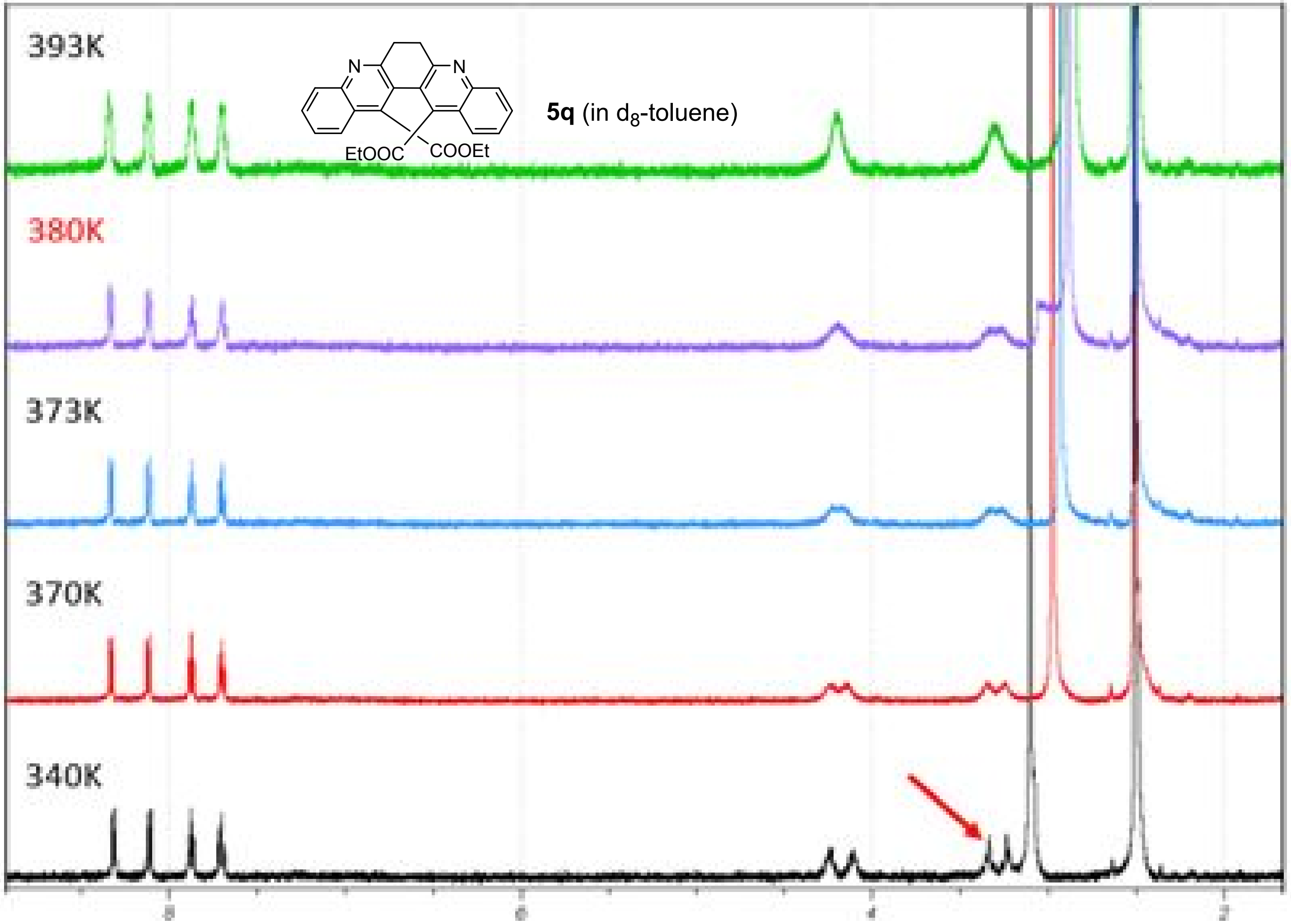

Figure S2-7: Variable temperature experiment to investigate the interconversion between enantiomers of **5q**

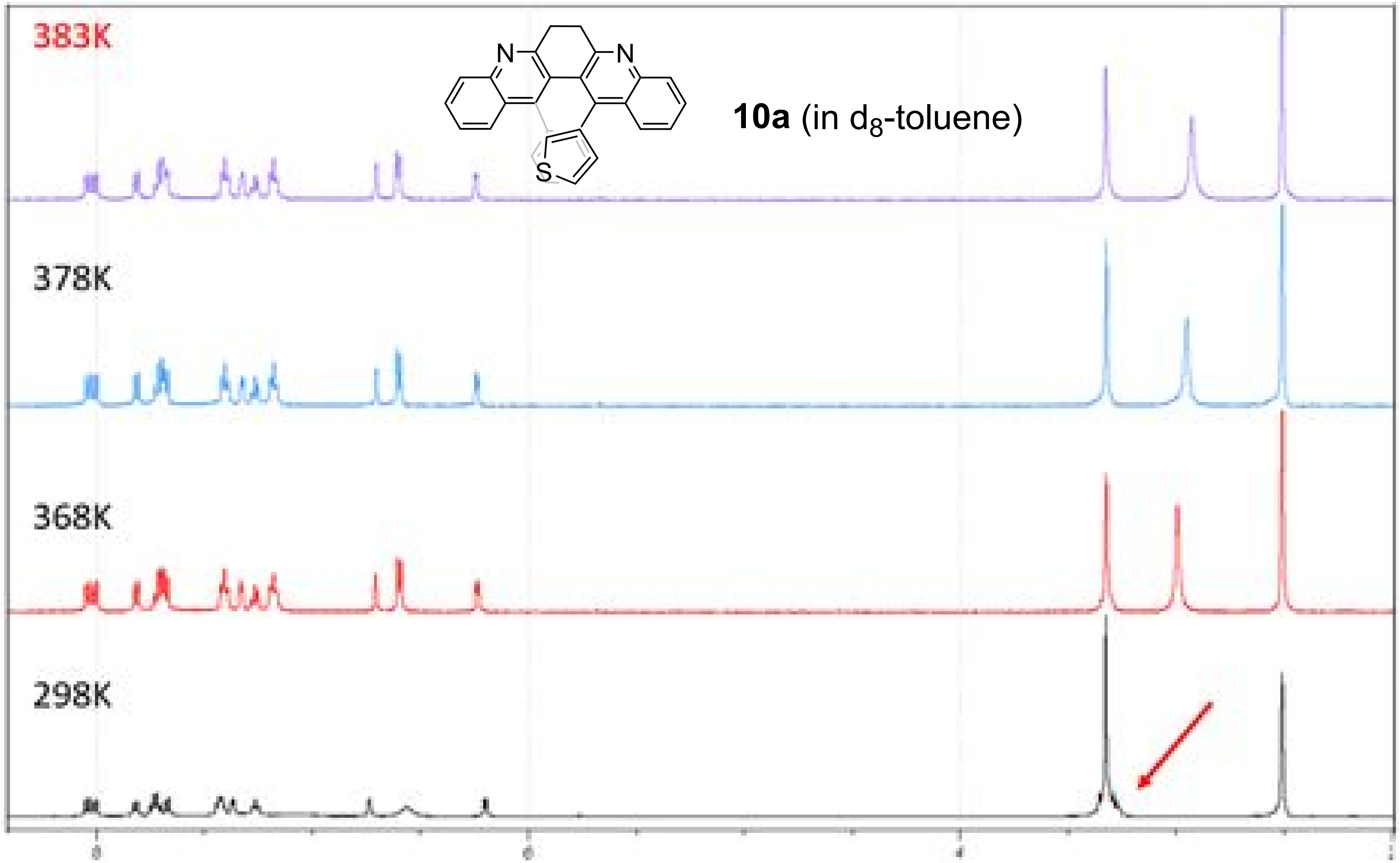

Figure S2-8: Variable temperature experiment to investigate the interconversion between enantiomers of **10a**

The activation energies were calculated with Eyring equation (1) and the relation between coalescence temperatures and chemical shift differences between the coalescing signals (2).

$$K = k_b T/\hbar \, e^{-\Delta G^*/RT} \qquad (1)$$

$$K = \pi\Delta\nu / \sqrt{2} \qquad (2)$$

K = rate constant, k_b= boltzmann constant, T = temperature, h = planck constant
R= gas constant, ΔG^*= activation free energy, $\Delta\nu$ = chemical shift difference between coalescing signals

$$\Delta G^* = RT\,[\ln(k_b T/\hbar) - \ln(\pi\Delta\nu / \sqrt{2})] \qquad (3)$$

	Coalescence temperature (k)	Racemization barrier (kcal/mol)		Coalescence temperature (k)	Racemization barrier (kcal/mol)
**2**	383	18.37	**5o**	>393	>18.6
**5g**	368	18.18	**5p**	>393	>20.5
**5h**	>393	>18.97	**5q**	380	18.57
**5n**	>393	>18.8	**10a**	378	18.50

Table S2-1: Racemization barriers and coalescence temperatures for selected bay region substituted dibenzophenanthroline derivatives

All data in table 1 were taken in d_8-toluene. Some spectral analyses were ambiguous because the water signal (in d_{8}-toluene) often coincides with the bridging ethylene signals near room temperature. This problem is particularly tricky in compound **10a**. The coalescence temperature (378 K) was roughly determined based on the width of overlapped signals near the bottom of the peak.

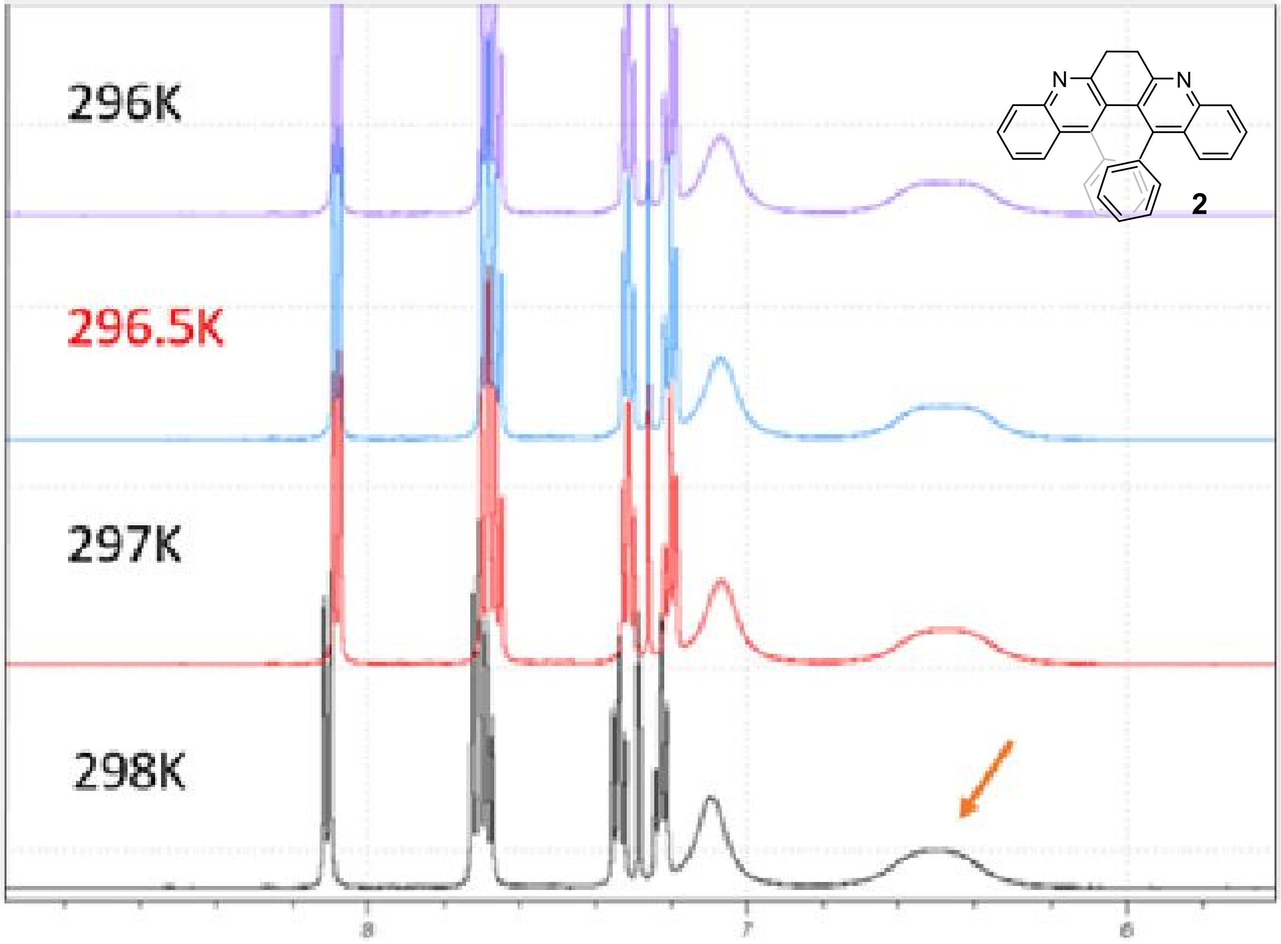

Figure S2-9: Variable temperature experiment to investigate the rotation of phenyl groups in **2**

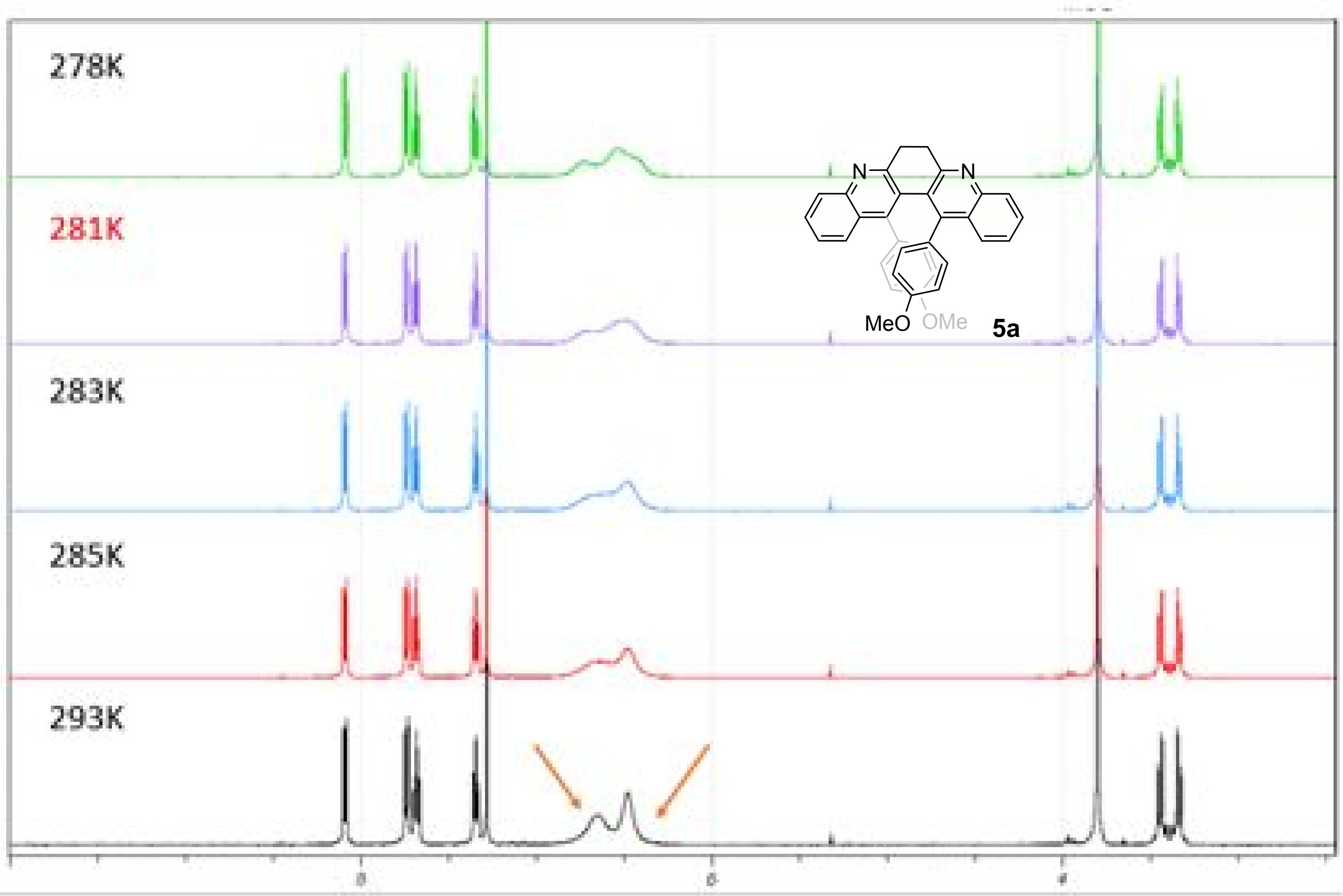

Figure S2-10: Variable temperature experiment to investigate the rotation of phenyl groups in **5a**

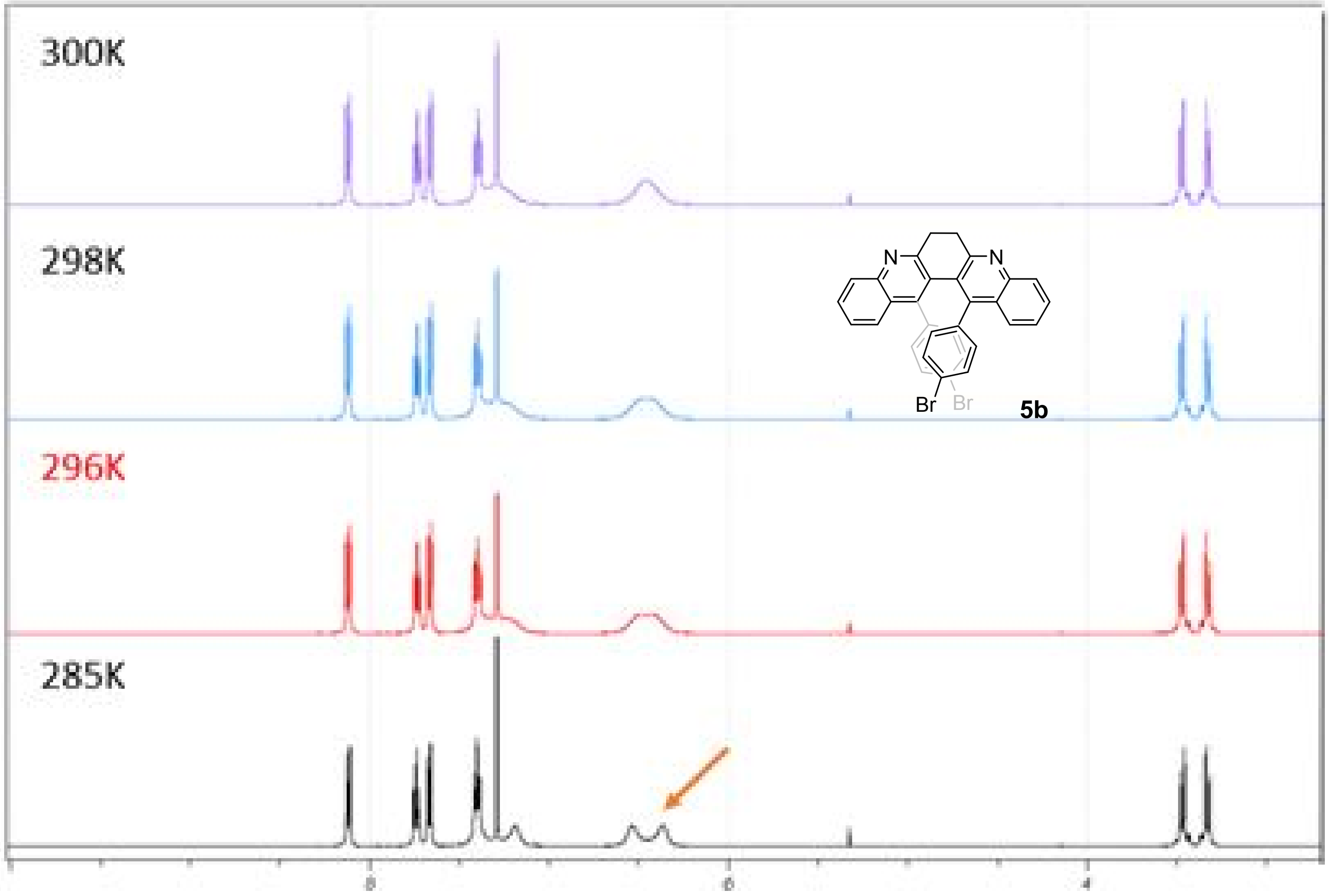

Figure S2-11: Variable temperature experiment to investigate the rotation of phenyl groups in **5b**

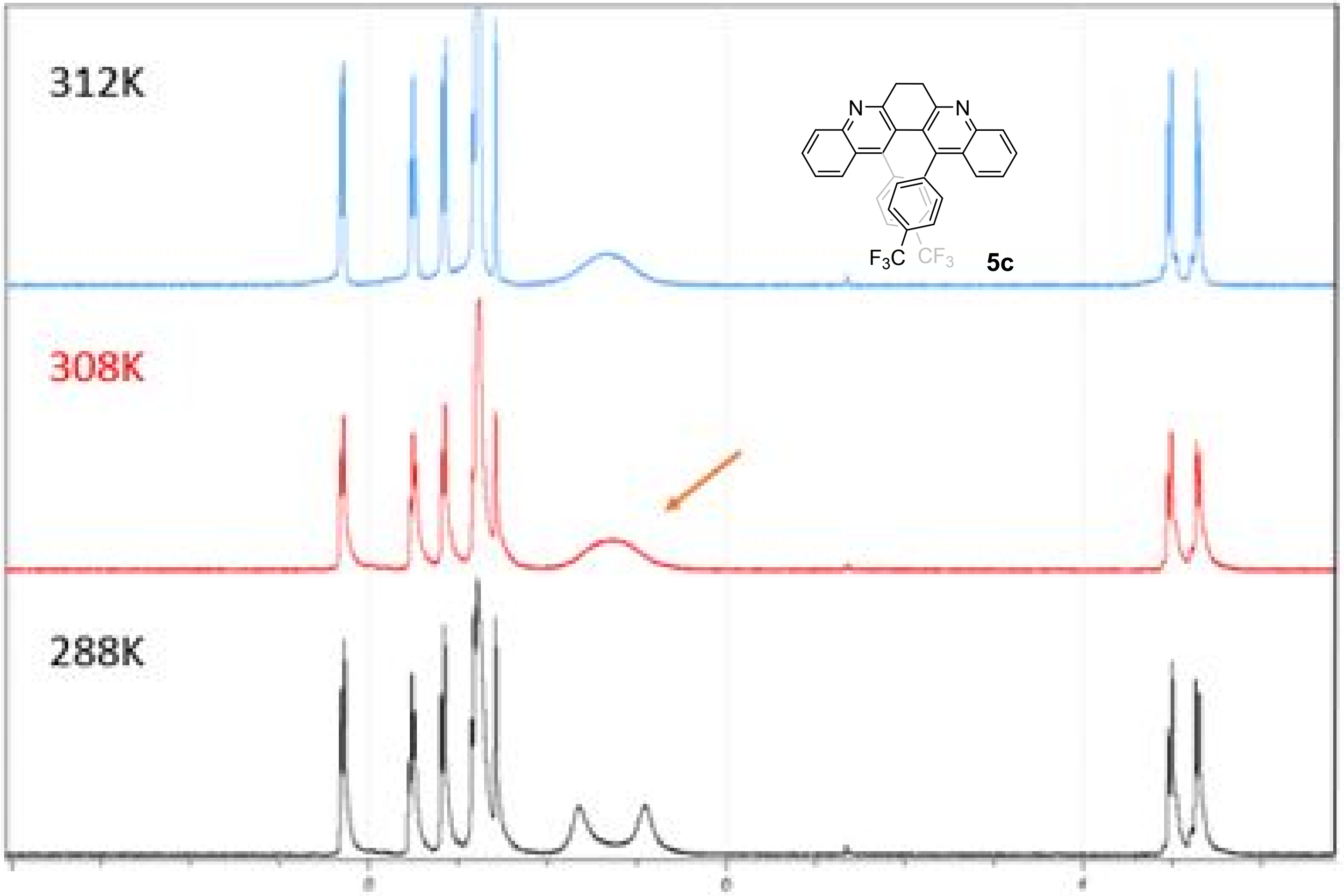

Figure S2-12: Variable temperature experiment to investigate the rotation of phenyl groups in **5c**

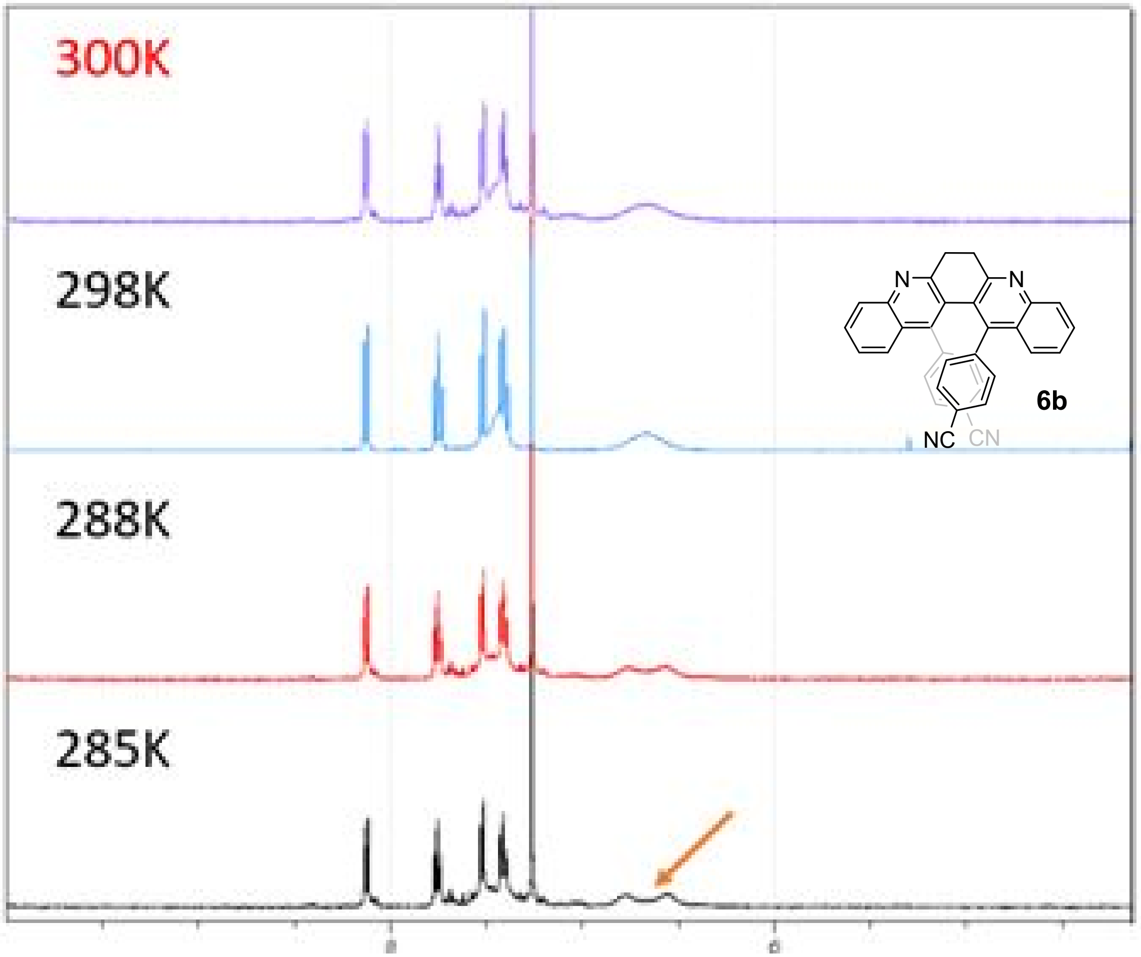

Figure S2-13: Variable temperature experiment to investigate the rotation of phenyl groups in **6b**

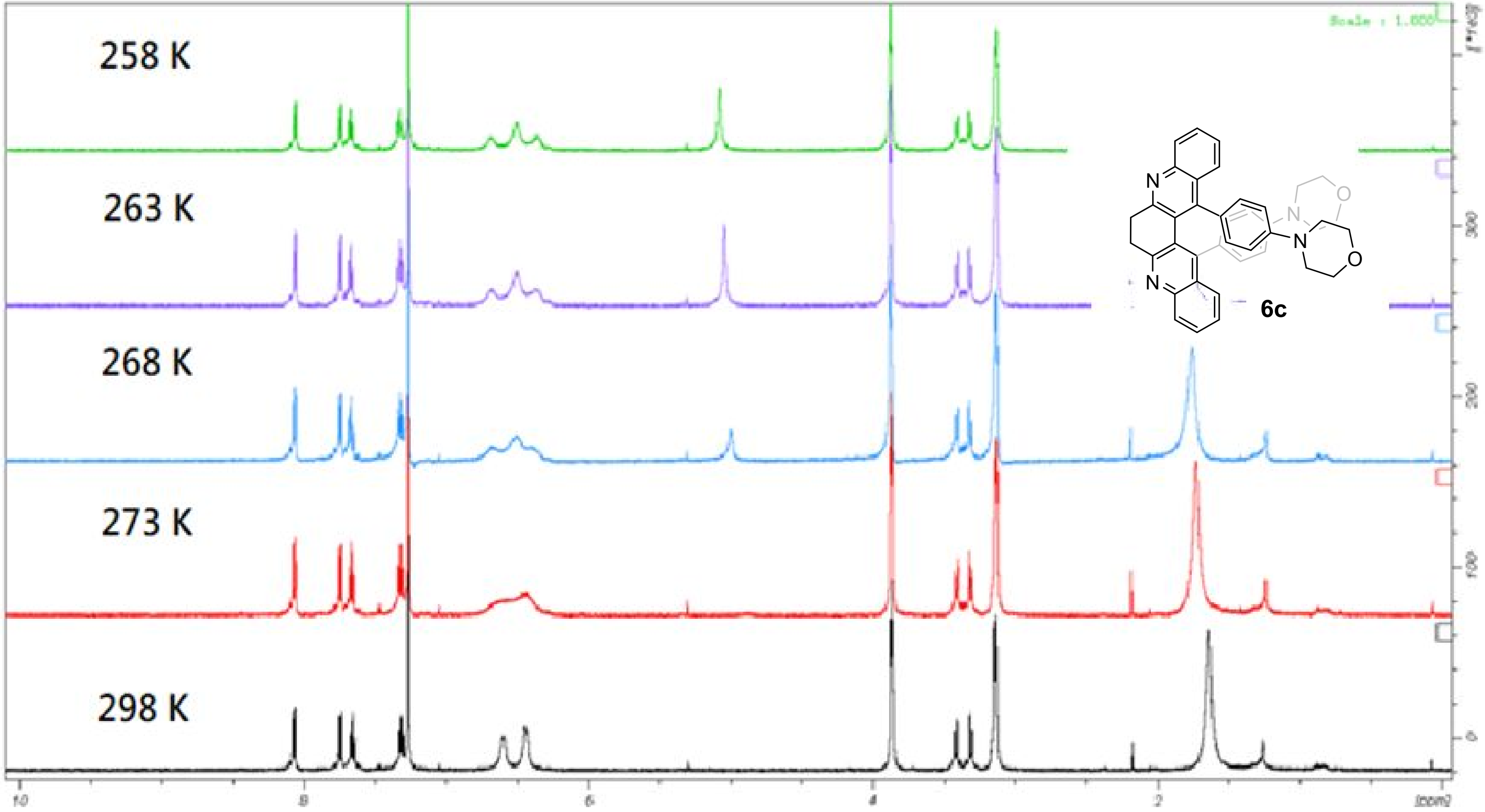

Figure S2-14: Variable temperature experiment to investigate the rotation of phenyl groups in **6c**

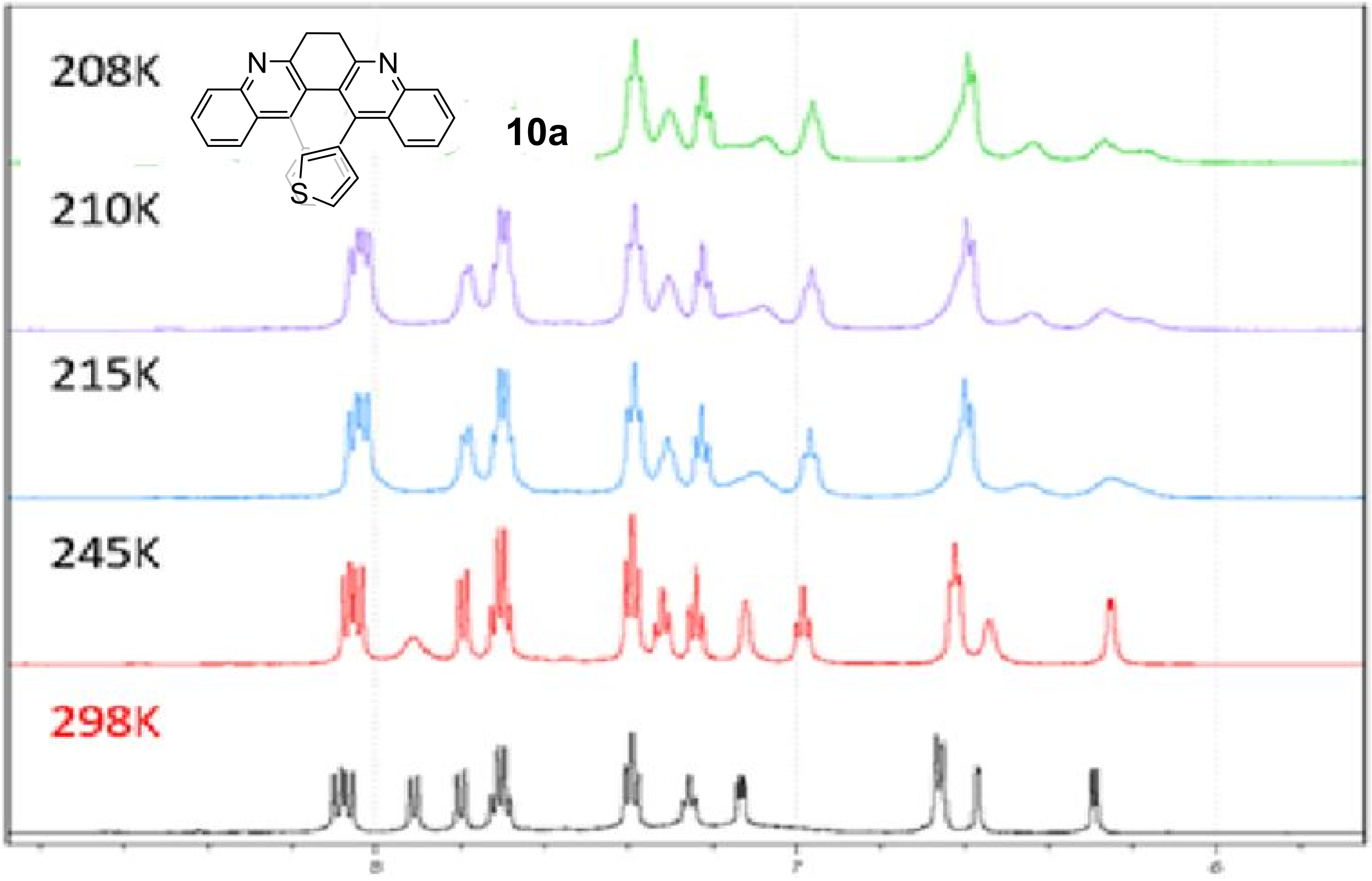

Figure S2-15: Variable temperature experiment to investigate the rotation of phenyl groups in **10a**

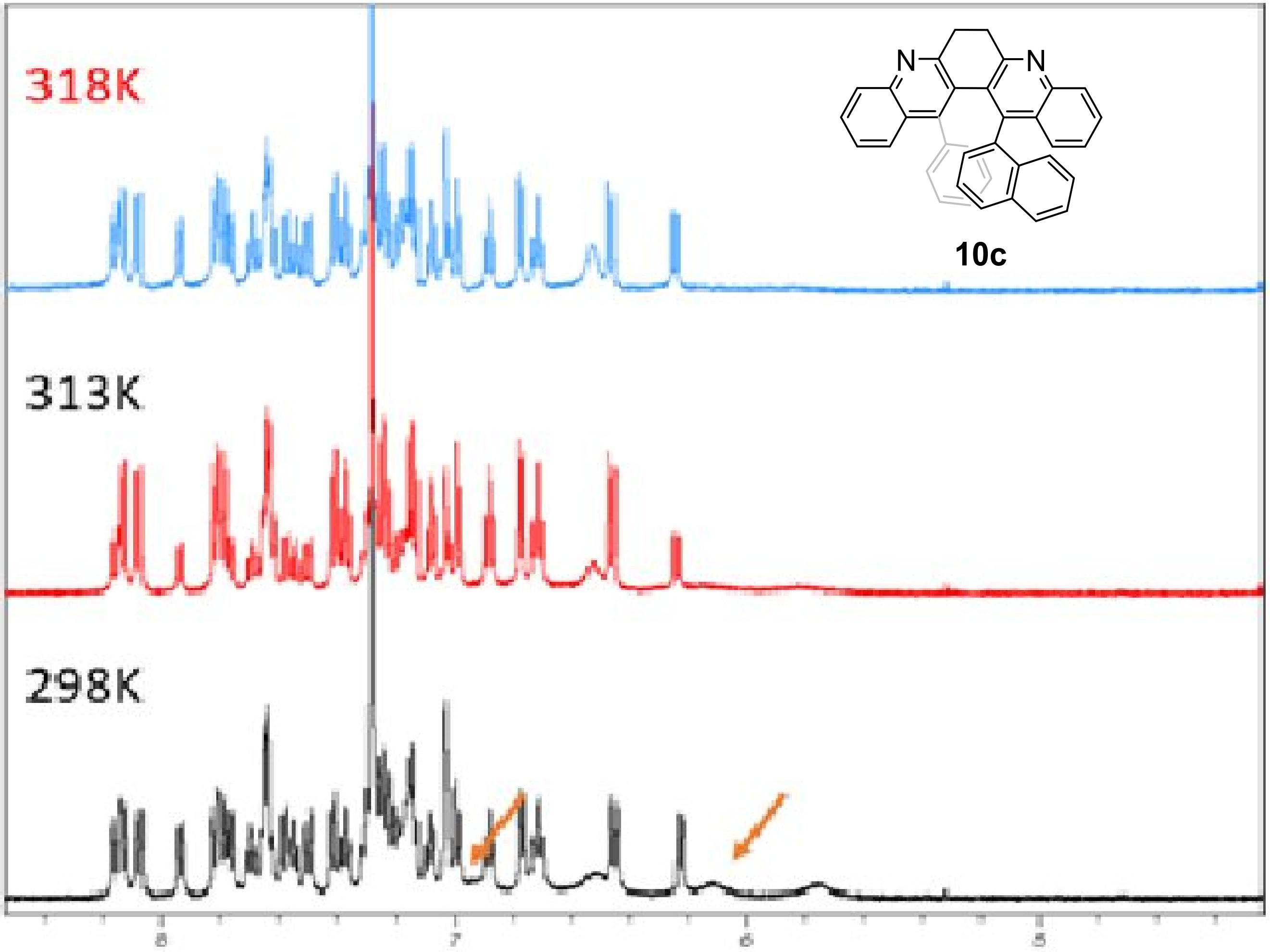

Figure S2-16: Variable temperature experiment to investigate the rotation of phenyl groups in **10c**

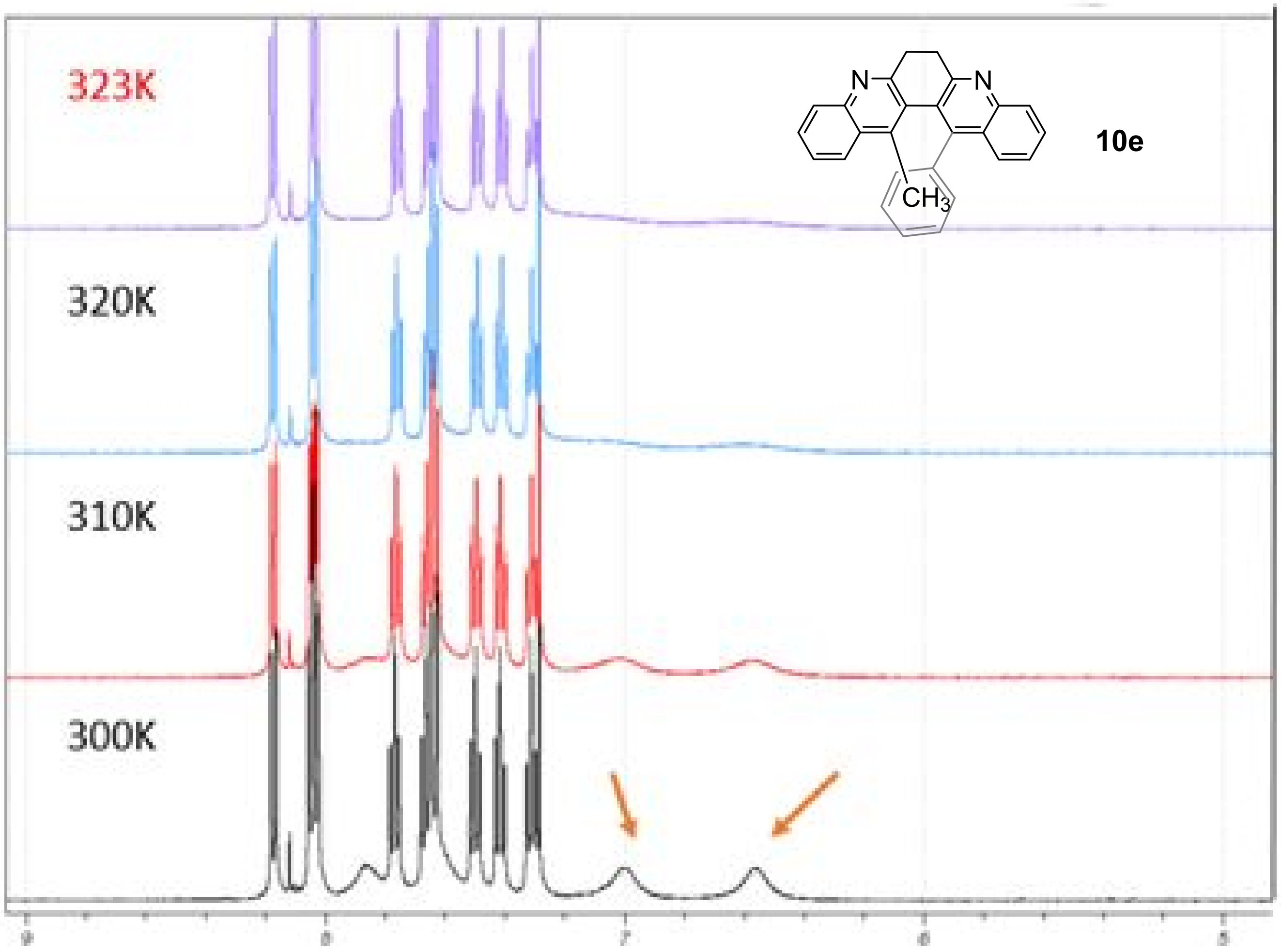

Figure S2-17: Variable temperature experiment to investigate the rotation of phenyl groups in **10e**

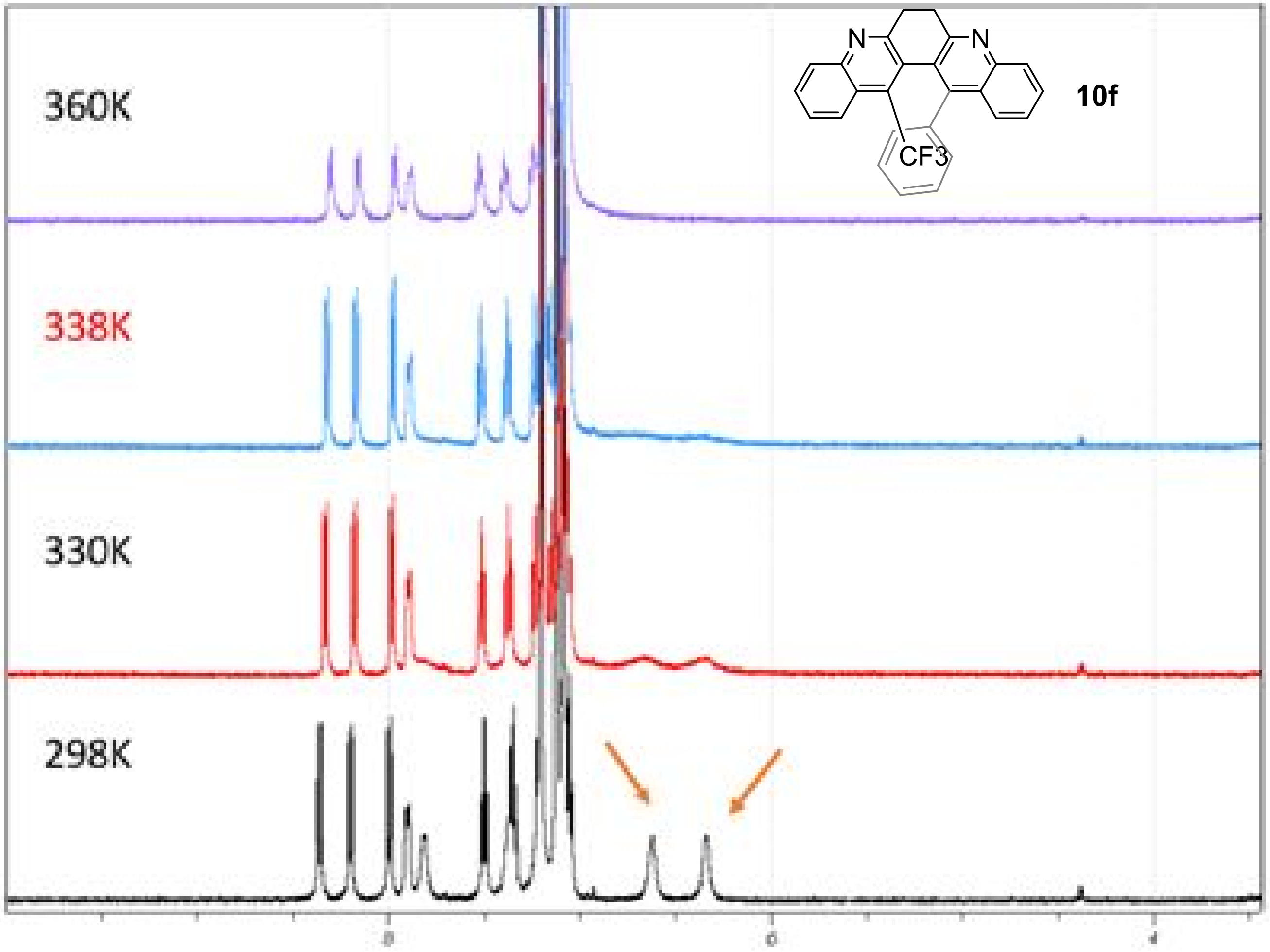

Figure S2-18: Variable temperature experiment to investigate the rotation of phenyl groups in **10f**

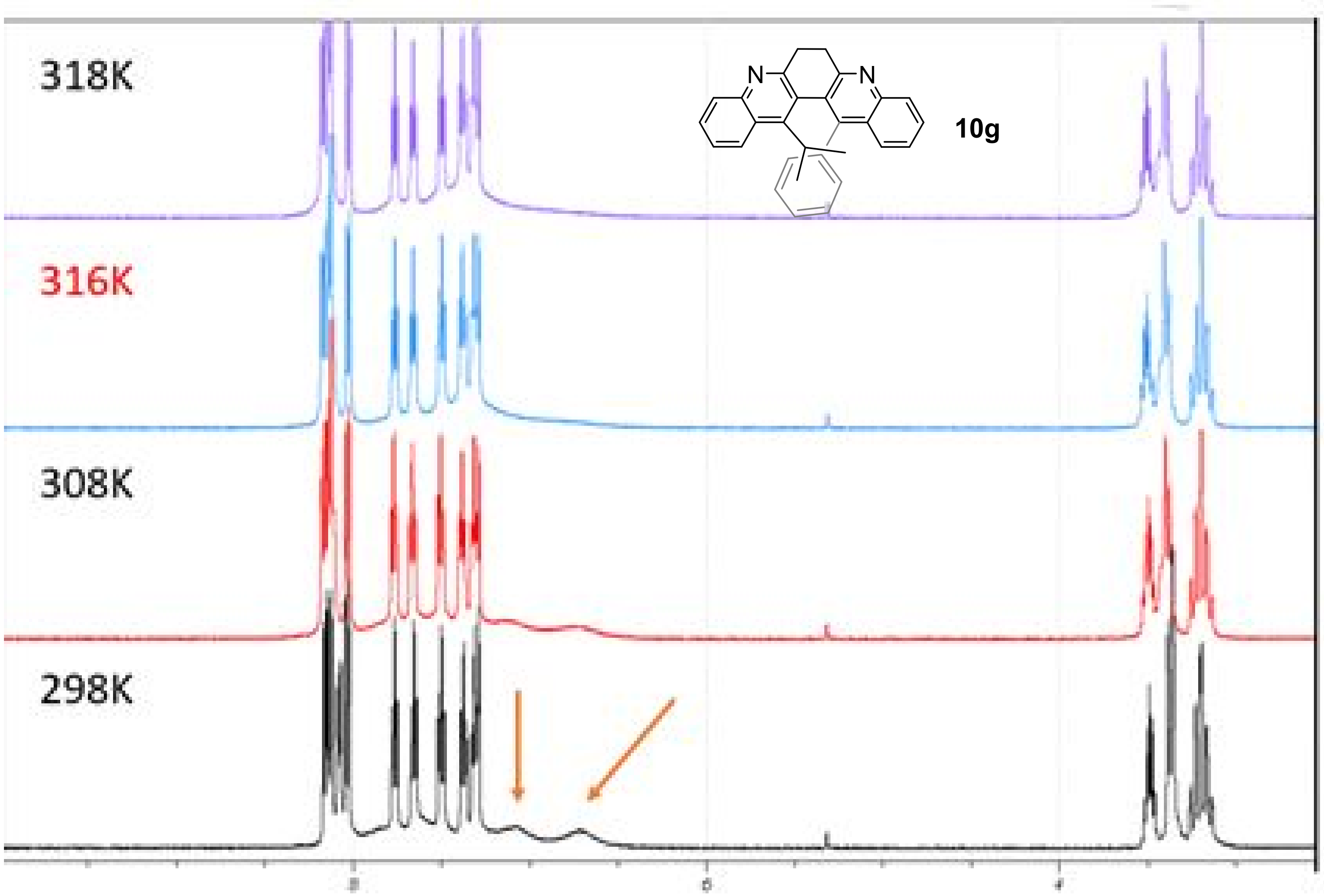

Figure S2-19: Variable temperature experiment to investigate the rotation of phenyl groups in **10g**

	Coalescence temperature (k)	Rotation barrier (kcal/mol)		Coalescence temperature (k)	rotation barrier (kcal/mol)
**2**	297	13.94	**10a**	298	13.95
**5a**	281	13.35	**10c**	318	14.34
**5b**	298	14.27	**10e**	323	14.99
**5c**	308	14.33	**10f**	338	15.62
**6b**	300	14.29	**10g**	316	16.78
**6c**	273	13.31			

Table S2-2: Rotational barriers and coalescence temperatures for selected bay region substituted dibenzo phenanthroline derivatives

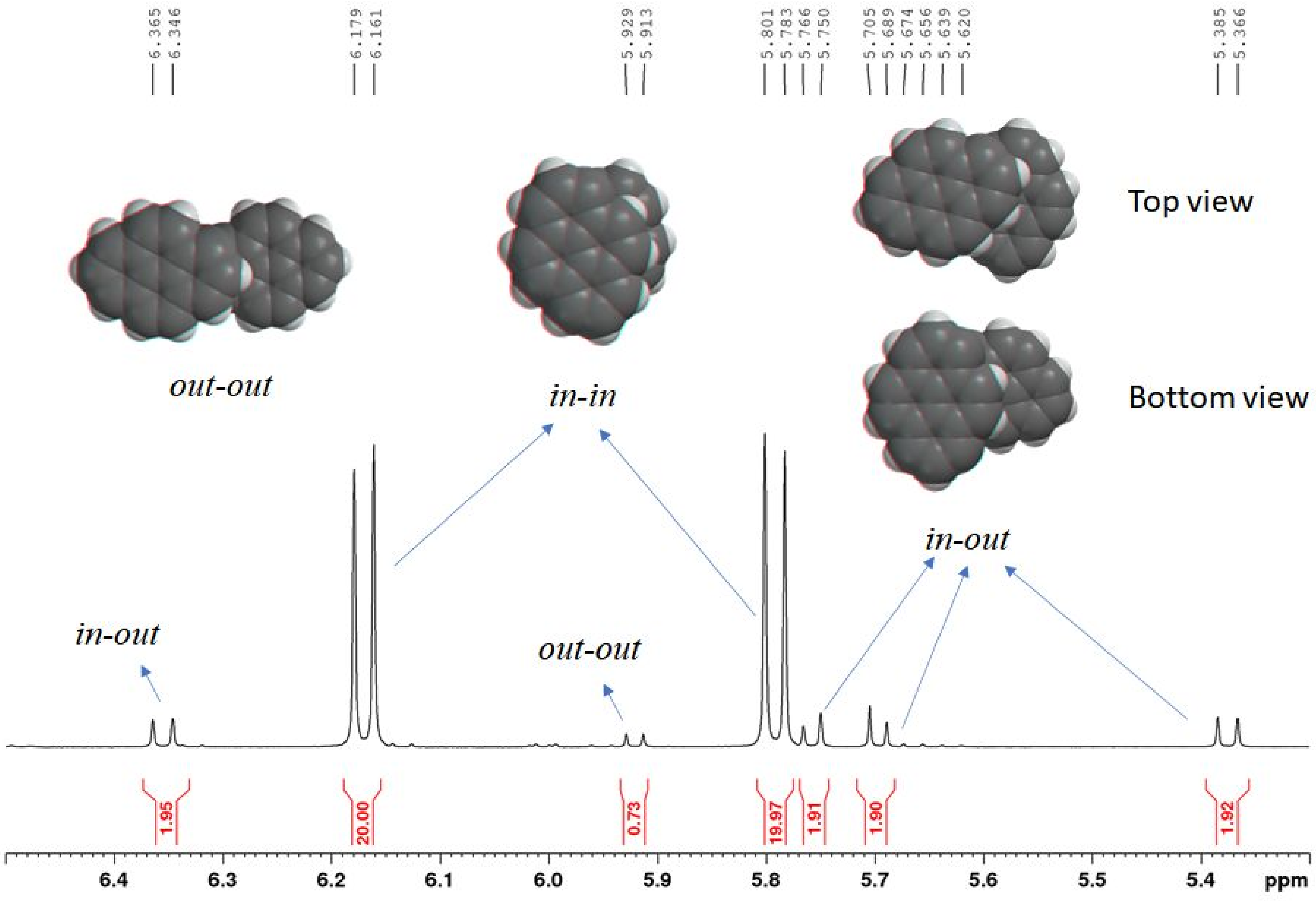

Figure S2-20: Assignment of NMR signals in the shielded region (5.00-6.50 ppm) to rotamers of **5l**

Chiral HPLC graphic of compound 5l

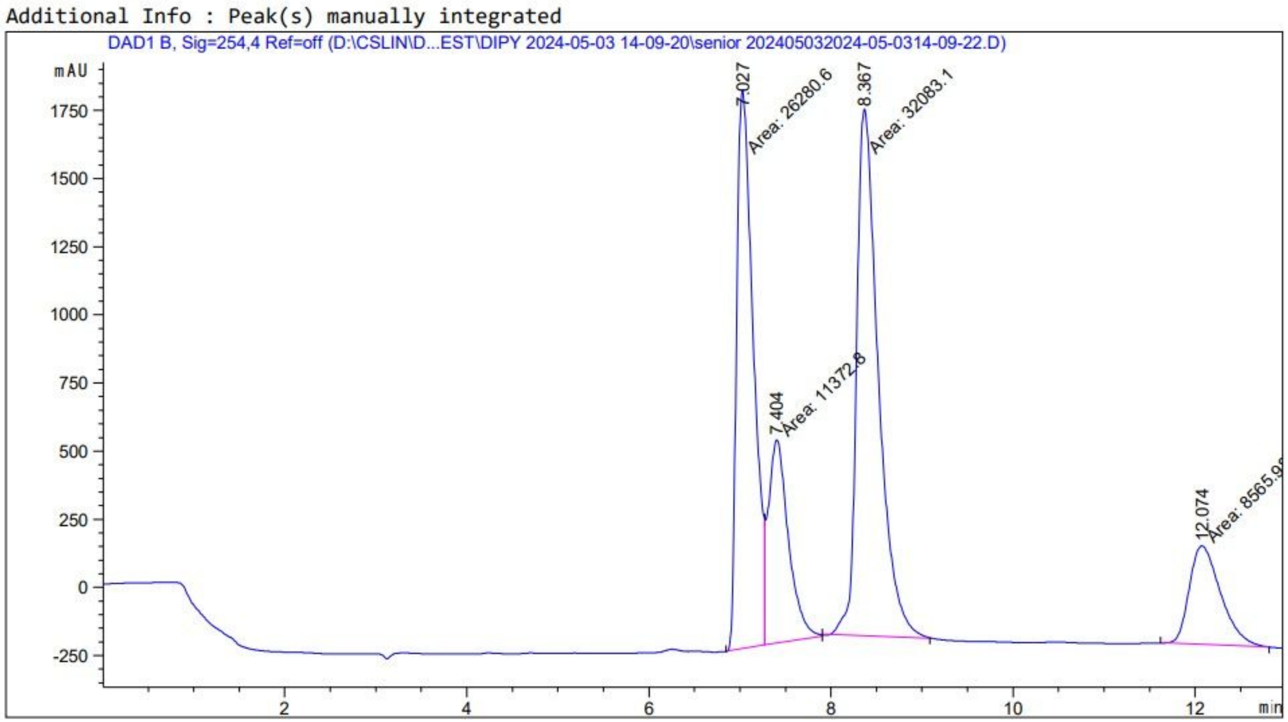

Figure S2-21: The HPLC analysis of **5l** was performed with CHIRALPAK IE column (D = 4.6 mm, L= 250 mm)
The mobile phase is a mixture of 3:7 hexane/ethyl acetate. The ratio is adjusted to 1:1 hexane/ethyl acetate after 10 min.
The flow rate was set to 1 mL/min.

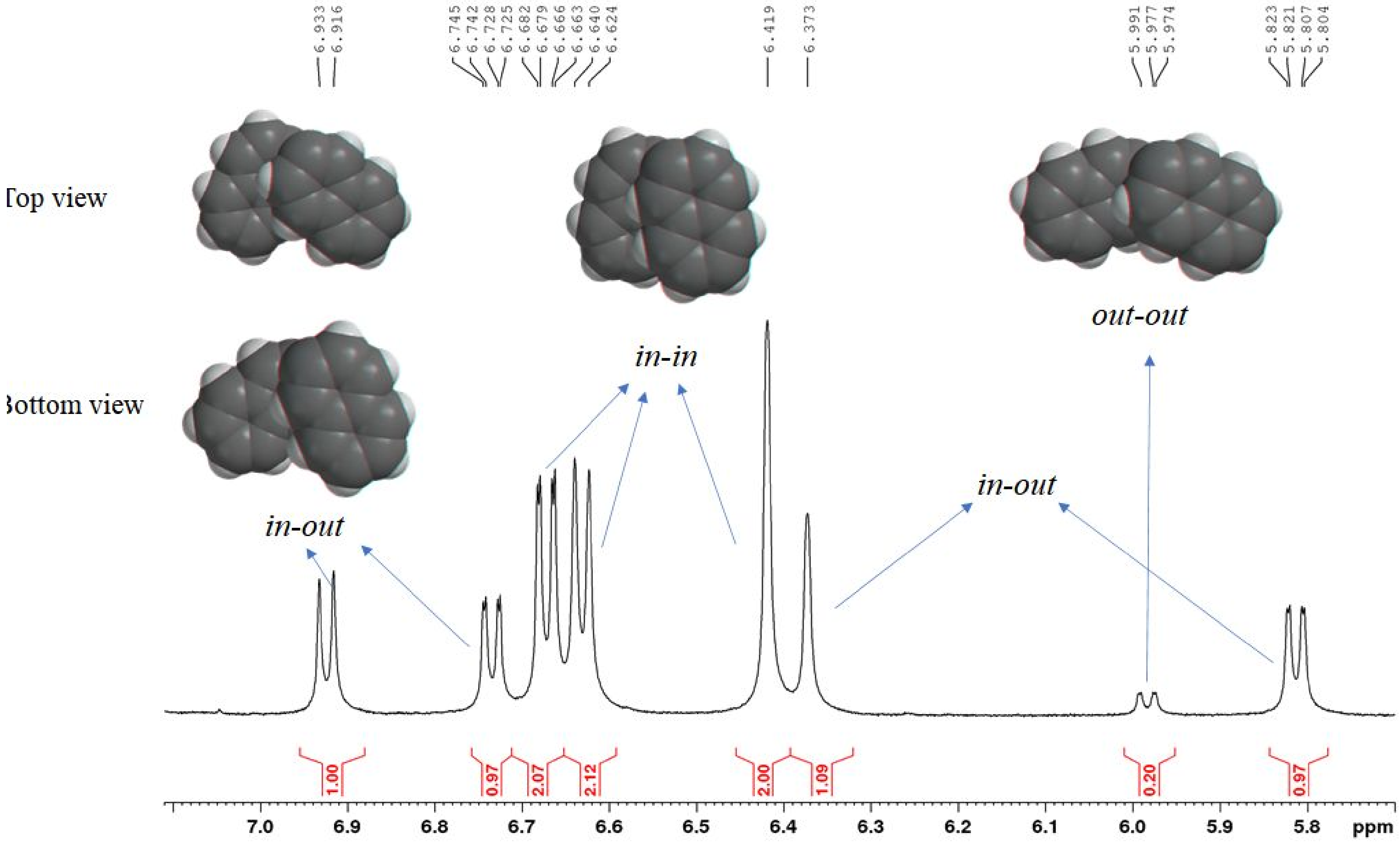

Figure S2-22: Assignment of NMR signals in the shielded region (5.70-7.10 ppm) to rotamers of **5k**
